# Electroacupuncture attenuates ischemic injury after stroke and promotes angiogenesis via activation of EPO mediated Src and VEGF signaling pathways

**DOI:** 10.1371/journal.pone.0274620

**Published:** 2022-09-15

**Authors:** Lifen Wang, Gang Sheng, Jinjun Cui, Yanling Yao, Xue Bai, Fan Chen, Wei Yu

**Affiliations:** 1 Shaanxi Academy of Traditional Chinese Medicine, Shaanxi Provincial Hospital of Chinese Medicine, Xi’an, China; 2 Department of Neurology, Hebei Cangzhou Hospital of Integrated Traditional Chinese Medicine and Western Medicine, Cangzhou, China; 3 College of Acupuncture-Moxibustion and Massage, Shaanxi University of Chinese Medicine, Xian yang, China; 4 Department of Physiology, Xi’an Medical University, Xi’an, China; Eötvös Loránd Research Network Biological Research Centre, HUNGARY

## Abstract

Although electroacupuncture (EA) has been shown to be effective in the treatment of stroke, its mechanisms of action remain undefined. This study explored the therapeutic effects of EA in rats with cerebral ischemia-reperfusion injury (CIRI) and evaluated its possible mechanisms in promoting angiogenesis. To evaluate the effect of EA, we used 2, 3, 5-Triphenyl-2H-Tetrazolium Chloride (TTC) staining and behavior score to calculate the cerebral infarct volume and neurological deficit score after CIRI. Western blot (WB) analysis was employed to evaluate the expression of cluster of differentiation 34 (CD34), erythropoietin (EPO), vascular endothelial growth factor (VEGF) and phospho-Src (p-Src) in the brain of the rats with CIRI. On the other hand, we established an oxygen-glucose deprivation/reoxygenation (OGD/R) injury model using brain microvascular endothelial cells (BMECs), and analyzed cell viability and expression of VEGF or p-Src using cell counting kit-8 (CCK-8) and WB, respectively. Our data showed that EA at the GV26 acupoint could significantly promote the expression of CD34, EPO, VEGF and p-Src in CIRI rats. Our CCK-8 results demonstrated that intervention with recombinant EPO and VEGF proteins remarkably improved the viability of BMECs after OGD/R, while a Src inhibitor, PP1, reversed this phenotype. The WB results showed that the recombinant EPO protein increased the expression of VEGF and p-Src, which was significantly inhibited by PP1. Taken together, our findings showed that EA at the GV26 acupoint can significantly attenuate ischemic injury after stroke and promote angiogenesis via activation of EPO-mediated Src and VEGF signaling pathways. Besides, the upregulation of VEGF may also be associated with the activation of Src by EPO.

## Introduction

Stroke, the second leading cause of death worldwide, is characterized by high morbidity, high mortality, high disability and high recurrence rate [1–3]. Ischemic stroke accounts for more than 80% of stroke cases [4]. Although recombinant tissue-type plasminogen activator (tPA) is considered an effective treatment option for ischemic stroke, 3–5% of patients may benefit due to the limitation of treatment time window [5]. Intravascular thrombus removal is another common clinical procedure used to restore cerebral blood flow in patients with ischemic stroke [6, 7]. However, the recovery of cerebral blood flow often leads to reperfusion injury, leaving patients with different degrees of sequelae [8]. This imposes a heavy economic burden on families and society. There is, therefore, an urgent need to develop more effective treatment options and intervention measures for cerebral ischemia-reperfusion injury.

Electroacupuncture (EA) is a novel therapy based on a combination of traditional acupuncture and modern electrotherapy. EA has been shown to improve brain injury and neurological function recovery after cerebral ischemia-reperfusion injury [9, 10]. Other studies have shown that EA can reduce inflammation response and oxidative stress, inhibit apoptosis, promote angiogenesis and regulate autophagy after stroke [11–14]. In addition, Renzhong (GV26) is an acupoint commonly used in the treatment of stroke in China. The acupoint is located on the Du meridian and acts by refreshing the brain and opening the orifices. EA at the GV26 acupoint has been shown to promote angiogenesis, thus reducing the sequelae in stroke patients [14]. However, its underlying mechanisms of action remain unclear.

Angiogenesis does not only increase collateral circulation to mitigate brain injury caused by ischemia, but exert a pivotal role in the recovery of neurological functions after ischemic stroke [14, 15]. The regeneration of vascular structures provide necessary "support" for neurogenesis and synapse formation, and accelerates recovery of neural networks, which play an important role in improving the quality of life of stroke patients [16, 17]. Angiogenesis is regulated by various vascular growth factors, which include classic angiogenesis factors such as vascular endothelial growth factor (VEGF), and non-classical angiogenesis factors such as erythropoietin (EPO). As a multifunctional tissue protective agent, EPO exerts anti-apoptosis, anti-inflammatory, anti-oxidation, angiogenesis and neurotrophic effects [18, 19]. The effects of EPO are mediated by its interaction with EPO receptor (EPOR). Both EPO and EPOR are not only expressed in erythroid cells, but also in neurons, astrocytes, cerebral vascular endothelial cells and cancer cells [20, 21]. Previous data has shown that VEGF is a key cytokine related to differentiation, proliferation, and migration of endothelial cells. When VEGF binds to its receptor (VEGFR), it activates downstream signaling pathways and transmits angiogenetic signals [22]. Similarly, non-receptor tyrosine kinase, Src, has been shown to play an important role in angiogenesis and participates in many biological processes, which include gene transcription, adhesion regulation, and cell proliferation [23–26]. In addition, our previous study showed that increased Src phosphorylation levels confer a protective effect in brain damage caused by ischemia [27]. Although there are reports that EA increases the expression of EPO, VEGF and p-Src in the brain tissue after ischemic stroke [16, 28, 29], data on how the three proteins interact to promote angiogenesis after EA remains unavailable. This study employed *in vivo* and *in vitro* experiments to explore the mechanisms of EA at the GV26 acupoint in CIRI rats by characterizing the relationship between EPO and VEGF in mediating angiogenesis.

## Materials and methods

### Ethics statement

All the experiments were conducted following the principles of the Basel Declaration and recommendations of the Care and Use of Laboratory Animals issued by the Ministry of science and technology of China. The protocol was approved by the Ethics Committee of Shaanxi Academy of Traditional Chinese Medicine.

### Drugs and reagents

2, 3, 5-Triphenyl-2H-Tetrazolium Chloride (TTC, G3005), Cell Counting Kit-8 (CCK-8, CA1210), high-efficiency RIPA tissue/cell lysate (R0010), Bicinchoninic acid protein concentration assay kit (BCA, PC0020), Tris-glycine running buffer (T1070) and Western blot transfer buffer (D1060) were purchased from Solarbio (Beijing, China). Sodium dodecyl sulfate-polyacrylamide gel electrophoresis (SDS-PAGE) protein loading buffer was purchased from Beyotime (P0015L, Shanghai, China). Pierce™ enhanced chemiluminescence (ECL) Western blotting substrate (32109) was acquired from Thermo (New York, United States), while polyvinylidene difluoride (PVDF) blotting membrane was purchased from GE Healthcare life science (Pittsburgh, United States). Src inhibitor (PP1, M2953) and Cabozantinib (XL184, M1757) were obtained from Abmole (Texas, United States). Recombinant VEGF protein (50159-MNAB) and erythropoietin protein (51099-M08H) were purchased from Sino Biological (Beijing, China). Anti-CD34 antibody (bs-0646R) and anti-VEGF antibody (bs-1313R) were purchased from Bioss (Beijing, China), while anti-EPO antibody (sc-5290) and anti-p-Src antibody (sc-166860) were acquired from Santa Cruz Biotechnology (Texas, United States).

### Experimental animals

Healthy male Wistar rats aged 6 ~ 8 weeks, weighing 220 ± 20 g, were provided by the experimental animal center of Xi’an Jiaotong University. The animals were kept in an environment with a temperature of 22°C ± 2°C and a humidity of 40% ± 5%, The animals were maintained a light dark cycle of 12 hours.

### Middle cerebral artery occlusion (MCAO) model

The rats were anesthetized with 1.5% isoflurane after intraperitoneal injection of tramadol (10 mg/kg). A cut of approximately 2 cm was made in the middle of the rat’s neck, and then the right common carotid artery (CCA), internal carotid artery (ICA) and external carotid artery (ECA) were separated. The CCA and ICA were clamped with a micro artery clamp, while the distal end of ECA was fastened with a sterile 6–0 suture and then disconnected with a single electrode burning pen. The free ECA was placed in a parallel position with the CCA, and then a "V"-shaped cut was made on the ECA using microscissors. Next, a silicone-coated nylon monofilament (Jialing Biotechnology Co., Ltd., Guangzhou, China) was inserted into the ECA and gently pushed into the ICA to block the right middle cerebral artery (MCA). After 1.5 hours of ischemia, blood flow was restored by pulling out the monofilament and removing the arteriole clamp. A temperature-controlled heating pad was used during and after the operation to maintain the body temperature of the rats. There was no operation performed in the control group, while the sham group underwent all the steps as in the study group except that no monofilament was inserted. The MCAO rats were randomly divided into model group and EA group for subsequent experiments.

### Electroacupuncture stimulation

The CIRI rats in the EA group were stimulated by EA. Briefly, a 0.5mm acupuncture needle was used to obliquely puncture the Renzhong point (GV26), a traditional Chinese acupuncture, with a 2mm depth, and connected it with the positive pole of the EA instrument. Thereafter, a ground electrode was placed at about 2mm below the GV26 and a needle was obliquely inserted. All the rats in the EA group were continuously stimulated with 15 Hz and 1 mA for 20 min.

### Measurement of cerebral infarct volume

The rats were anesthetized with isoflurane and then euthanized by cervical dislocation. Thereafter, brain tissues of the euthanized MCAO rats were removed at 3 h, 12 h, 24 h, 3 days and 7 days of reperfusion. Six coronal slices of brain tissue were made at an interval of 2 mm and then placed in 2% TTC solution before incubation at 37°C in the dark for 20 min. The cerebral infarction area was calculated using Image J analysis software. Cerebral infarct area rate (%) = infarct area/total area of sections × 100%.

### Neurological deficit score

To evaluate the neurological deficit, the rats in each group were scored according to the Bederson score [30], at the five time points. The score sheet was as follows: 0, walking normally; 1, forelimb flexion; 2, decreased resistance to lateral push without circling; 3, reduced lateral pushing resistance and circling; 4, hemiplegia or inability.

### Cell culture

Mouse brain microvascular endothelial cell line, bEnd.3, acquired from Shanghai Zhongqiao Xinzhou Biotechnology Co., Ltd., was cultured in Dulbecco’s modified eagle medium (DMEM), supplemented with 1% penicillin-streptomycin and 10% fetal bovine serum (FBS). The cells were kept in a 37°C incubator with 95% air and 5% CO_2_.

### Establishment of oxygen-glucose deprivation/reoxygenation (OGD/R) model

The bEnd.3 cells were inoculated in 96-well plates at a density of 6000/well. After the cells adhered to the wall, the complete culture medium was replaced with glucose-free DMEM and then the cells were cultured in a hypoxic chamber containing 95% N_2_ and 5% CO_2_ at 37°C to achieve oxygen-glucose deprivation (OGD). After 1.5h of OGD, reoxygenation was performed for 24 h to achieve OGD/R injury.

To define the relationship between EPO, VEGF and p-Src, the bEnd.3 cells were divided into five groups, which included control, OGD/R, EPO (40 ng/mL), EPO+PP1 (40 ng/mL+5 μM) and EPO+XL184 (40 ng/mL+0.1 μM) groups.

### Cell viability assay

After OGD, the bEnd.3 cells in 96-well plates were divided into 6 groups, which included the control group, OGD/R group, VEGF (50 ng/mL) group, VEGF+PP1 group (50 ng/mL+5 μM), EPO (40 ng/mL) group and EPO+PP1 group (40 ng/mL+5 μM). During reoxygenation, the culture medium in the control group and OGD/R group was replaced with new complete medium, while the other groups received complete medium containing different drugs. After 24 h of culture, CCK-8 solution (10 μL/100 μL) was added to each well and incubated at 37°C for 4 h. Absorbance at 450 nm was measured using a microplate reader. The cell viability of the bEnd.3 cells was evaluated by the measured optical density (OD) values. Each group was set in triplicate, and the experiment was repeated three times.

### Western blotting

The brain tissues in each group were lysed and then the protein concentration in the supernatant was determined using the BCA kit. Thereafter, SDS-PAGE protein loading buffer was added to each sample and heated in a metal heating block at 95°C for 10 min. The protein samples were resolved in SDS-PAGE and then transferred to PVDF membranes. The membranes were then blocked with 5% protein blocking solution for 2 h. Next, the membranes were incubated with primary antibodies (CD34, 1:1000; EPO, 1:1000; VEGF, 1:1000; p-Src, 1:1000; GAPDH, 1:3000) at 4°C overnight, followed by incubation with corresponding secondary antibodies (1:4000) for 2 h at room temperature. The membranes were washed 3 times with Tris-buffered saline-tween 20 (TBS-T) and then processed using the ECL Western blotting substrate before scanning in a gel imaging system. The CD34, EPO, VEGF and p-Src protein expression levels were analyzed using the ImageJ software. Similarly, the effect of EPO on the expression of VEGF and p-Src in the bEnd.3 cells after OGD/R was also analyzed.

### Statistical analysis

All the data were analyzed using GraphPad Prism 7 software (GraphPad Software, Inc., La Jolla, CA, United States). Data were expressed as mean ± SEM. Comparison between the two groups was carried out using the Student’s two-tailed t-test, while one-way analysis of variance (ANOVA) was used for analysis of multiple groups. Post hoc tests were then performed using Dunnett’s t-test. *P*<0.05 was considered statistically significant.

## Results

### Effect of EA on infarct volume in CIRI rats

**Fig 1:**
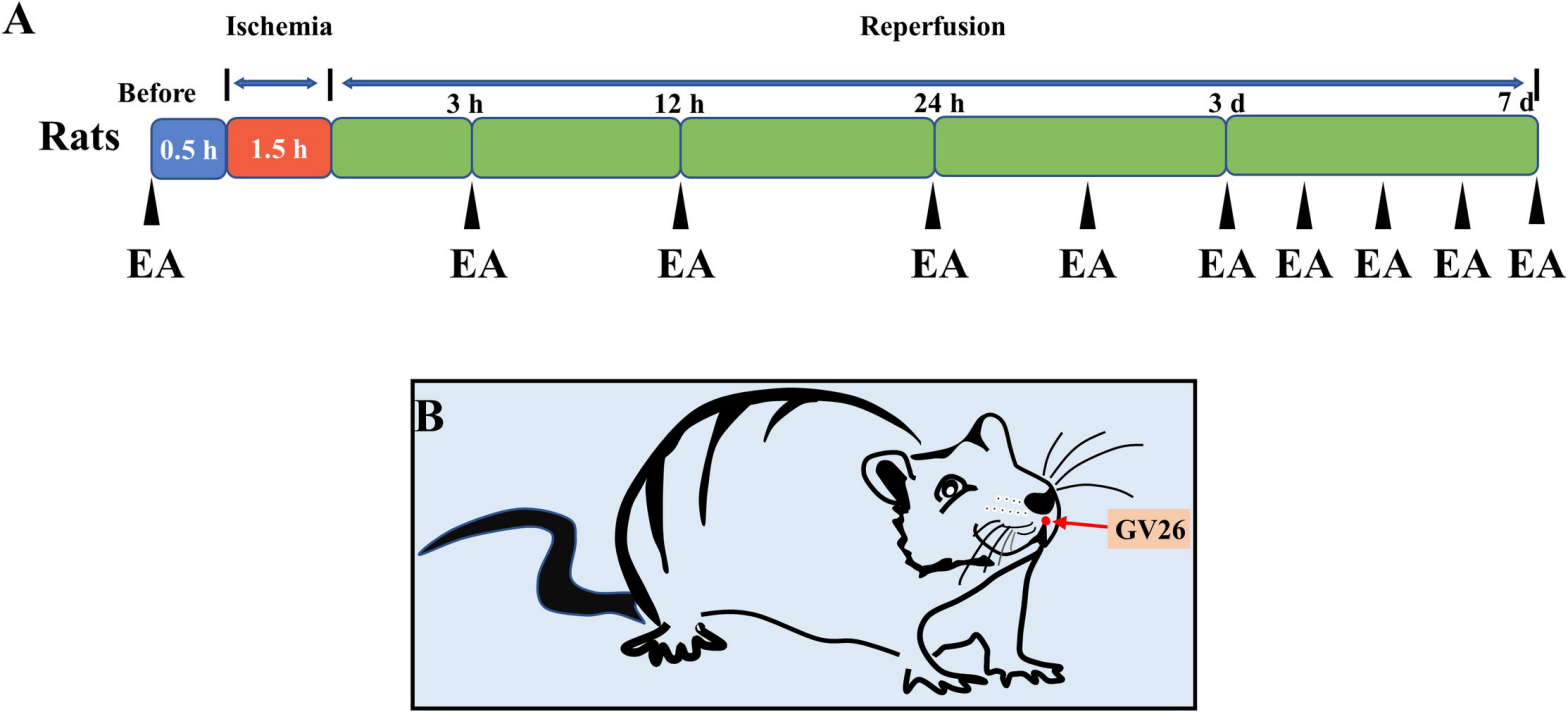
Experimental scheme. (A) Neurological deficit scores, TTC staining, and follow-up experiments were performed on rats in each group at 3 h, 12 h, 24 h, 3 d, and 7 d after operation. Rats in the EA group were treated 30 min before surgery and 3 h, 12 h or 24 h after reperfusion. Thereafter, the EA group received EA at GV26 acupoint once a day. (B) Schematic diagram showing the GV26 acupoint location.

To evaluate the effect of EA at GV26 on CIRI rats, the rats were randomly divided into 4 groups, which included the control group, sham group, model group and EA group. The rats in the control group did not undergo any surgery, while those in the sham group underwent surgery but did not suffer from cerebral ischemia-reperfusion injury (CIRI). Whereas the rats in the model group and the EA group were subjected to CIRI, those in the EA group underwent EA 30 min before the operation, and 3 h, 12 h, and 24 h after the recovery of cerebral blood flow (Fig 1). Afterwards, the rats in the EA group received EA once a day (Fig 1).

**Fig 2:**
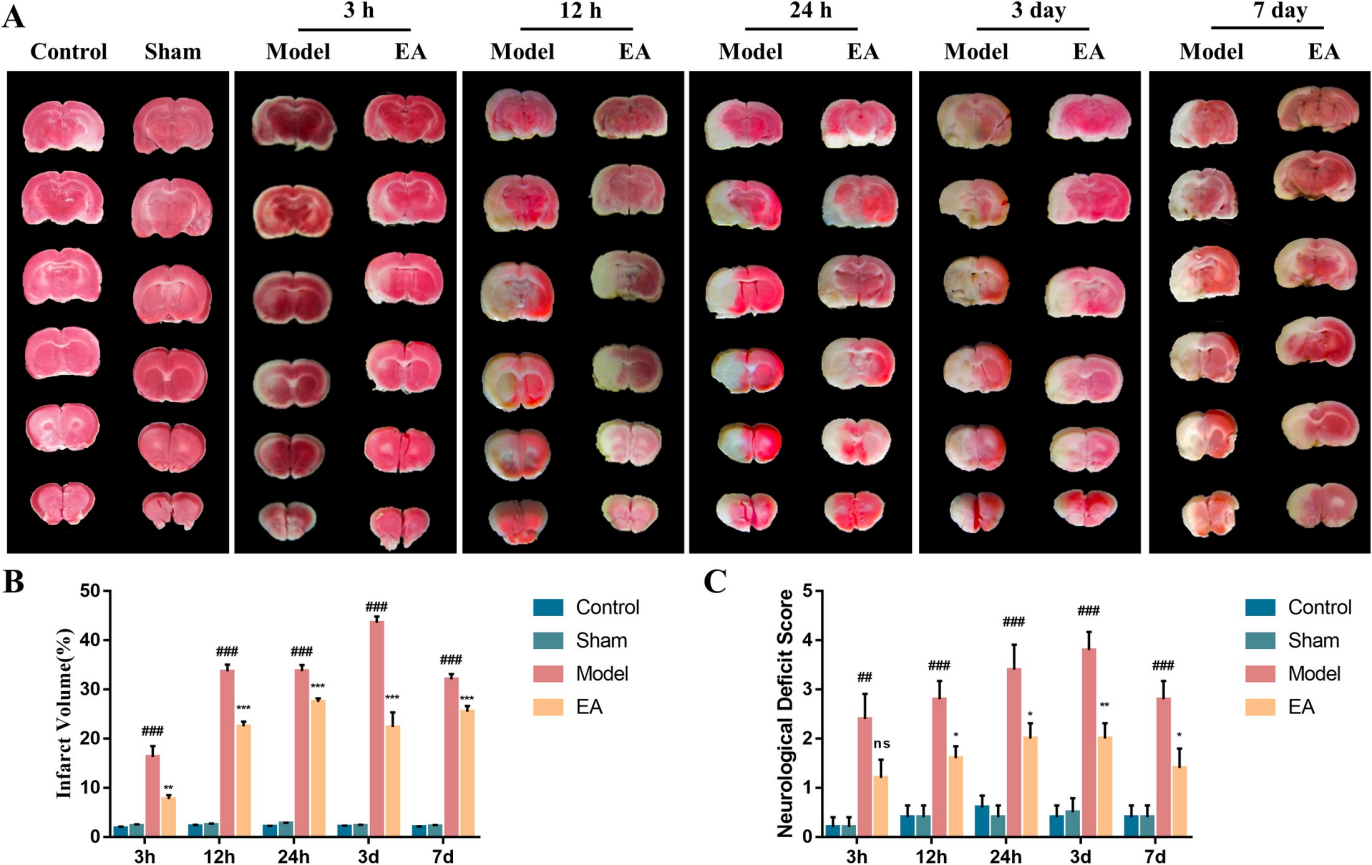
EA ameliorates infarct volume and neurological deficit score in CIRI rats. (A) Representative images of the TTC staining at different time points in the control, sham, model and EA groups. After TTC staining, red represented normal tissue, while white showed the infarcted area. (B) Quantification of infarct volume in each group (n = 5). (C) Quantification of neurological deficit score (n = 5). Data were expressed as mean ± SEM. ^##^*P* < 0.01, ^###^*P* < 0.001 vs. sham group; *P < 0.05, **P < 0.01, ***P < 0.001 vs. model group.

To assess the effect of EA at GV26 on the cerebral infarct volume of the CIRI rats, the brain tissues of rats in each group were stained with TTC at 3 h, 12 h, 24 h, 3 d and 7 d after reperfusion (Fig 1). As shown in Fig 2, the surgical injury without CIRI did not cause cerebral infarction in rats compared with those in the control group (*P*>0.05). Compared with the sham group, there was significant increase in the cerebral infarction volume of rats in the model group (*P*<0.001), which showed a rising trend at 3 h, 12 h, 24 h and 3 d (Fig 2A and 2B). Besides, EA was shown to obviously reduce the infarct volume of the CIRI rats compared with the model group at various time points (Fig 2A and 2B; *P*<0.01). These findings demonstrate that EA at GV26 significantly improve the brain damage in CIRI rats.

### Effect of EA on neurological deficit in CIRI rats

The rats were scored for neurological deficits after recovery of their consciousness. Compared with the control group, surgical injury without cerebral ischemia did not affect the neurological function of the rats (*P*>0.05), while CIRI substantially increased the neurological deficit score of the rats (Fig 2C; *P*<0.01). Although the EA treatment did not markedly ameliorate the neurological deficit score of the CIRI rats after 3 h of reperfusion, there was significant improvement of effects at subsequent time points (12 h, 24 h, 3 d and 7 d; *P*<0.05). These data indicated that EA at GV26 point could significantly restore the neurological function of CIRI rats.

### Effect of EA on the expression of CD34, EPO, VEGF and p-Src proteins in CIRI rats

**Fig 3:**
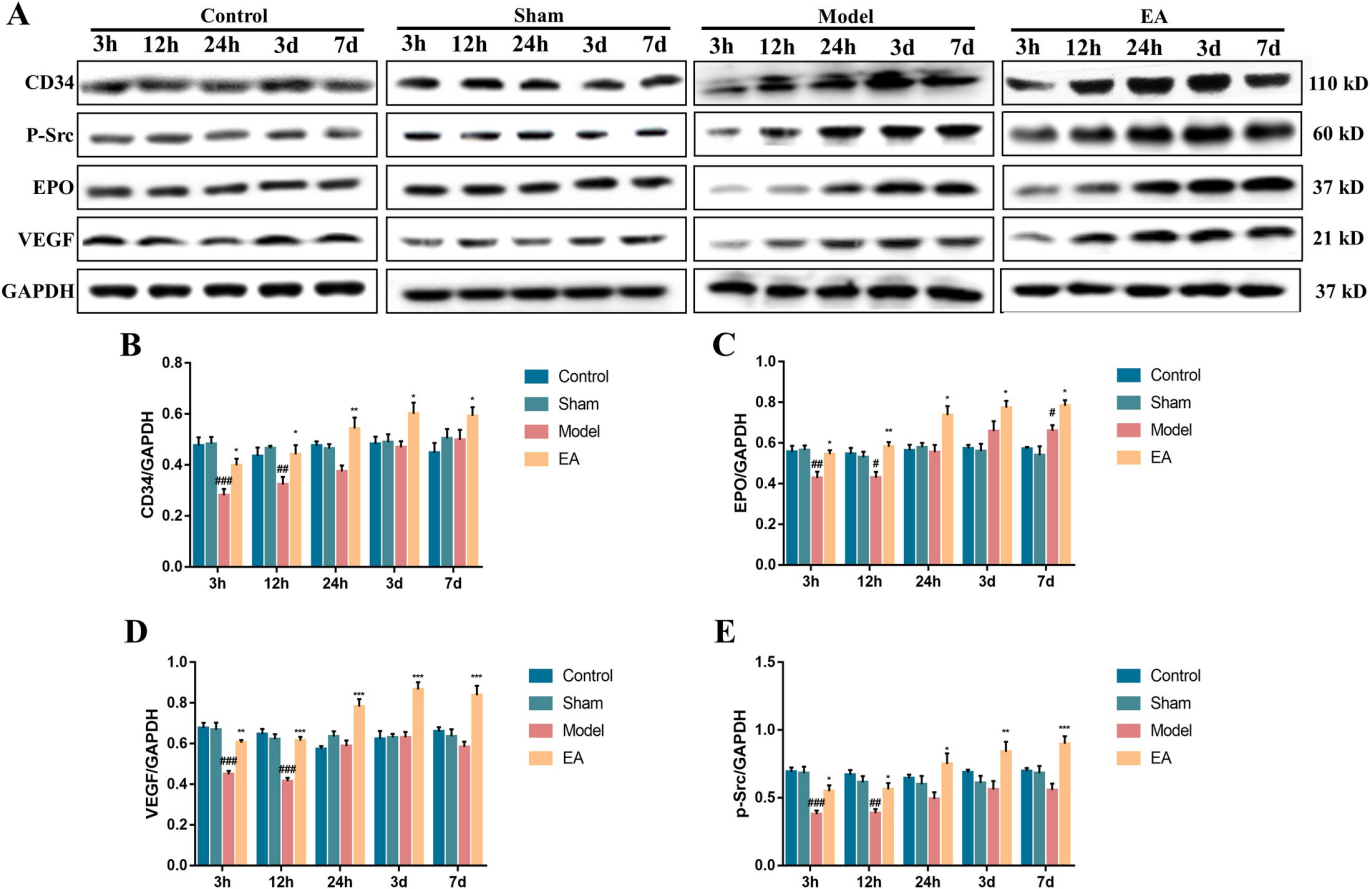
EA improved the expression of CD34, EPO, VEGF and p-Src proteins in the brain tissues of CIRI rats. (A) Representative blots showing the expression of CD34, EPO, VEGF and p-Src at different time points in the control, sham, model and EA groups. (B-E) Quantification of the expression level of CD34, EPO, VEGF and p-Src proteins (n = 5). Data were expressed as mean ± SEM. ^#^*P* < 0.05, ^##^*P* < 0.01, ^###^*P* < 0.001 vs. sham group; *P < 0.05, **P < 0.01, ***P < 0.001 vs. model group.

CD34 protein is often used as a marker of angiogenesis [31, 32]. In this study, angiogenesis in CIRI rats was evaluated by analyzing the expression of CD34 protein in the brain tissue. Compared with the control group, surgical trauma without cerebral ischemic injury did not change the expression of CD34, EPO, VEGF and p-Src in the brain tissues of rats (Fig 3; *P*>0.05). At 3 h and 12 h after reperfusion, the expression of CD34 protein in the brain tissues of CIRI rats was significantly lower than that in the sham group (Fig 3A and 3B; *P*<0.001). However, EA at the GV26 obviously improved the CD34 expression in the brain tissue of CIRI rats compared with that in the model group (Fig 3A and 3B; *P*<0.05). To define potential mechanisms of EA at GV26 on angiogenesis in the CIRI rats, the expression levels of EPO, VEGF and p-Src were analyzed using WB. Compared with the model group, EA at GV26 acupoint remarkably promoted the expression of EPO, VEGF and p-Src in the brain tissue of CIRI rats (Fig 3C–3E; *P*<0.05). These results demonstrated that EA at GV26 point could promote angiogenesis in the CIRI rats, which was associated with increased expression of EPO, VEGF and p-Src.

### Effects of recombinant EPO and VEGF proteins on the viability of bEnd.3 cells after OGD/R

**Fig 4:**
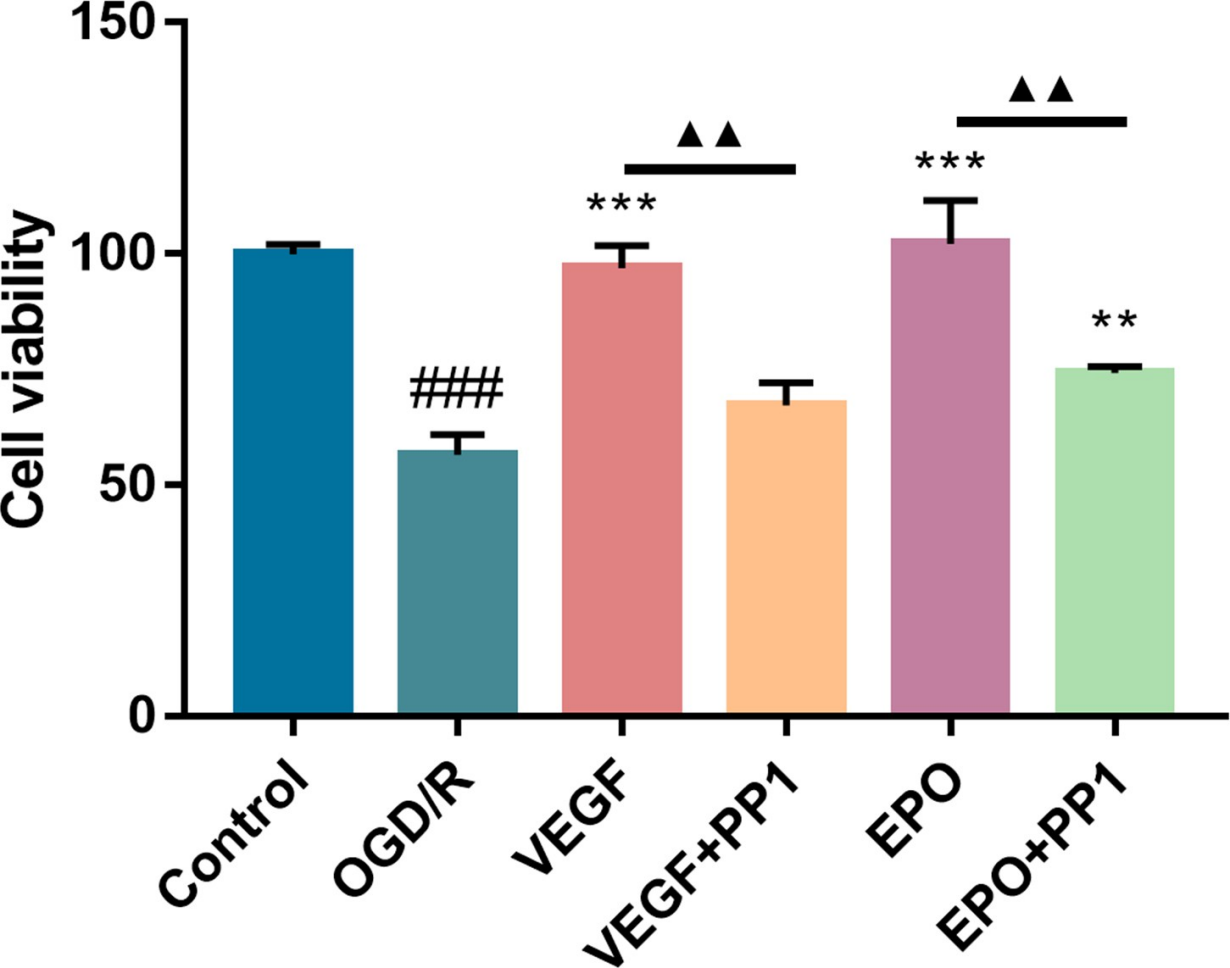
EPO and VEGF proteins enhance the viability of bEnd.3 cells after OGD/R. An oxygen-glucose deprivation/reoxygenation (OGD/R) model was established *in vitro*. The bEnd. 3 cells were divided into 6 groups; control, OGD/R, VEGF, VEGF + PP1, EPO and EPO + PP1 groups. The control group did not experience OGD/R injury. The quantitative results of the cell viability in each group were obtained from three independent experiments. Data were expressed as mean ± SEM. ^###^*P* < 0.001 vs. control group; **P < 0.01, ***P < 0.001 vs. OGD/G group.

Our data has shown that EA at GV26 acupoint promotes angiogenesis in CIRI rats by increasing the expression of EPO, VEGF and p-Src, to reduce brain damage. However, the relationship between EPO, VEGF and p-Src remains unknown. We simulated ischemia-reperfusion injury by inducing OGD/R injury in bEnd.3 cells. After ODG/R, viability of the bEnd.3 cells was significantly suppressed compared with the control group (Fig 4; *P*<0.001). Interestingly, intervention with recombinant EPO or VEGF protein improved the viability of the bEnd.3 cells, while the Src inhibitor PP1 obviously reversed this outcome (Fig 4; *P*<0.01). These data showed that EPO or VEGF can alleviate OGD/R damage in bEnd.3 cells via activating the Src signaling pathway.

### Effect of recombinant EPO protein on the expression of VEGF and p-Src in bEnd.3 cells after OGD/R

**Fig 5:**
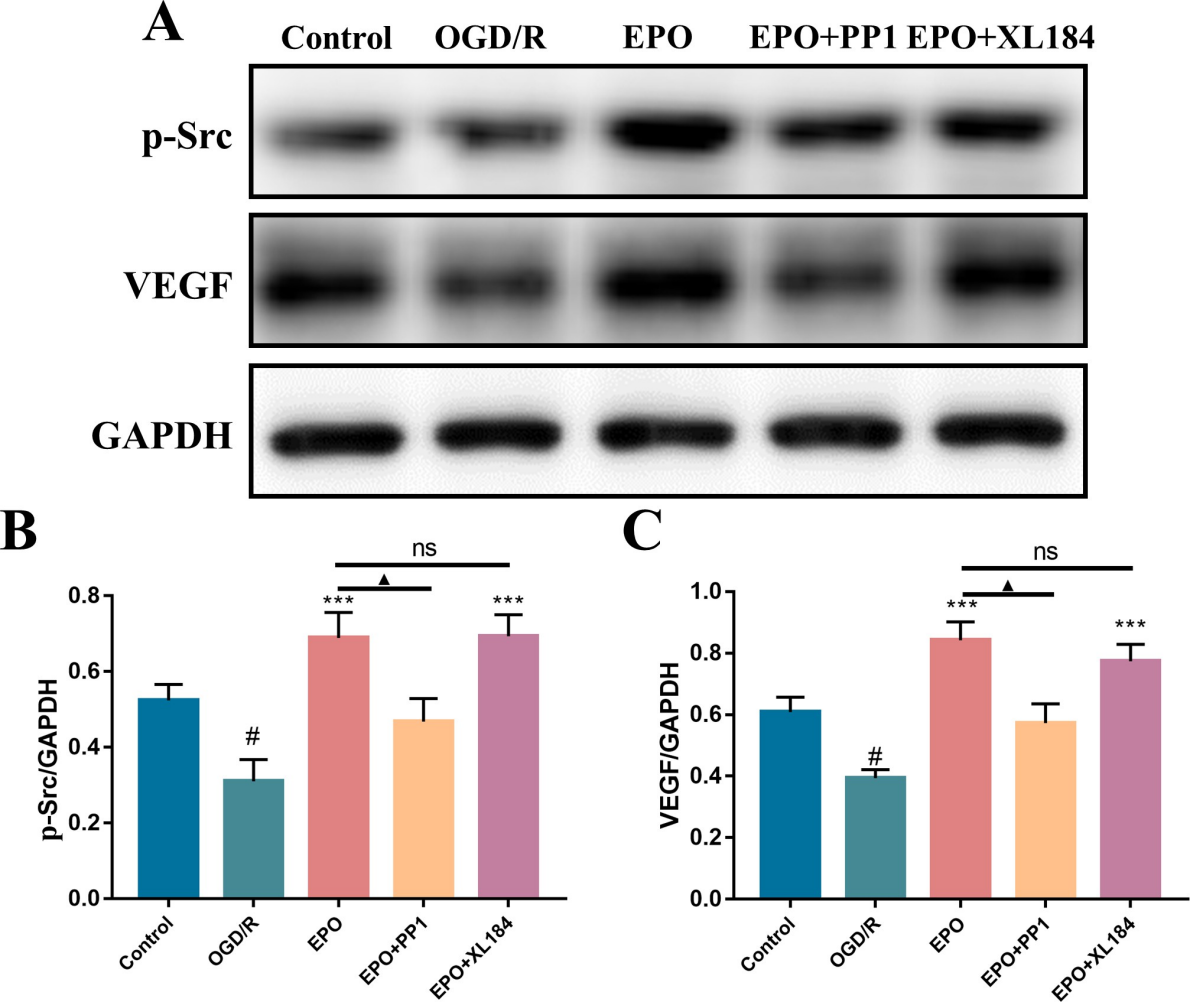
The recombinant EPO protein increased the expression of VEGF and p-Src in bEnd.3 cells after OGD/R. (A) Representative expression of the VEGF and p-Src in the control, OGD/R, EPO, EPO + PP1 and EPO + XL184 groups. (B) Quantification of the expression level of VEGF and p-Src proteins (n = 5). Data were expressed as mean ± SEM. ^#^*P* < 0.05 vs. control group; ***P < 0.001 vs. OGD/G group.

Src kinase plays an important role in angiogenesis and is associated with EPO and VEGF mediated angiogenesis [24, 29, 33–36]. Previous data has demonstrated that EPO can enhance the secretion of VEGF in neural progenitor cells via activation of the phosphatidylinositol 3-kinase/protein kinase B (PI3K/AKT) and extracellular regulated protein kinases1/2 (ERK1/2) signaling pathways, while Src may mediate the expression of VEGF [37–40]. To further assess the relationship among EPO, VEGF and Src, the effect of EPO on the expression of VEGF and p-Src in bEnd.3 cells after OGD/R was analyzed by WB. Compared with the control group, OGD/R injury significantly reduced the expression of VEGF and p-Src in bEnd.3 cells (Fig 5; *P*<0.05). The recombinant EPO protein substantially enhanced the expression of VEGF and p-Src in bEnd.3 cells after OGD/R (Fig 5; *P*<0.001). In addition, the Src inhibitor PP1 effectively inhibited the effect of EPO on VEGF and p-Src, while the VEGFR inhibitor XL184 did not show any significant effect (Fig 5; *P*>0.05). These data demonstrated that the recombinant EPO protein promotes the expression of VEGF in End.3 cells after OGD/R via activation of the Src signaling pathway.

### The EA mechanism in promoting angiogenesis in CIRI rats

**Fig 6:**
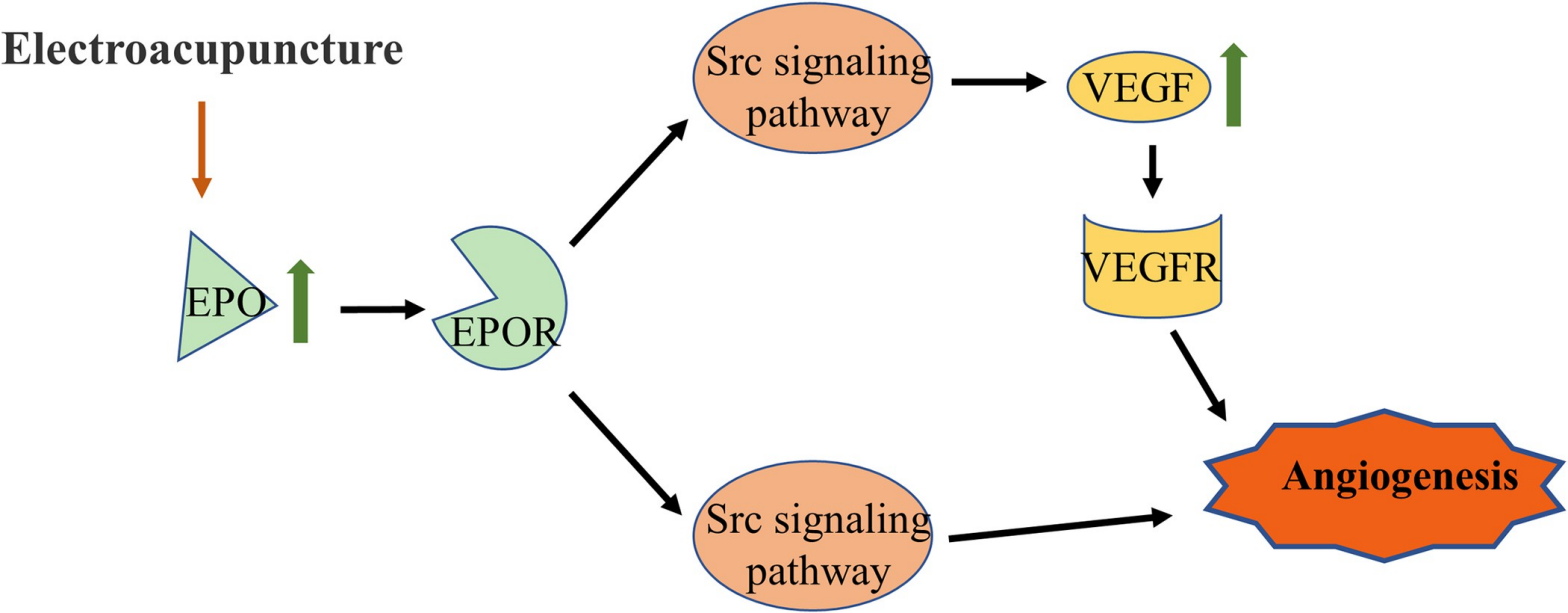
EA activated EPO mediated Src signaling pathway and VEGF signaling pathway to promote angiogenesis in CIRI rat brain tissue. EA at GV26 increased the expression of EPO in the brain tissue of CIRI rats and promoted angiogenesis by activating the downstream Src signaling pathway. In addition, the increased EPO enhanced the pro-angiogenesis effect of EA at GV26 by promoting the expression of VEGF.

Our analysis showed that EA at GV26 acupoint mitigated the ischemia/reperfusion injury of MCAO rats through promotion of angiogenesis in brain tissues. Angiogenesis did not only save the damaged neurons in the penumbra by increasing collateral circulation, but also provided the necessary material basis for neurogenesis and synapse formation which accelerated the recovery of neural function. EA at the GV26 point of MCAO rats promoted angiogenesis, which was associated with increased secretion of EPO and VEGF (Fig 6). The data showed that EA at the GV26 point of the MCAO rats resulted in increased expression of EPO in the brain tissue, which promoted angiogenesis by fueling the VEGF expression and activating downstream Src signaling pathway. Notably, the increased expression of VEGF was related with the activation of Src signaling pathway by EPO.

## Discussion

Although previous studies have shown that EA confers a significant effect on cerebral ischemia/reperfusion injury (CIRI), the effect is influenced by different electrical stimulations and acupoints [41]. The choice of acupoints was the primary factor in EA treatment. GV26, one of the traditional Chinese acupoints, can improve cerebral blood flow, promote angiogenesis, regulate cell apoptosis and autophagy, as well as inhibit inflammation response [14, 42–44]. We used common electrical stimulation parameters for the treatment of stroke (frequency of 15 Hz, current amplitude of 1 mA for 20 min) were used in this experiment [45]. In this study, a rat model of cerebral ischemia/reperfusion injury was established. Thereafter, the cerebral infarction volume, neurological function, and the expression level of CD34, an angiogenesis marker, was analyzed in CIRI rats by TTC staining, neurological defect score and WB. The data showed that EA at GV26 acupoint substantially improved the brain injury of CIRI rats and promoted angiogenesis, which is consistent with previous reports [14].

A previous study demonstrated that high density of new capillaries in the ischemic area is essential in reducing mortality and improving prognosis in patients with ischemic stroke [46]. Although EA at GV26 could significantly promote angiogenesis after stroke, its mechanism remains poorly understood. In this study, we revealed the mechanism of EA at GV26 in promoting angiogenesis through *in vivo* and *in vitro* experiments. *In vivo*, we measured the expression levels of EPO and VEGF, the key regulators of angiogenesis, in the brain tissue of CIRI rats. The results showed that EA at GV26 significantly improved the expression of EPO and VEGF, as well as the expression and phosphorylation level of Src. We then evaluated the interaction among EPO, VEGF and Src proteins in promoting angiogenesis by performing glucose-oxygen deprivation/reoxygenation (OGD/R) experiments on brain microvascular endothelial bEnd.3 cells to simulate cerebral ischemia/reperfusion injury (CIRI). Importantly, the Src inhibitor PP1 has been shown to inhibit phosphorylation of Src induced by transformation of growth factor-β1 (TGF-β1) in renal interstitial fibroblasts and phosphorylation of Src in rat cardiomyocytes in a dependent manner. Besides, the Src inhibitor interferes with the mitotic response of vascular smooth muscle cells to prevent vascular remodeling [47–49]. The inhibitory effect on Src phosphorylation was most significant at a concentration of 5μM PP1 [47, 48]. Therefore, to achieve effective inhibition of Src in bEnd.3 cells, we selected a PP1 dose of 5μM for our analyses. Through the effect of Src inhibitor PP1 on EPO and VEGF, we demonstrate that EA at GV26 acupoint can lead to increased EPO expression, which can promote angiogenesis by activating the downstream Src signal pathway and enhancing VEGF expression.

Previous data reported that recombinant EPO protein could promote the expression and secretion of VEGF, and activate PI3K/AKT and ERK1/2 in neural progenitor cells, while the inhibition of the AKT and ERK1/2 signaling pathways markedly reduced the rhEPO-induced VEGF expression in neural progenitor cells [37]. However, this study showed that the use of PP1 obviously inhibited the expression of VEGF in bEbnd.3 cells, demonstrating that the activation of the Src signaling pathway may be another mechanism for EPO in promotion of VEGF expression. In summary, it can be concluded that EA at GV26 acupoint in CIRI rats could increase the expression of EPO and VEGF in brain tissue and promote angiogenesis by activating the downstream Src signaling pathway. At the same time, our data revealed that EA at GV26 acupoint increases the VEGF expression. In addition to the reported data which showed that EPO activates PI3K/AKT and EPK1/2 signaling pathways to promote VEGF expression, EPO was also shown to fuel VEGF expression via activating the Src signaling pathway in vascular endothelial cells. Although our work had revealed the mechanism of promoting angiogenesis and VEGF expression after EA at GV26 acupoints, the mechanism underlying the increased EPO expression remained unknown. However, there is a need to explore the mechanism of EA in promoting vascular growth factors such as EPO, to reveal the exact scientific value of traditional Chinese acupoints.

## Conclusion

Taken together, our data demonstrated that EA at GV26 acupoint improves cerebral infarction volume and neurological deficit score of CIRI rats by promoting brain tissue angiogenesis. We further revealed that EA at GV26 point may promote angiogenesis via EPO-mediated activation of the Src signaling pathway and VEGF signaling pathway. Besides, the data showed that the activation of Src signaling pathway by EPO contributed to the increase in the expression of VEGF.

## Supporting information

S1 File The data set. Raw data of relevant experiments in the manuscript.
(XLSX)

S2 File Blot results. Original picture of the western blot experiments in the manuscript.
(ZIP)
