# Supplementary figures and images for: Electroacupuncture attenuates ischemic injury after stroke and promotes angiogenesis via activation of EPO mediated Src and VEGF signaling pathways (part 2 of 2)
Source: PLoS One. 2022 Sep 15;17(9):e0274620. doi: 10.1371/journal.pone.0274620 (PMC9477374; doi:10.1371/journal.pone.0274620)

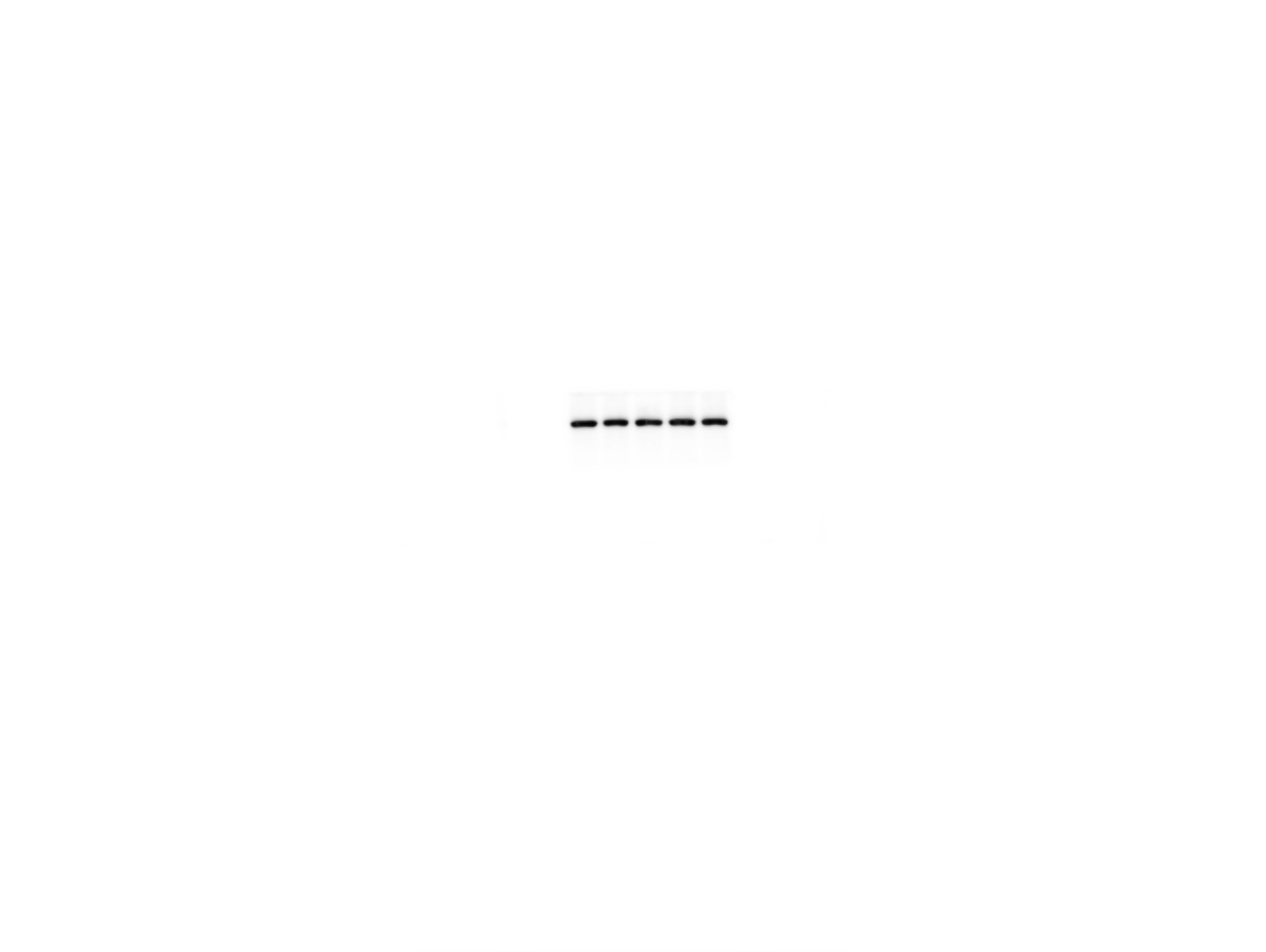

Supplement: S2 File — Original picture of the western blot experiments in the manuscript. (ZIP) [file pone.0274620.s002.zip › S2. blot results/Fig 5/GAPDH/1.tif]

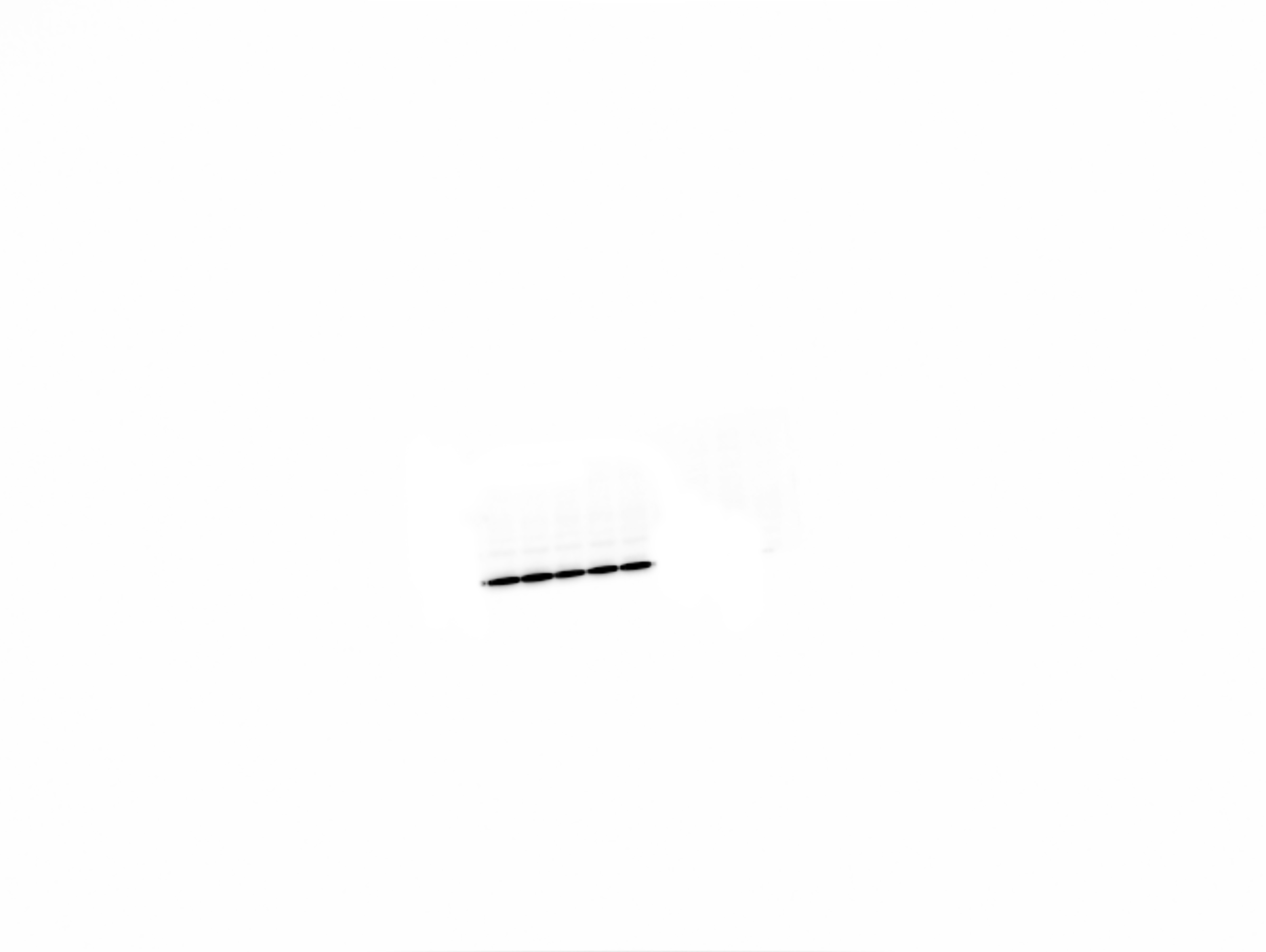

Supplement: S2 File — Original picture of the western blot experiments in the manuscript. (ZIP) [file pone.0274620.s002.zip › S2. blot results/Fig 5/GAPDH/2.tif]

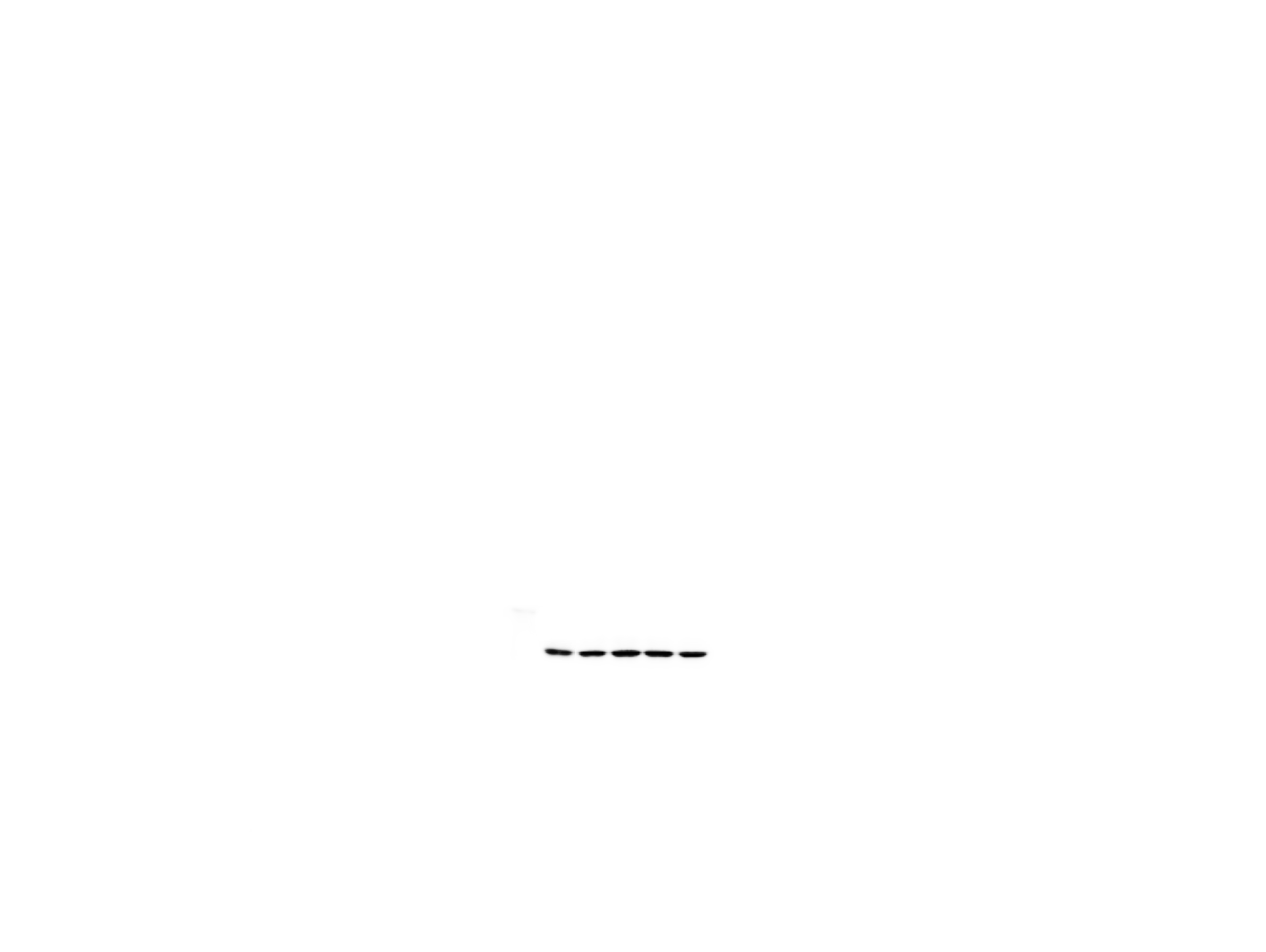

Supplement: S2 File — Original picture of the western blot experiments in the manuscript. (ZIP) [file pone.0274620.s002.zip › S2. blot results/Fig 5/GAPDH/3.tif]

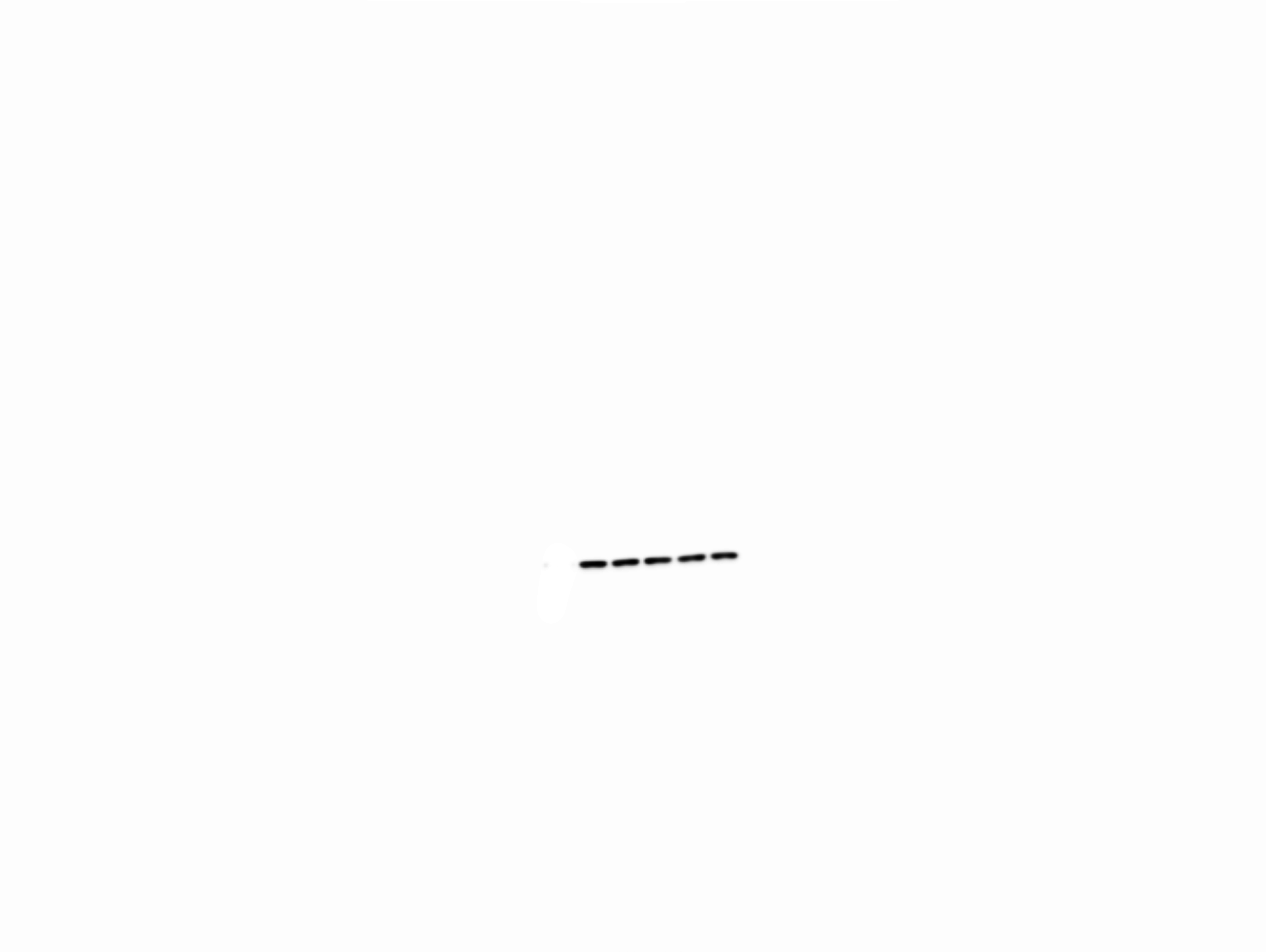

Supplement: S2 File — Original picture of the western blot experiments in the manuscript. (ZIP) [file pone.0274620.s002.zip › S2. blot results/Fig 5/GAPDH/4.tif]

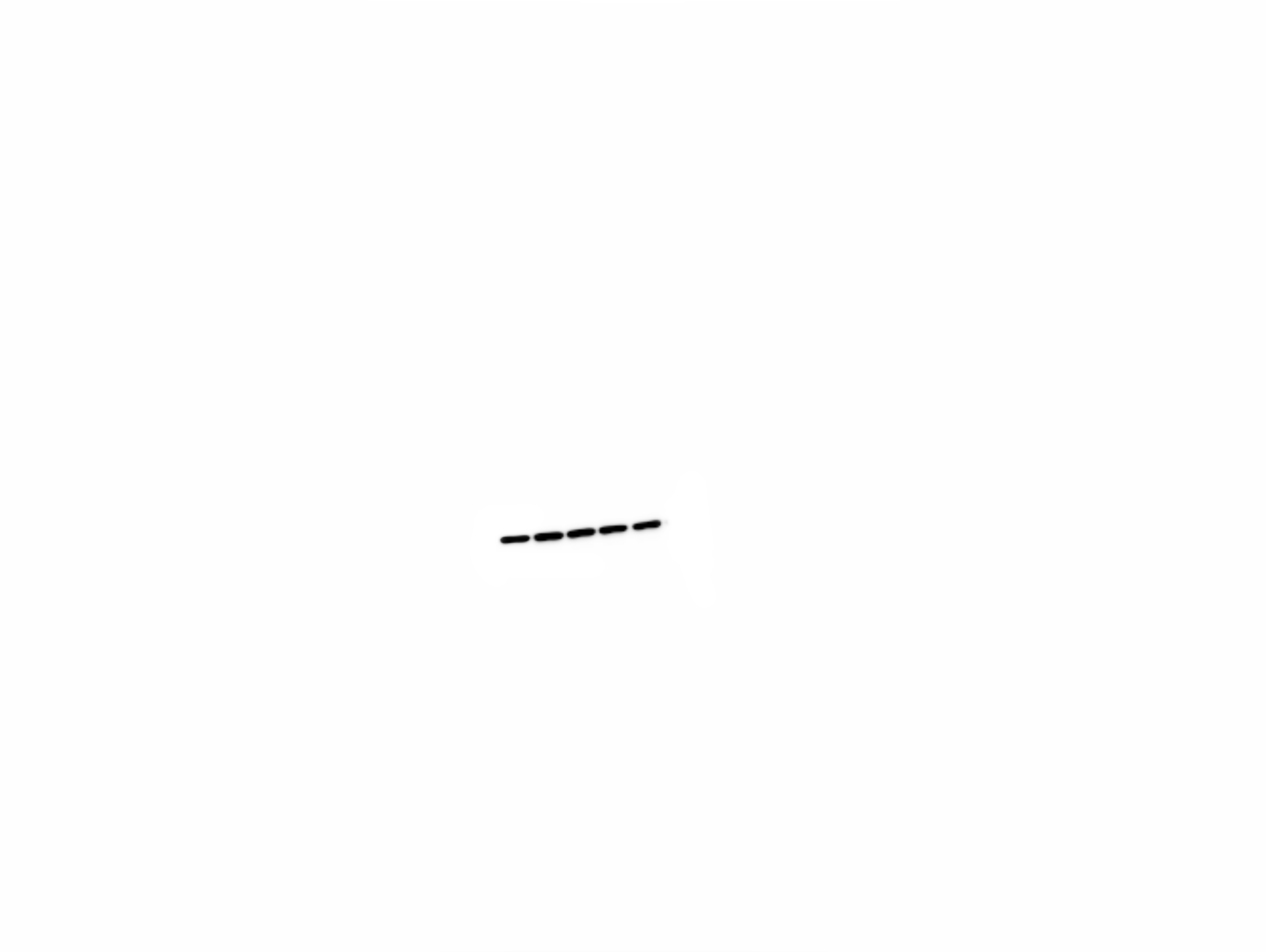

Supplement: S2 File — Original picture of the western blot experiments in the manuscript. (ZIP) [file pone.0274620.s002.zip › S2. blot results/Fig 5/GAPDH/5.tif]

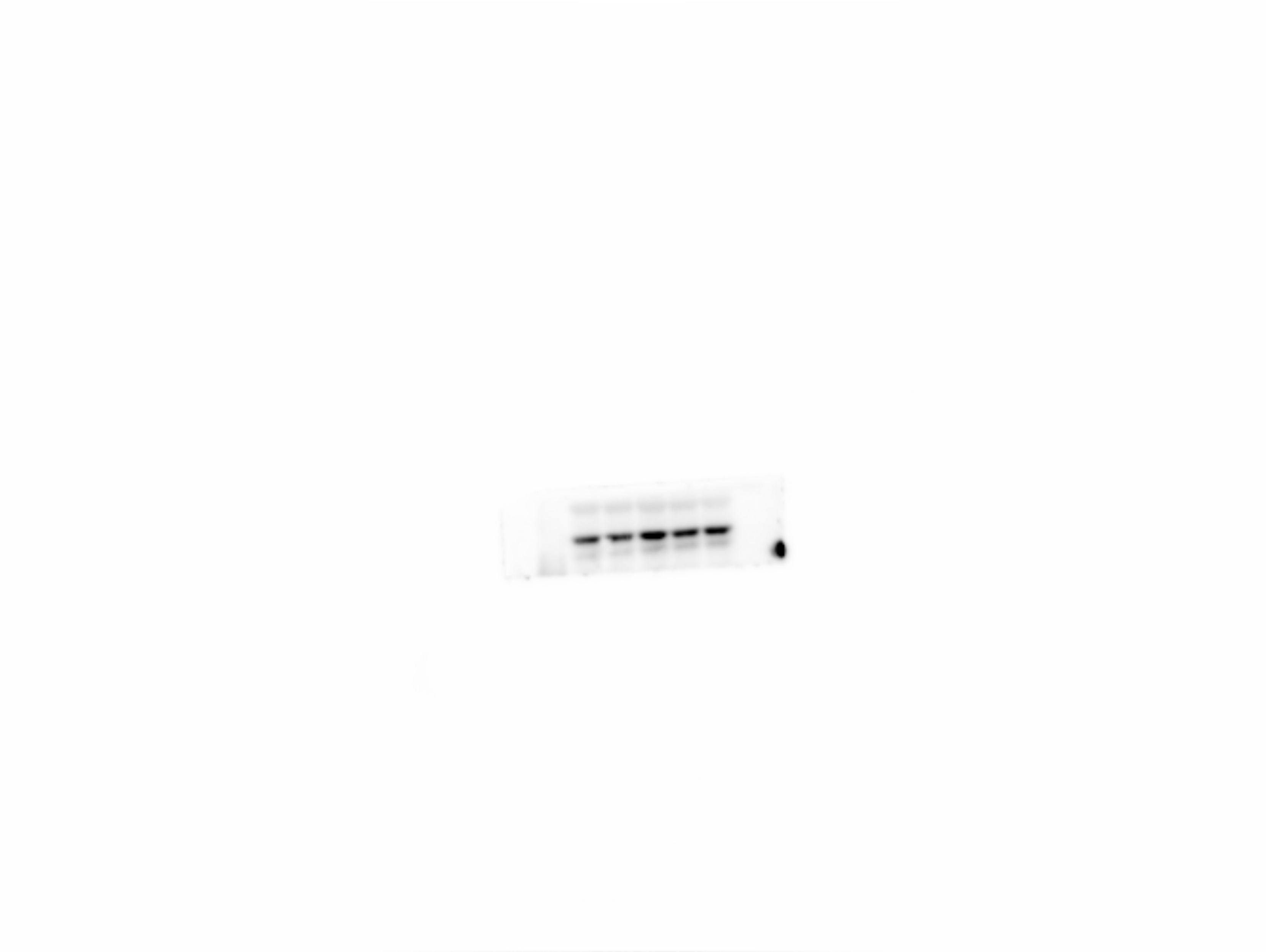

Supplement: S2 File — Original picture of the western blot experiments in the manuscript. (ZIP) [file pone.0274620.s002.zip › S2. blot results/Fig 5/p-Src/1.tif]

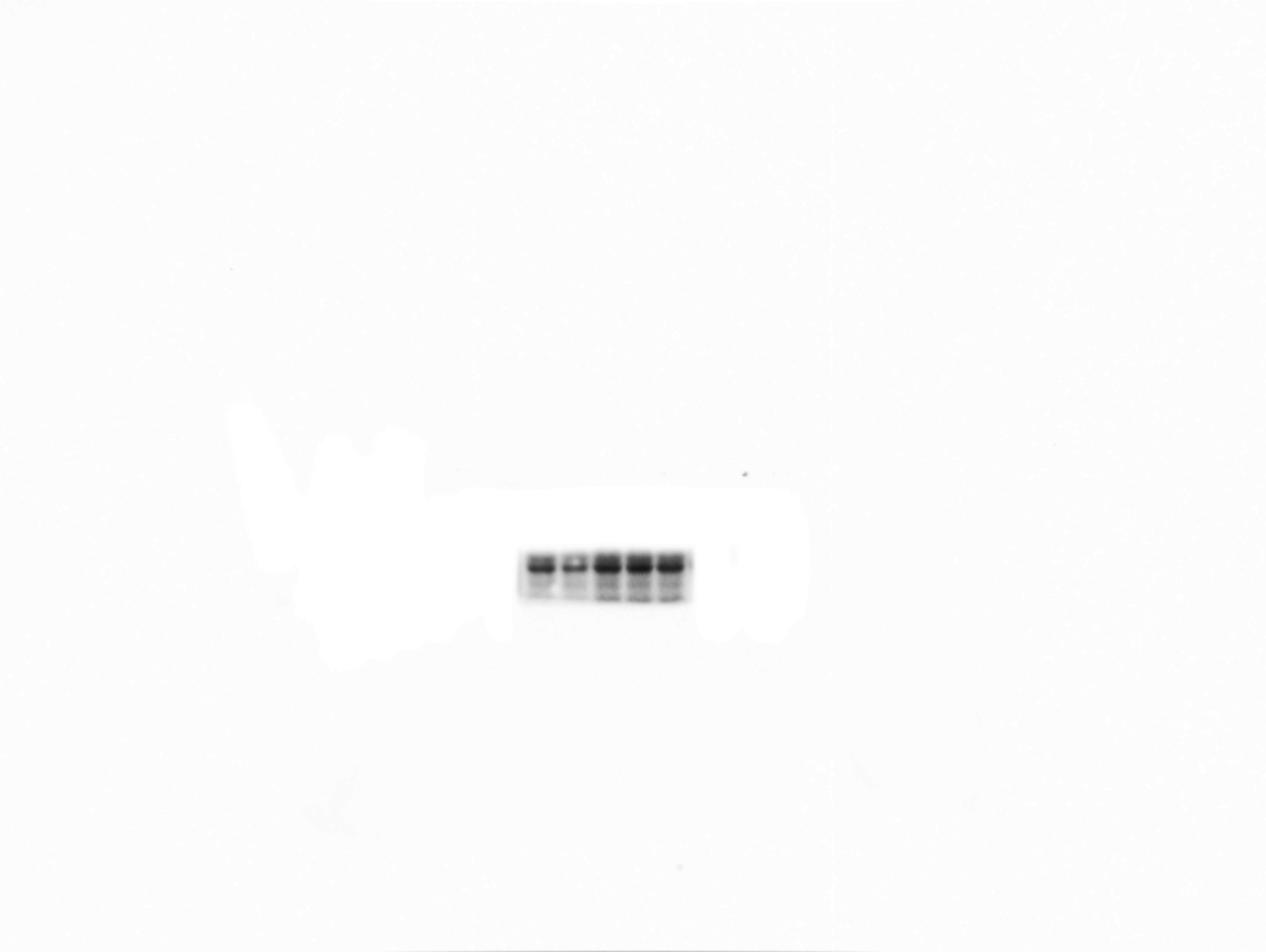

Supplement: S2 File — Original picture of the western blot experiments in the manuscript. (ZIP) [file pone.0274620.s002.zip › S2. blot results/Fig 5/p-Src/2.tif]

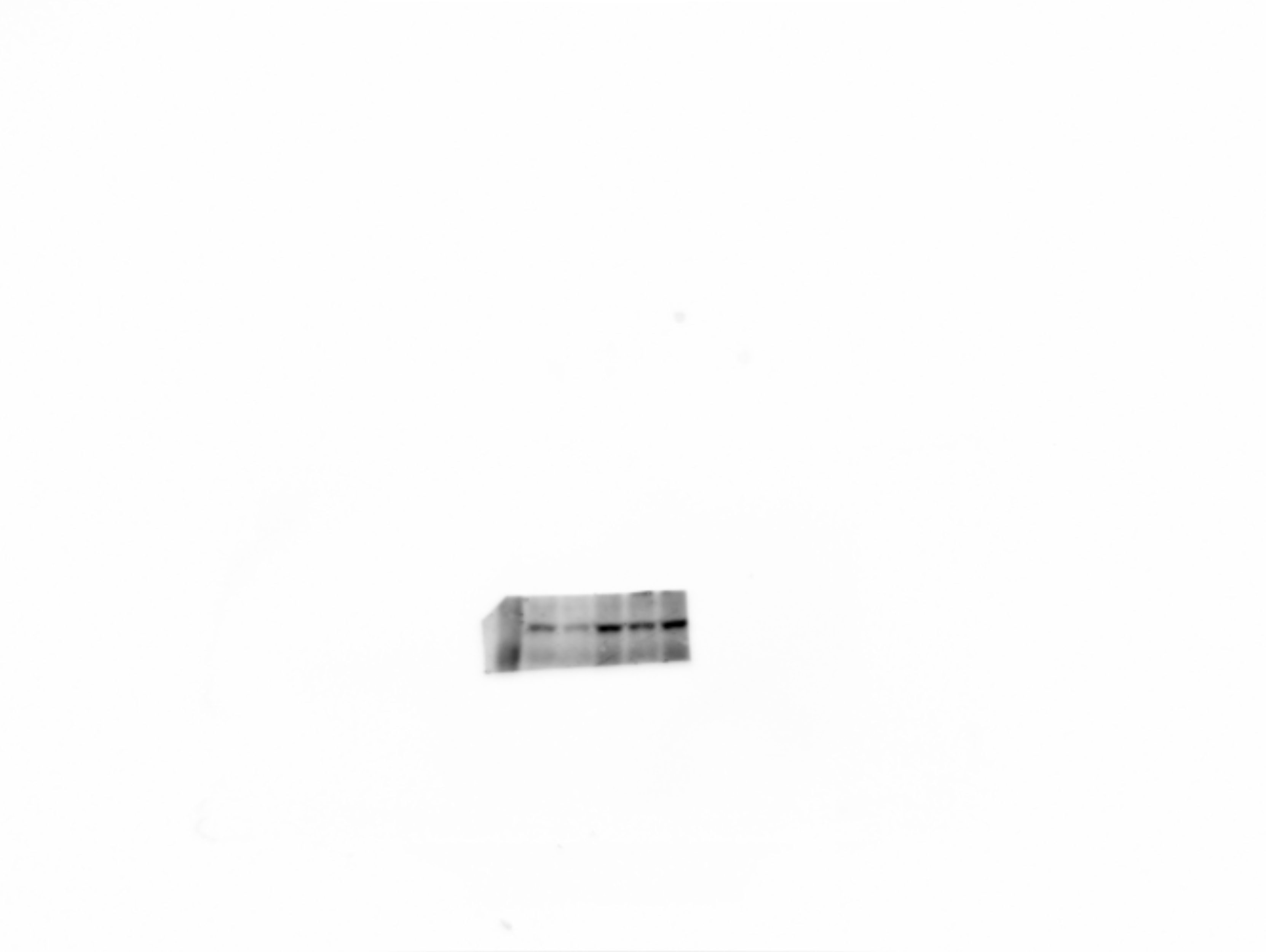

Supplement: S2 File — Original picture of the western blot experiments in the manuscript. (ZIP) [file pone.0274620.s002.zip › S2. blot results/Fig 5/p-Src/3.tif]

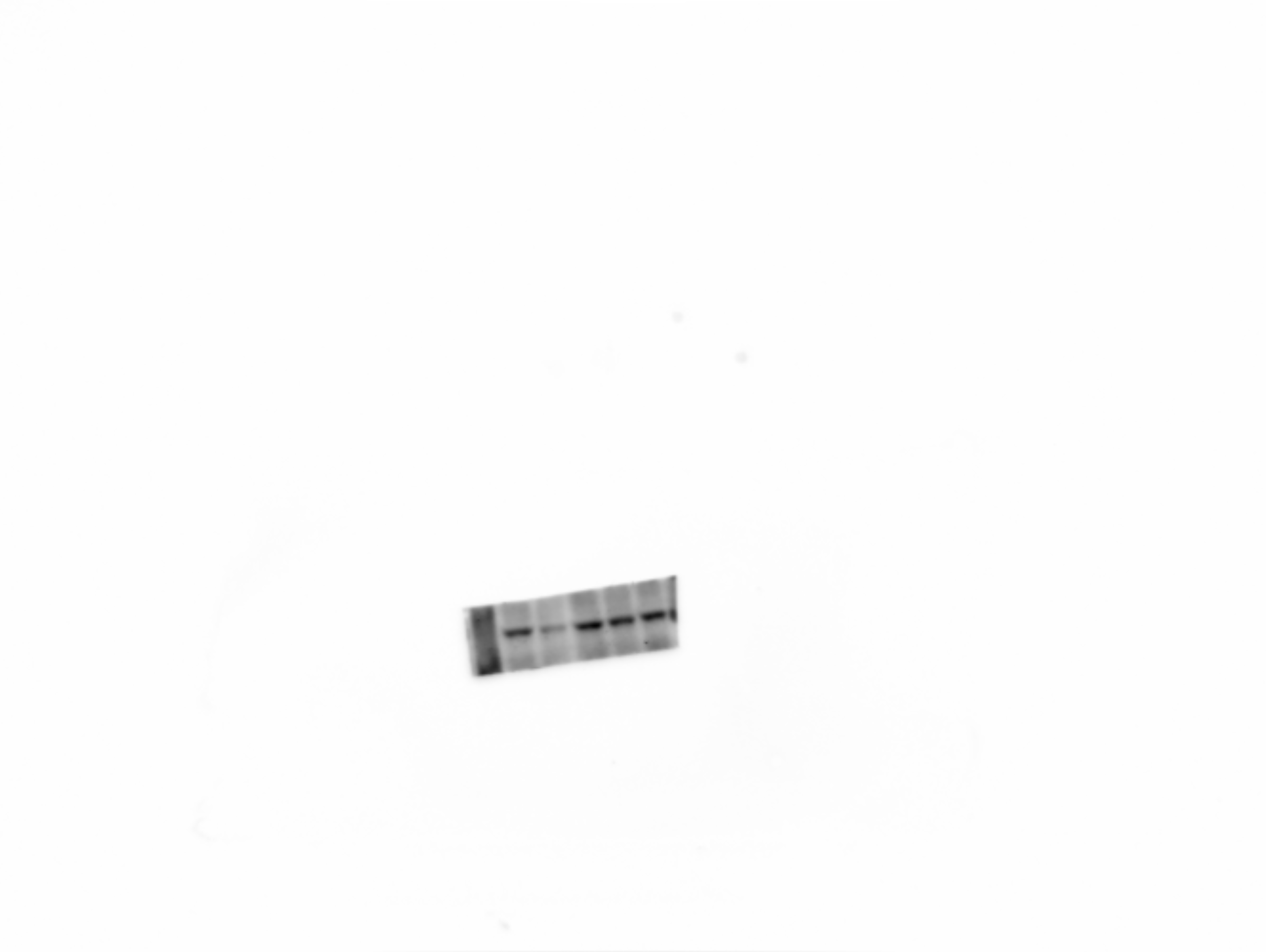

Supplement: S2 File — Original picture of the western blot experiments in the manuscript. (ZIP) [file pone.0274620.s002.zip › S2. blot results/Fig 5/p-Src/4.tif]

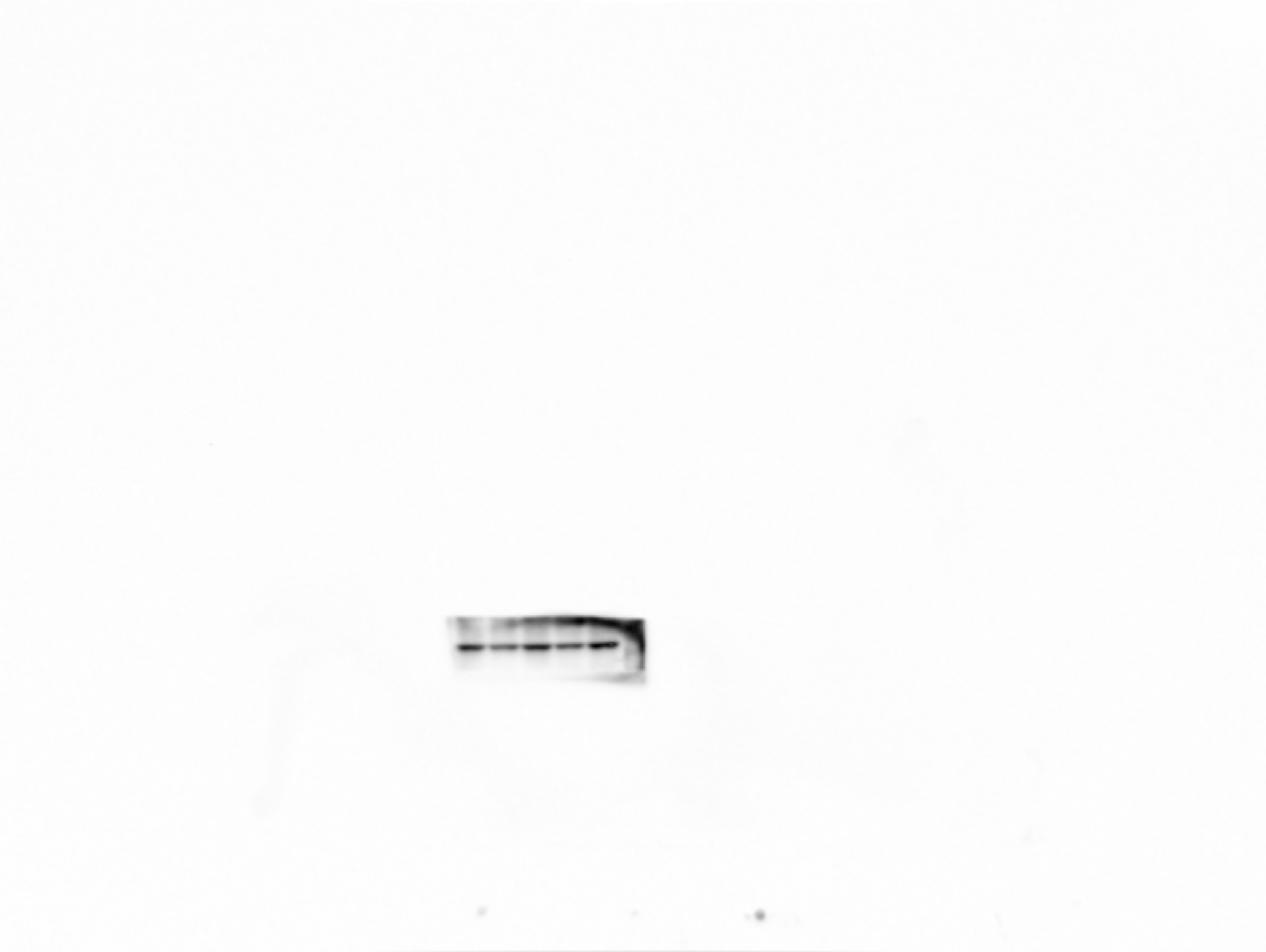

Supplement: S2 File — Original picture of the western blot experiments in the manuscript. (ZIP) [file pone.0274620.s002.zip › S2. blot results/Fig 5/p-Src/5.tif]

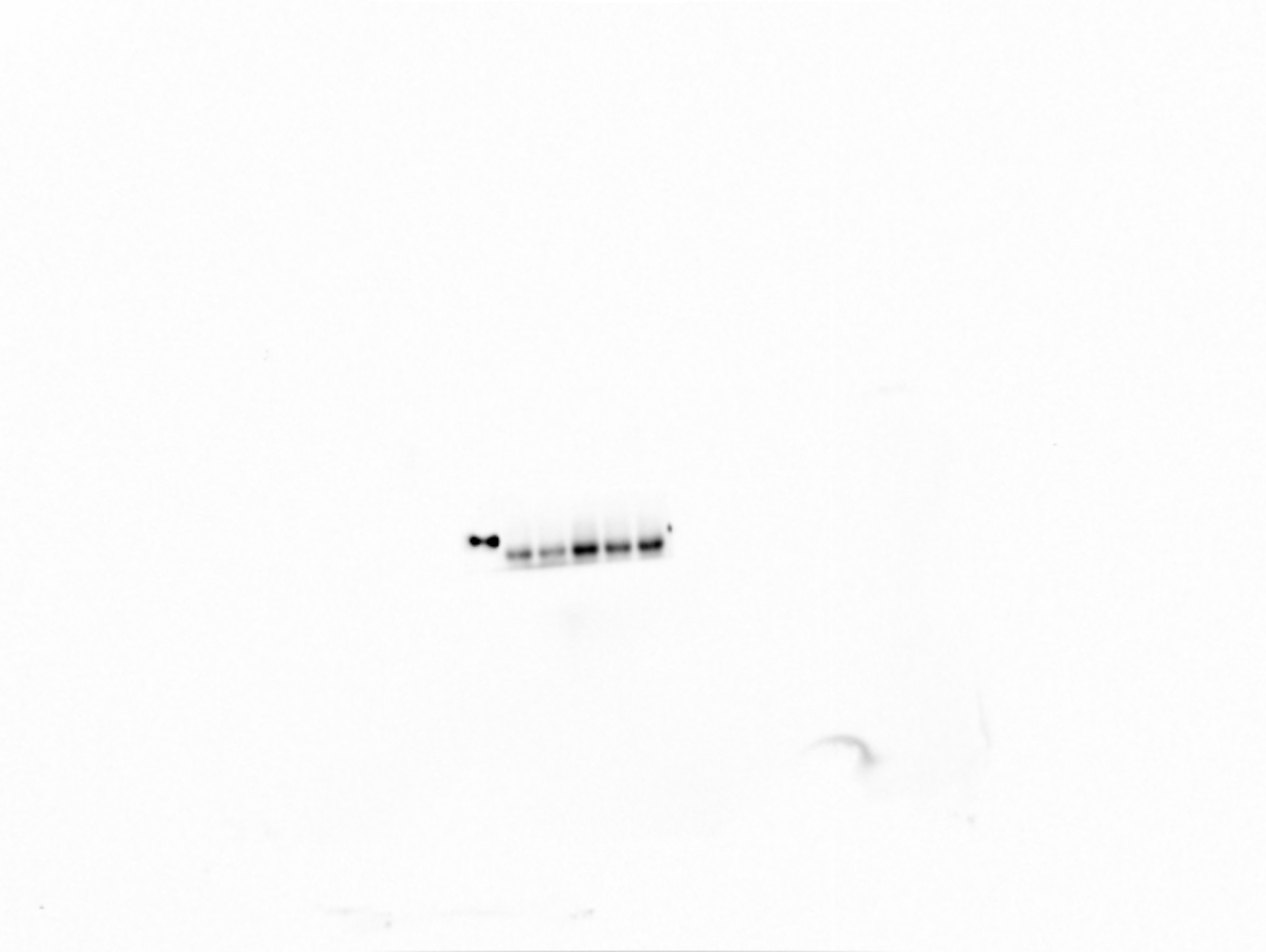

Supplement: S2 File — Original picture of the western blot experiments in the manuscript. (ZIP) [file pone.0274620.s002.zip › S2. blot results/Fig 5/VEGF/1.tif]

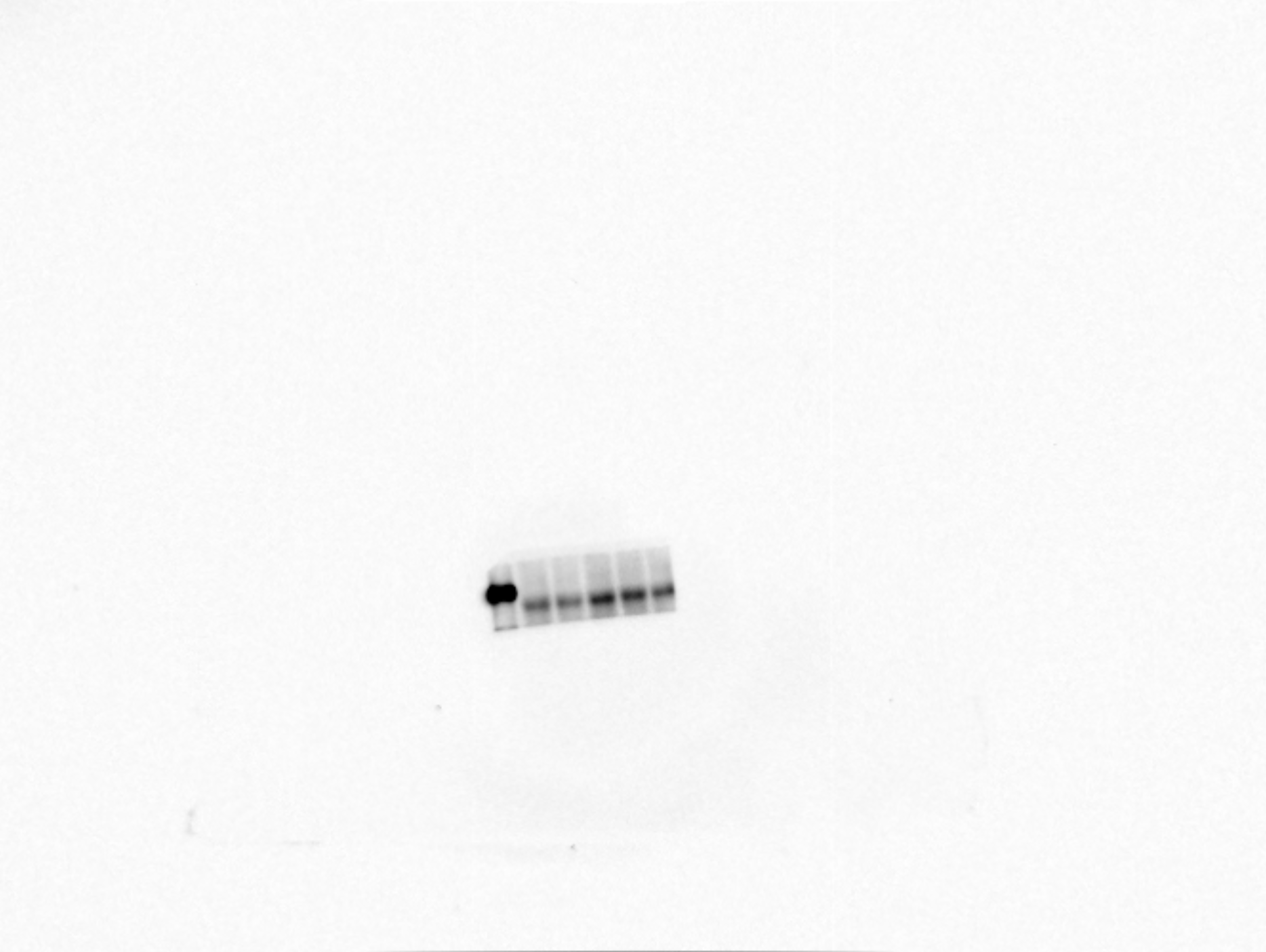

Supplement: S2 File — Original picture of the western blot experiments in the manuscript. (ZIP) [file pone.0274620.s002.zip › S2. blot results/Fig 5/VEGF/2.tif]

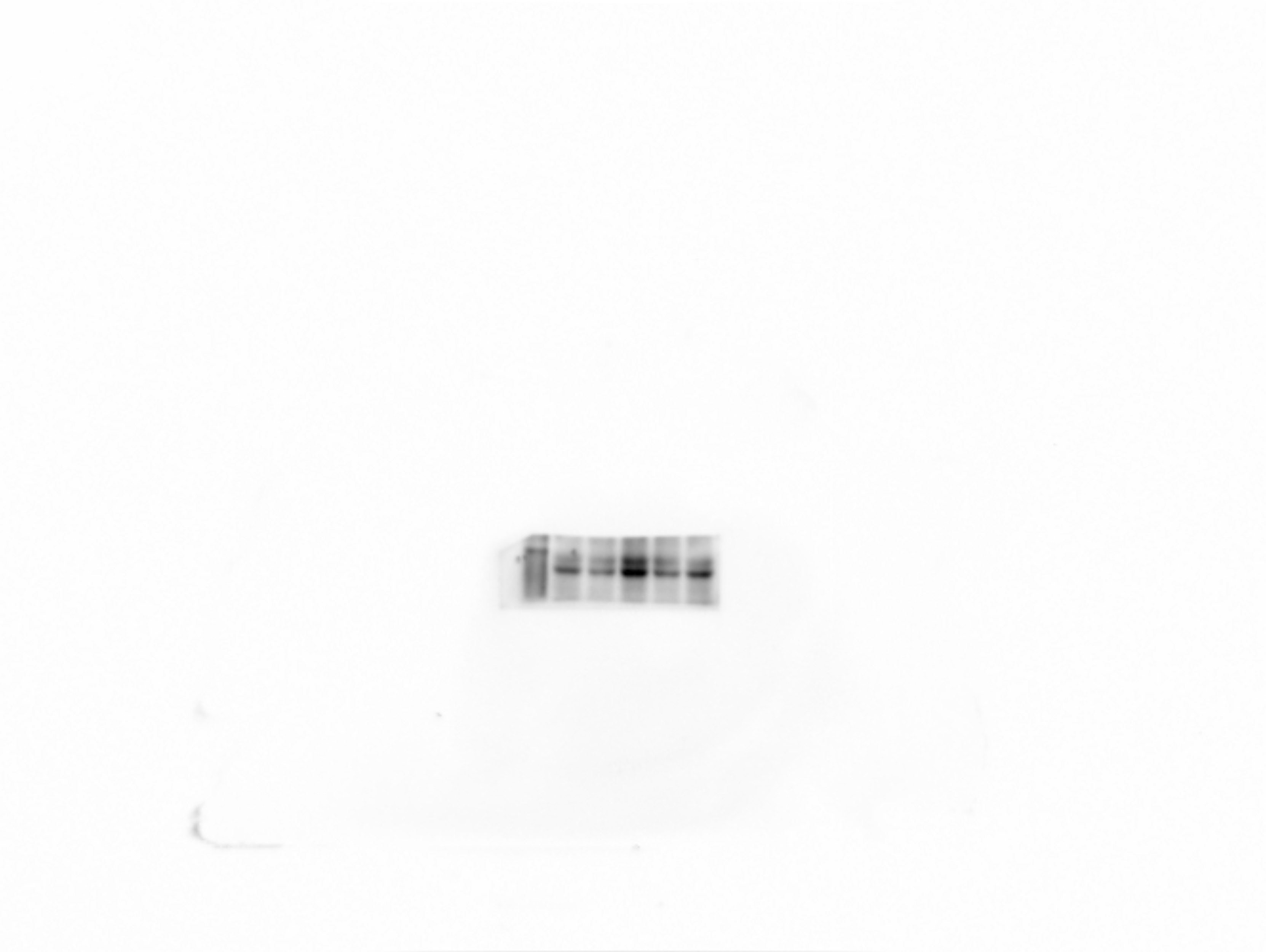

Supplement: S2 File — Original picture of the western blot experiments in the manuscript. (ZIP) [file pone.0274620.s002.zip › S2. blot results/Fig 5/VEGF/3.tif]

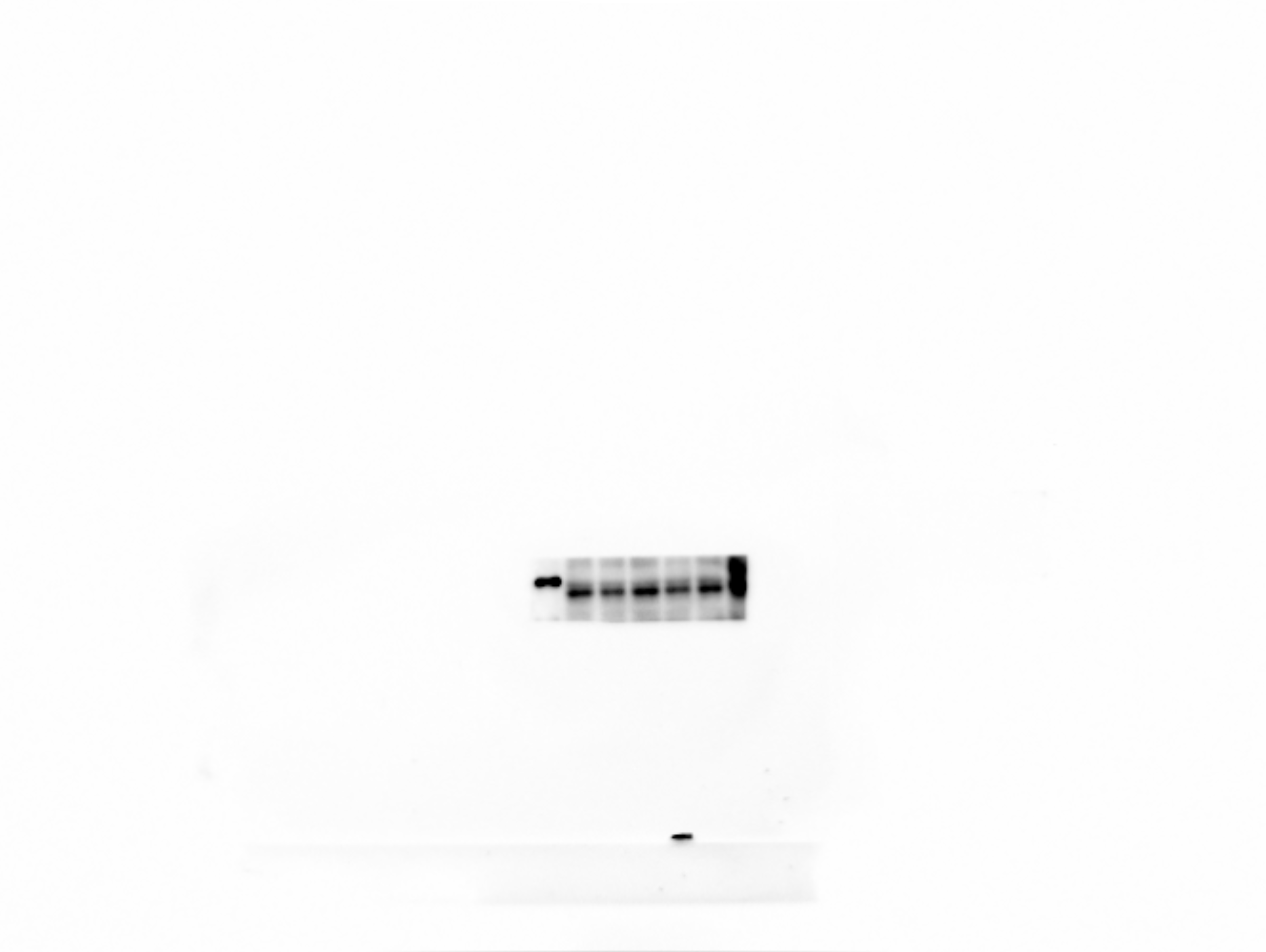

Supplement: S2 File — Original picture of the western blot experiments in the manuscript. (ZIP) [file pone.0274620.s002.zip › S2. blot results/Fig 5/VEGF/4.tif]

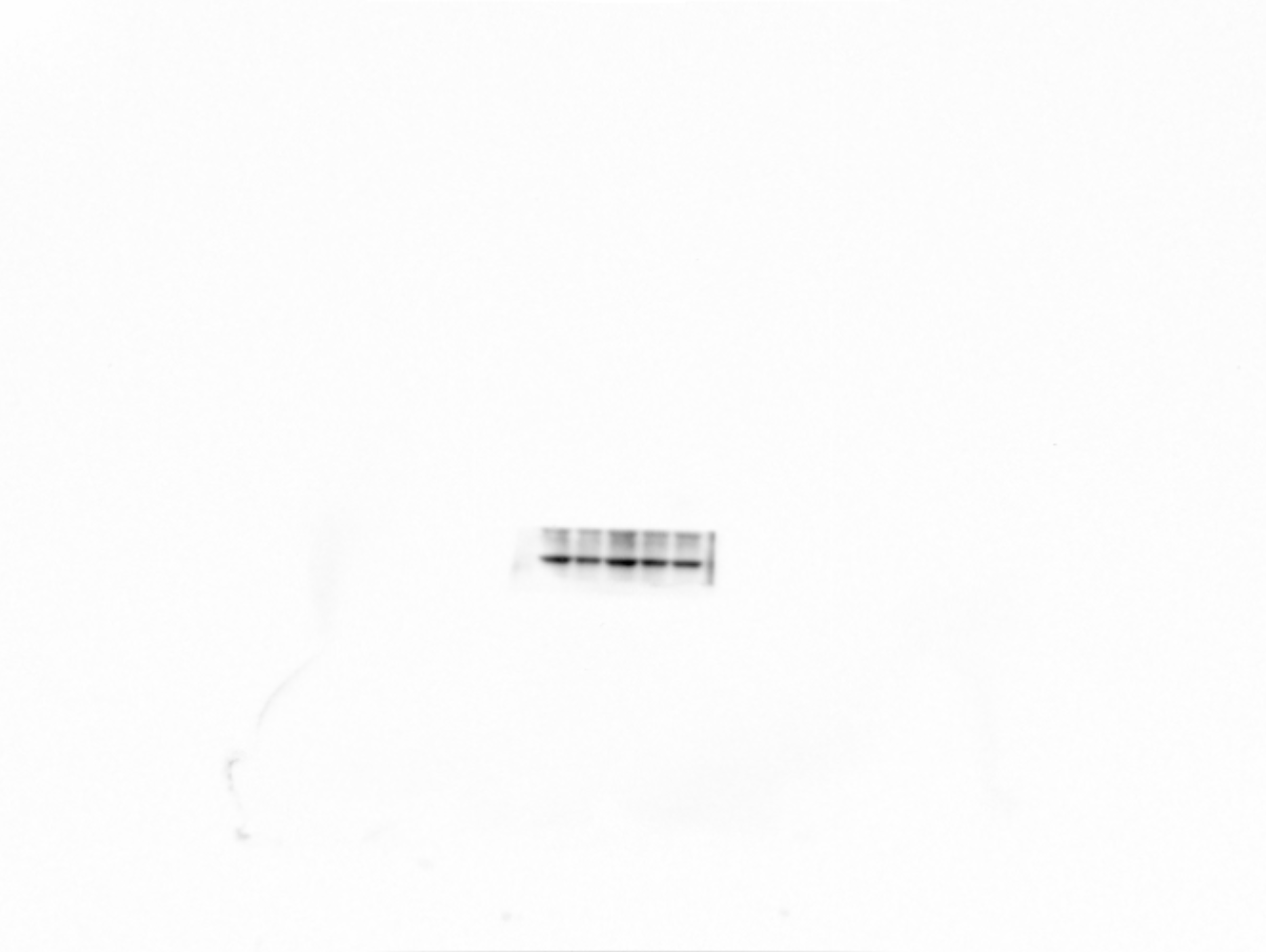

Supplement: S2 File — Original picture of the western blot experiments in the manuscript. (ZIP) [file pone.0274620.s002.zip › S2. blot results/Fig 5/VEGF/5.tif]
