# Supplementary figures and images for: Electroacupuncture attenuates ischemic injury after stroke and promotes angiogenesis via activation of EPO mediated Src and VEGF signaling pathways (part 1 of 2)
Source: PLoS One. 2022 Sep 15;17(9):e0274620. doi: 10.1371/journal.pone.0274620 (PMC9477374; doi:10.1371/journal.pone.0274620)

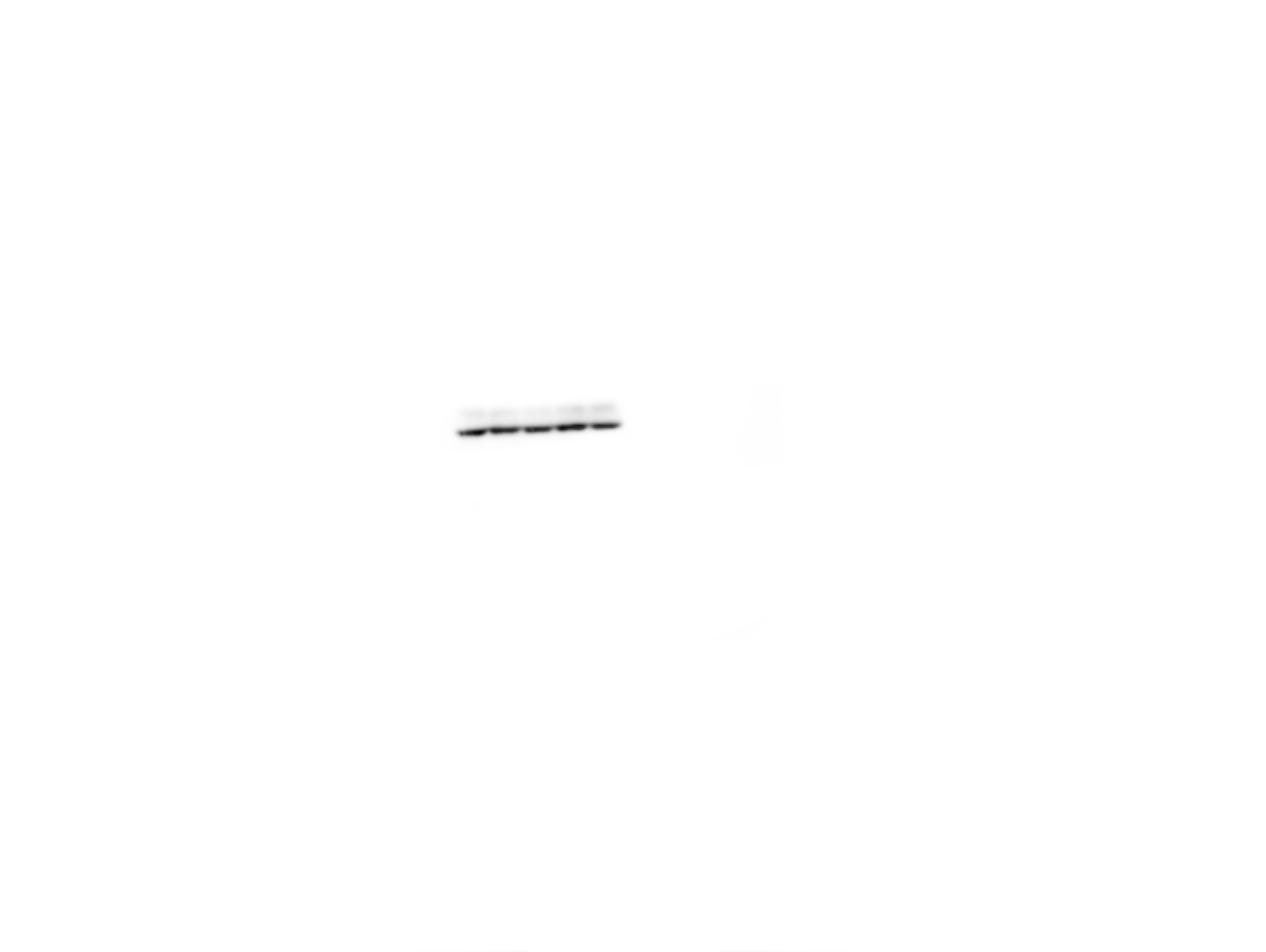

Supplement: S2 File — Original picture of the western blot experiments in the manuscript. (ZIP) [file pone.0274620.s002.zip › S2. blot results/Fig 3/CD34/1control/1.tif]

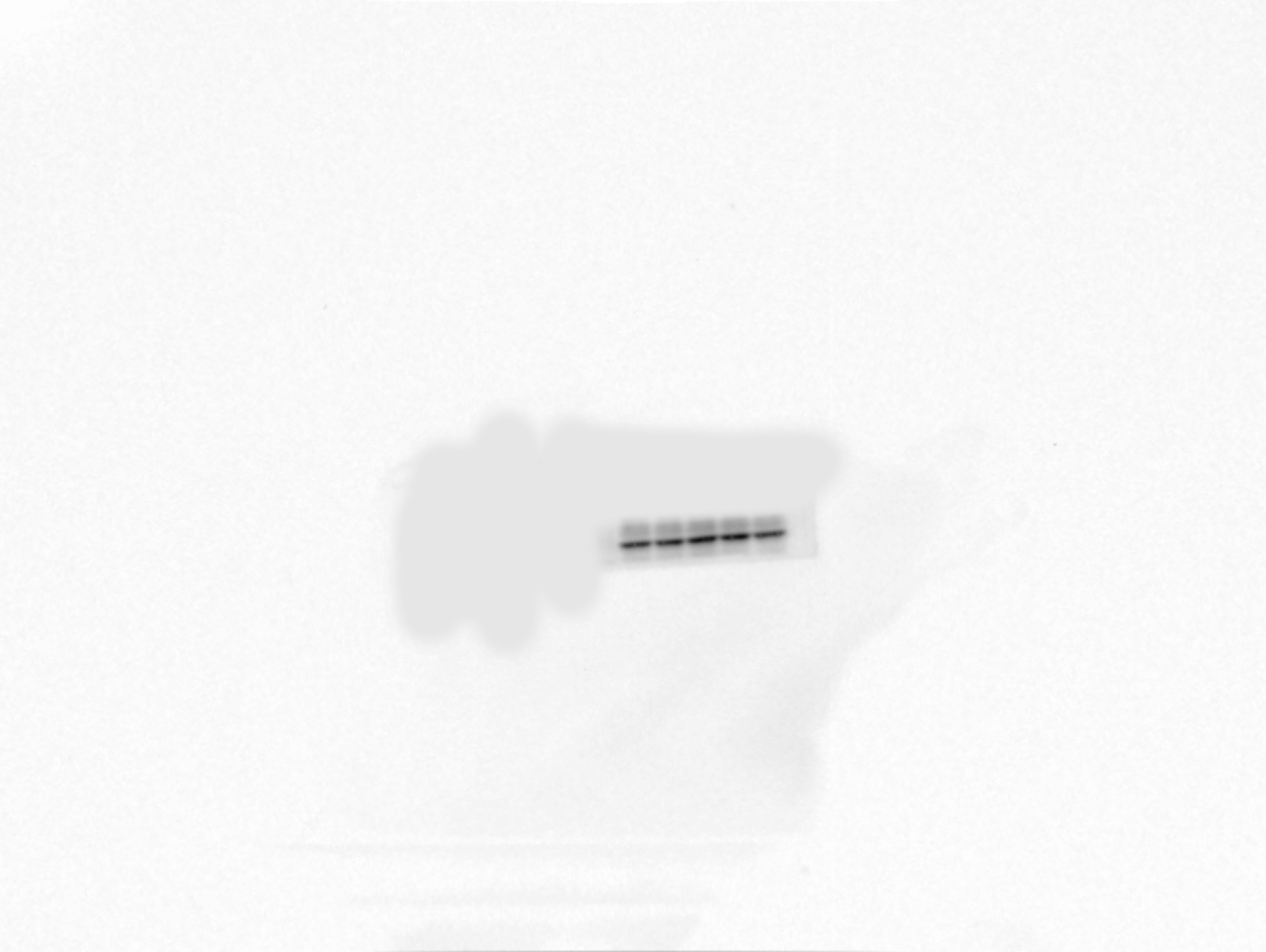

Supplement: S2 File — Original picture of the western blot experiments in the manuscript. (ZIP) [file pone.0274620.s002.zip › S2. blot results/Fig 3/CD34/1control/2.tif]

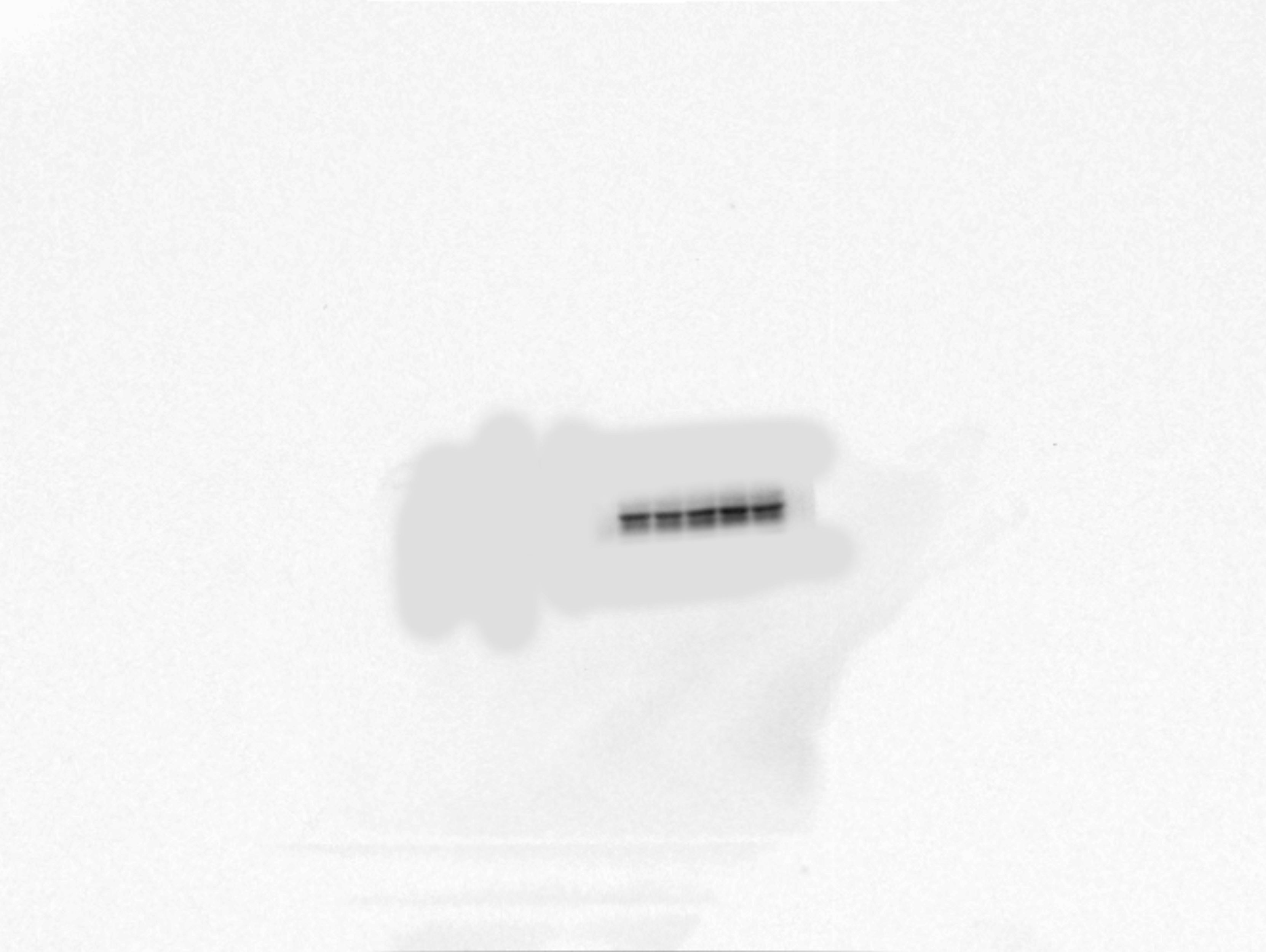

Supplement: S2 File — Original picture of the western blot experiments in the manuscript. (ZIP) [file pone.0274620.s002.zip › S2. blot results/Fig 3/CD34/1control/3.tif]

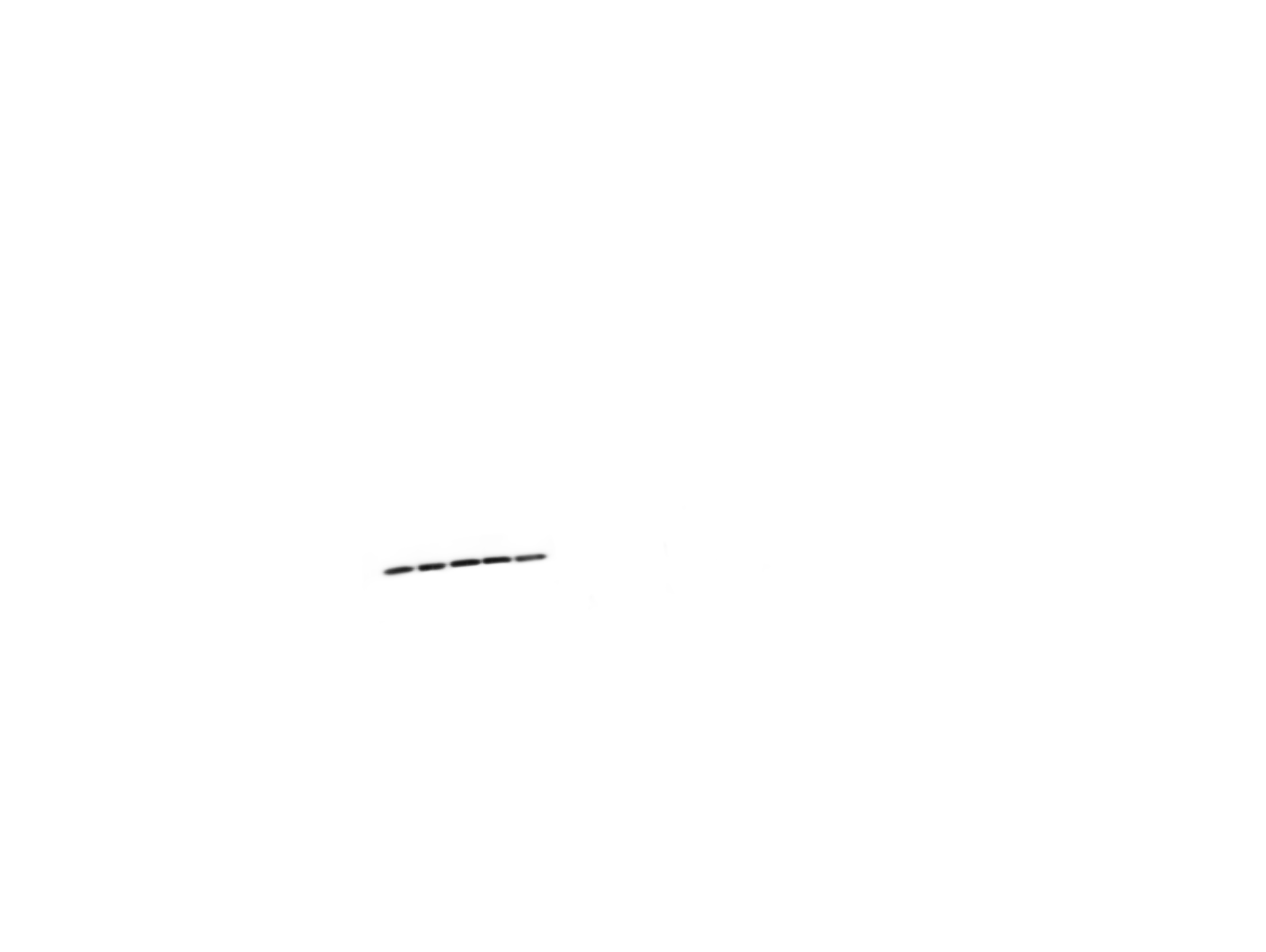

Supplement: S2 File — Original picture of the western blot experiments in the manuscript. (ZIP) [file pone.0274620.s002.zip › S2. blot results/Fig 3/CD34/1control/4.tif]

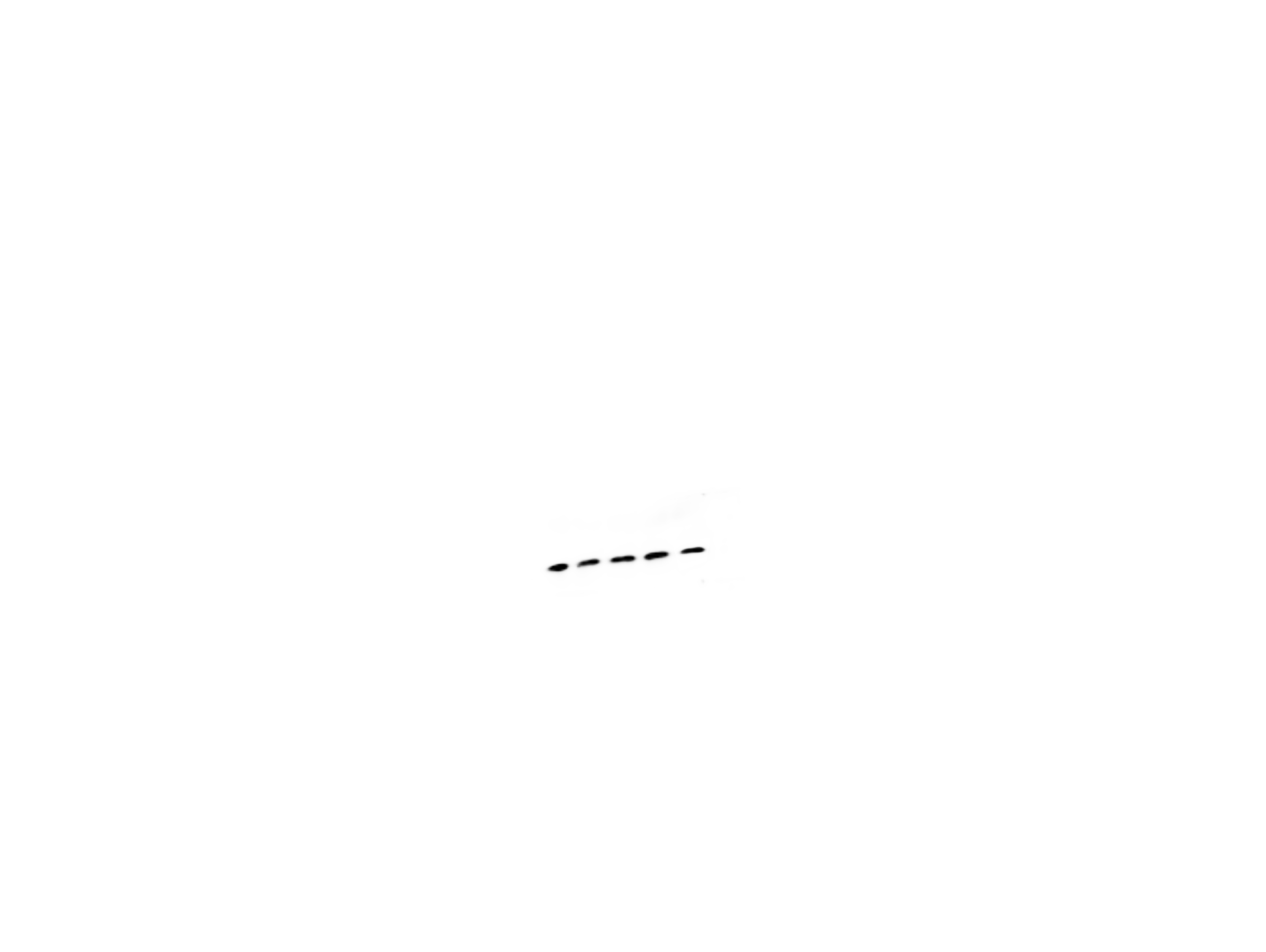

Supplement: S2 File — Original picture of the western blot experiments in the manuscript. (ZIP) [file pone.0274620.s002.zip › S2. blot results/Fig 3/CD34/1control/5.tif]

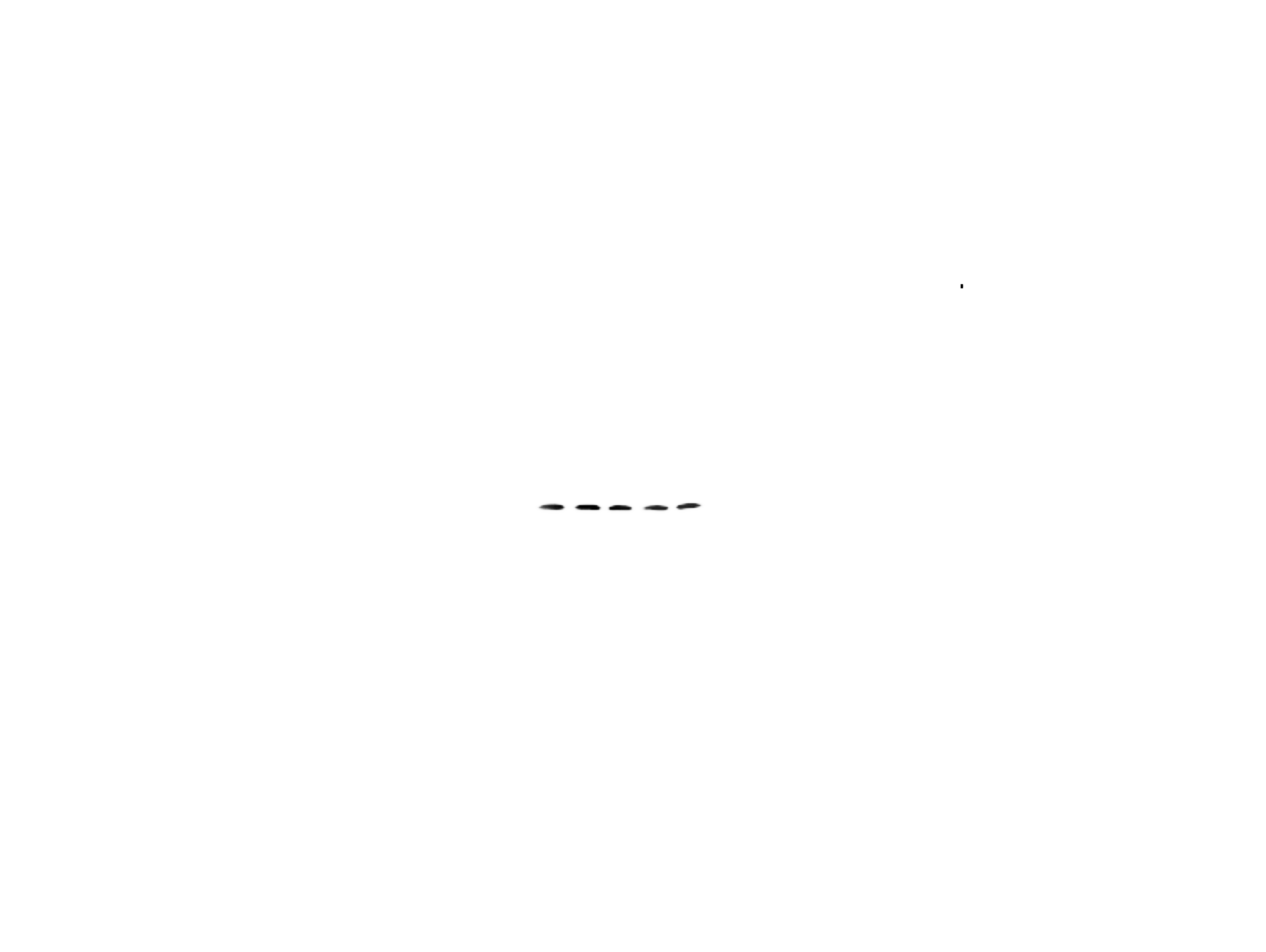

Supplement: S2 File — Original picture of the western blot experiments in the manuscript. (ZIP) [file pone.0274620.s002.zip › S2. blot results/Fig 3/CD34/2sham/1.tif]

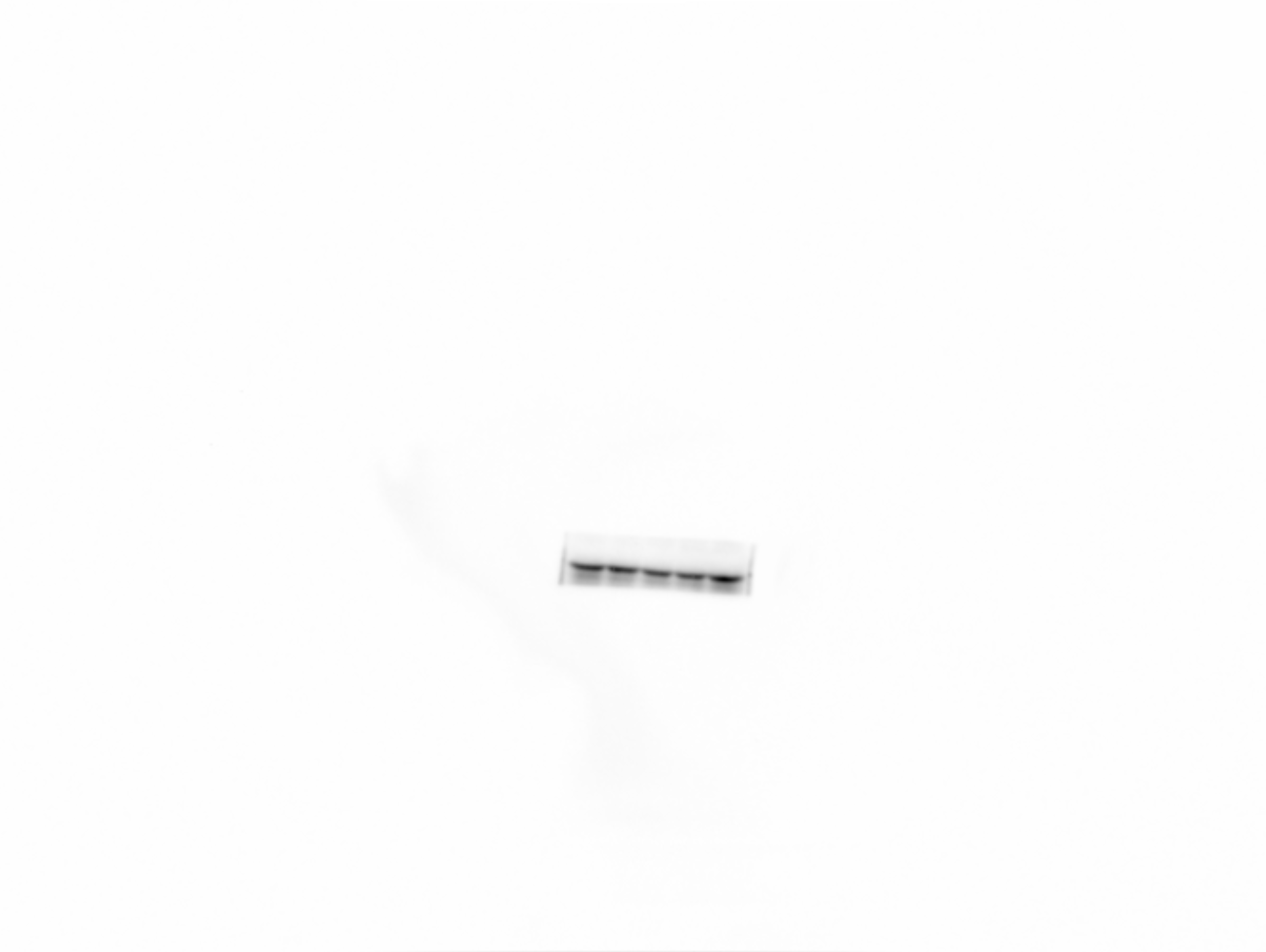

Supplement: S2 File — Original picture of the western blot experiments in the manuscript. (ZIP) [file pone.0274620.s002.zip › S2. blot results/Fig 3/CD34/2sham/2.tif]

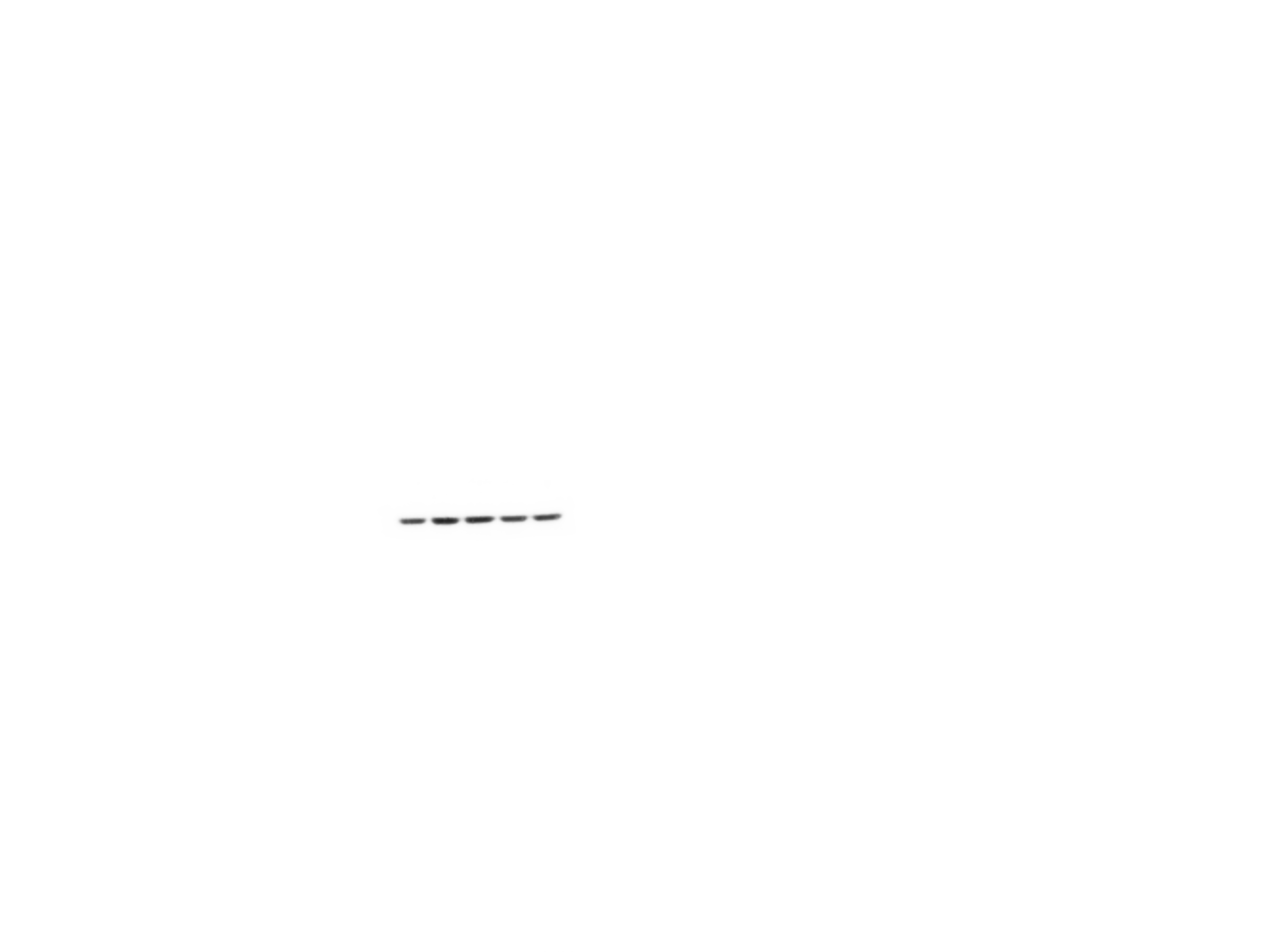

Supplement: S2 File — Original picture of the western blot experiments in the manuscript. (ZIP) [file pone.0274620.s002.zip › S2. blot results/Fig 3/CD34/2sham/3.tif]

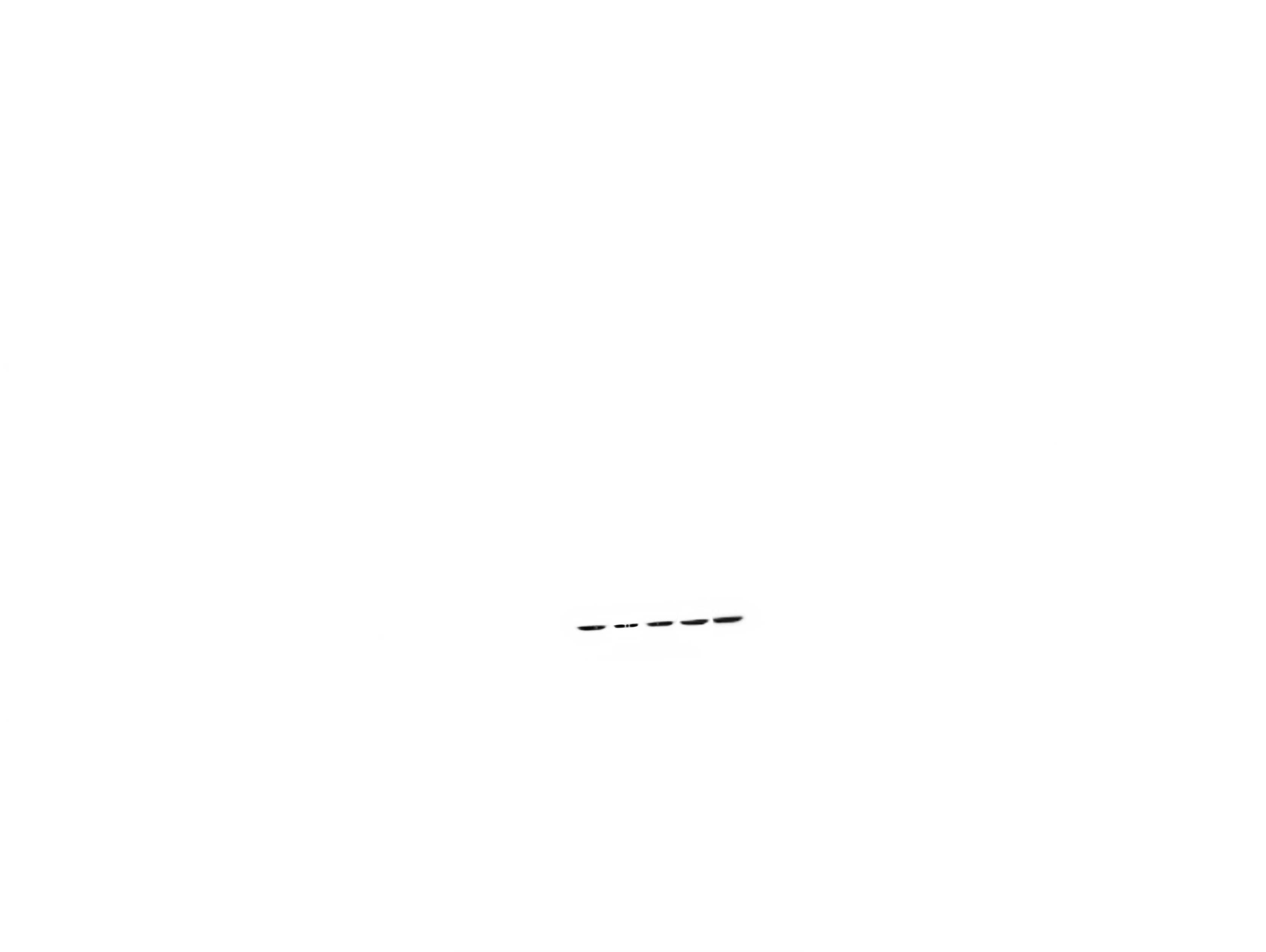

Supplement: S2 File — Original picture of the western blot experiments in the manuscript. (ZIP) [file pone.0274620.s002.zip › S2. blot results/Fig 3/CD34/2sham/4.tif]

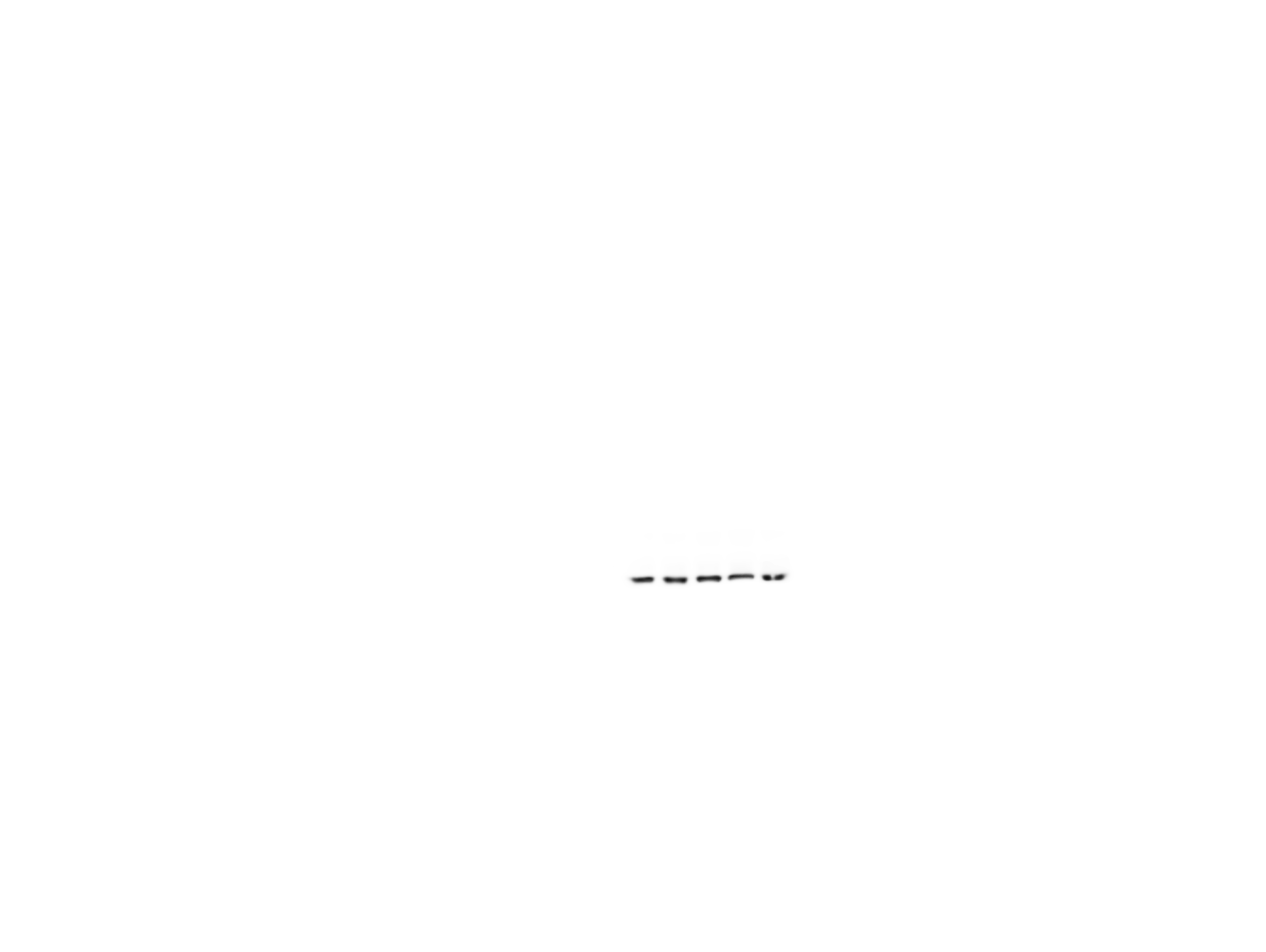

Supplement: S2 File — Original picture of the western blot experiments in the manuscript. (ZIP) [file pone.0274620.s002.zip › S2. blot results/Fig 3/CD34/2sham/5.tif]

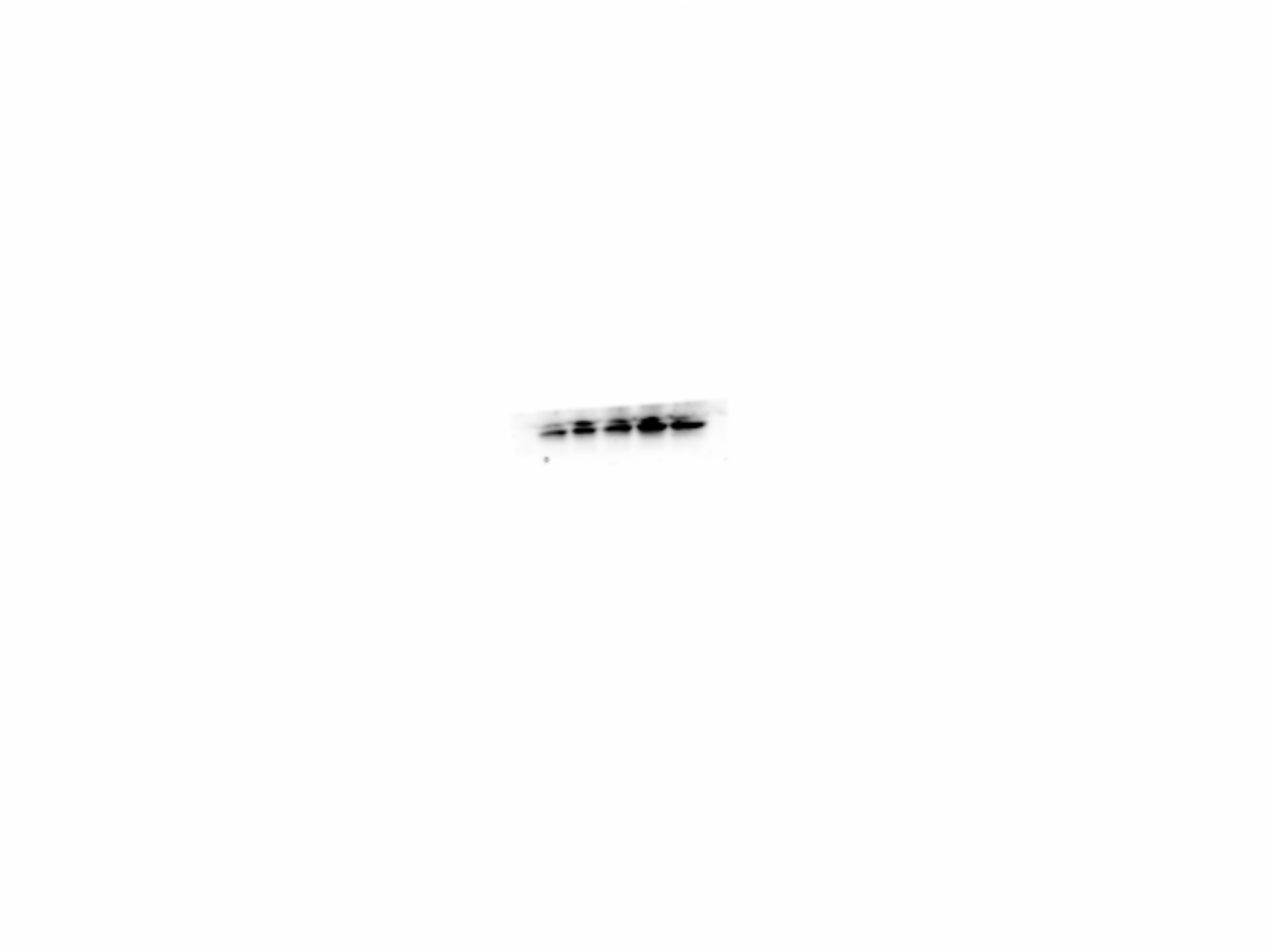

Supplement: S2 File — Original picture of the western blot experiments in the manuscript. (ZIP) [file pone.0274620.s002.zip › S2. blot results/Fig 3/CD34/3model/1.tif]

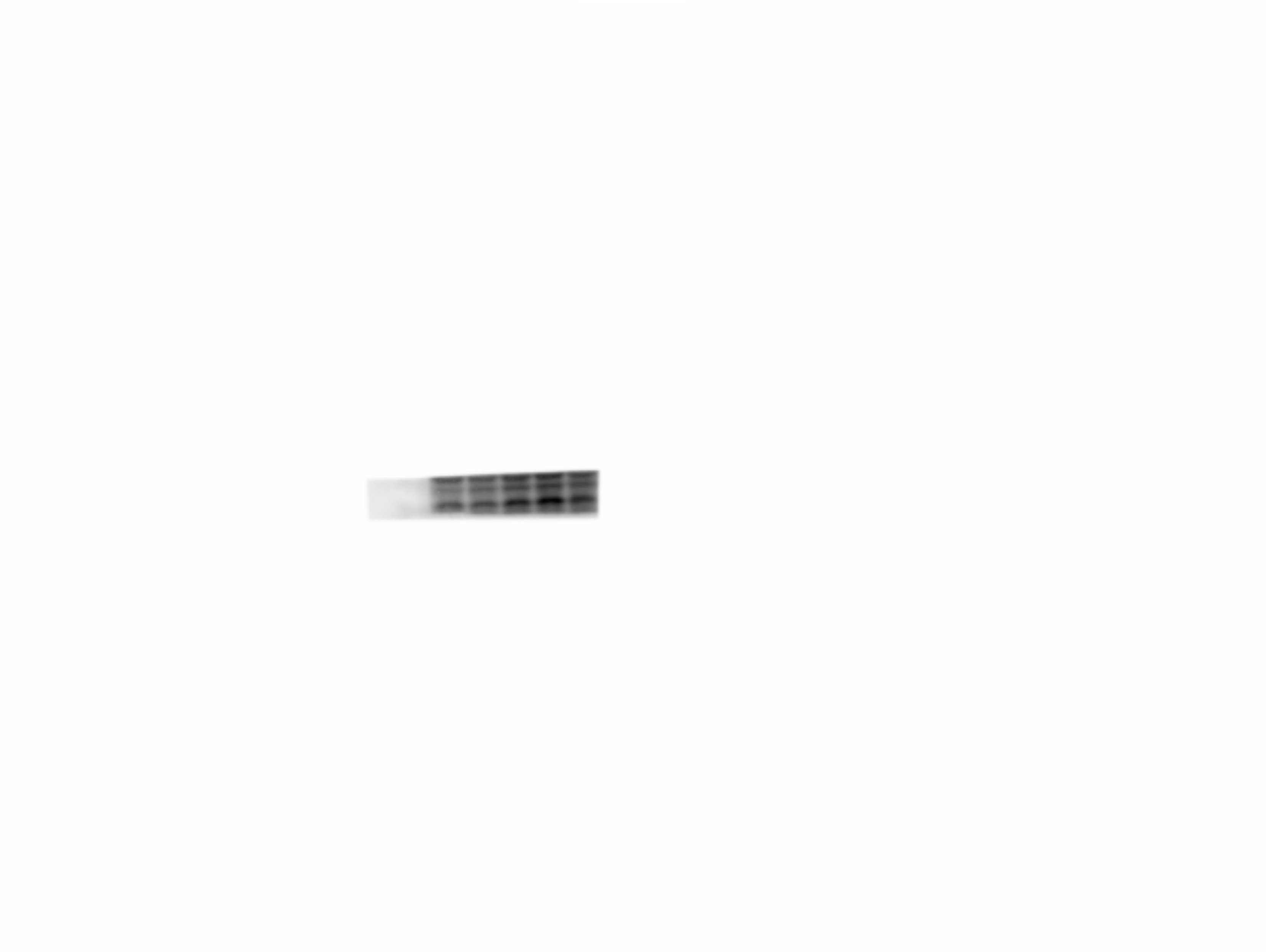

Supplement: S2 File — Original picture of the western blot experiments in the manuscript. (ZIP) [file pone.0274620.s002.zip › S2. blot results/Fig 3/CD34/3model/2.tif]

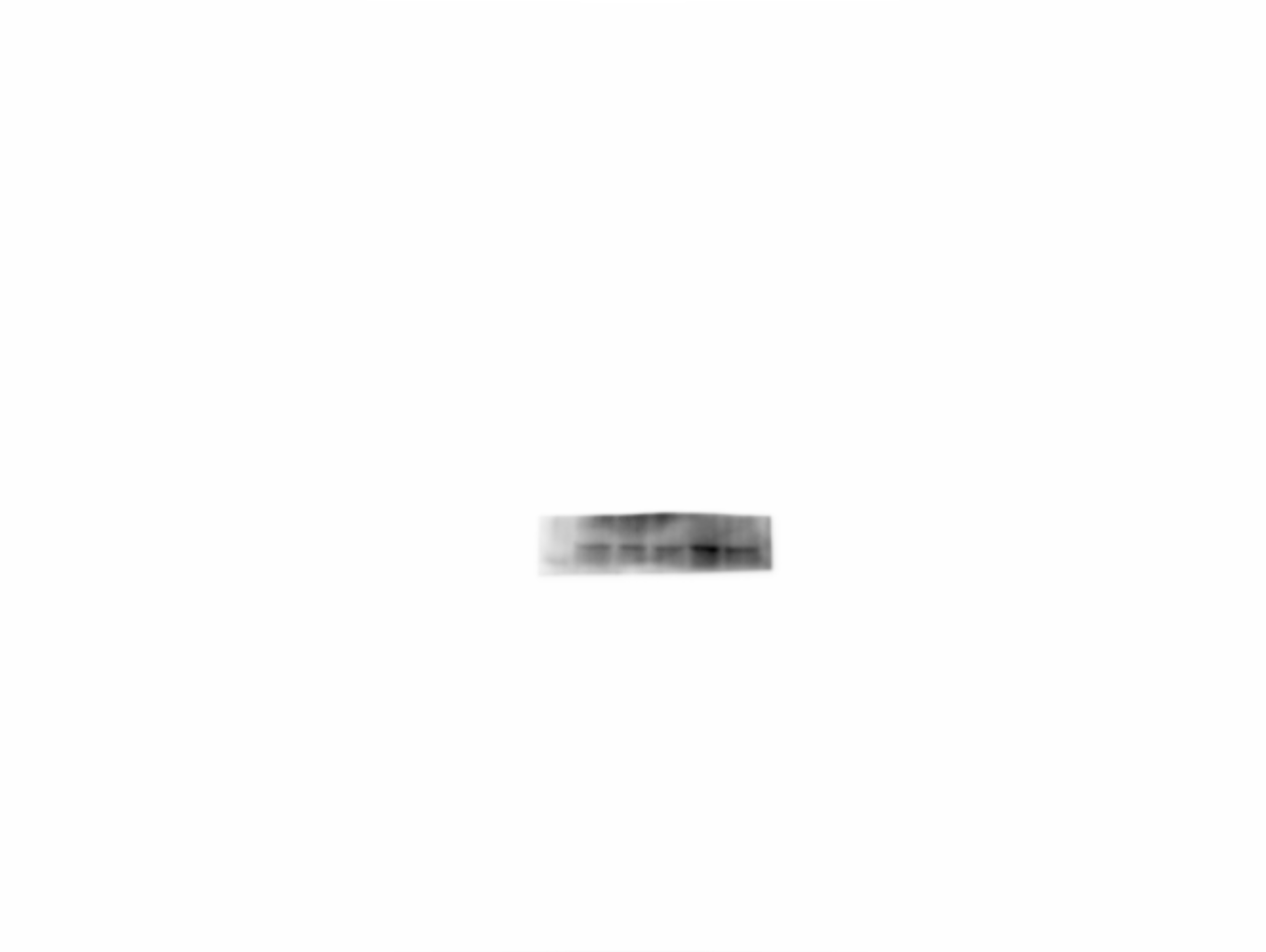

Supplement: S2 File — Original picture of the western blot experiments in the manuscript. (ZIP) [file pone.0274620.s002.zip › S2. blot results/Fig 3/CD34/3model/3.tif]

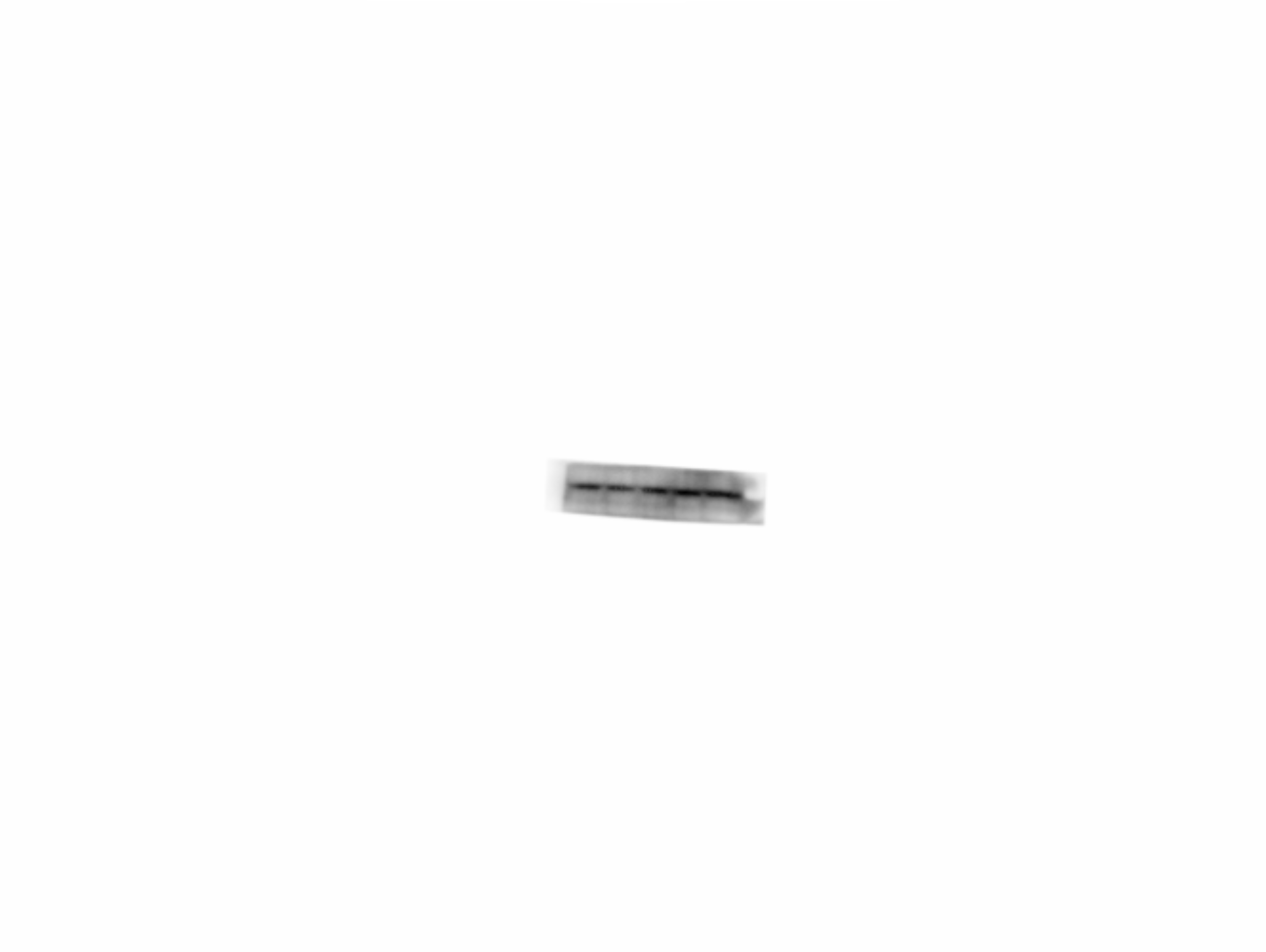

Supplement: S2 File — Original picture of the western blot experiments in the manuscript. (ZIP) [file pone.0274620.s002.zip › S2. blot results/Fig 3/CD34/3model/4.tif]

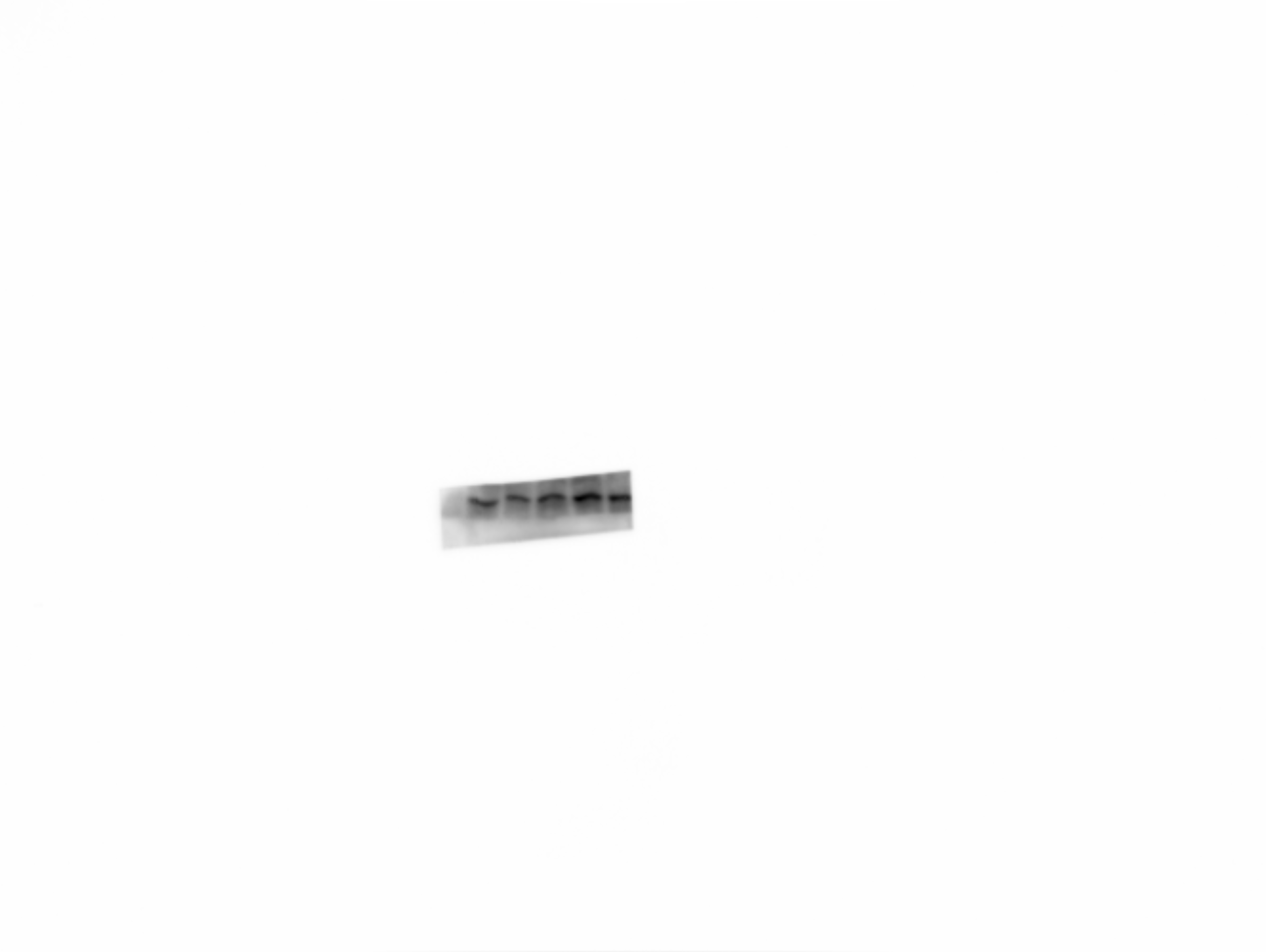

Supplement: S2 File — Original picture of the western blot experiments in the manuscript. (ZIP) [file pone.0274620.s002.zip › S2. blot results/Fig 3/CD34/3model/5.tif]

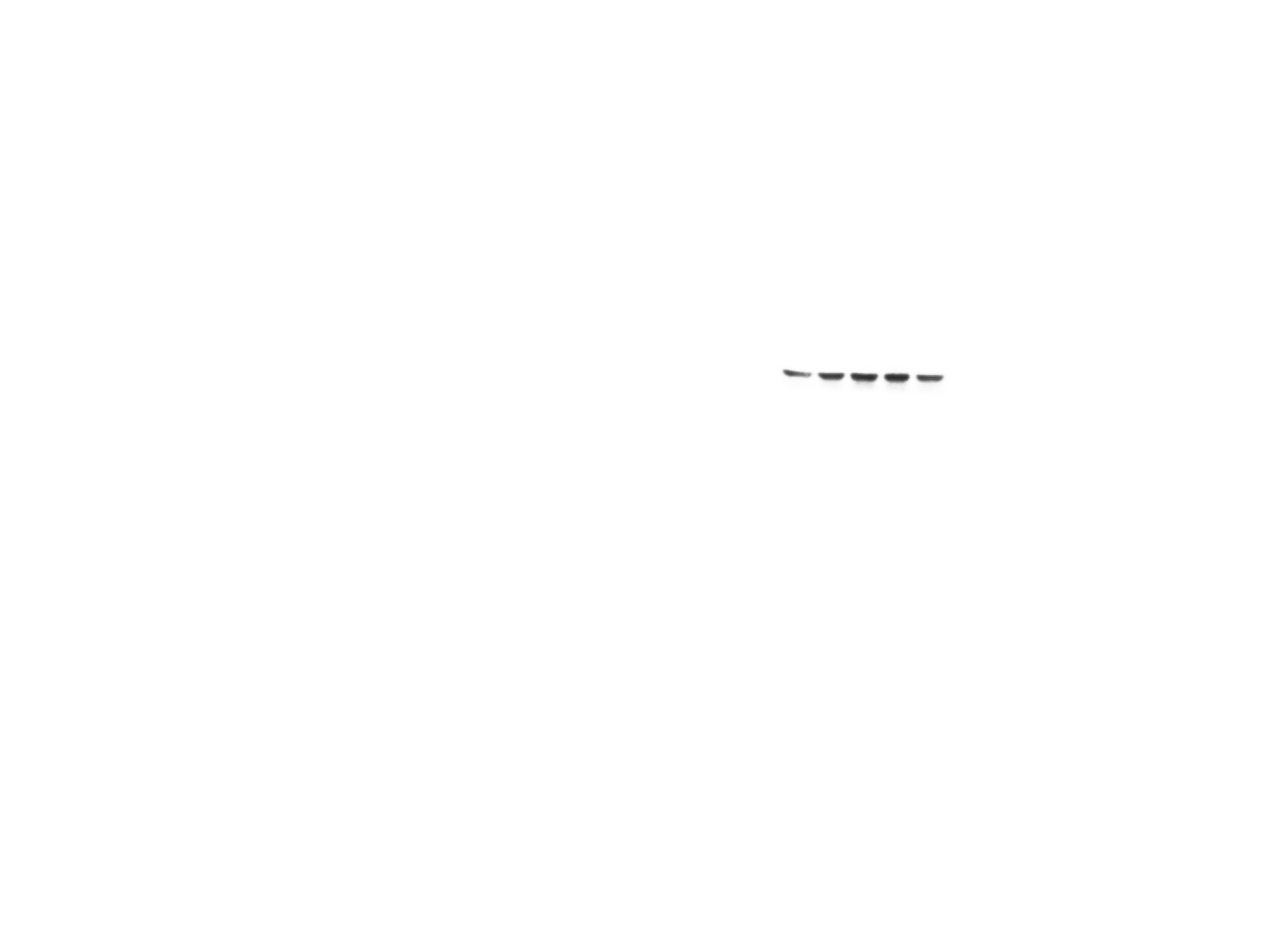

Supplement: S2 File — Original picture of the western blot experiments in the manuscript. (ZIP) [file pone.0274620.s002.zip › S2. blot results/Fig 3/CD34/4EA/1.tif]

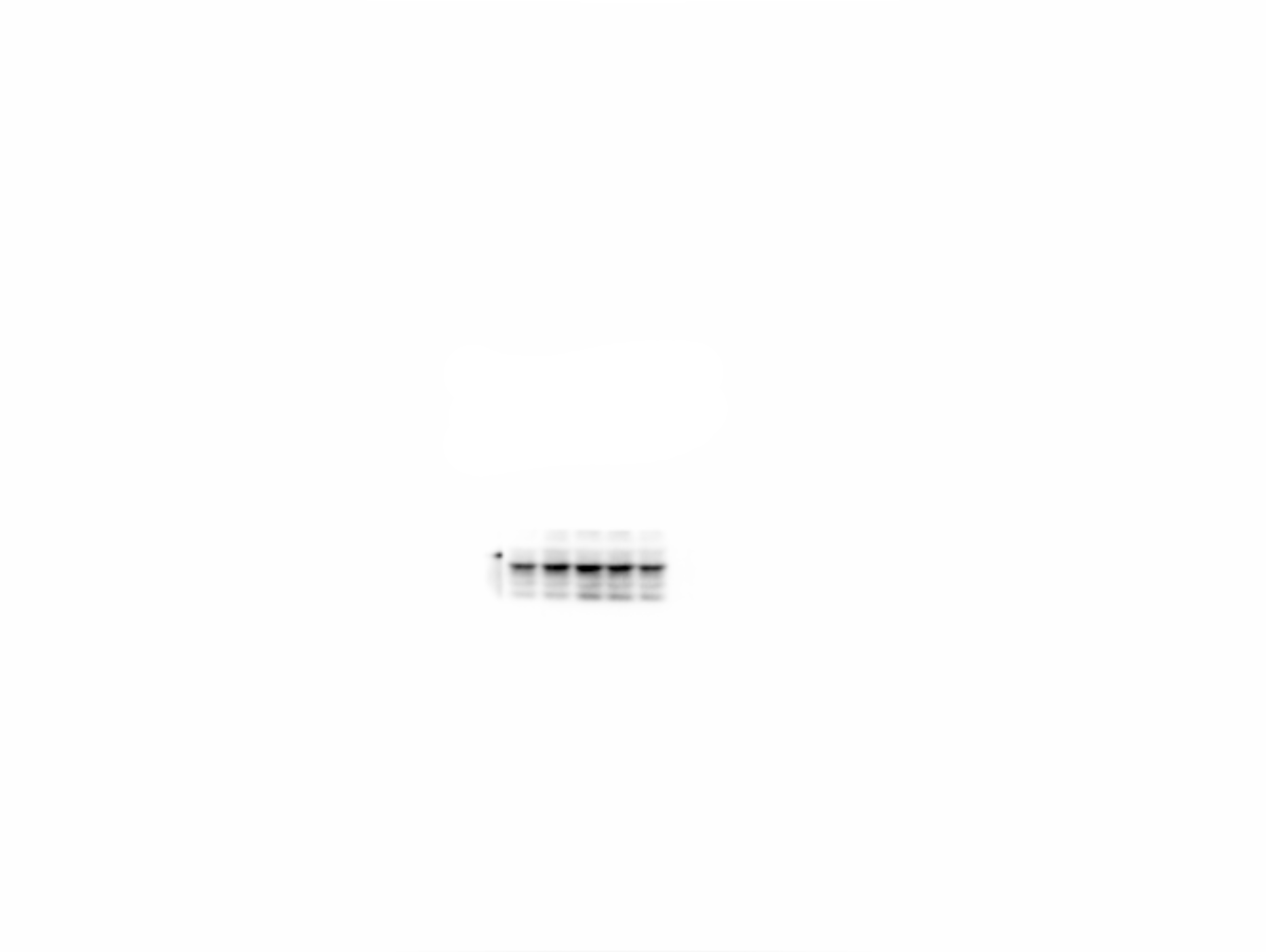

Supplement: S2 File — Original picture of the western blot experiments in the manuscript. (ZIP) [file pone.0274620.s002.zip › S2. blot results/Fig 3/CD34/4EA/2.tif]

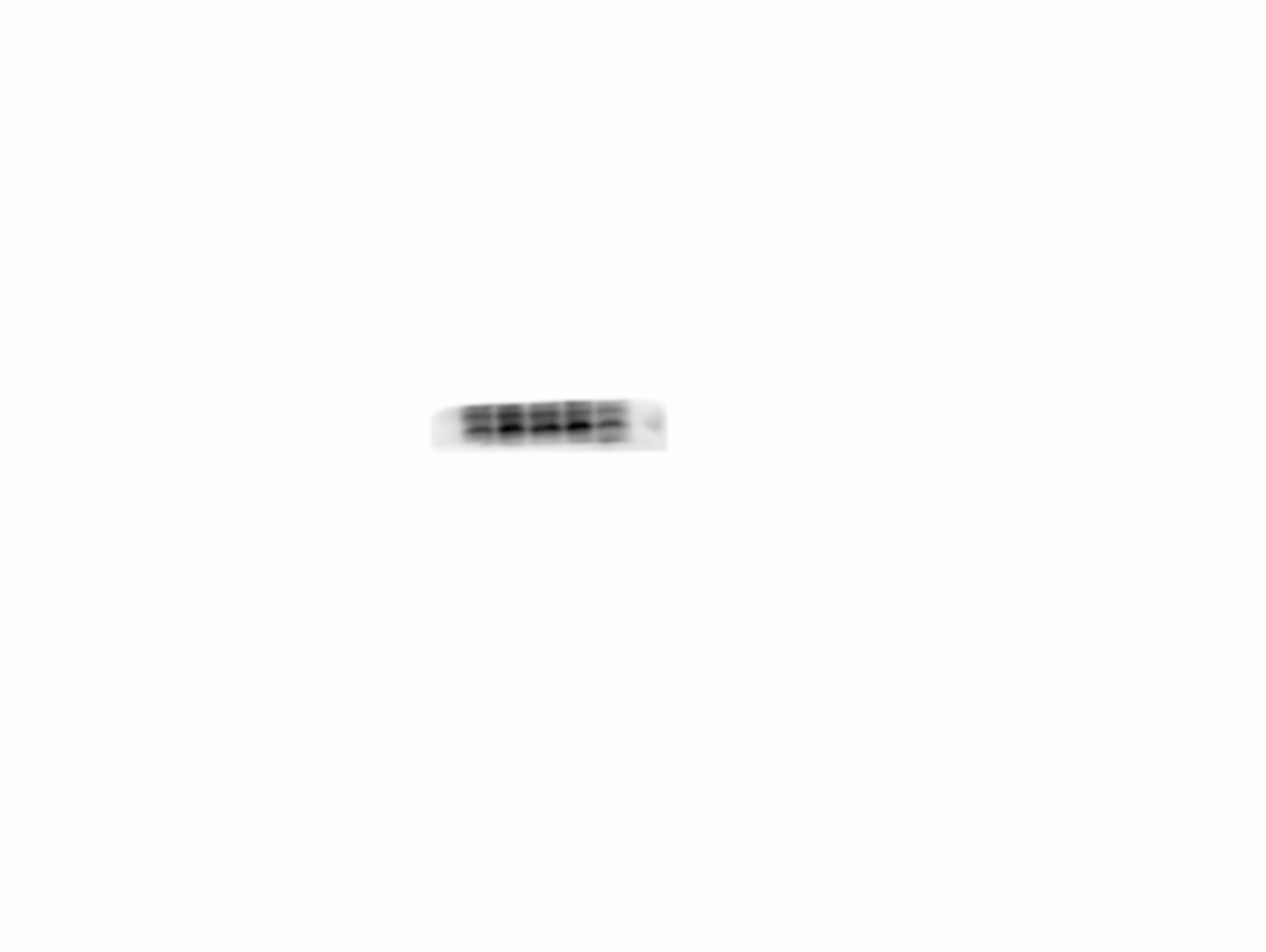

Supplement: S2 File — Original picture of the western blot experiments in the manuscript. (ZIP) [file pone.0274620.s002.zip › S2. blot results/Fig 3/CD34/4EA/3.tif]

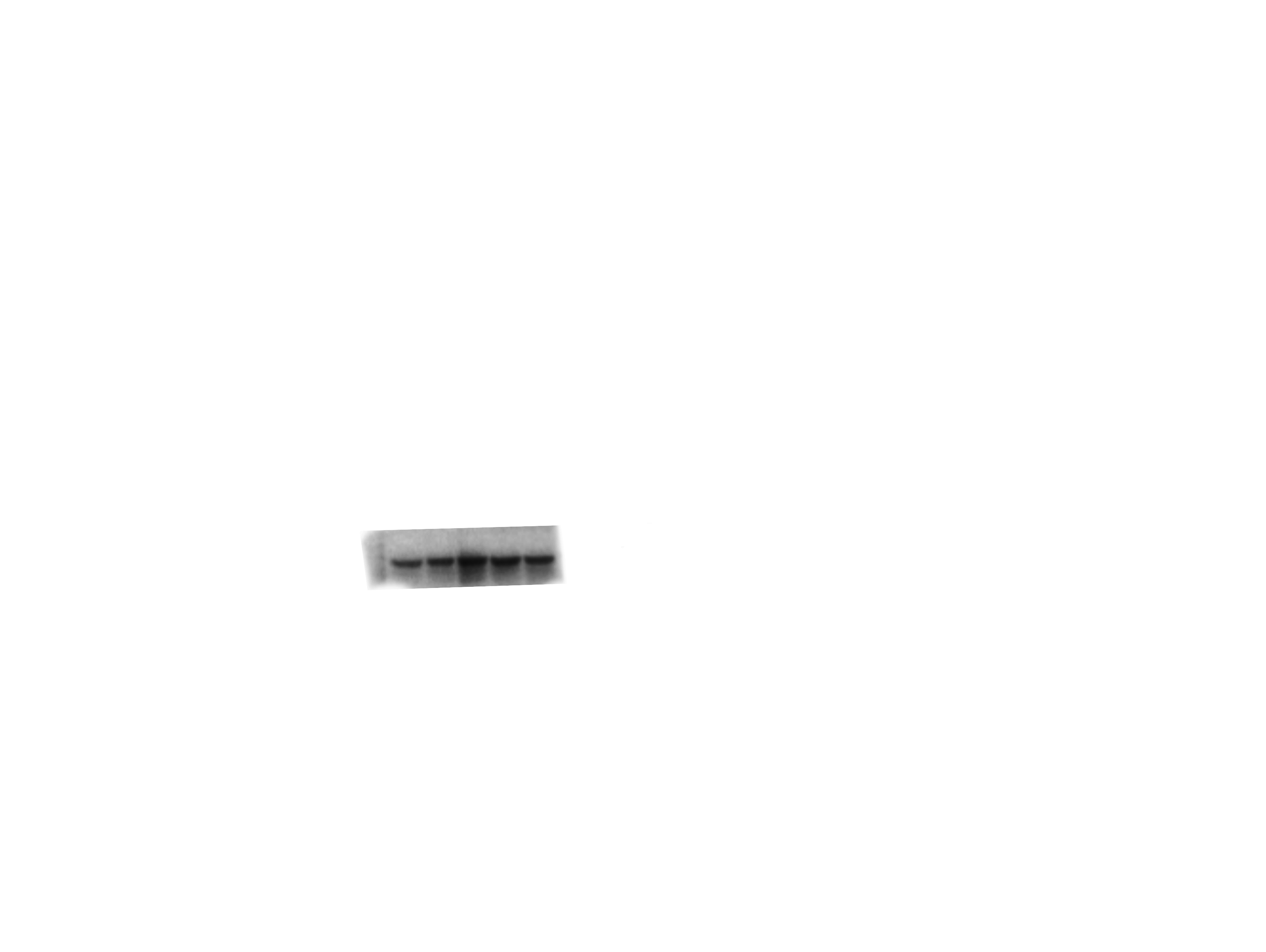

Supplement: S2 File — Original picture of the western blot experiments in the manuscript. (ZIP) [file pone.0274620.s002.zip › S2. blot results/Fig 3/CD34/4EA/4.tif]

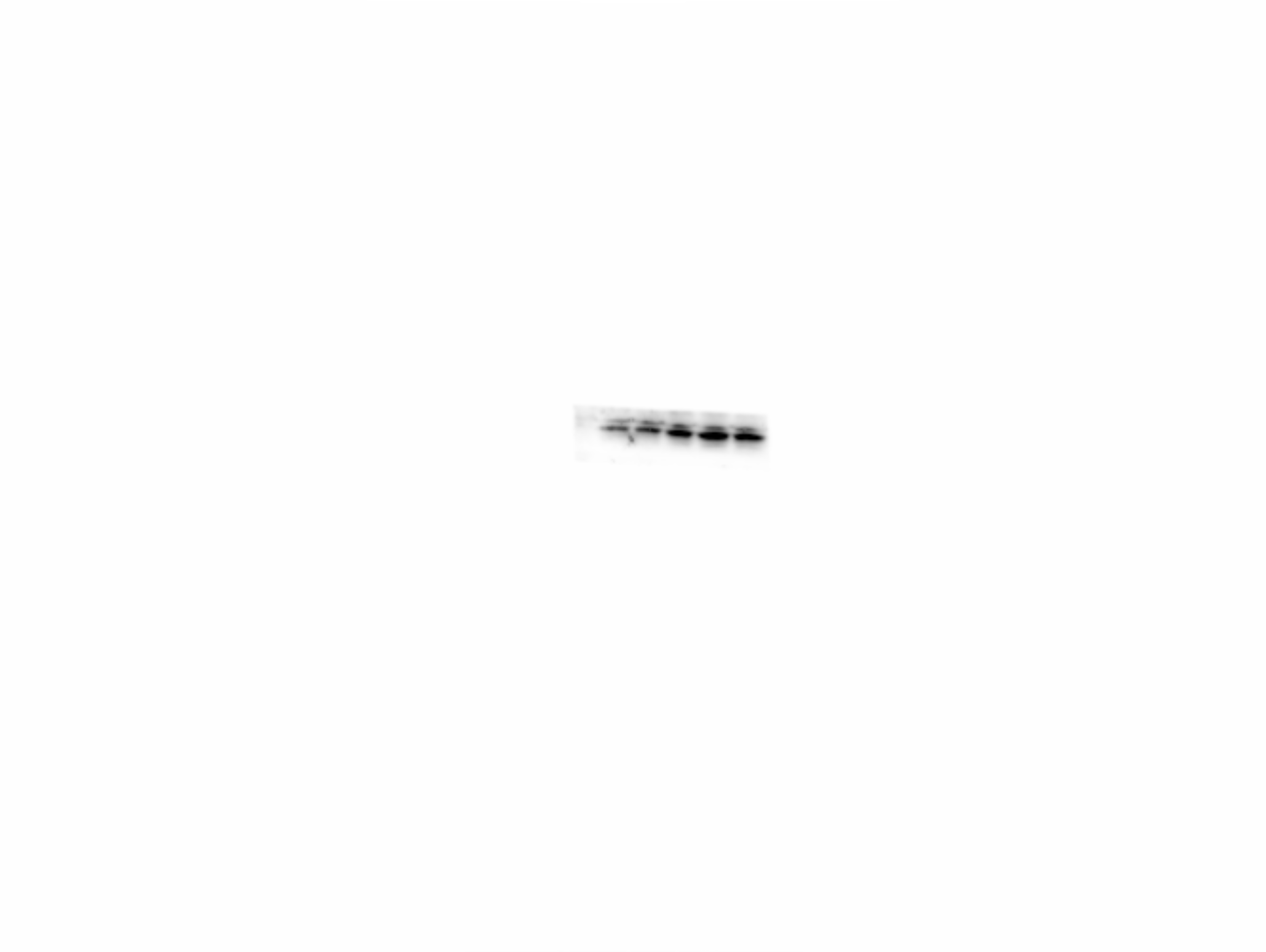

Supplement: S2 File — Original picture of the western blot experiments in the manuscript. (ZIP) [file pone.0274620.s002.zip › S2. blot results/Fig 3/CD34/4EA/5.tif]

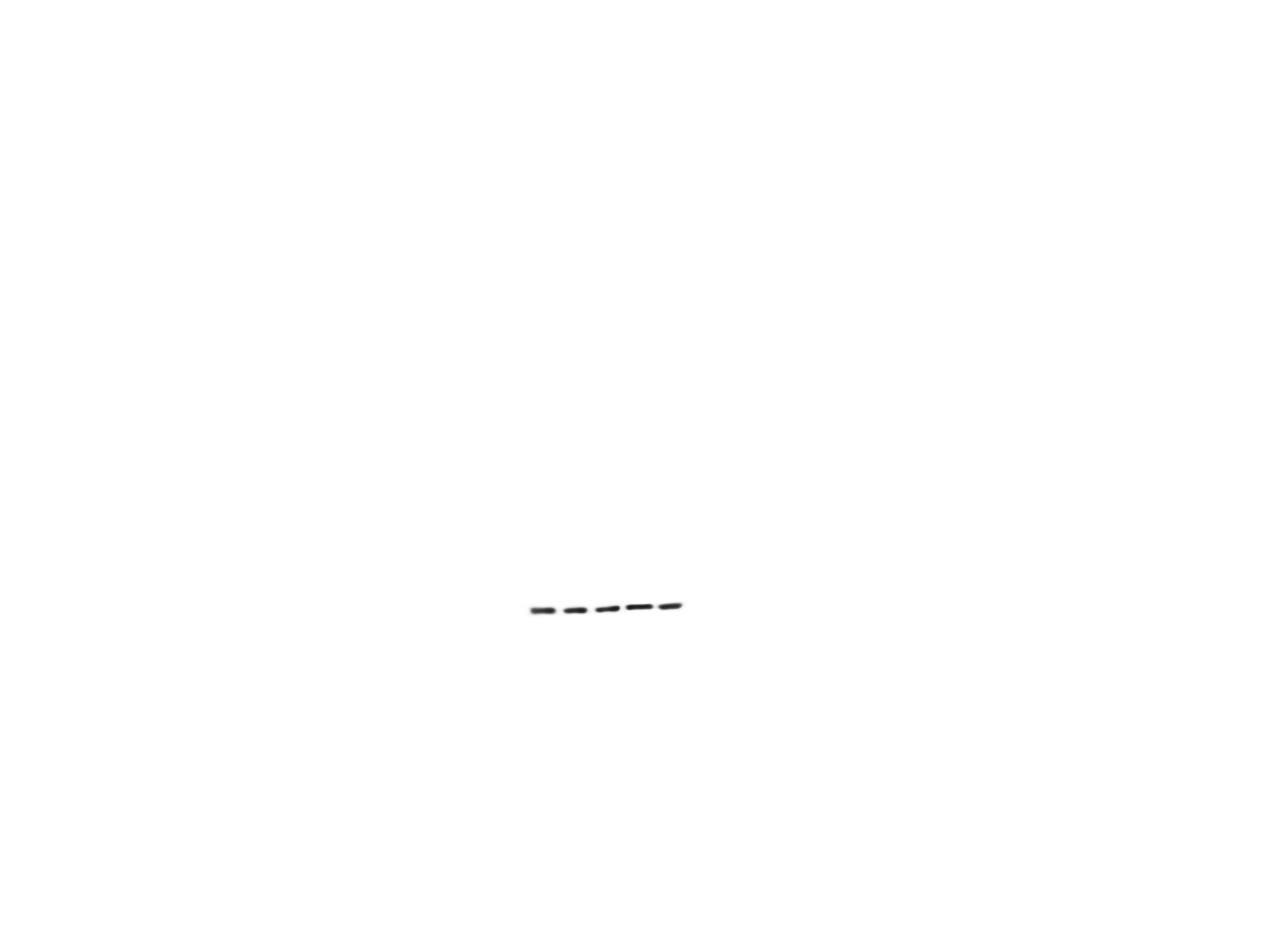

Supplement: S2 File — Original picture of the western blot experiments in the manuscript. (ZIP) [file pone.0274620.s002.zip › S2. blot results/Fig 3/EPO/1control/1.tif]

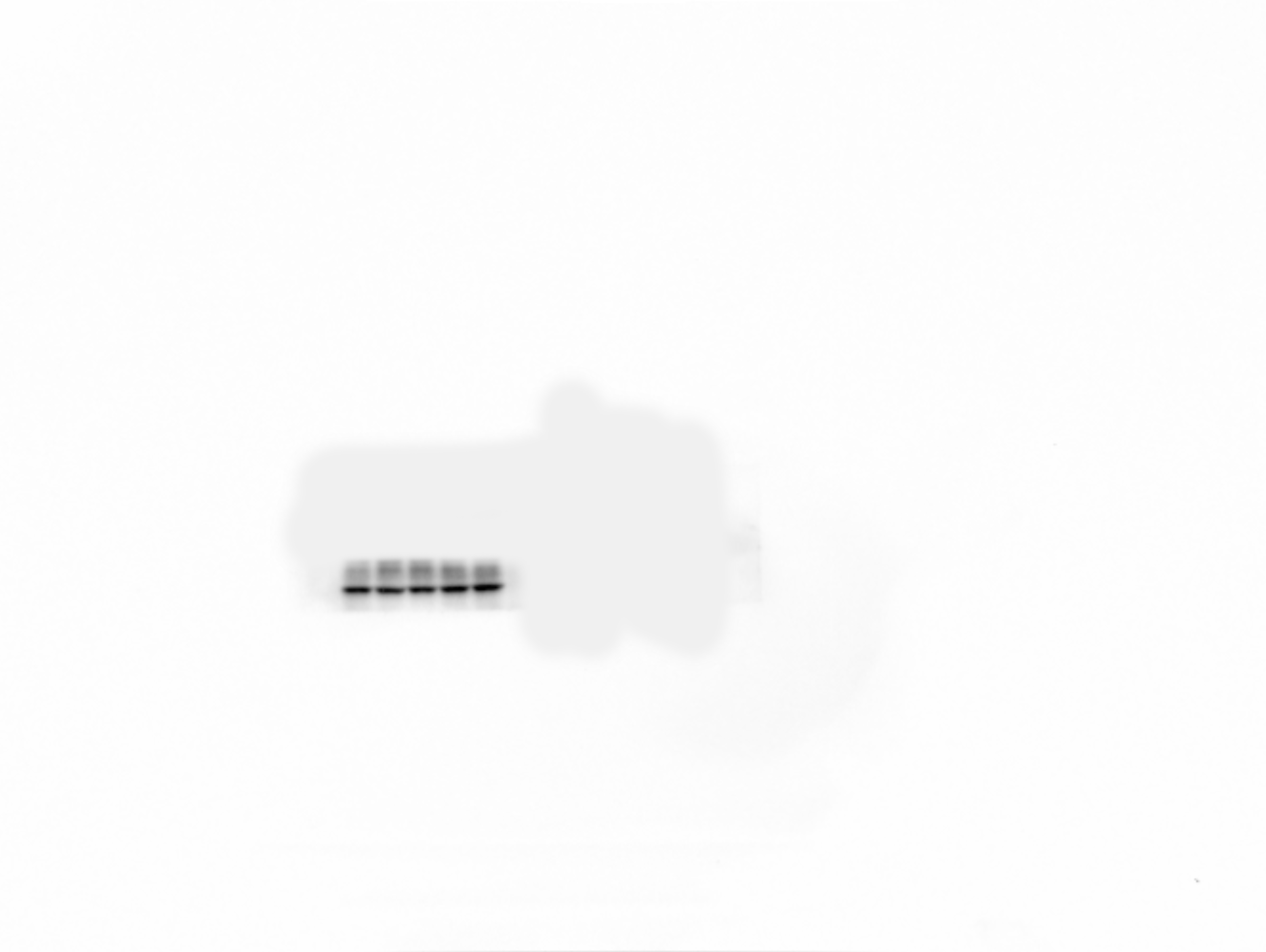

Supplement: S2 File — Original picture of the western blot experiments in the manuscript. (ZIP) [file pone.0274620.s002.zip › S2. blot results/Fig 3/EPO/1control/2.tif]

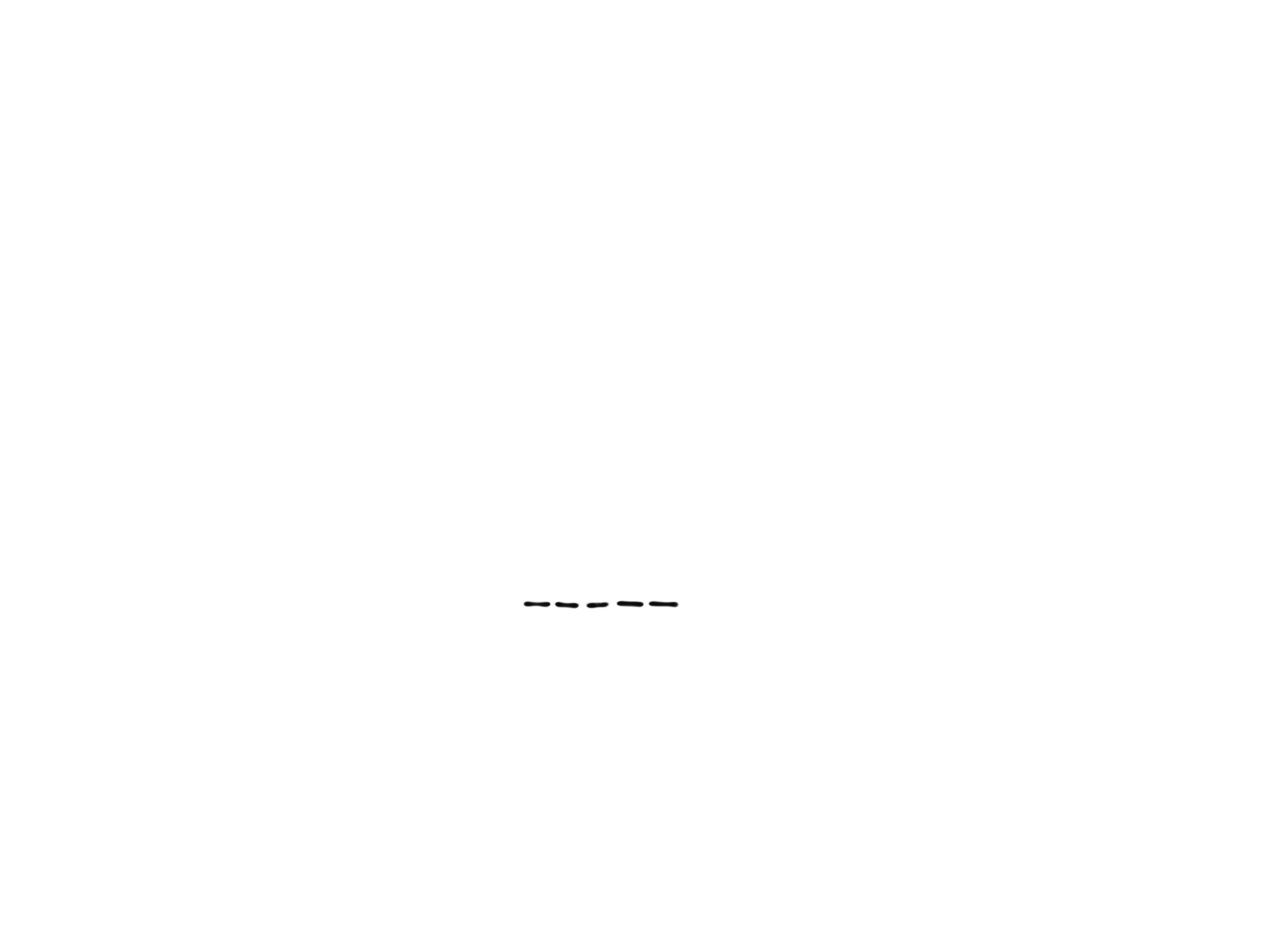

Supplement: S2 File — Original picture of the western blot experiments in the manuscript. (ZIP) [file pone.0274620.s002.zip › S2. blot results/Fig 3/EPO/1control/3.tif]

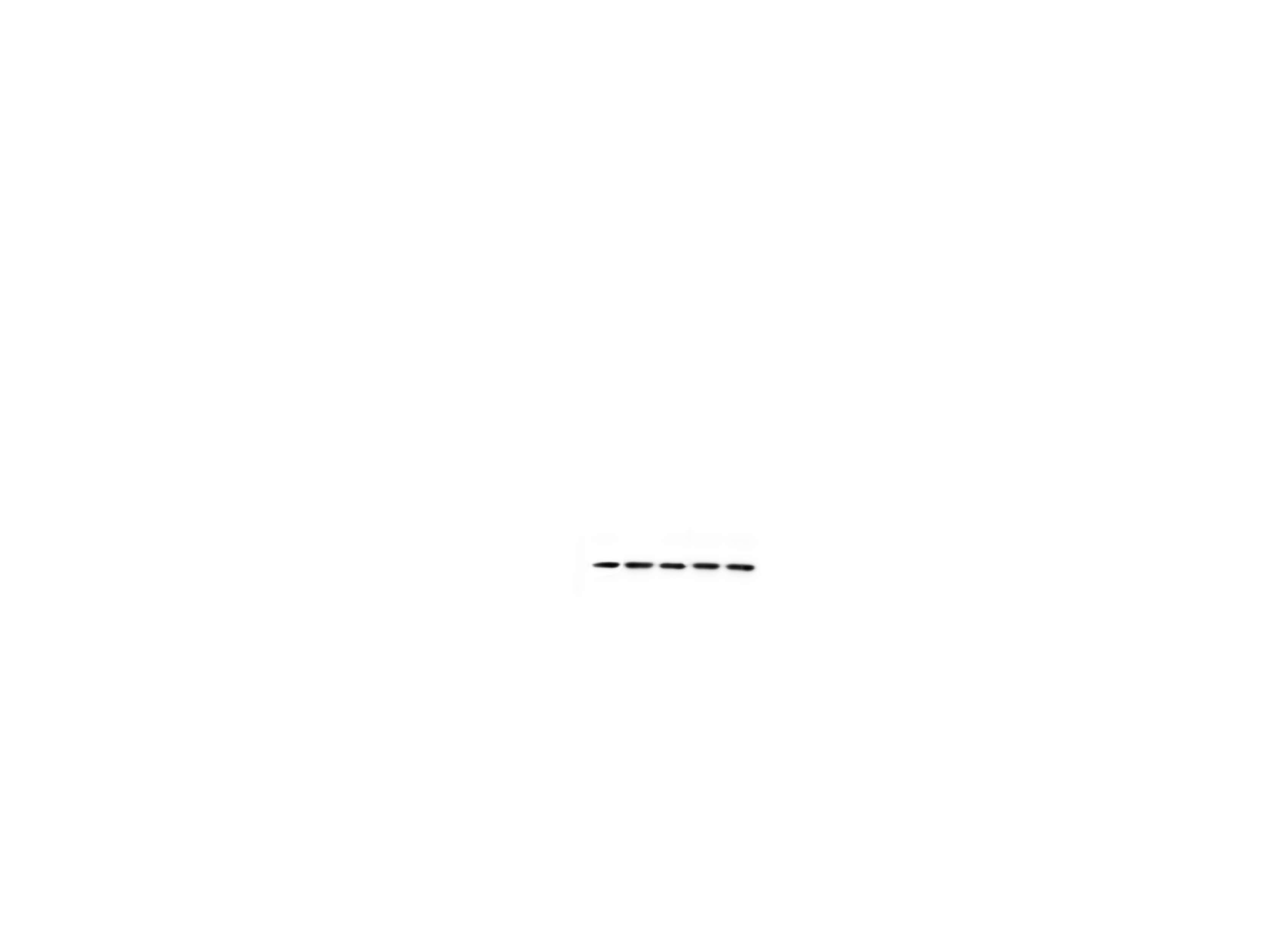

Supplement: S2 File — Original picture of the western blot experiments in the manuscript. (ZIP) [file pone.0274620.s002.zip › S2. blot results/Fig 3/EPO/1control/4.tif]

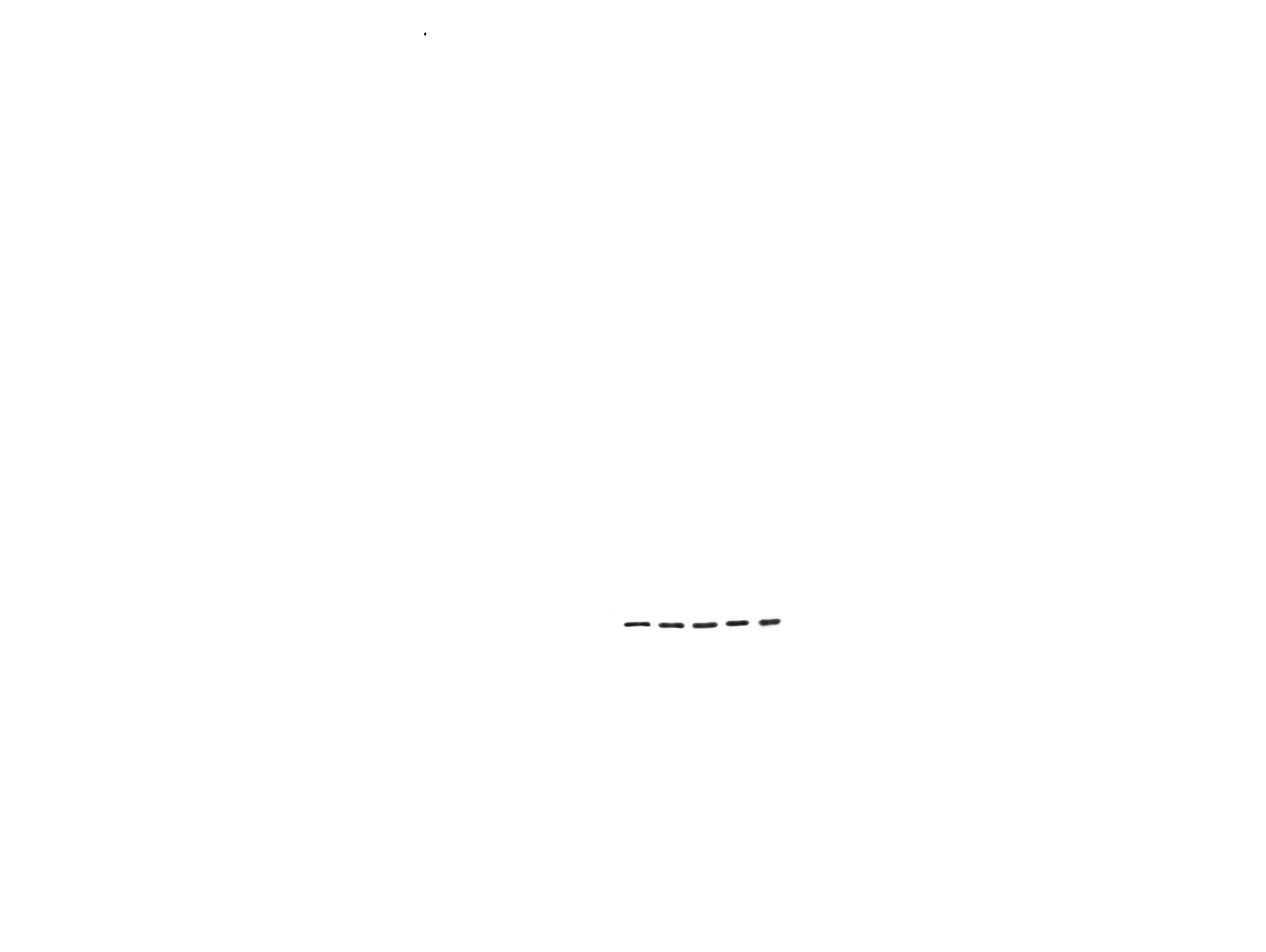

Supplement: S2 File — Original picture of the western blot experiments in the manuscript. (ZIP) [file pone.0274620.s002.zip › S2. blot results/Fig 3/EPO/1control/5.tif]

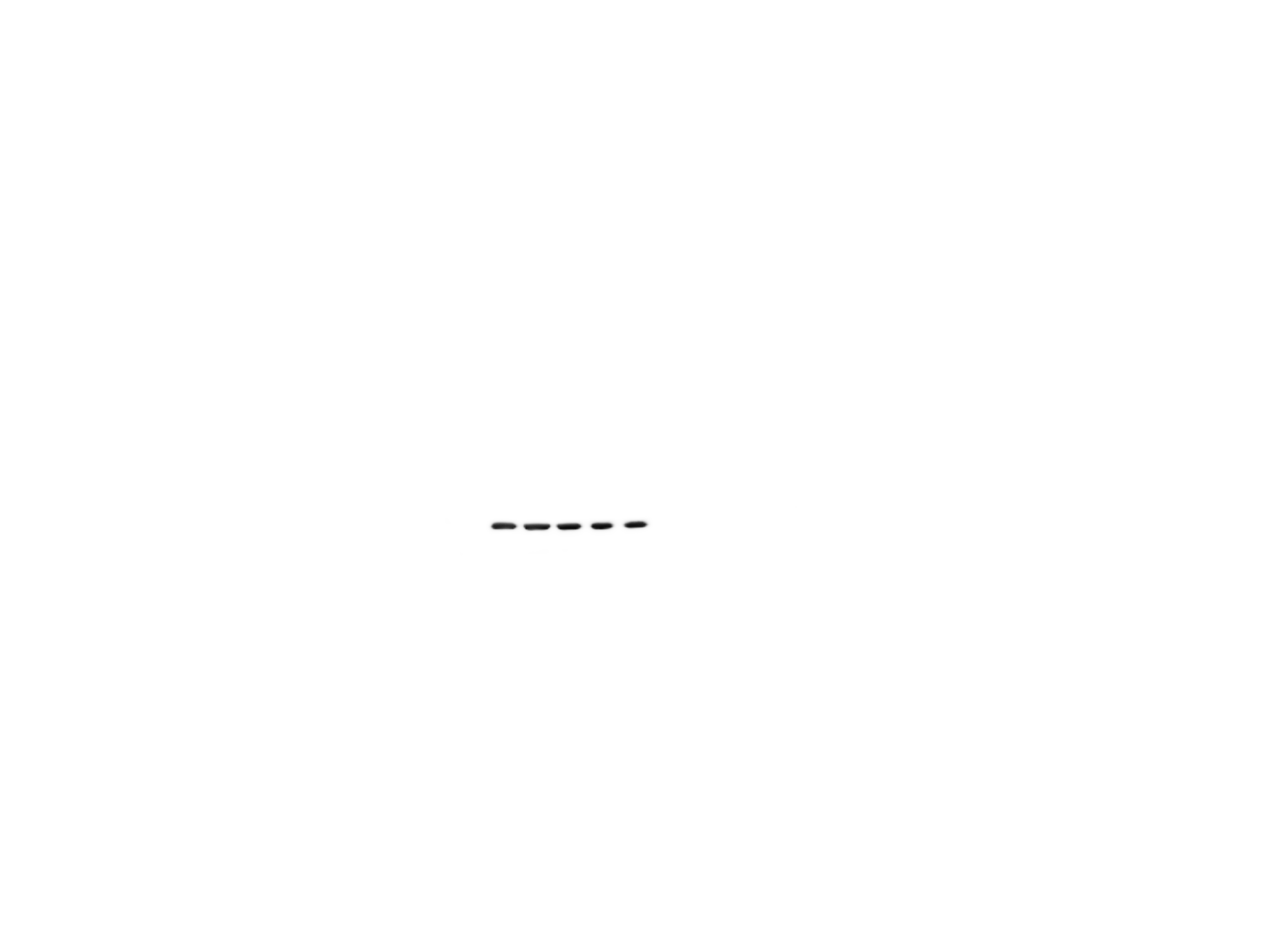

Supplement: S2 File — Original picture of the western blot experiments in the manuscript. (ZIP) [file pone.0274620.s002.zip › S2. blot results/Fig 3/EPO/2sham/1.tif]

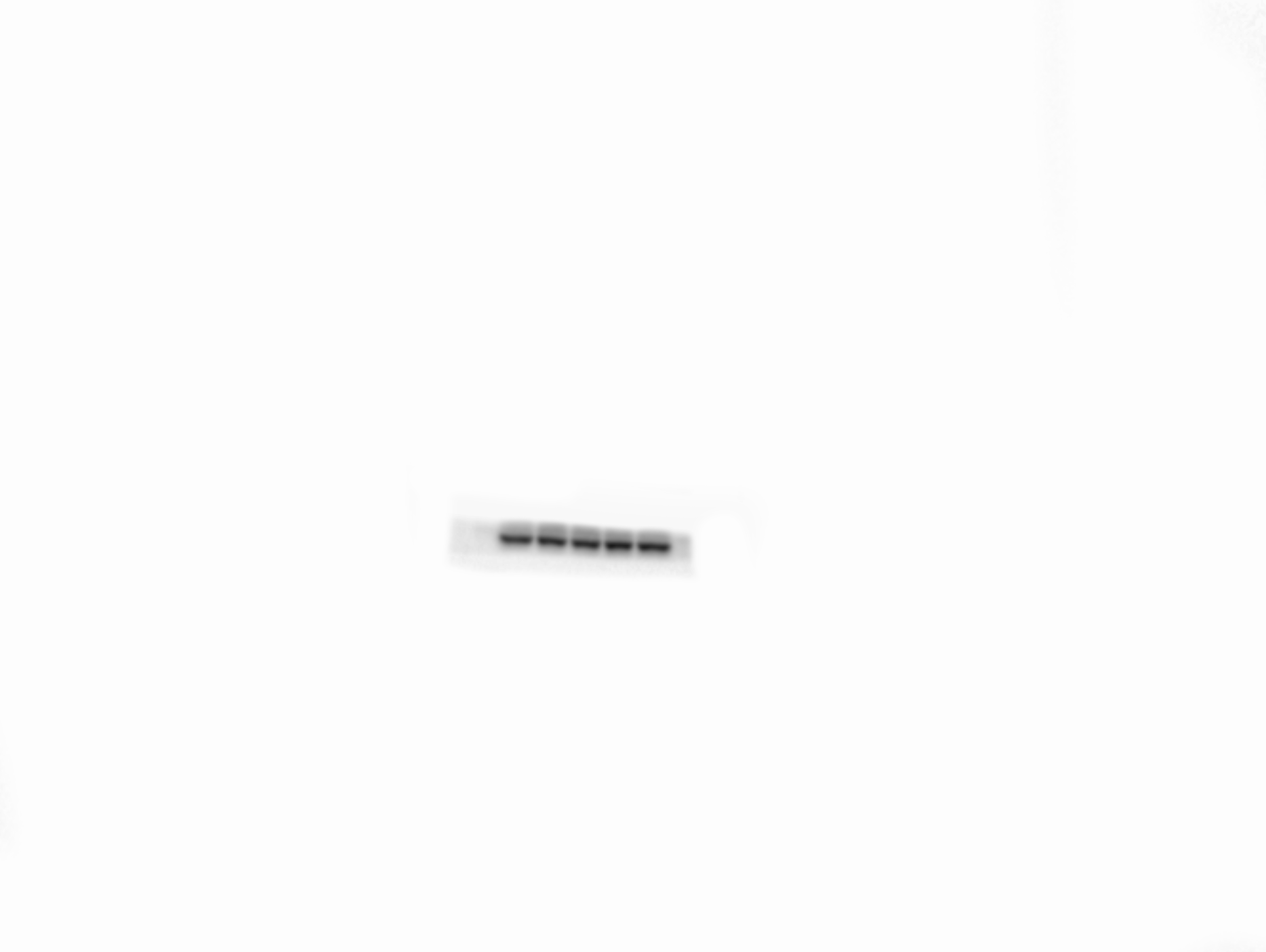

Supplement: S2 File — Original picture of the western blot experiments in the manuscript. (ZIP) [file pone.0274620.s002.zip › S2. blot results/Fig 3/EPO/2sham/2.tif]

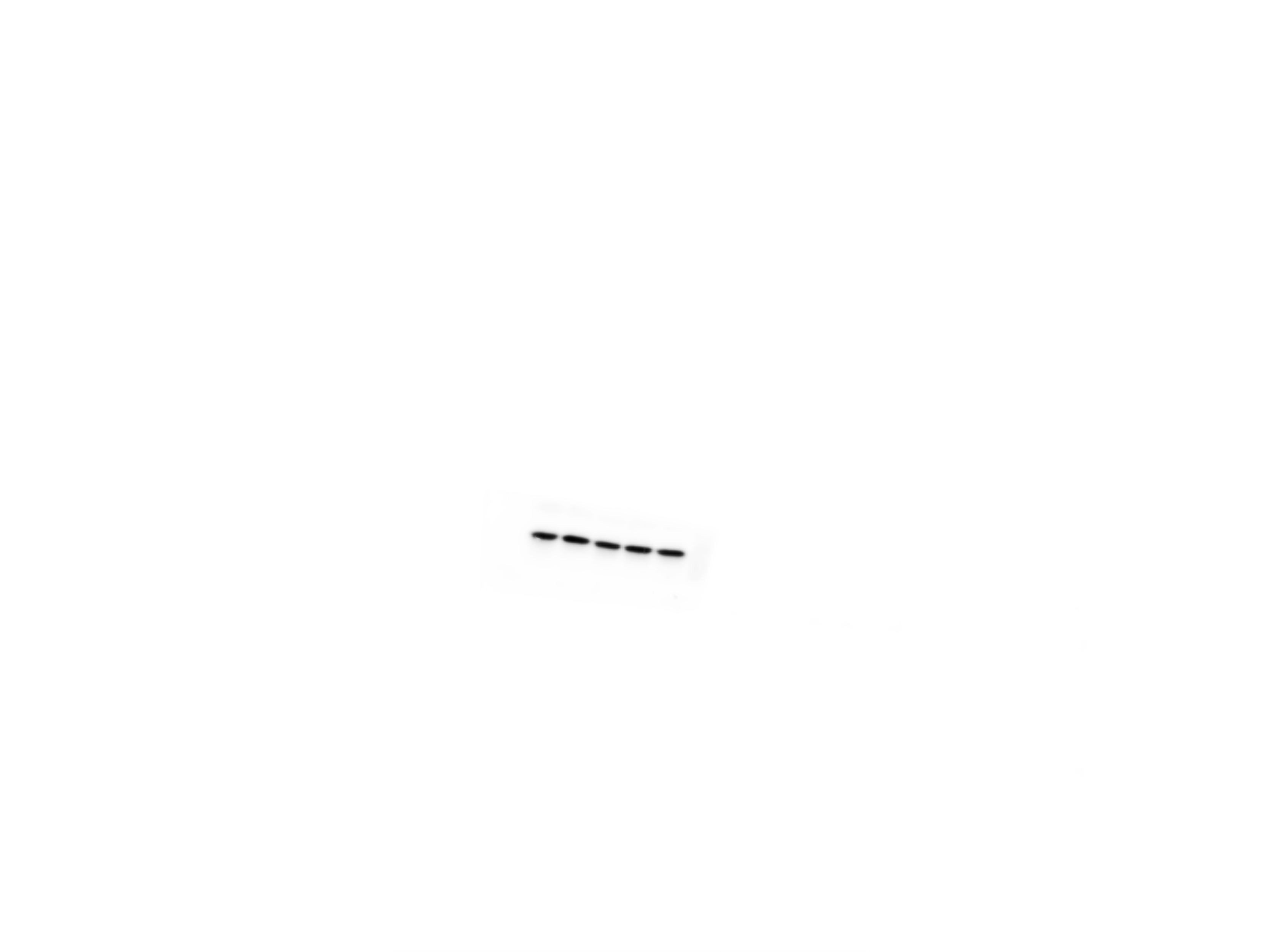

Supplement: S2 File — Original picture of the western blot experiments in the manuscript. (ZIP) [file pone.0274620.s002.zip › S2. blot results/Fig 3/EPO/2sham/3.tif]

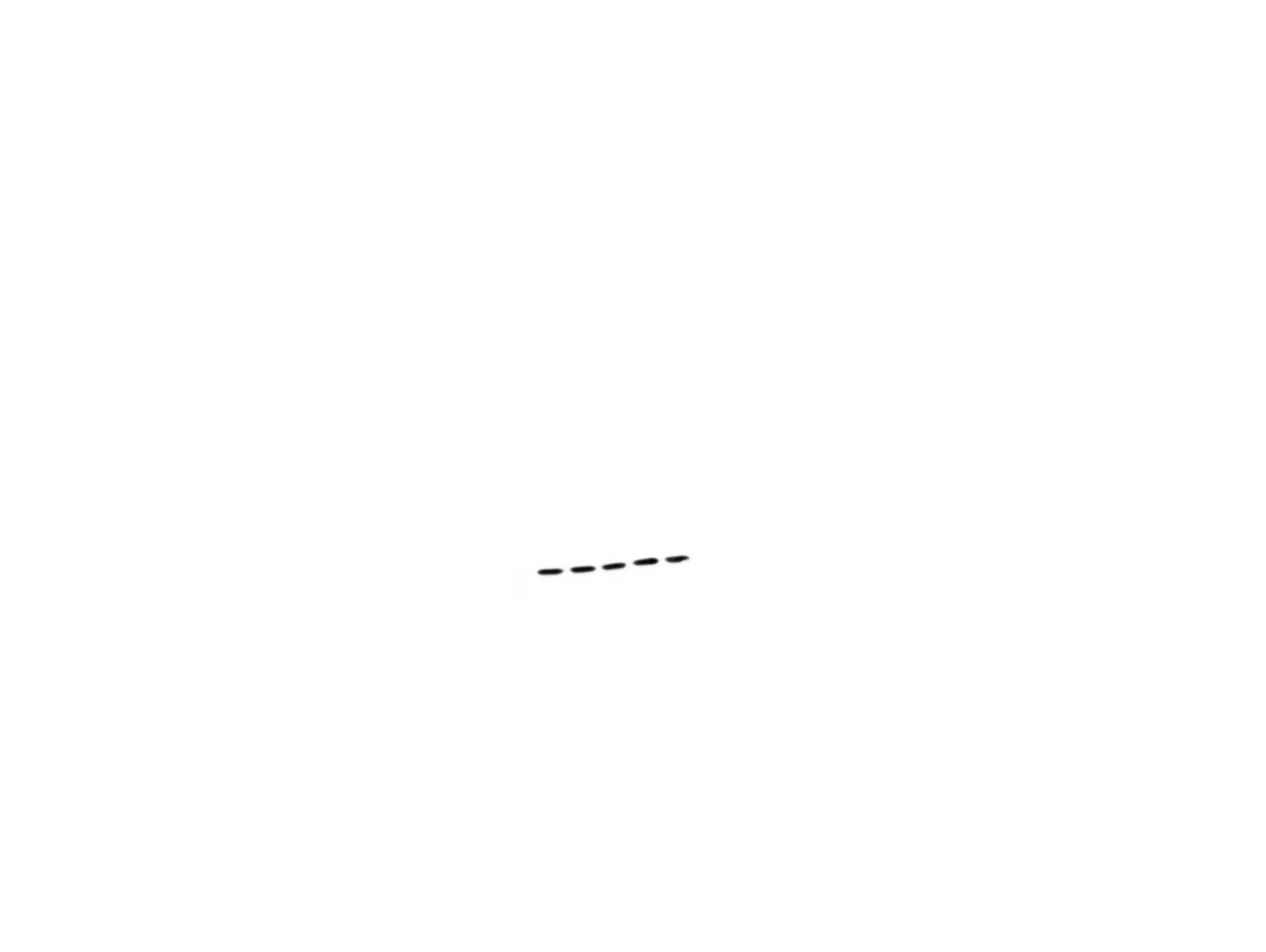

Supplement: S2 File — Original picture of the western blot experiments in the manuscript. (ZIP) [file pone.0274620.s002.zip › S2. blot results/Fig 3/EPO/2sham/4.tif]

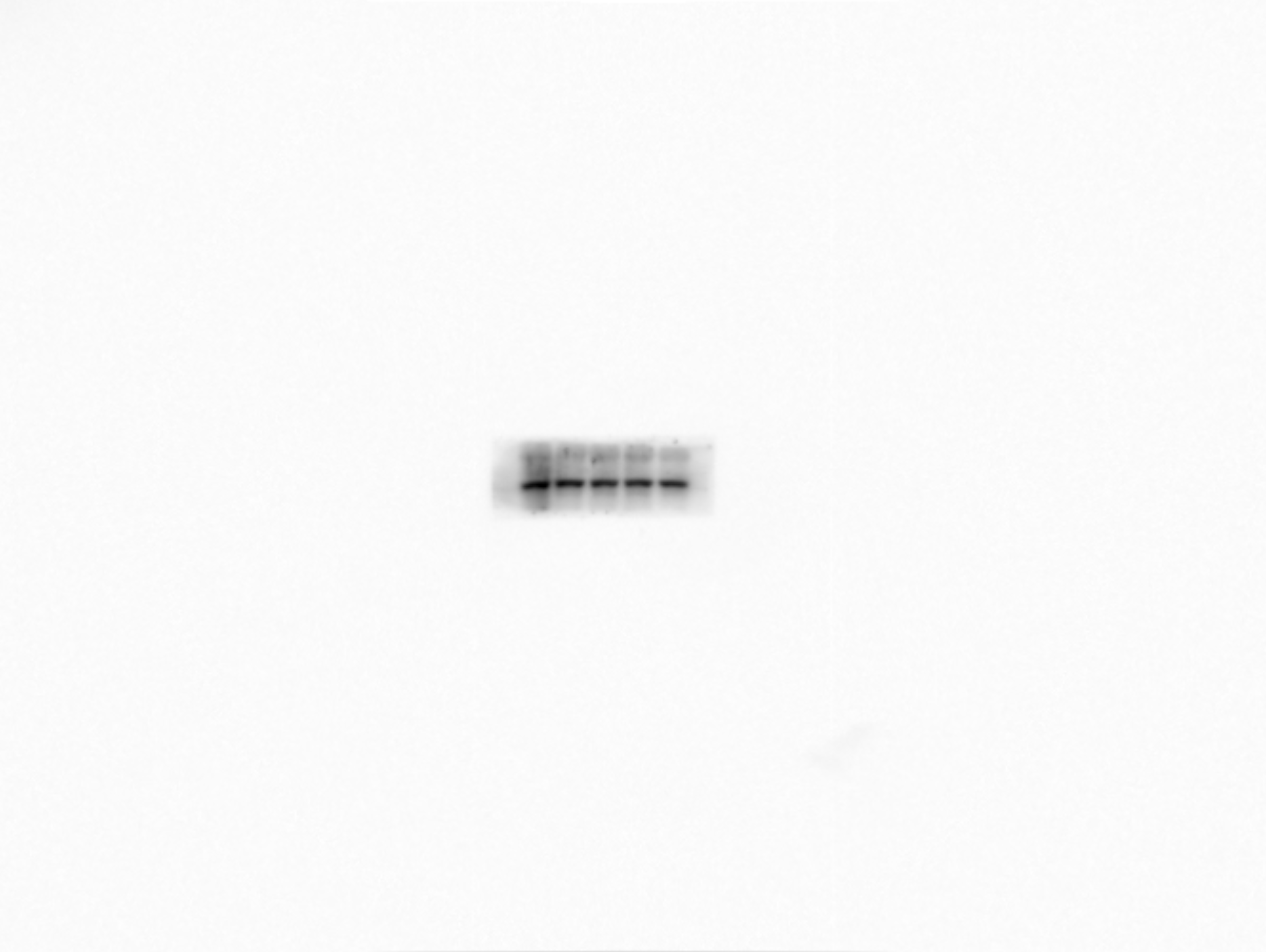

Supplement: S2 File — Original picture of the western blot experiments in the manuscript. (ZIP) [file pone.0274620.s002.zip › S2. blot results/Fig 3/EPO/2sham/5.tif]

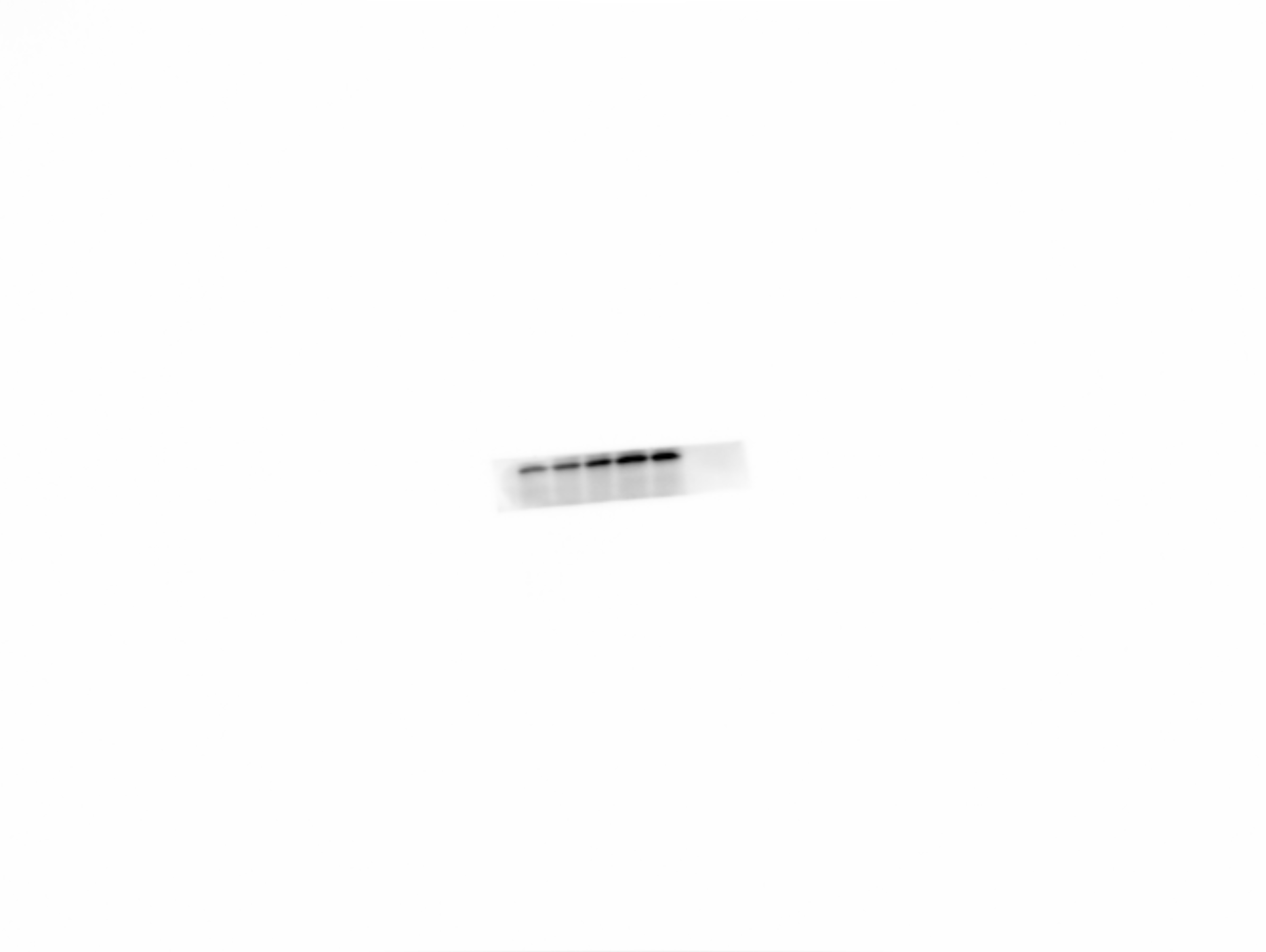

Supplement: S2 File — Original picture of the western blot experiments in the manuscript. (ZIP) [file pone.0274620.s002.zip › S2. blot results/Fig 3/EPO/3model/1.tif]

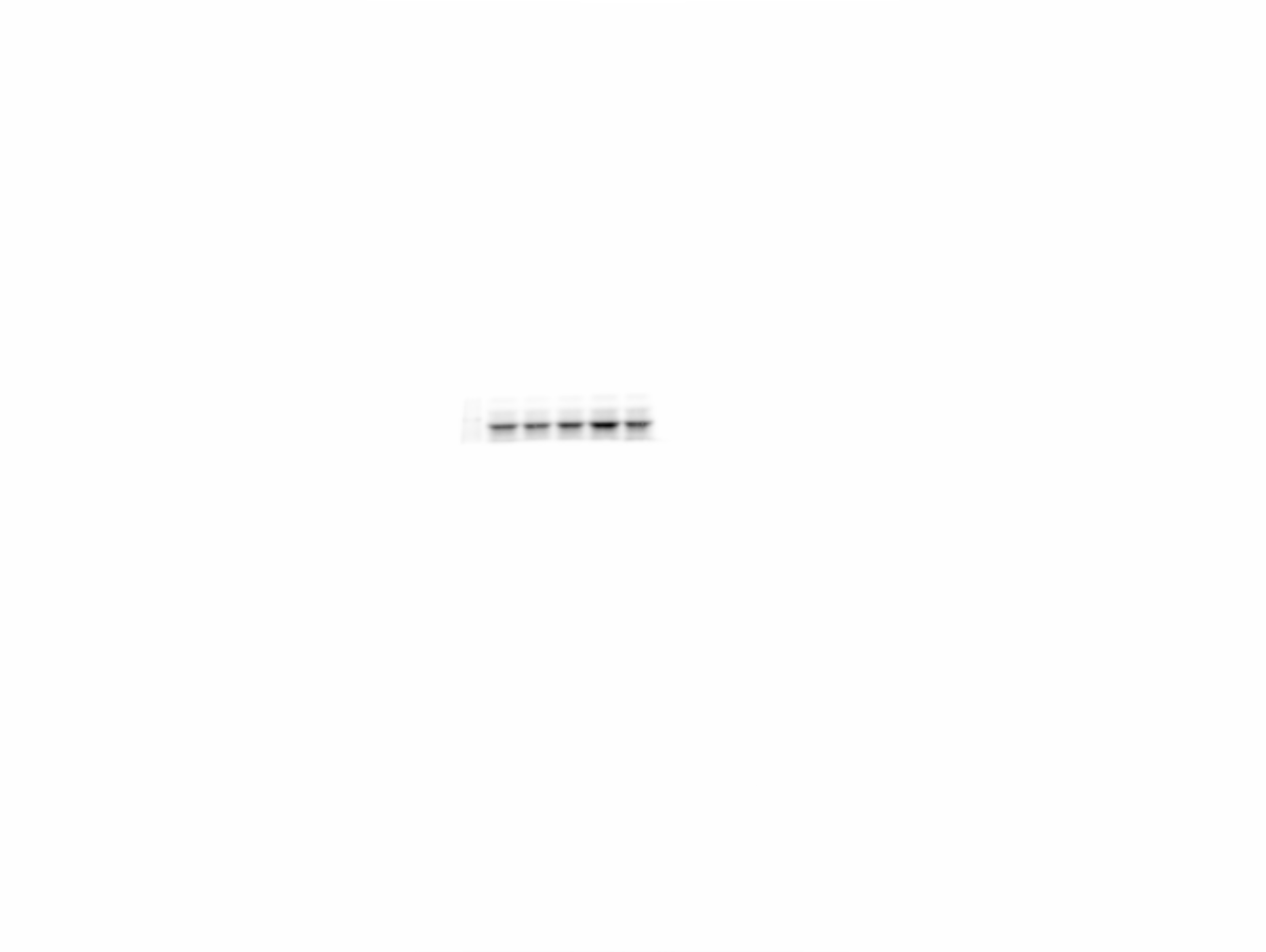

Supplement: S2 File — Original picture of the western blot experiments in the manuscript. (ZIP) [file pone.0274620.s002.zip › S2. blot results/Fig 3/EPO/3model/2.tif]

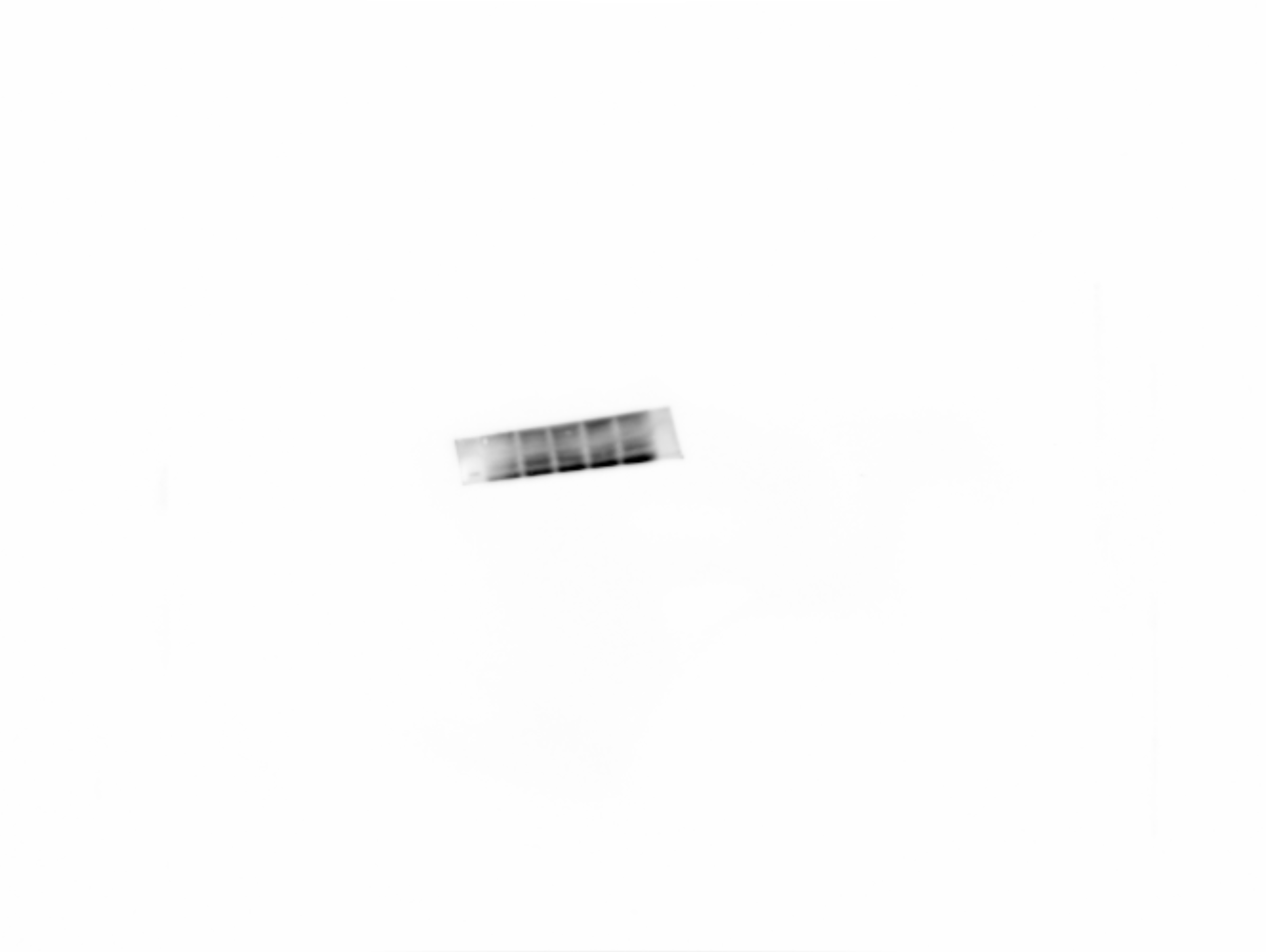

Supplement: S2 File — Original picture of the western blot experiments in the manuscript. (ZIP) [file pone.0274620.s002.zip › S2. blot results/Fig 3/EPO/3model/3.tif]

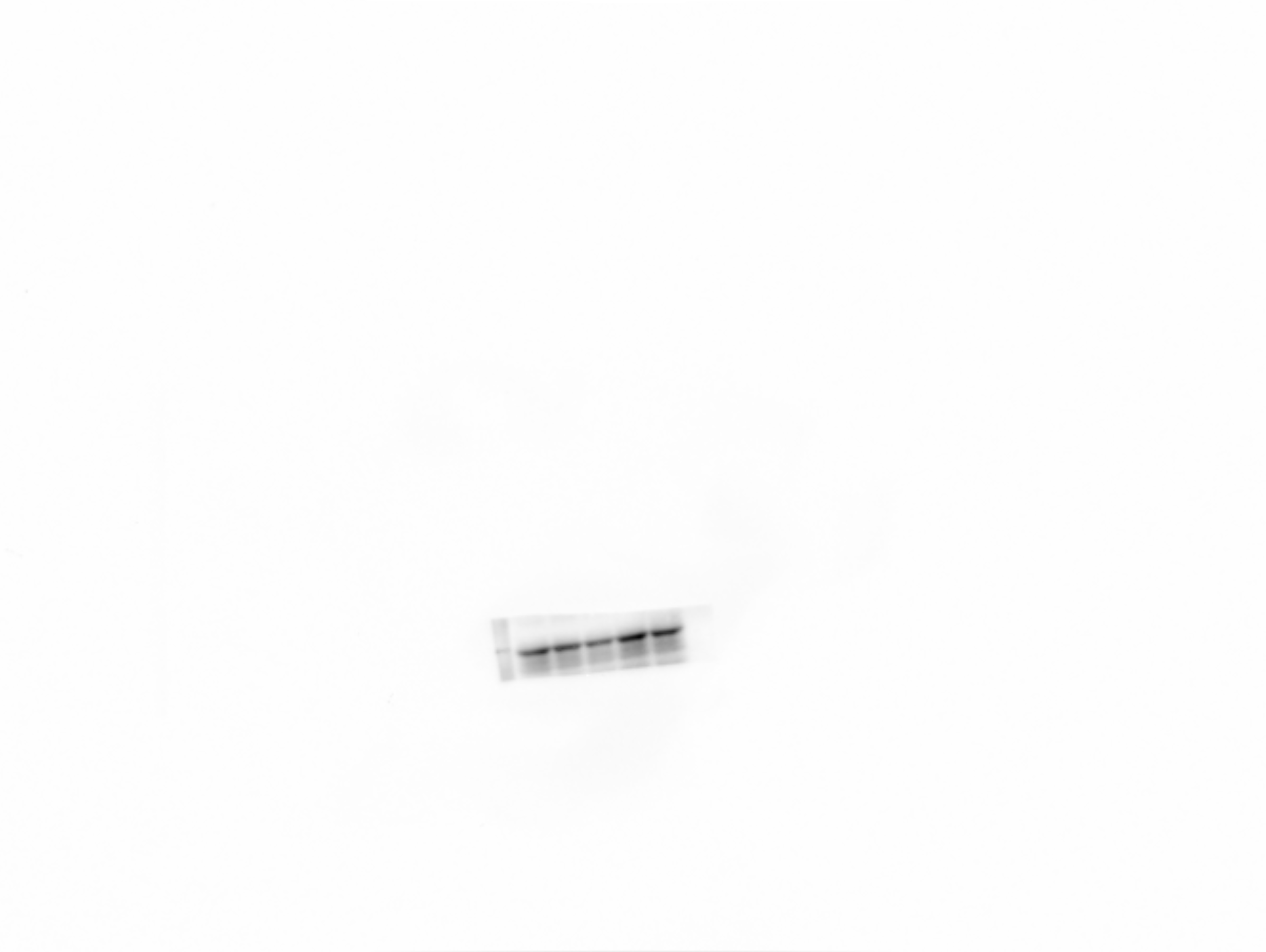

Supplement: S2 File — Original picture of the western blot experiments in the manuscript. (ZIP) [file pone.0274620.s002.zip › S2. blot results/Fig 3/EPO/3model/4.tif]

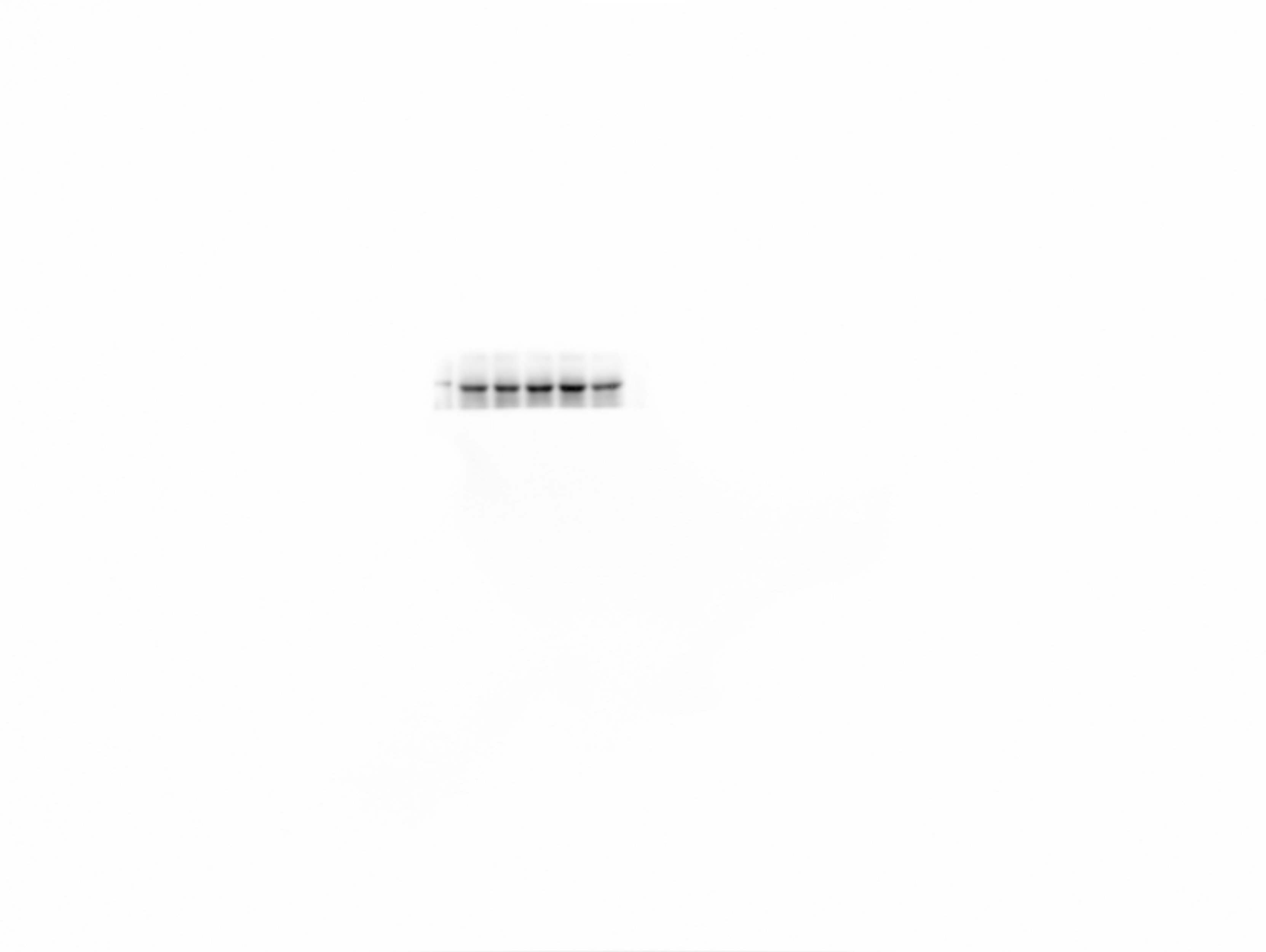

Supplement: S2 File — Original picture of the western blot experiments in the manuscript. (ZIP) [file pone.0274620.s002.zip › S2. blot results/Fig 3/EPO/3model/5.tif]

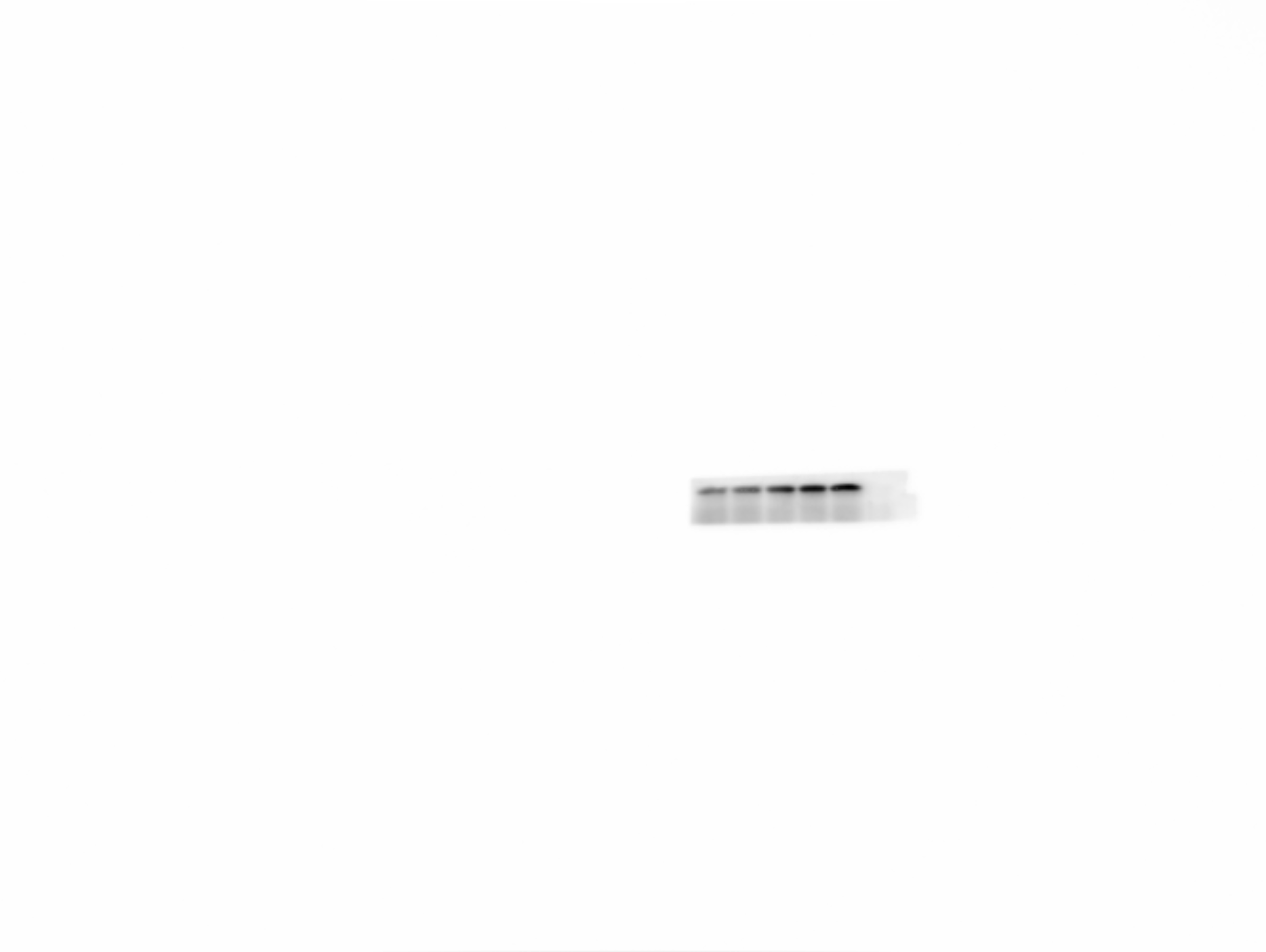

Supplement: S2 File — Original picture of the western blot experiments in the manuscript. (ZIP) [file pone.0274620.s002.zip › S2. blot results/Fig 3/EPO/4EA/1.tif]

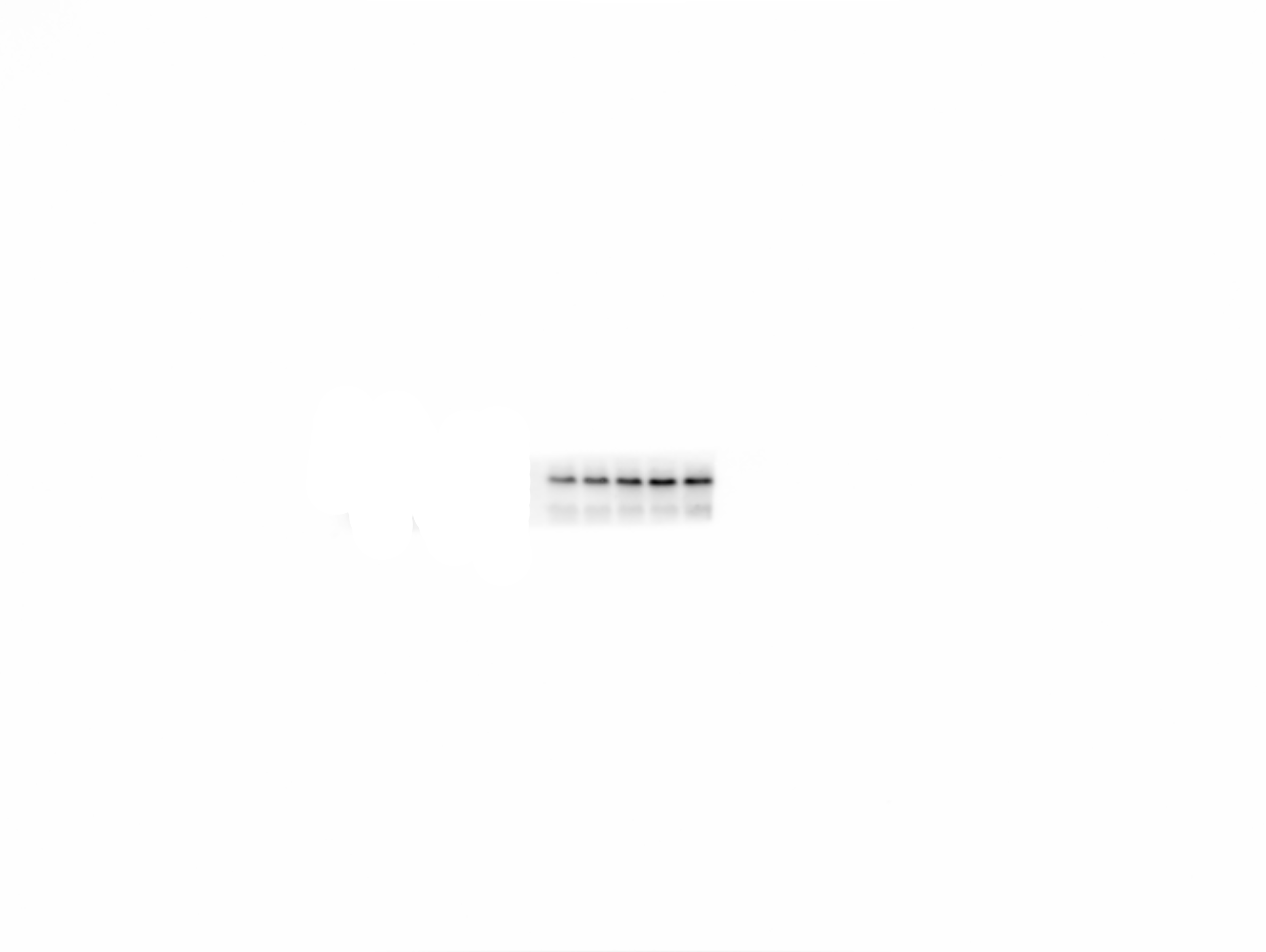

Supplement: S2 File — Original picture of the western blot experiments in the manuscript. (ZIP) [file pone.0274620.s002.zip › S2. blot results/Fig 3/EPO/4EA/2.tif]

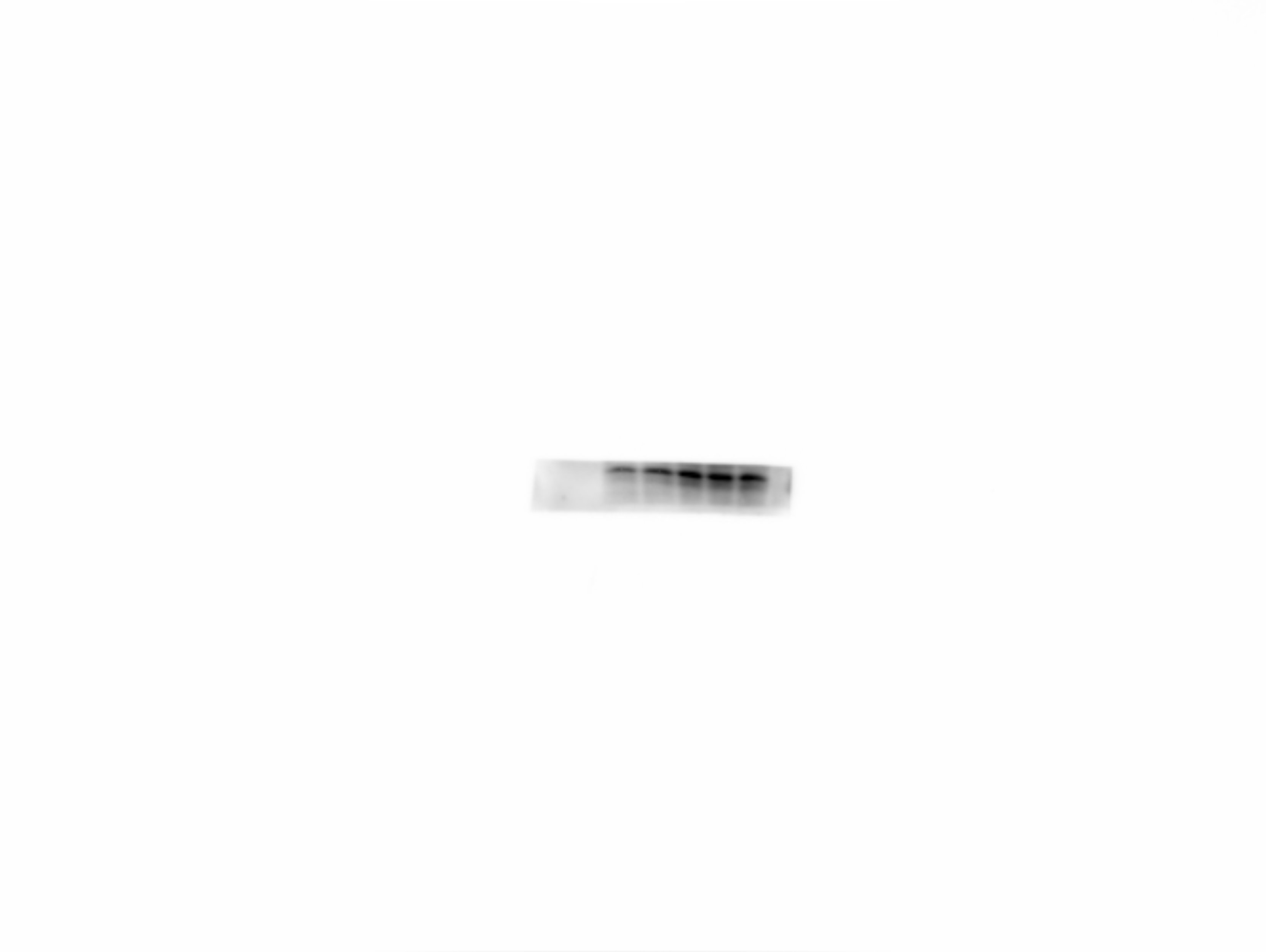

Supplement: S2 File — Original picture of the western blot experiments in the manuscript. (ZIP) [file pone.0274620.s002.zip › S2. blot results/Fig 3/EPO/4EA/3.tif]

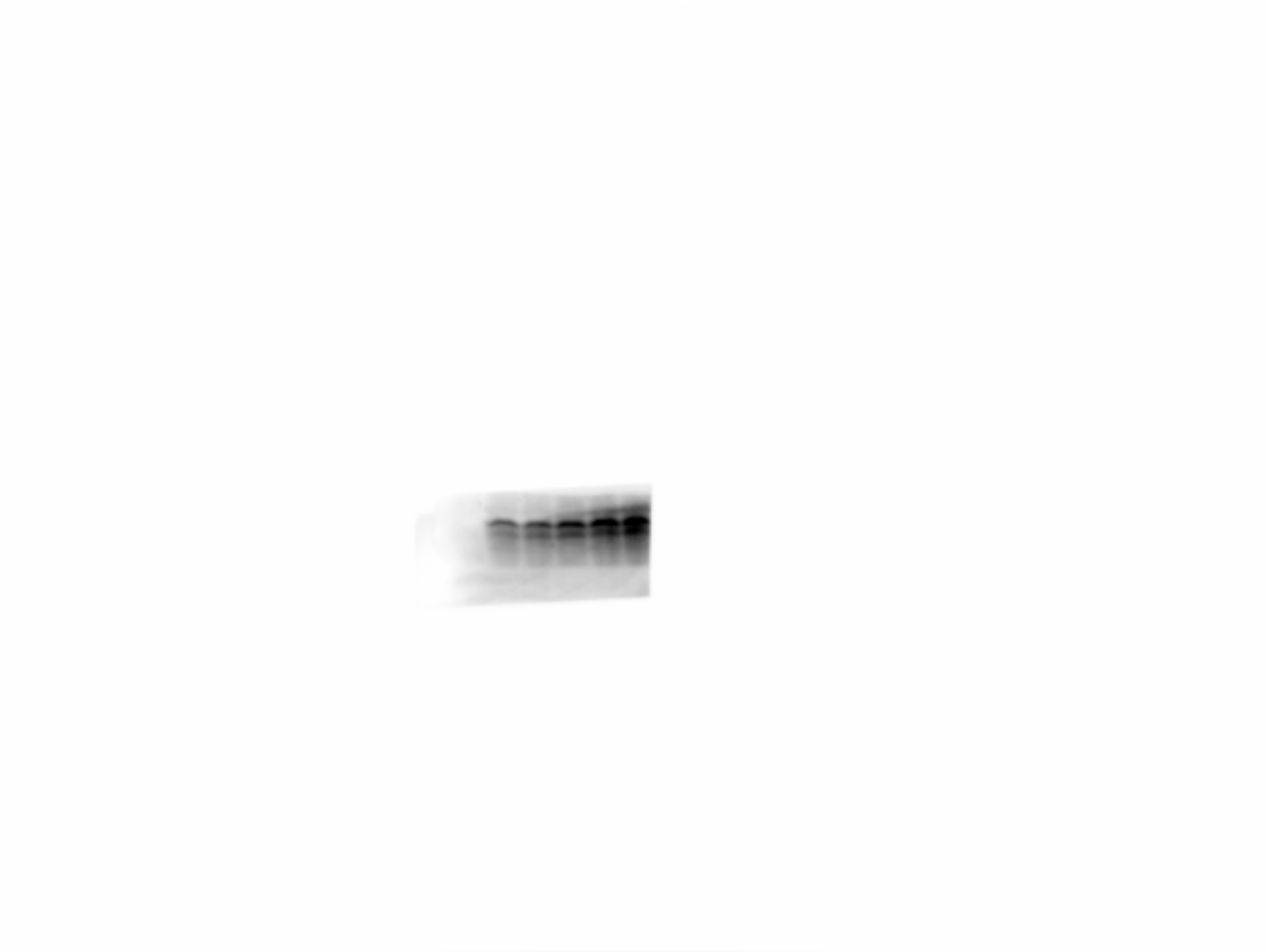

Supplement: S2 File — Original picture of the western blot experiments in the manuscript. (ZIP) [file pone.0274620.s002.zip › S2. blot results/Fig 3/EPO/4EA/4.tif]

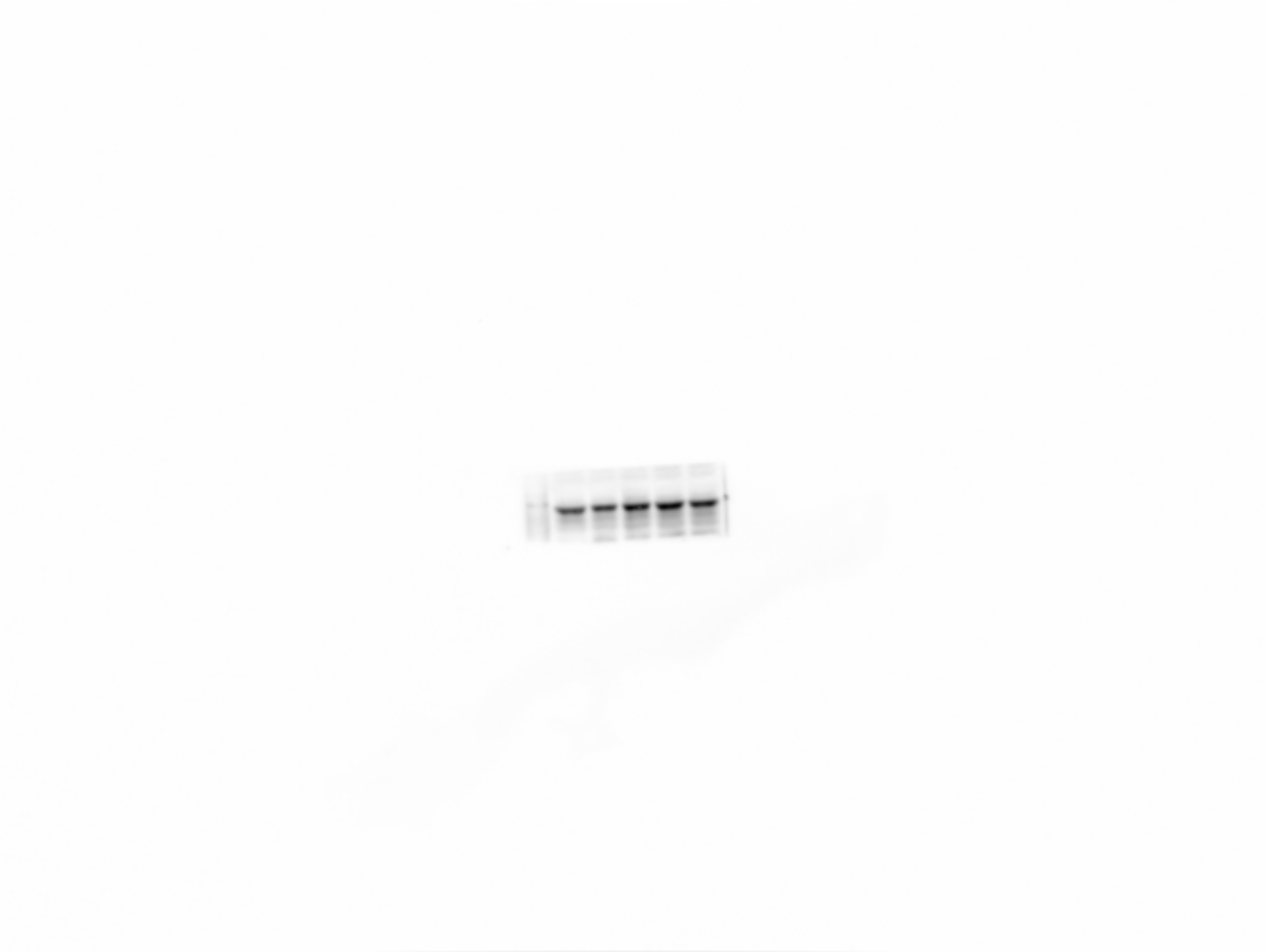

Supplement: S2 File — Original picture of the western blot experiments in the manuscript. (ZIP) [file pone.0274620.s002.zip › S2. blot results/Fig 3/EPO/4EA/5.tif]

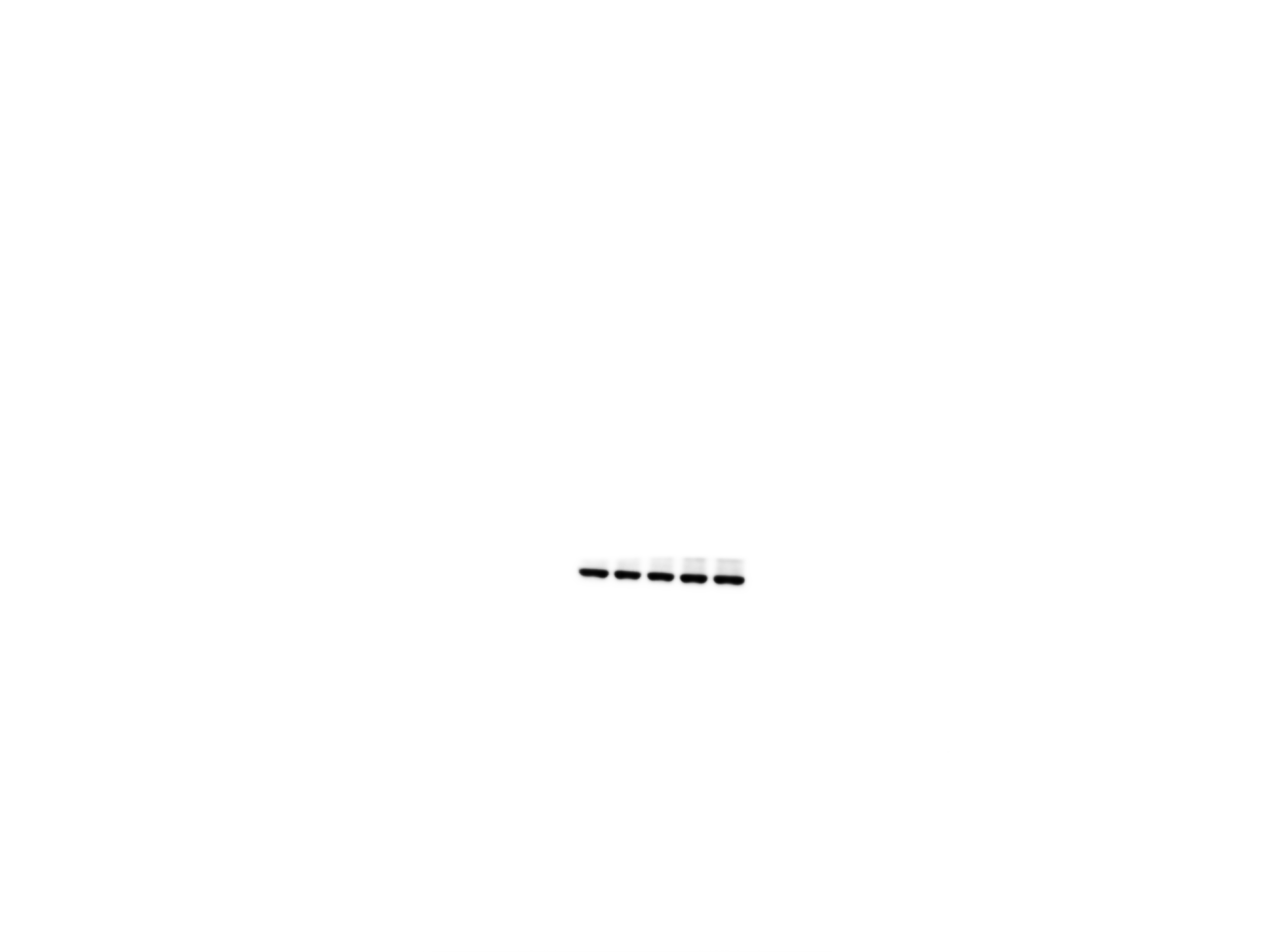

Supplement: S2 File — Original picture of the western blot experiments in the manuscript. (ZIP) [file pone.0274620.s002.zip › S2. blot results/Fig 3/GAPDH/1control/1.tif]

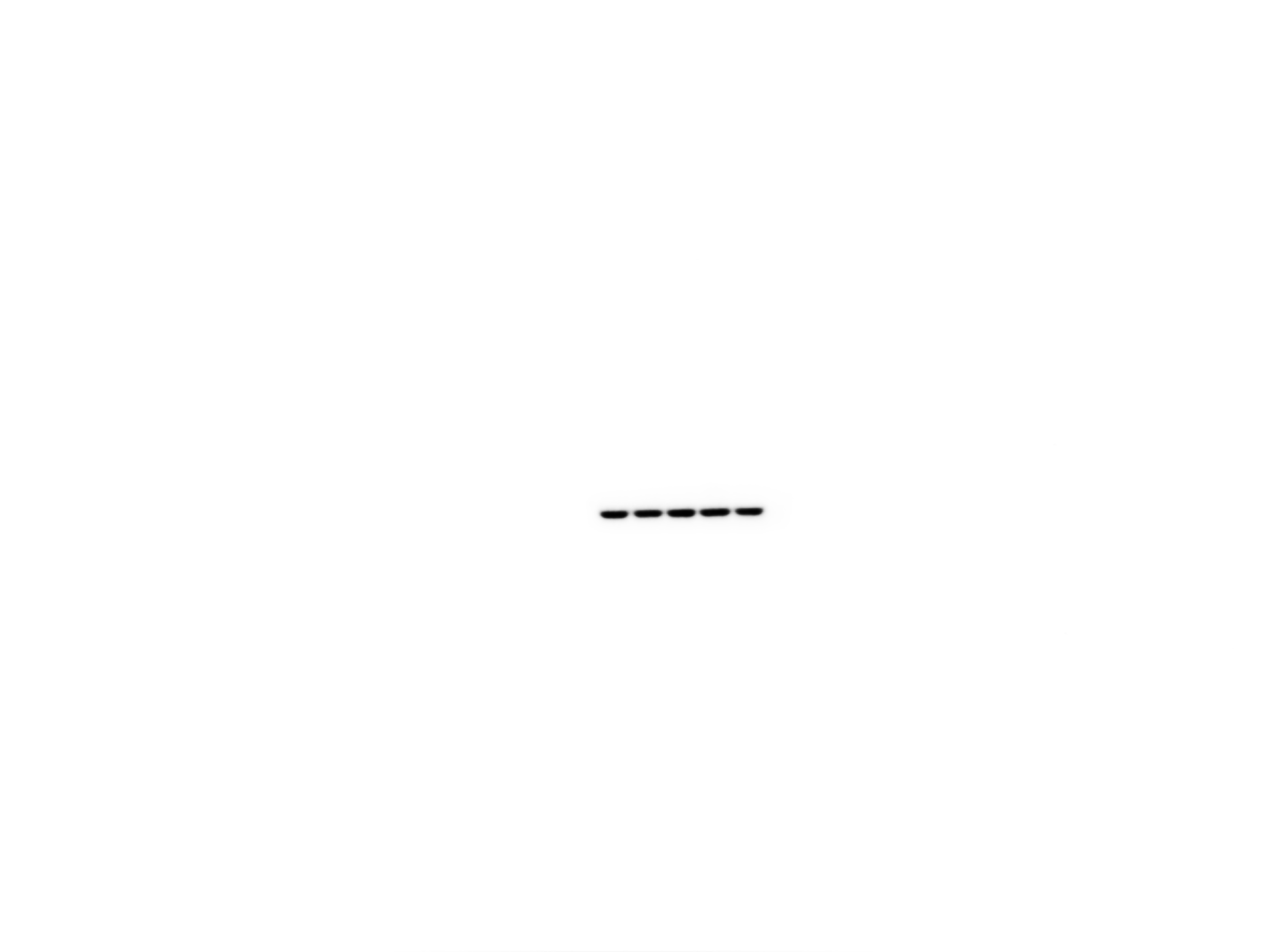

Supplement: S2 File — Original picture of the western blot experiments in the manuscript. (ZIP) [file pone.0274620.s002.zip › S2. blot results/Fig 3/GAPDH/1control/2.tif]

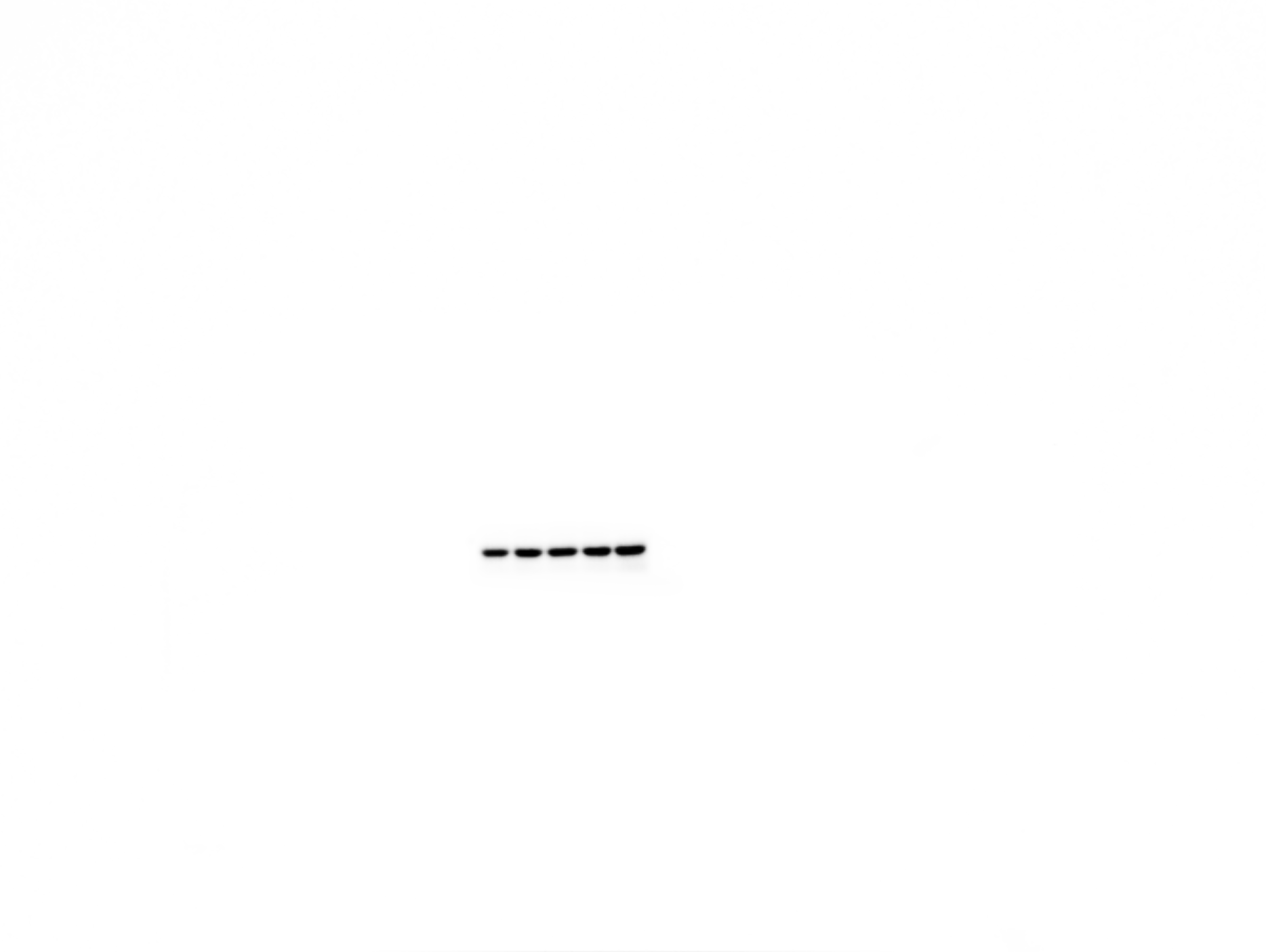

Supplement: S2 File — Original picture of the western blot experiments in the manuscript. (ZIP) [file pone.0274620.s002.zip › S2. blot results/Fig 3/GAPDH/1control/3.tif]

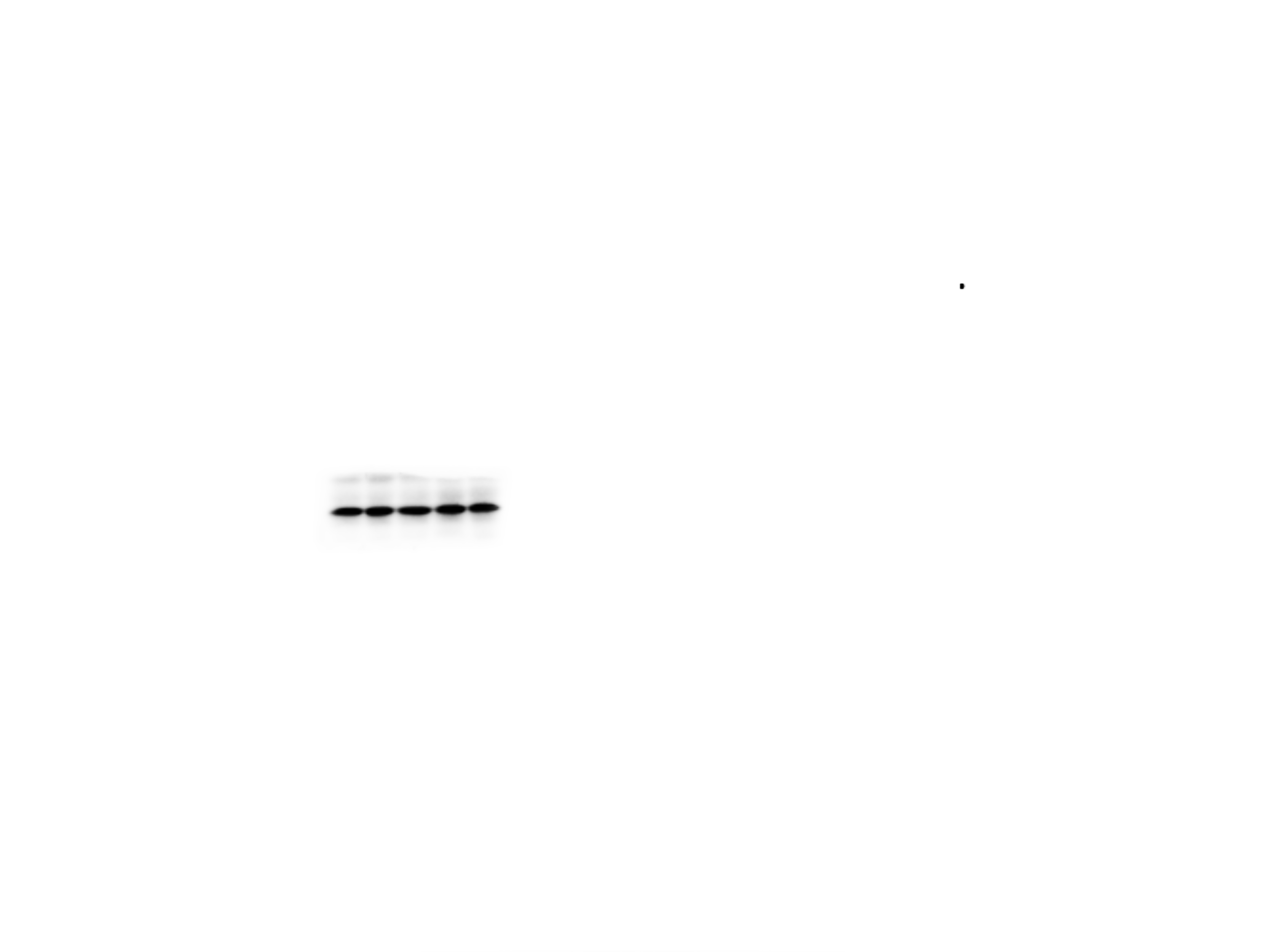

Supplement: S2 File — Original picture of the western blot experiments in the manuscript. (ZIP) [file pone.0274620.s002.zip › S2. blot results/Fig 3/GAPDH/1control/4.tif]

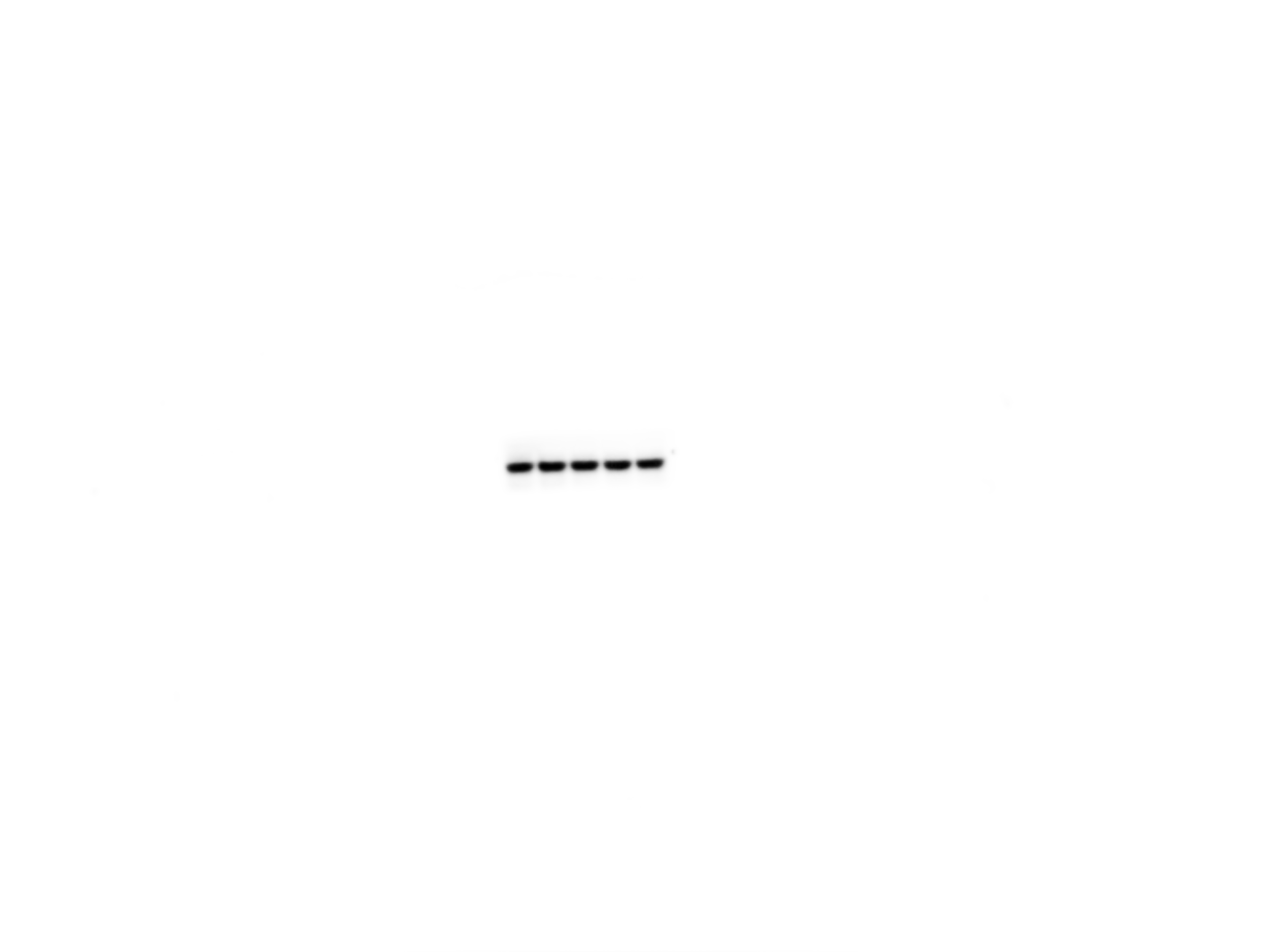

Supplement: S2 File — Original picture of the western blot experiments in the manuscript. (ZIP) [file pone.0274620.s002.zip › S2. blot results/Fig 3/GAPDH/1control/5.tif]

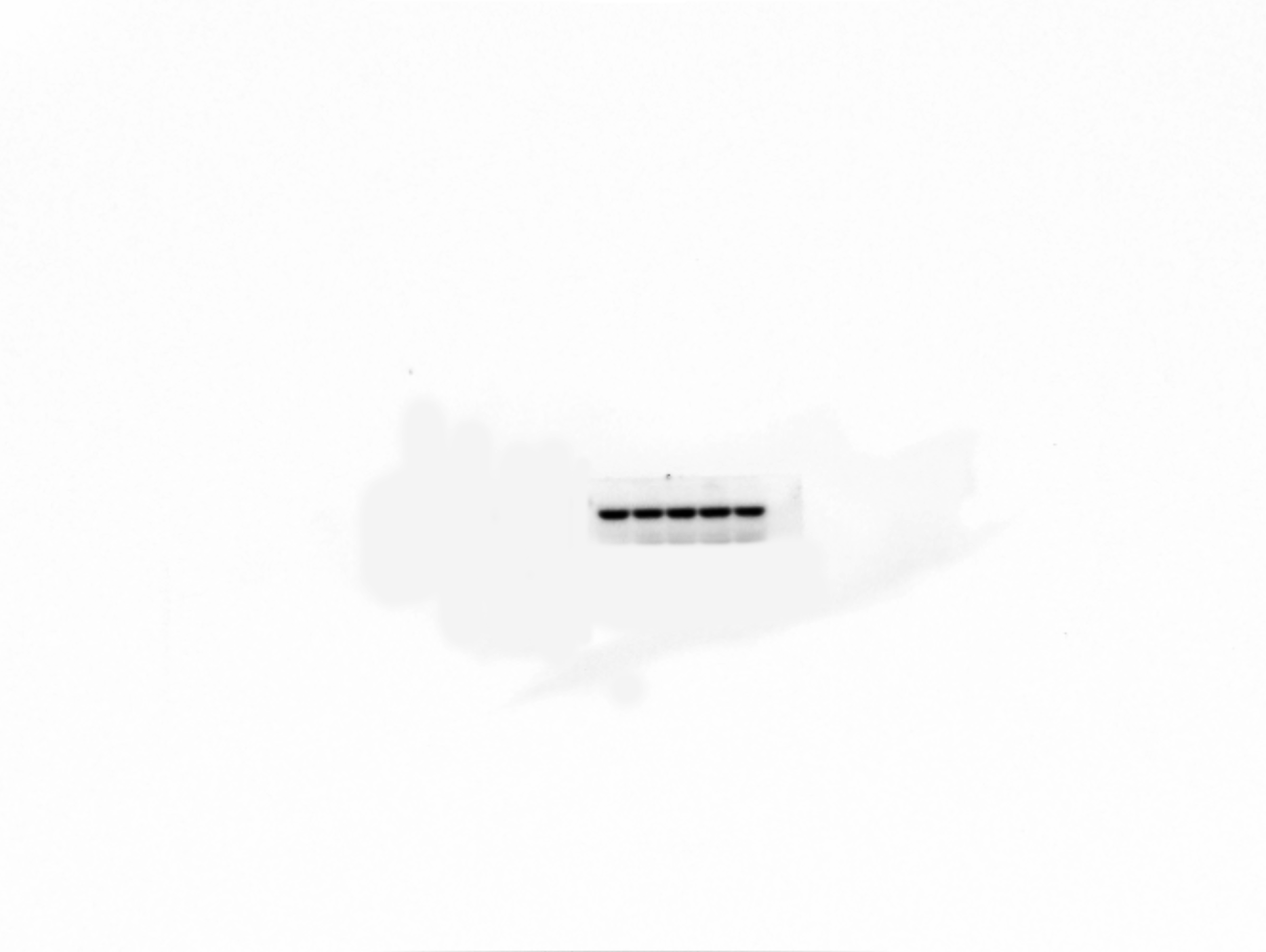

Supplement: S2 File — Original picture of the western blot experiments in the manuscript. (ZIP) [file pone.0274620.s002.zip › S2. blot results/Fig 3/GAPDH/2sham/1.tif]

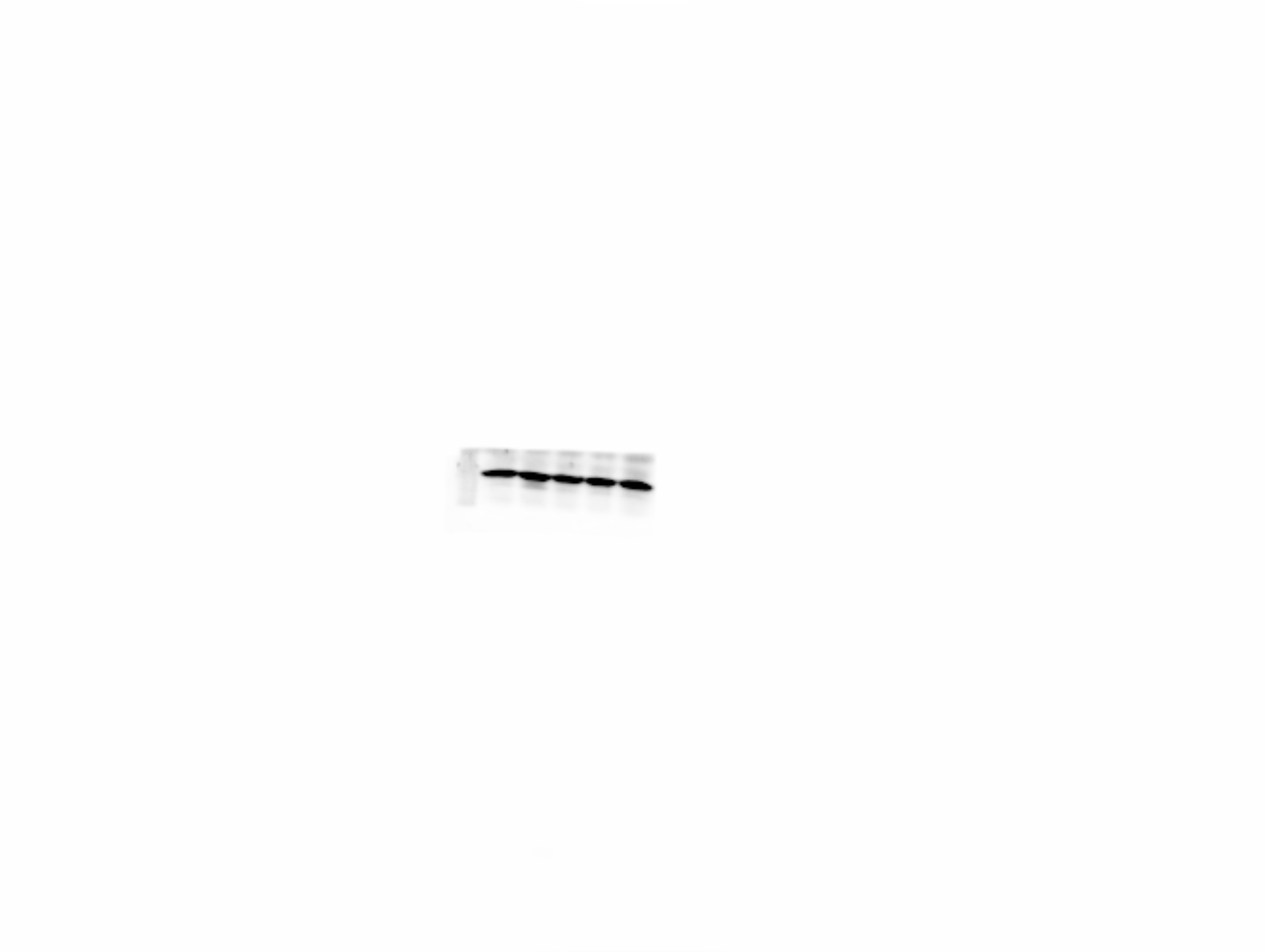

Supplement: S2 File — Original picture of the western blot experiments in the manuscript. (ZIP) [file pone.0274620.s002.zip › S2. blot results/Fig 3/GAPDH/2sham/2.tif]

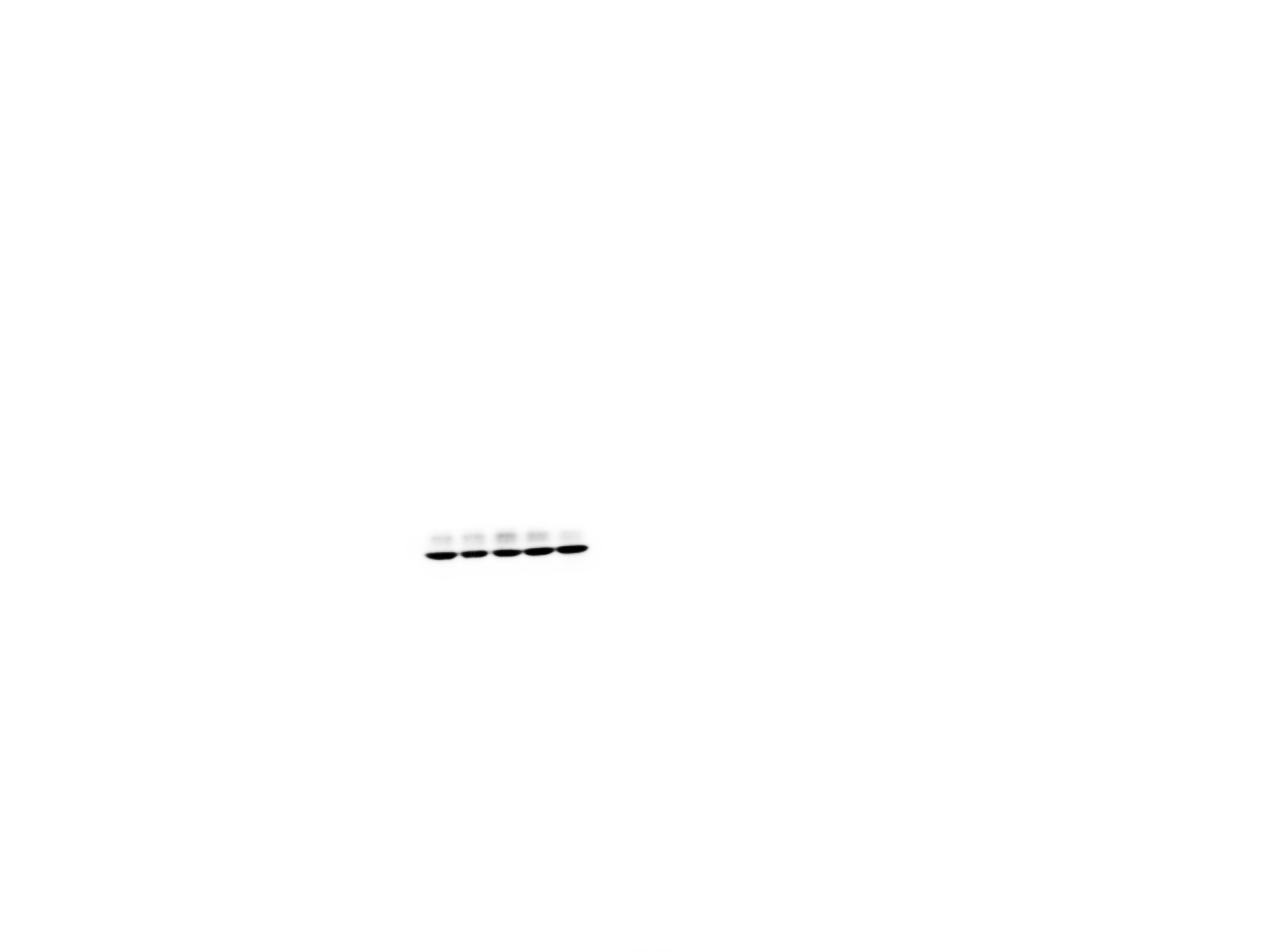

Supplement: S2 File — Original picture of the western blot experiments in the manuscript. (ZIP) [file pone.0274620.s002.zip › S2. blot results/Fig 3/GAPDH/2sham/3.tif]

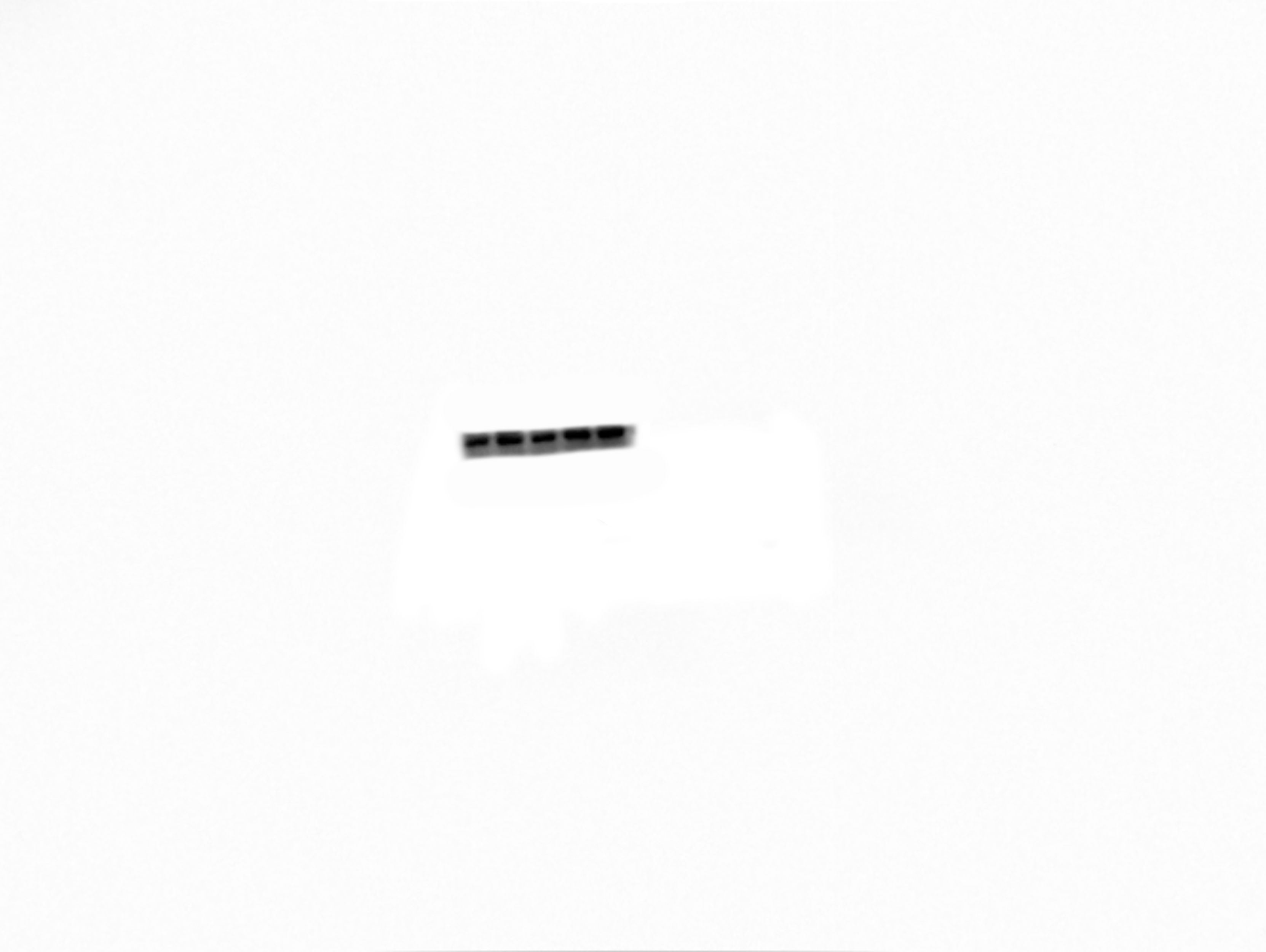

Supplement: S2 File — Original picture of the western blot experiments in the manuscript. (ZIP) [file pone.0274620.s002.zip › S2. blot results/Fig 3/GAPDH/2sham/4.tif]

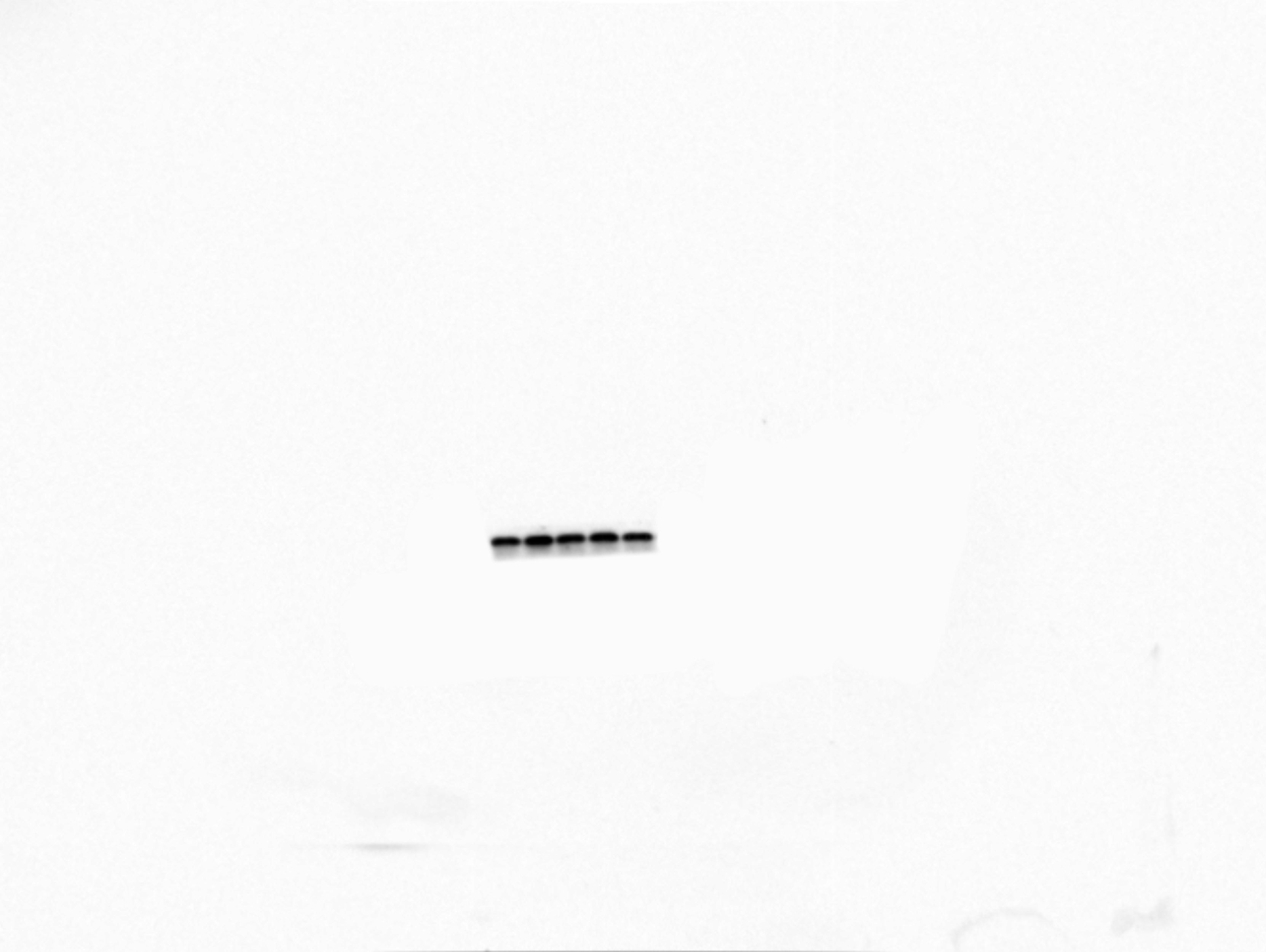

Supplement: S2 File — Original picture of the western blot experiments in the manuscript. (ZIP) [file pone.0274620.s002.zip › S2. blot results/Fig 3/GAPDH/2sham/5.tif]

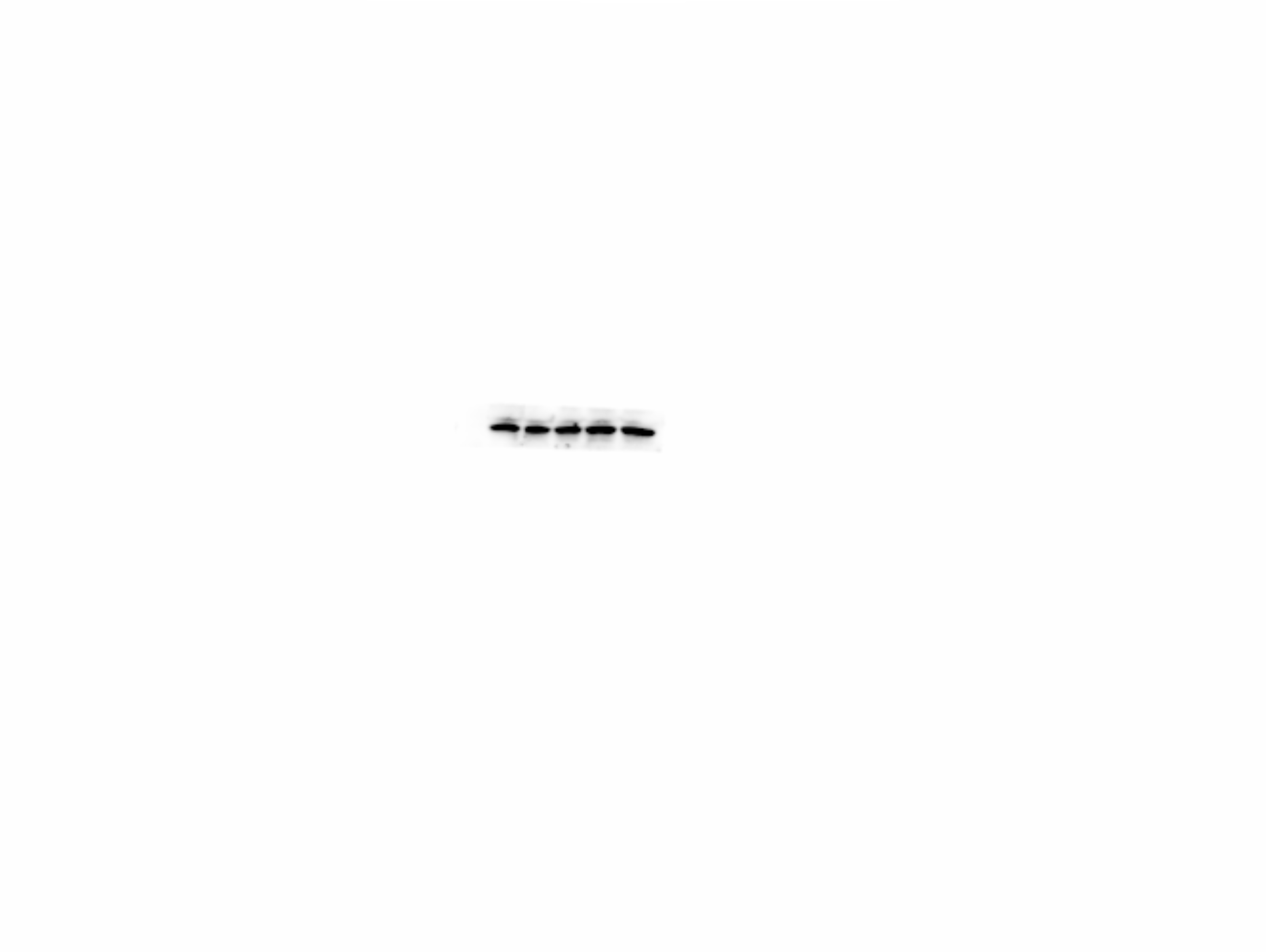

Supplement: S2 File — Original picture of the western blot experiments in the manuscript. (ZIP) [file pone.0274620.s002.zip › S2. blot results/Fig 3/GAPDH/3model/1.tif]

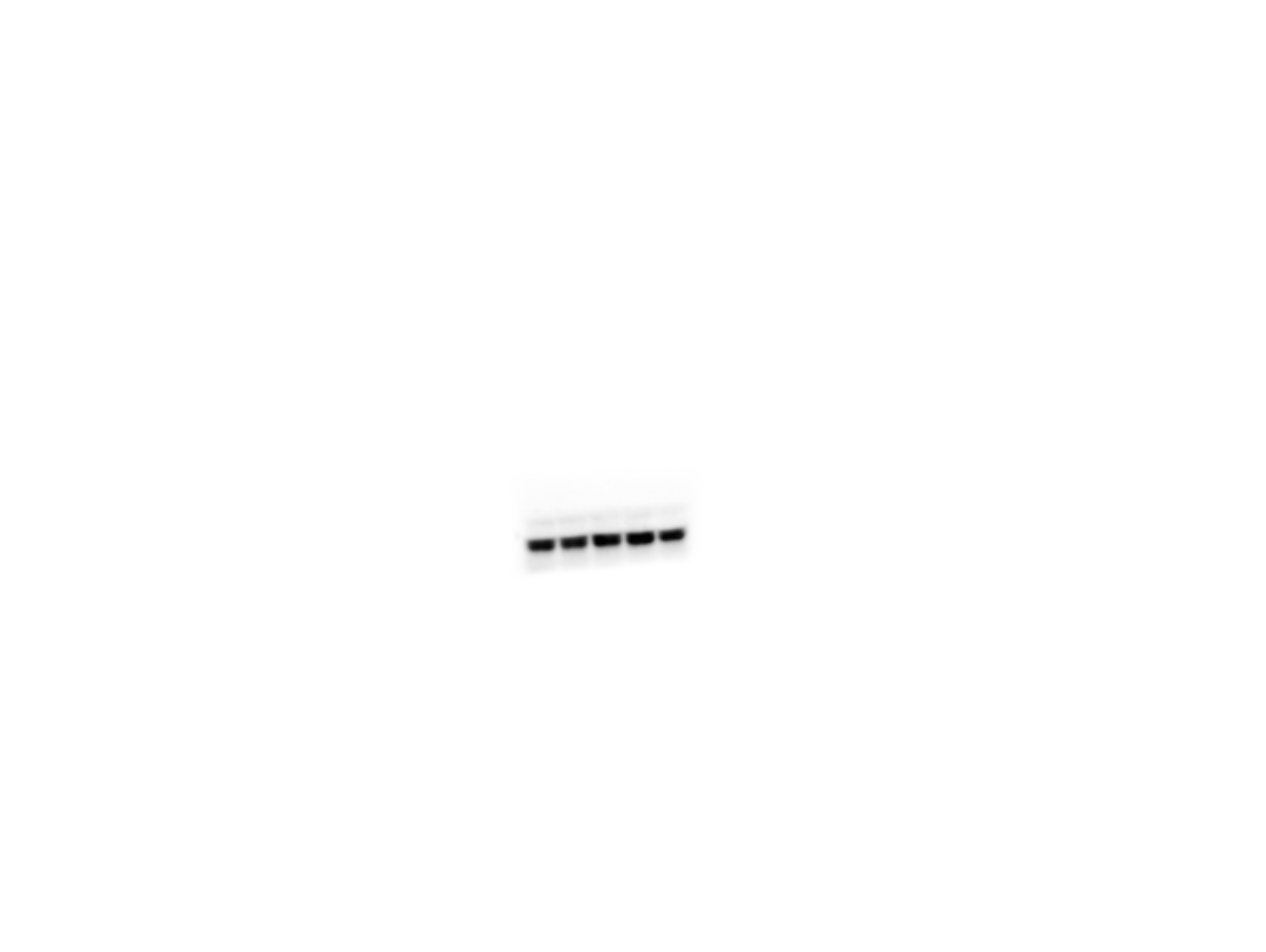

Supplement: S2 File — Original picture of the western blot experiments in the manuscript. (ZIP) [file pone.0274620.s002.zip › S2. blot results/Fig 3/GAPDH/3model/2.tif]

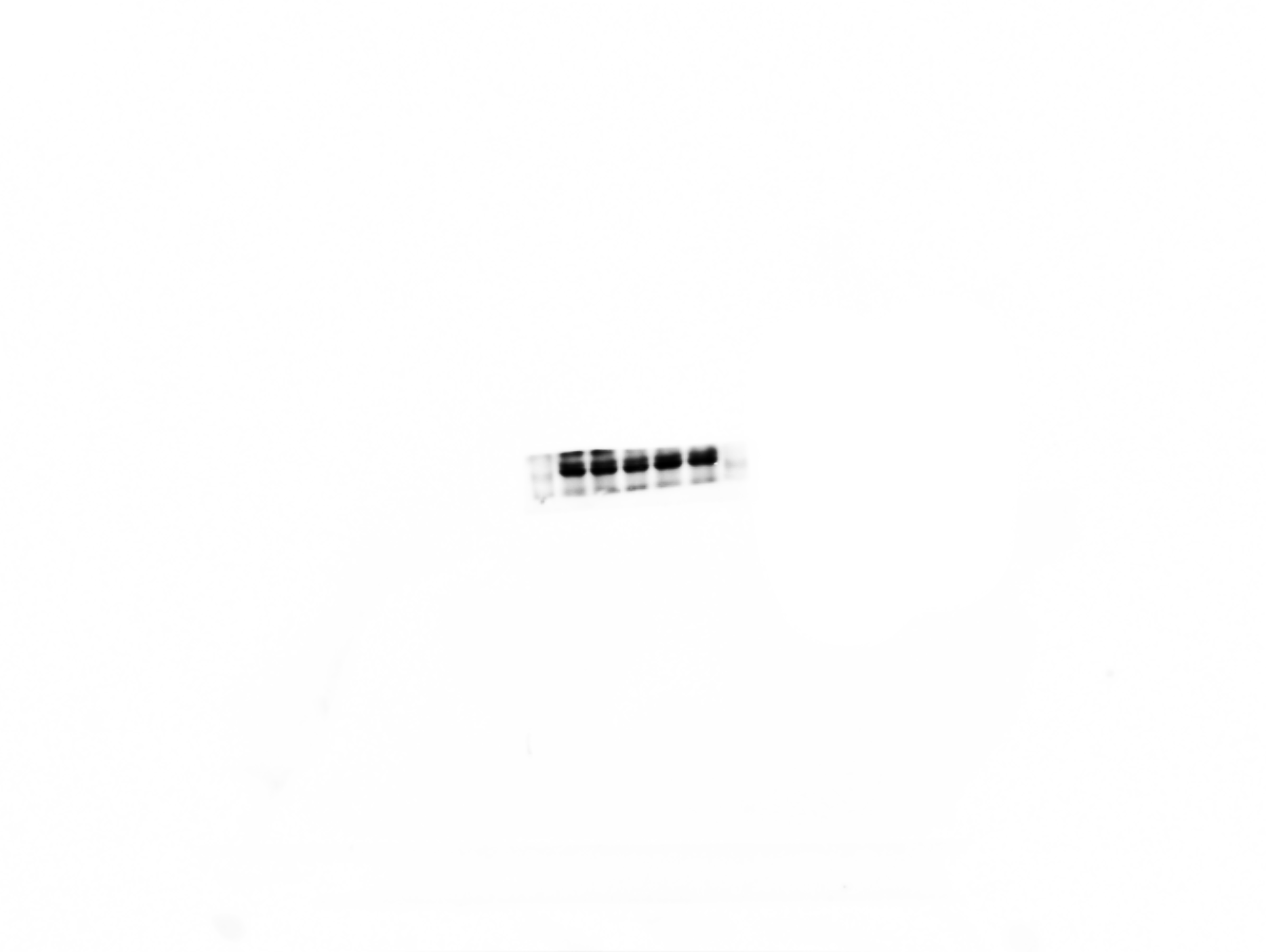

Supplement: S2 File — Original picture of the western blot experiments in the manuscript. (ZIP) [file pone.0274620.s002.zip › S2. blot results/Fig 3/GAPDH/3model/3.tif]

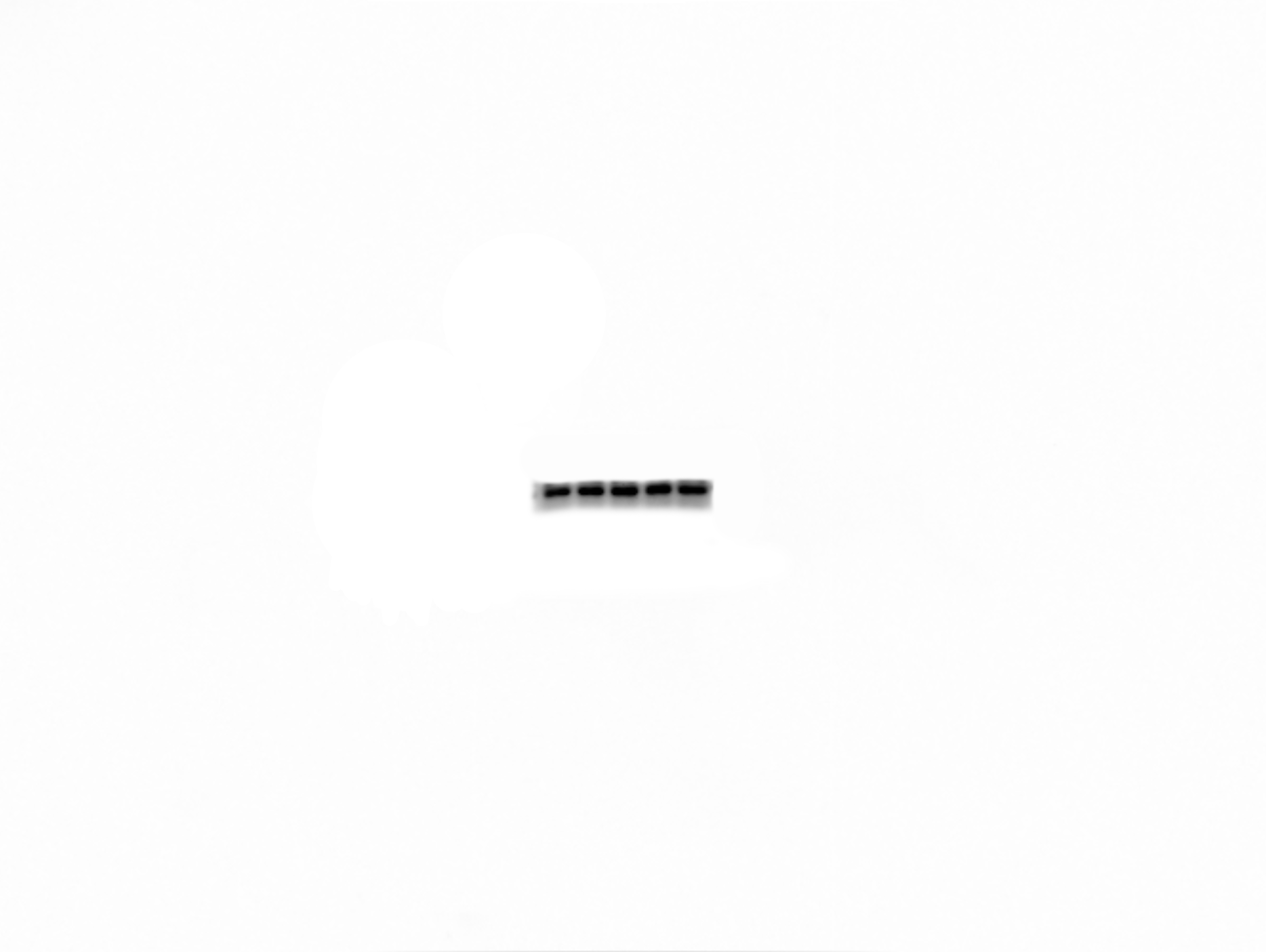

Supplement: S2 File — Original picture of the western blot experiments in the manuscript. (ZIP) [file pone.0274620.s002.zip › S2. blot results/Fig 3/GAPDH/3model/4.tif]

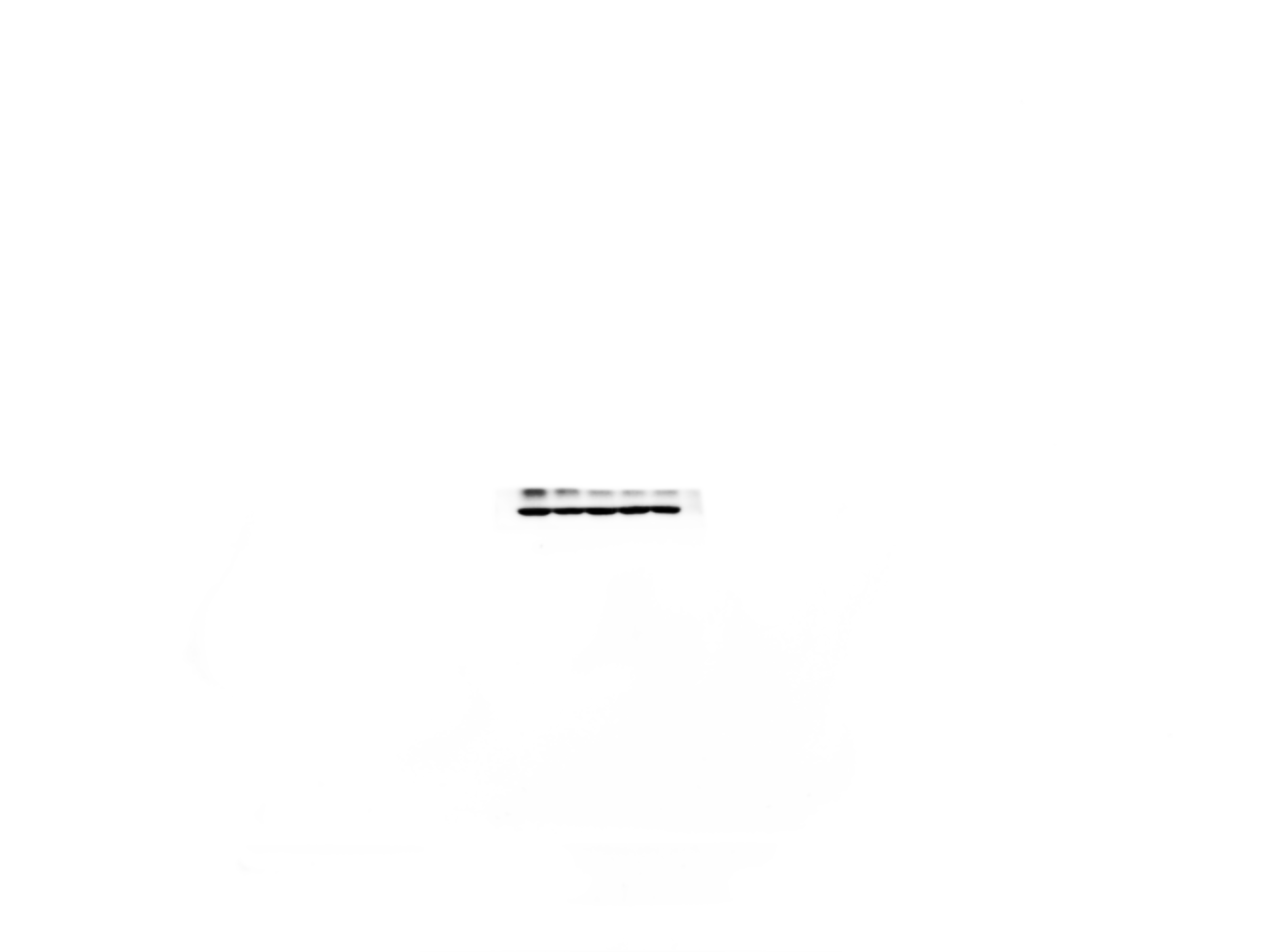

Supplement: S2 File — Original picture of the western blot experiments in the manuscript. (ZIP) [file pone.0274620.s002.zip › S2. blot results/Fig 3/GAPDH/3model/5.tif]

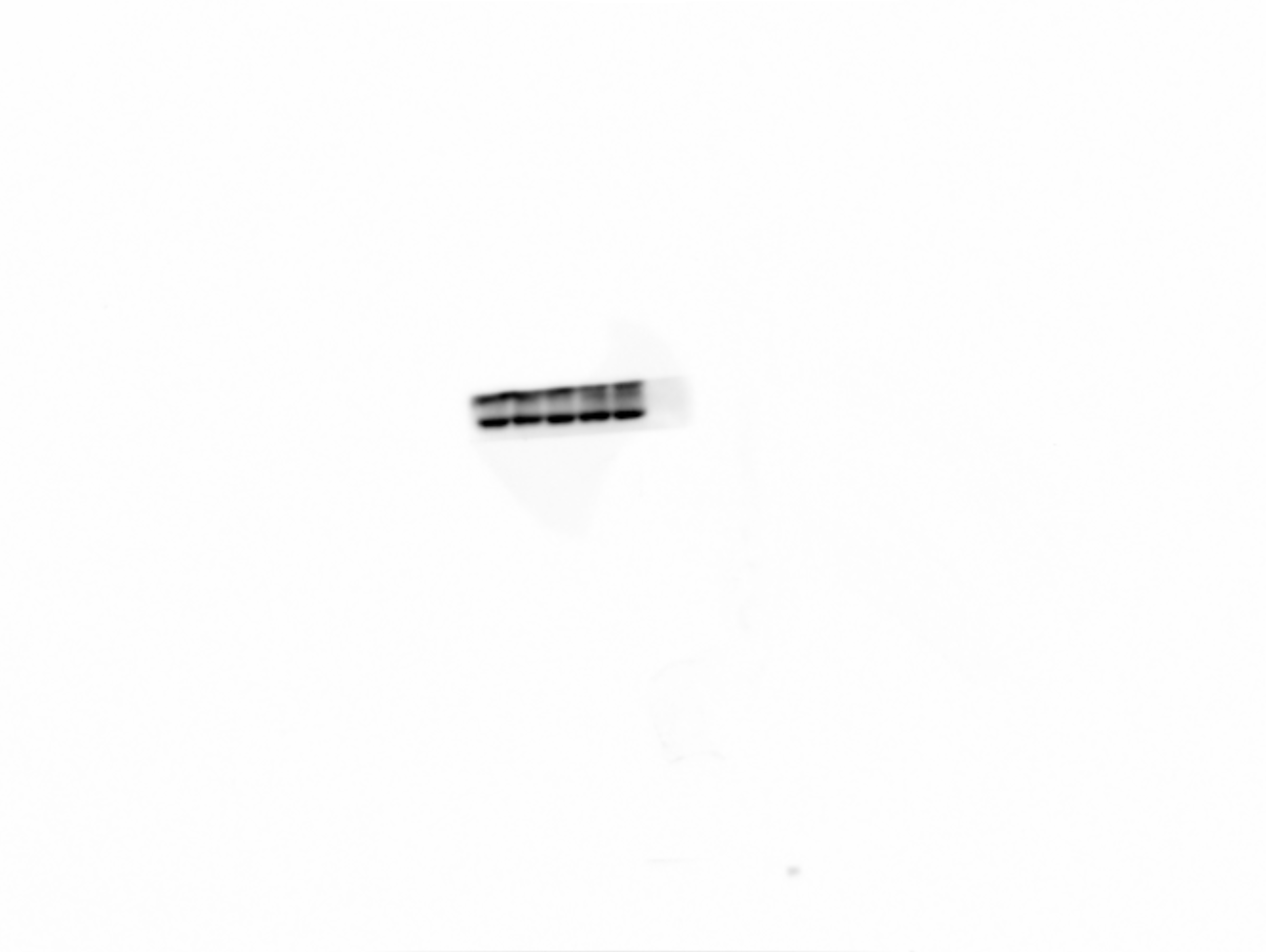

Supplement: S2 File — Original picture of the western blot experiments in the manuscript. (ZIP) [file pone.0274620.s002.zip › S2. blot results/Fig 3/GAPDH/4EA/1.tif]

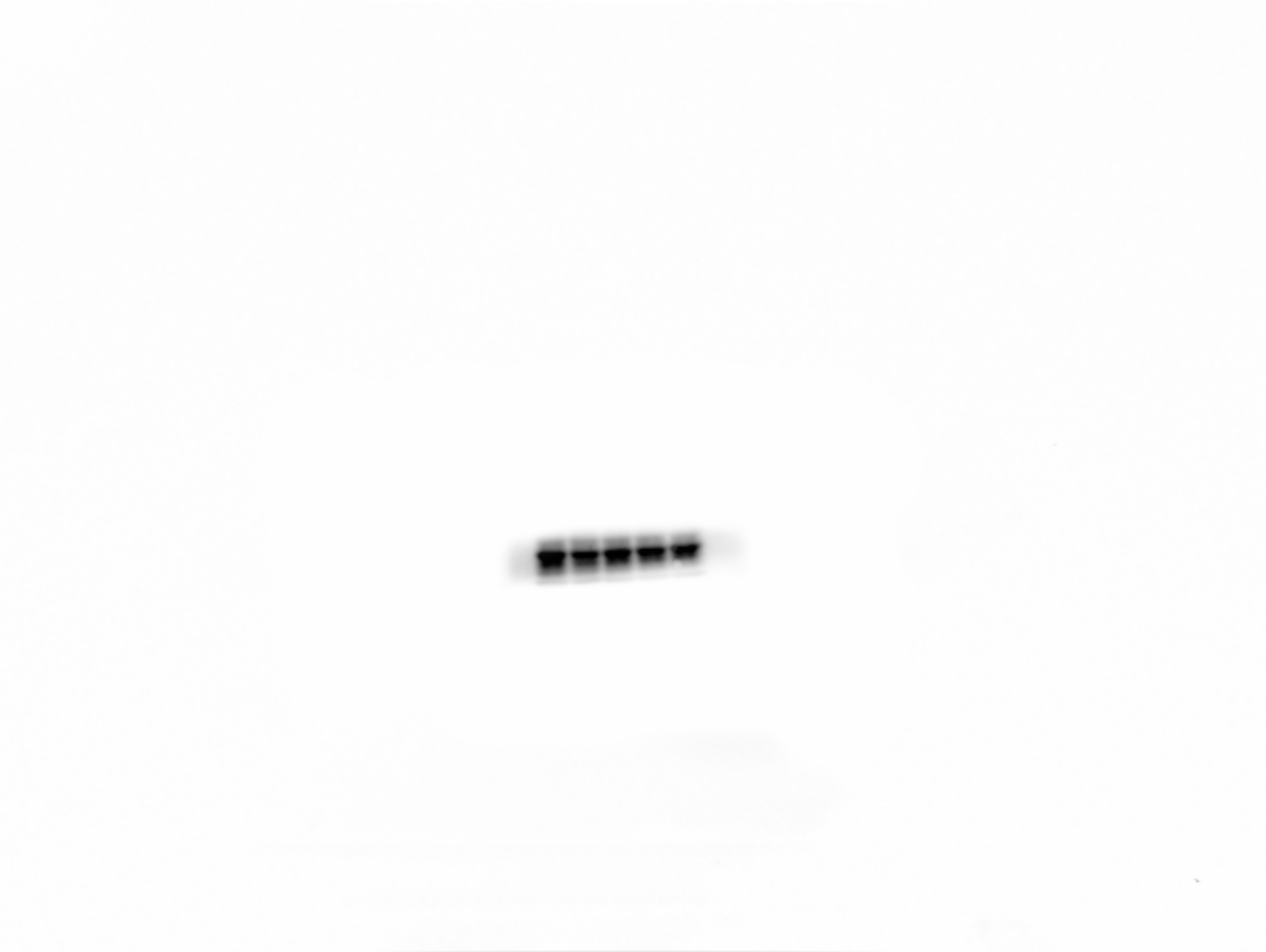

Supplement: S2 File — Original picture of the western blot experiments in the manuscript. (ZIP) [file pone.0274620.s002.zip › S2. blot results/Fig 3/GAPDH/4EA/2.tif]

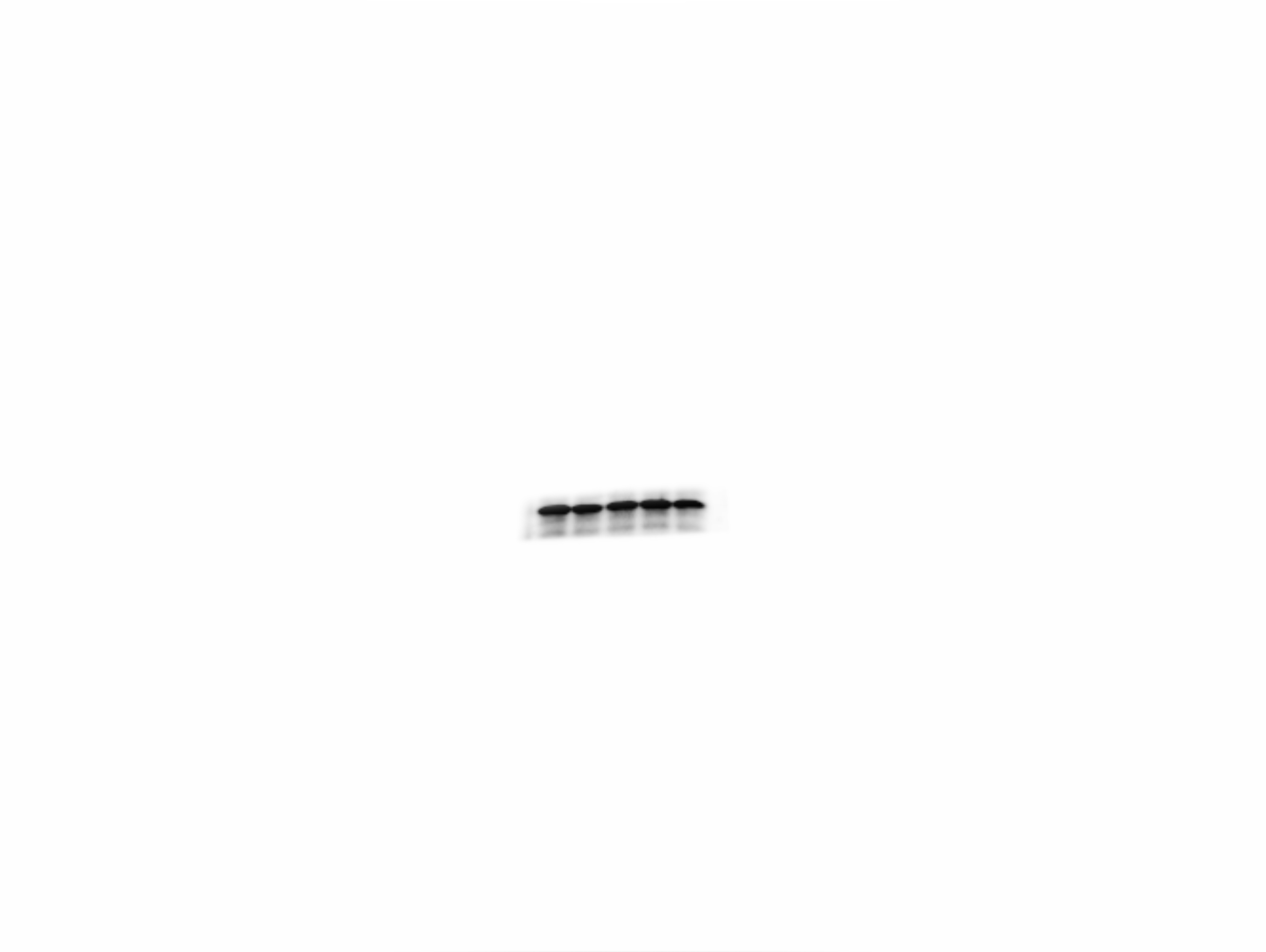

Supplement: S2 File — Original picture of the western blot experiments in the manuscript. (ZIP) [file pone.0274620.s002.zip › S2. blot results/Fig 3/GAPDH/4EA/3.tif]

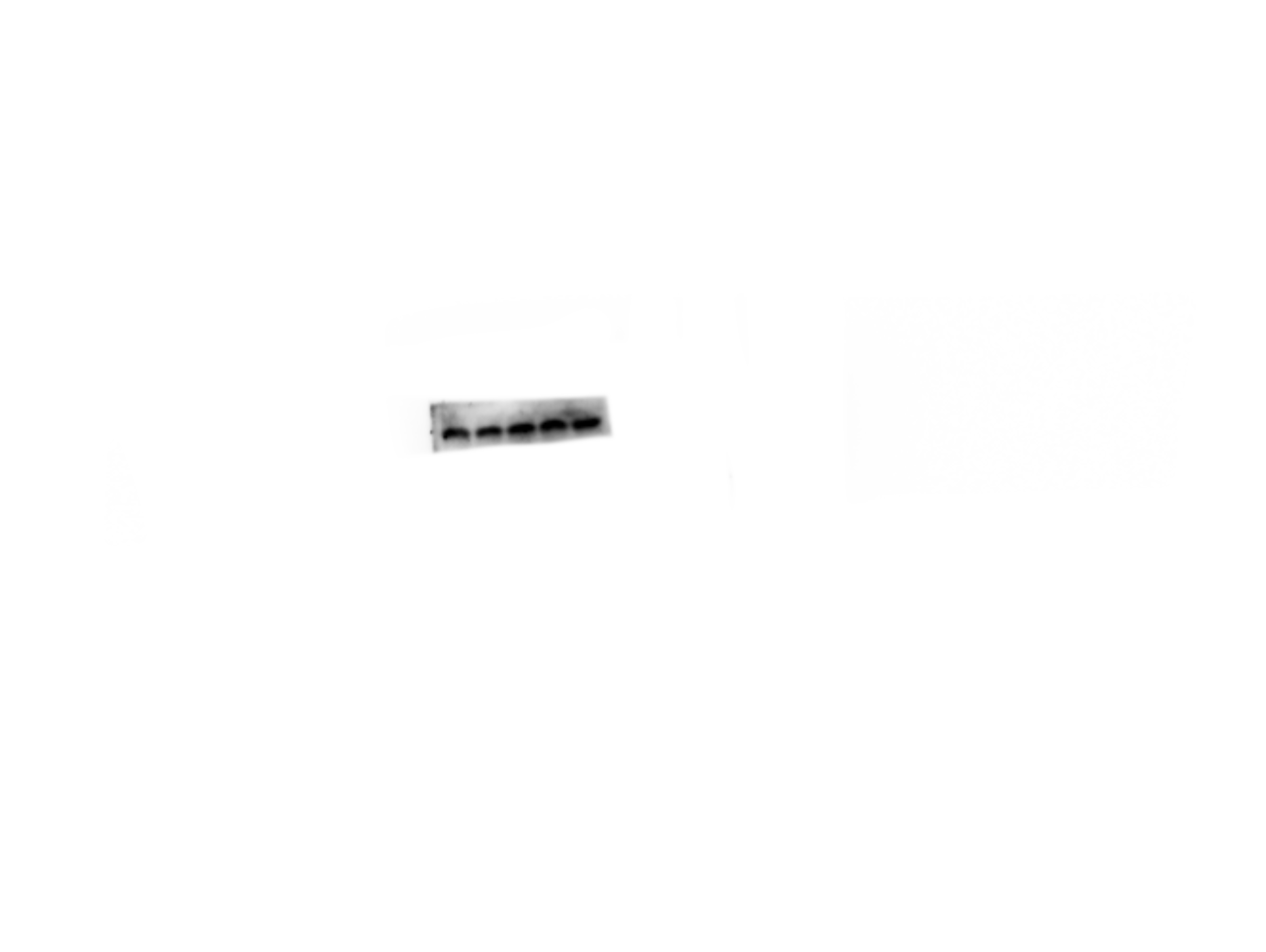

Supplement: S2 File — Original picture of the western blot experiments in the manuscript. (ZIP) [file pone.0274620.s002.zip › S2. blot results/Fig 3/GAPDH/4EA/4.tif]

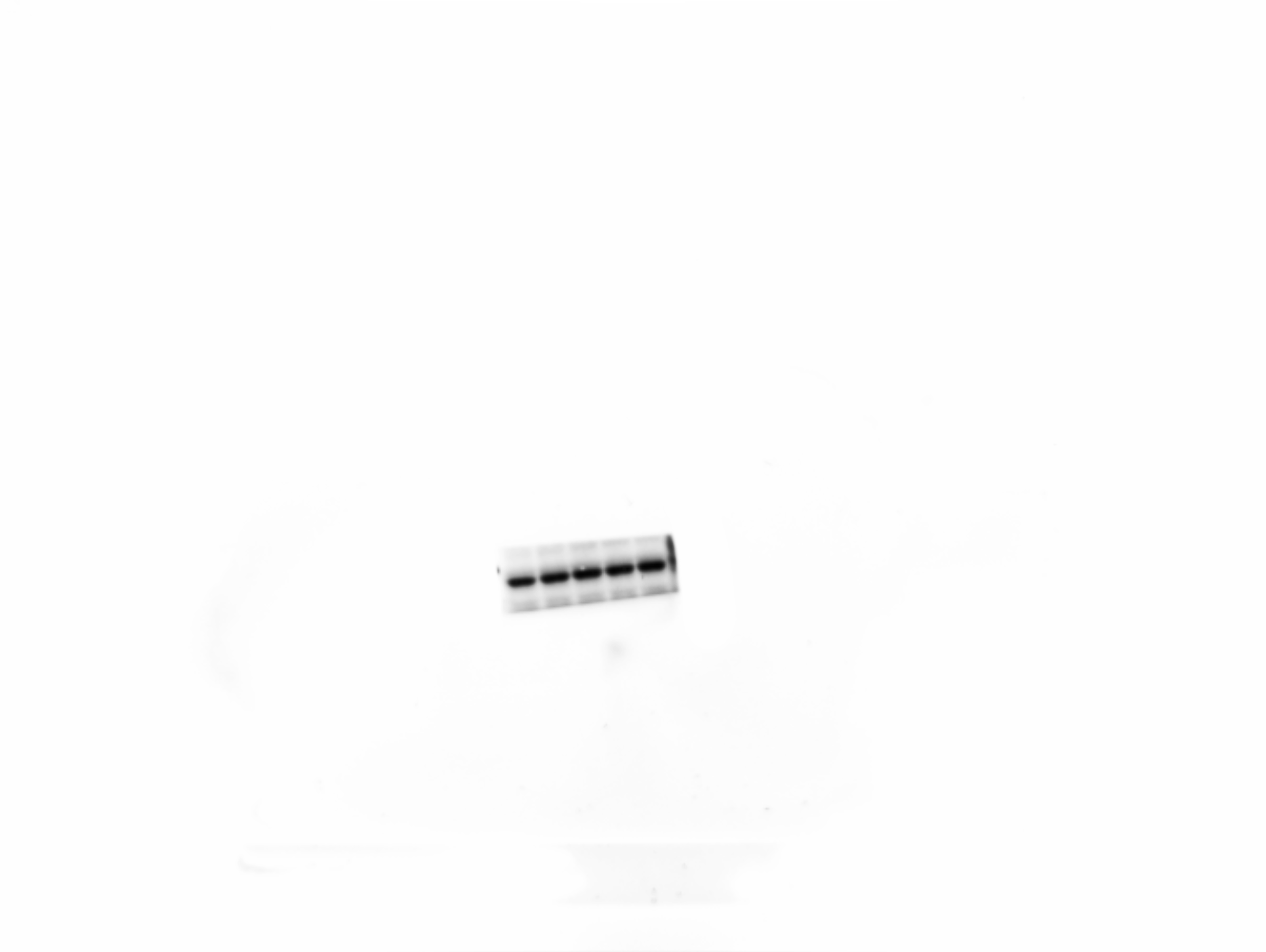

Supplement: S2 File — Original picture of the western blot experiments in the manuscript. (ZIP) [file pone.0274620.s002.zip › S2. blot results/Fig 3/GAPDH/4EA/5.tif]

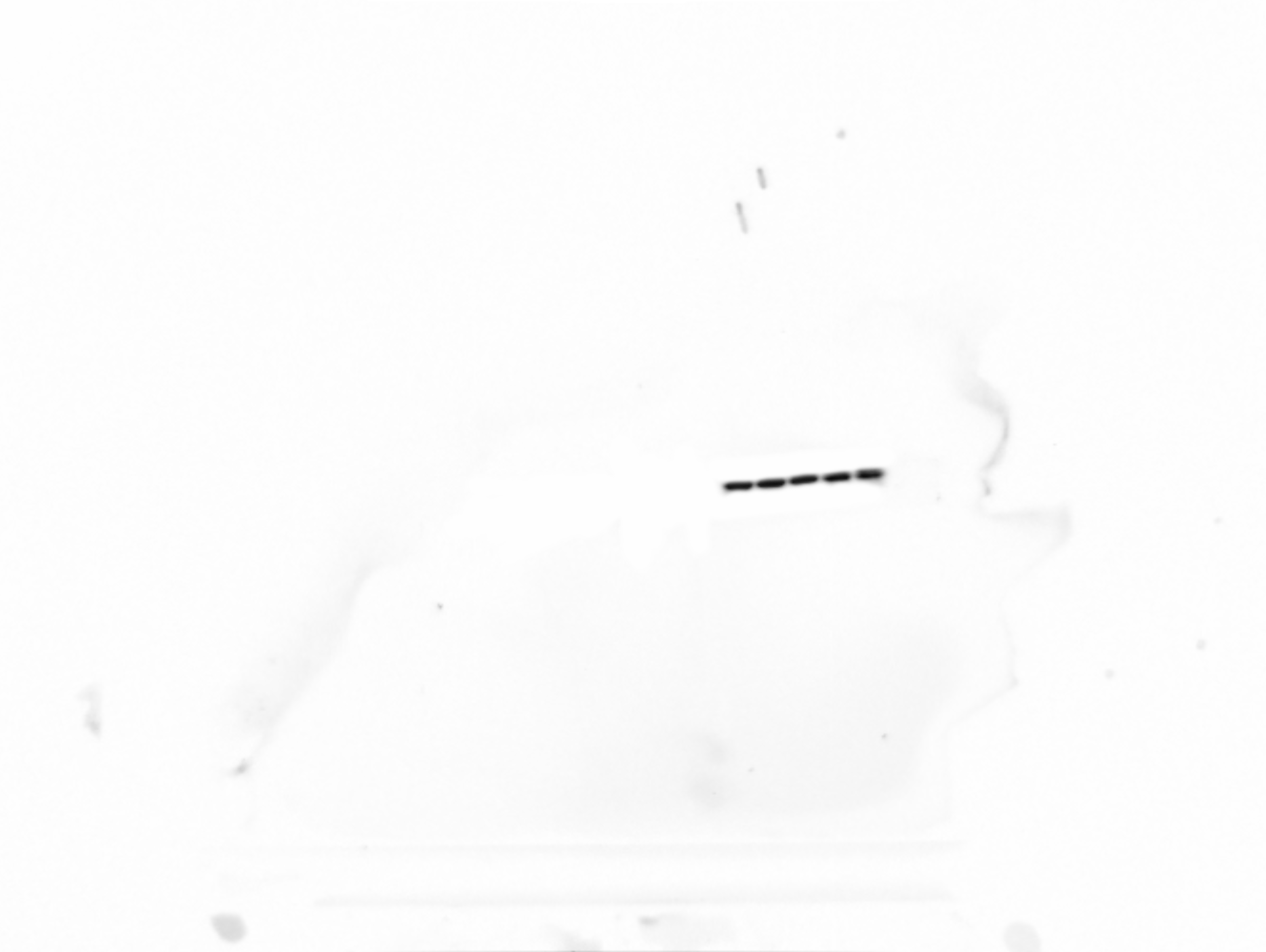

Supplement: S2 File — Original picture of the western blot experiments in the manuscript. (ZIP) [file pone.0274620.s002.zip › S2. blot results/Fig 3/p-Src/1control/1.tif]

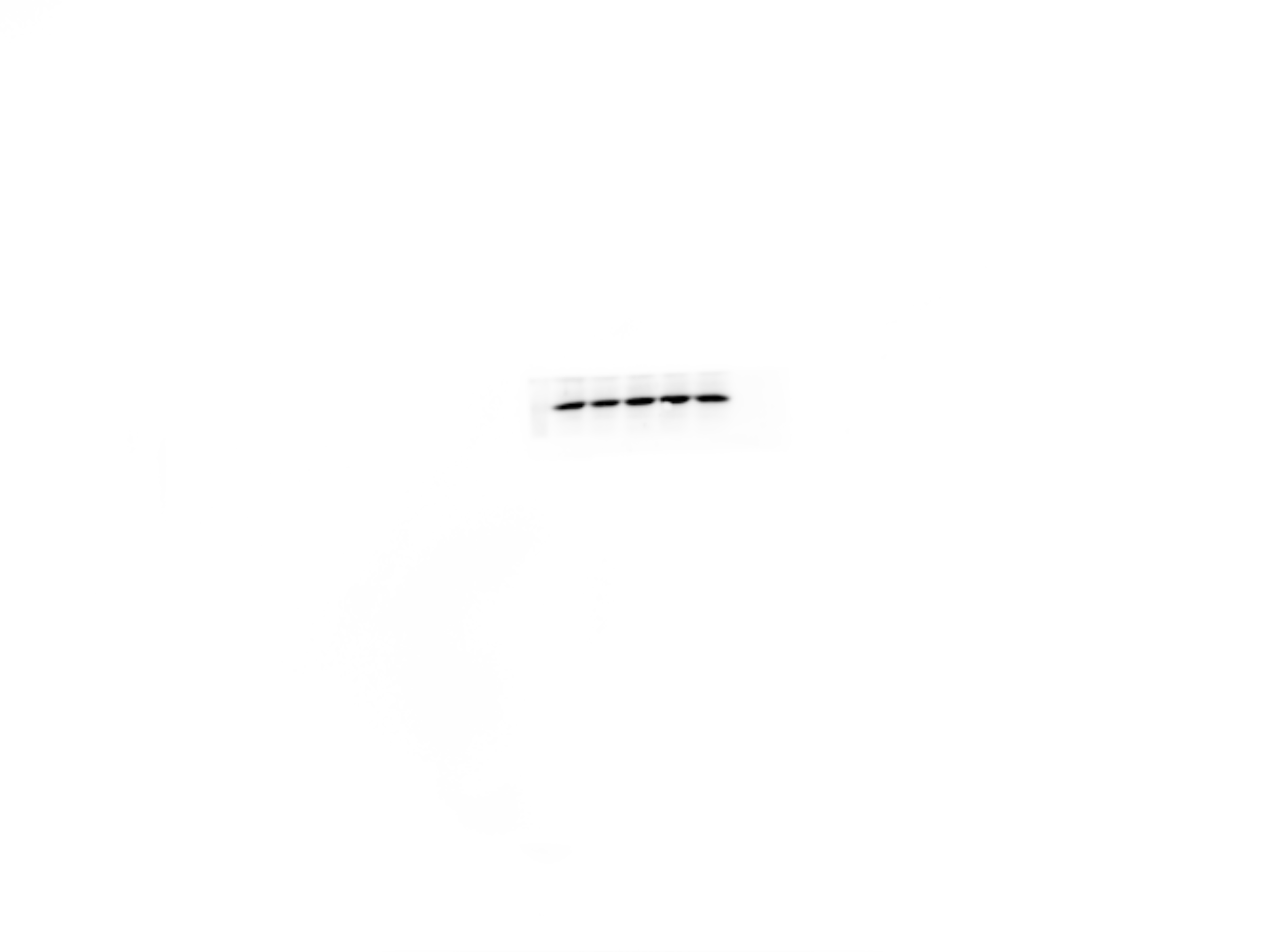

Supplement: S2 File — Original picture of the western blot experiments in the manuscript. (ZIP) [file pone.0274620.s002.zip › S2. blot results/Fig 3/p-Src/1control/2.tif]

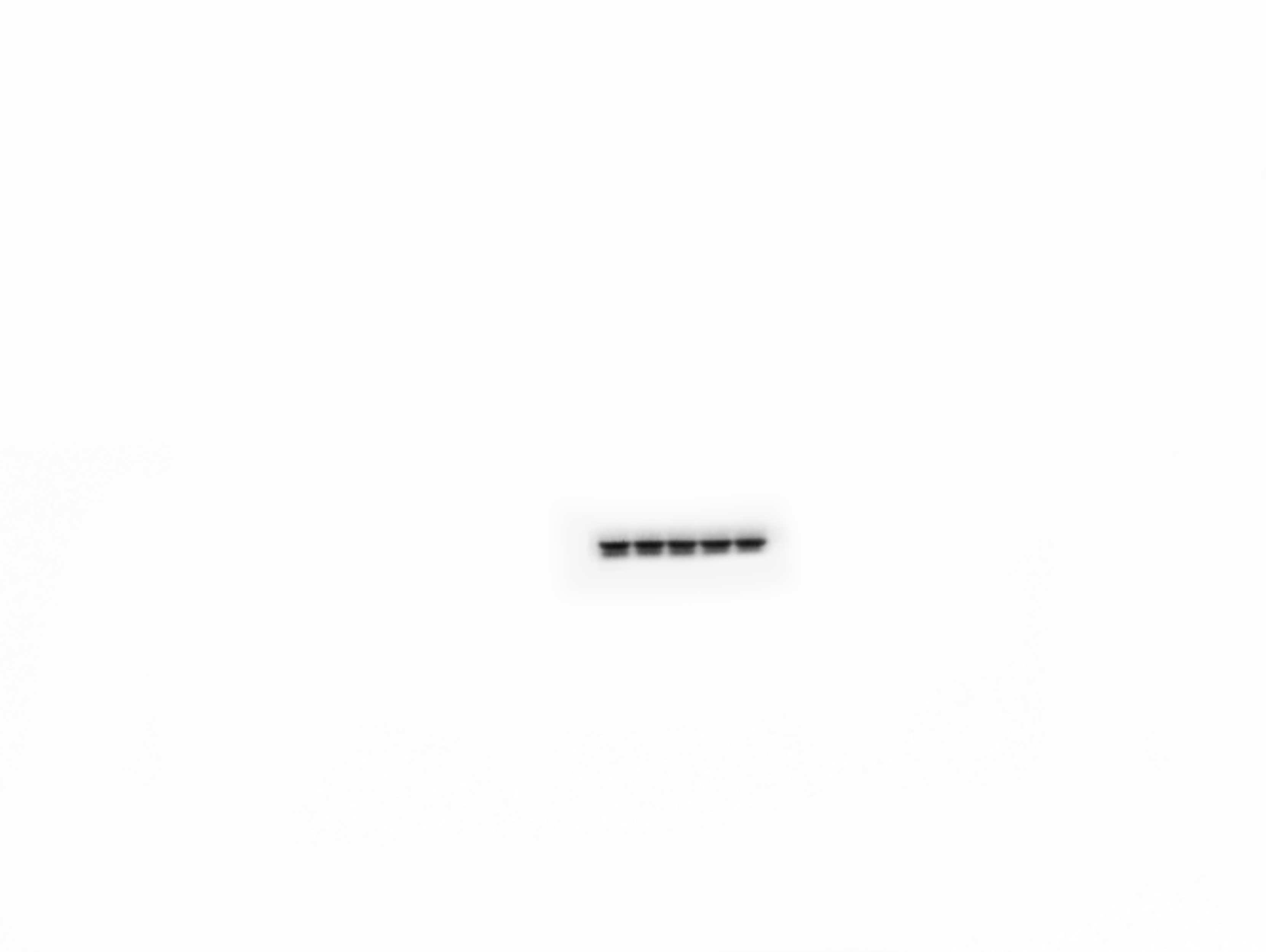

Supplement: S2 File — Original picture of the western blot experiments in the manuscript. (ZIP) [file pone.0274620.s002.zip › S2. blot results/Fig 3/p-Src/1control/3.tif]

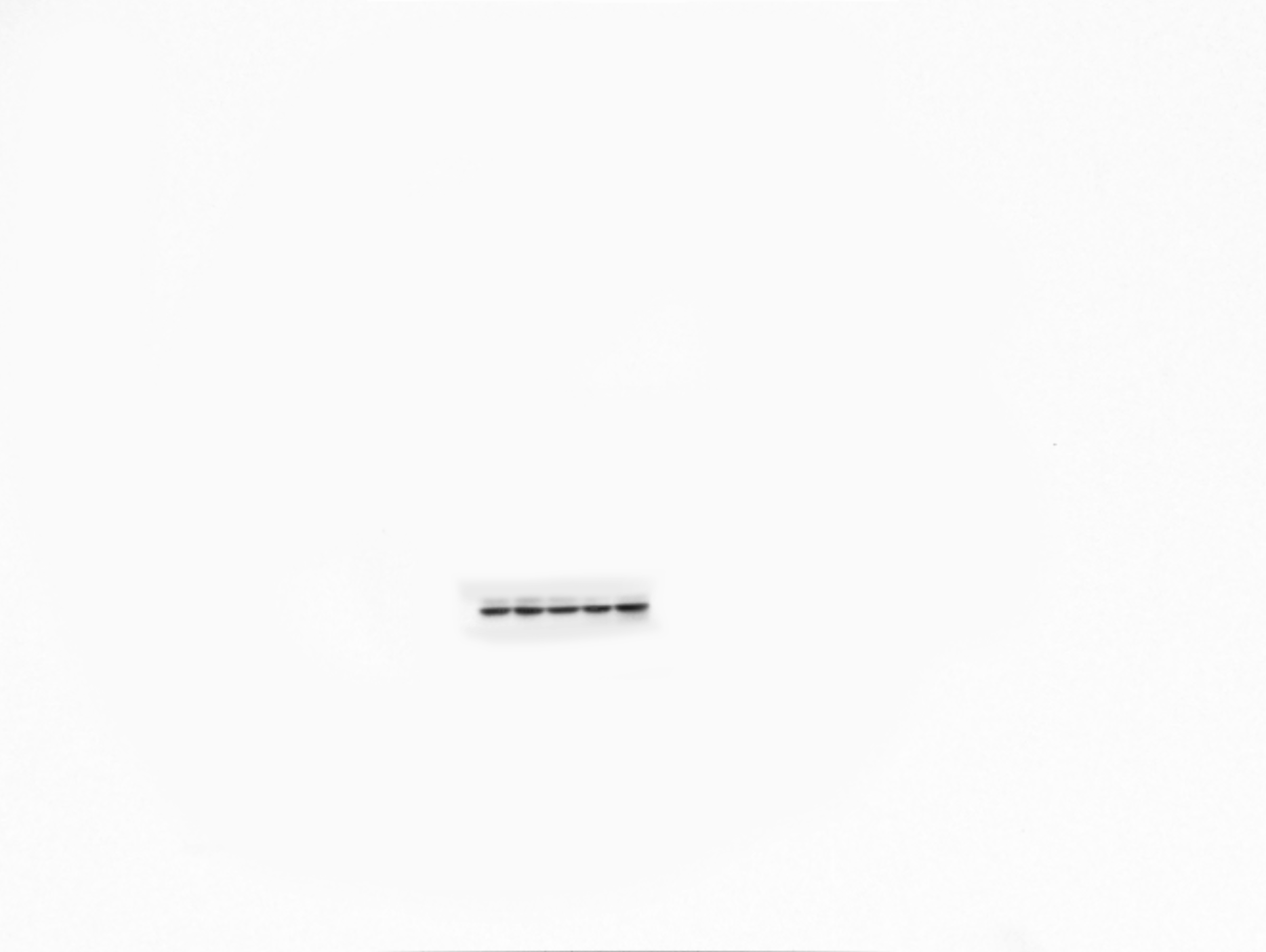

Supplement: S2 File — Original picture of the western blot experiments in the manuscript. (ZIP) [file pone.0274620.s002.zip › S2. blot results/Fig 3/p-Src/1control/4.tif]

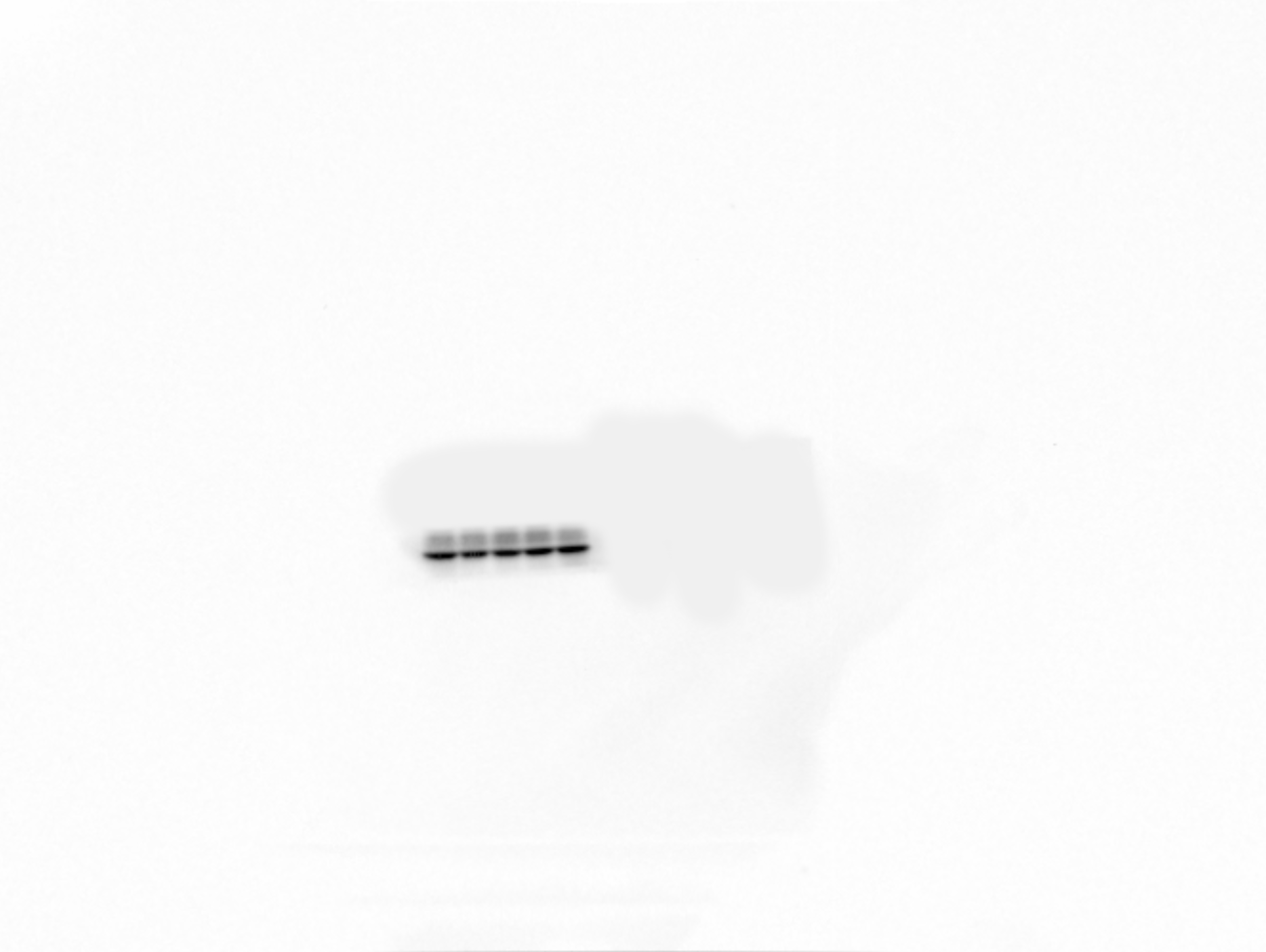

Supplement: S2 File — Original picture of the western blot experiments in the manuscript. (ZIP) [file pone.0274620.s002.zip › S2. blot results/Fig 3/p-Src/1control/5.tif]

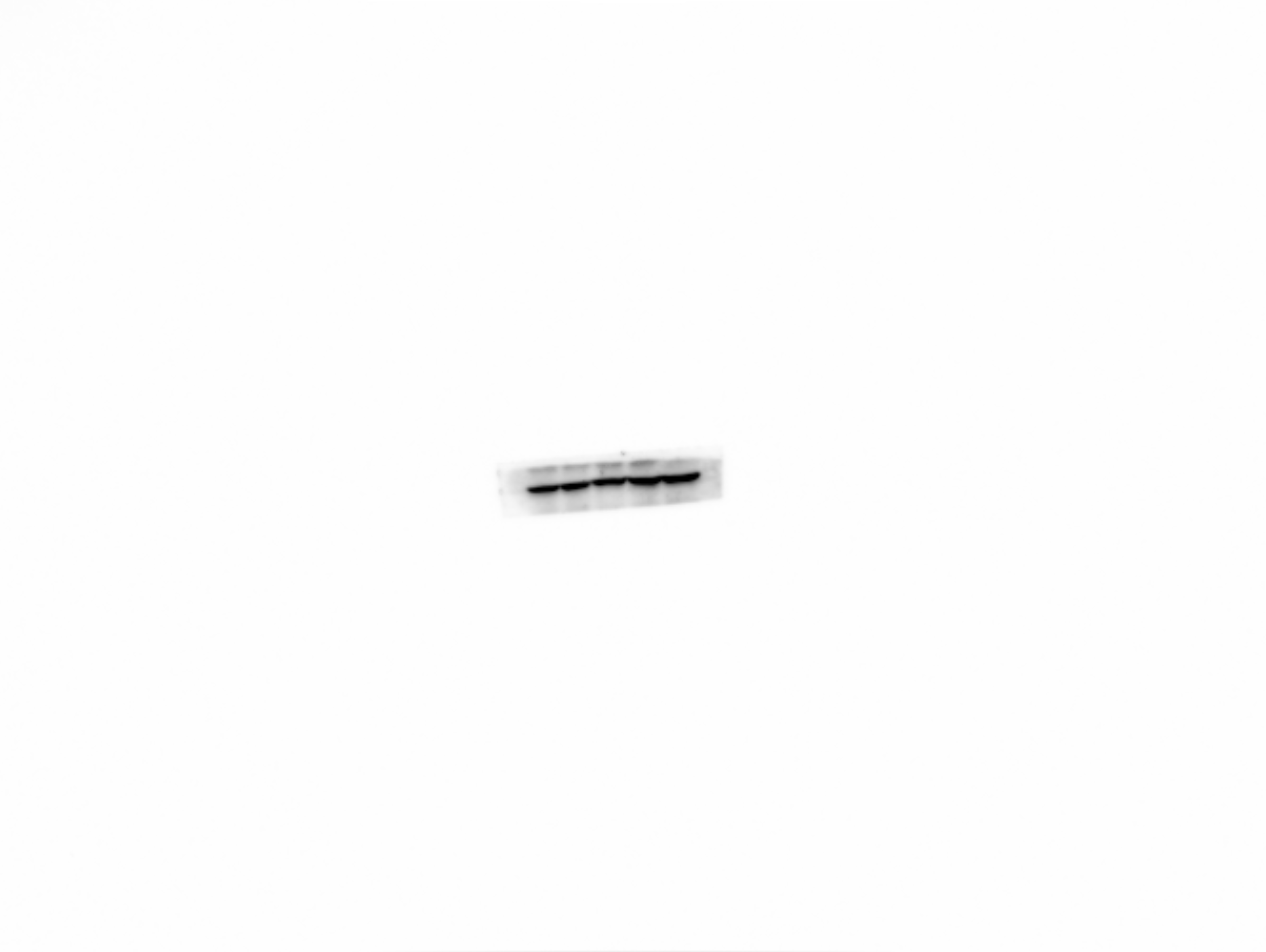

Supplement: S2 File — Original picture of the western blot experiments in the manuscript. (ZIP) [file pone.0274620.s002.zip › S2. blot results/Fig 3/p-Src/2sham/1.tif]

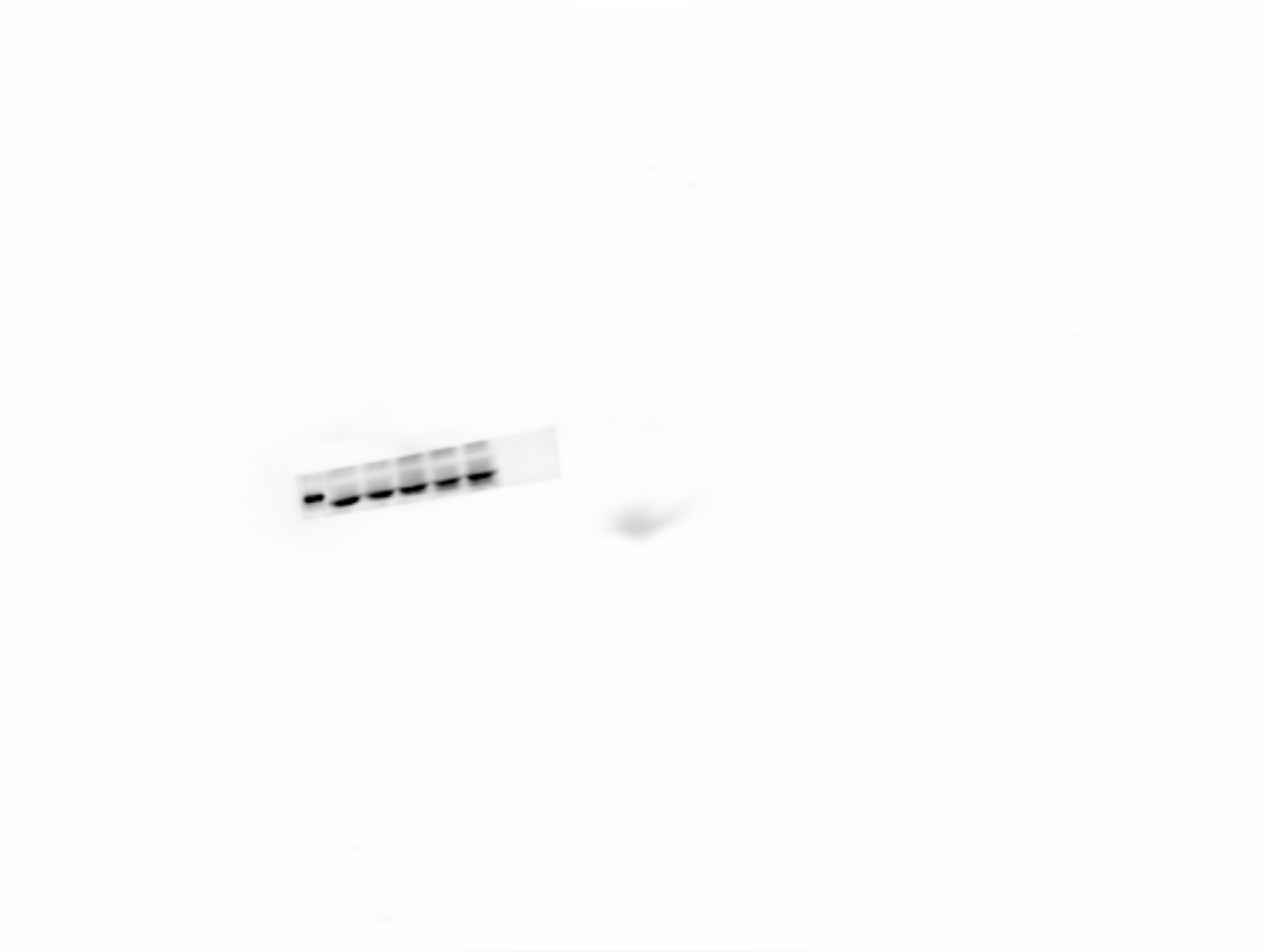

Supplement: S2 File — Original picture of the western blot experiments in the manuscript. (ZIP) [file pone.0274620.s002.zip › S2. blot results/Fig 3/p-Src/2sham/2.tif]

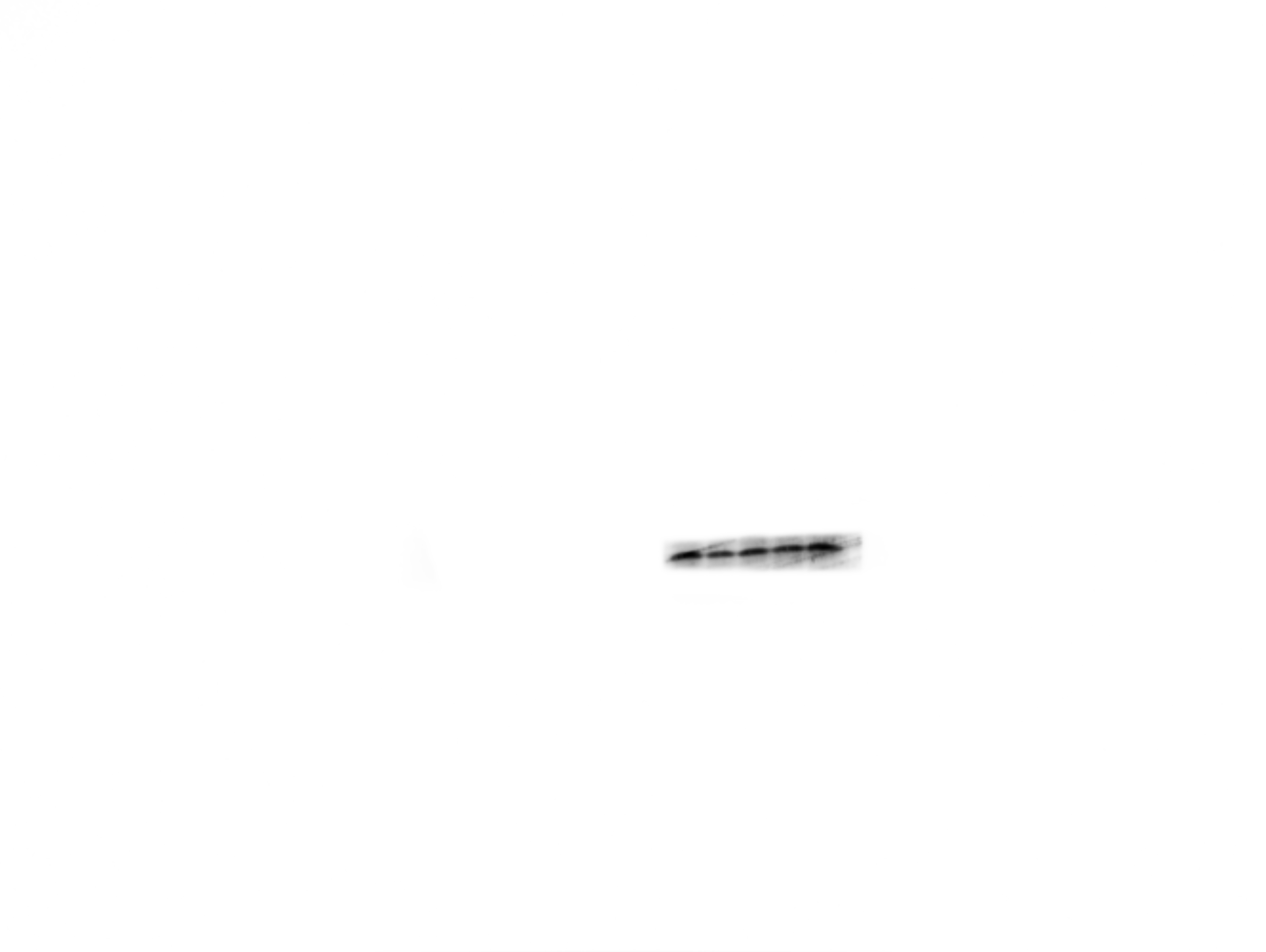

Supplement: S2 File — Original picture of the western blot experiments in the manuscript. (ZIP) [file pone.0274620.s002.zip › S2. blot results/Fig 3/p-Src/2sham/3.tif]

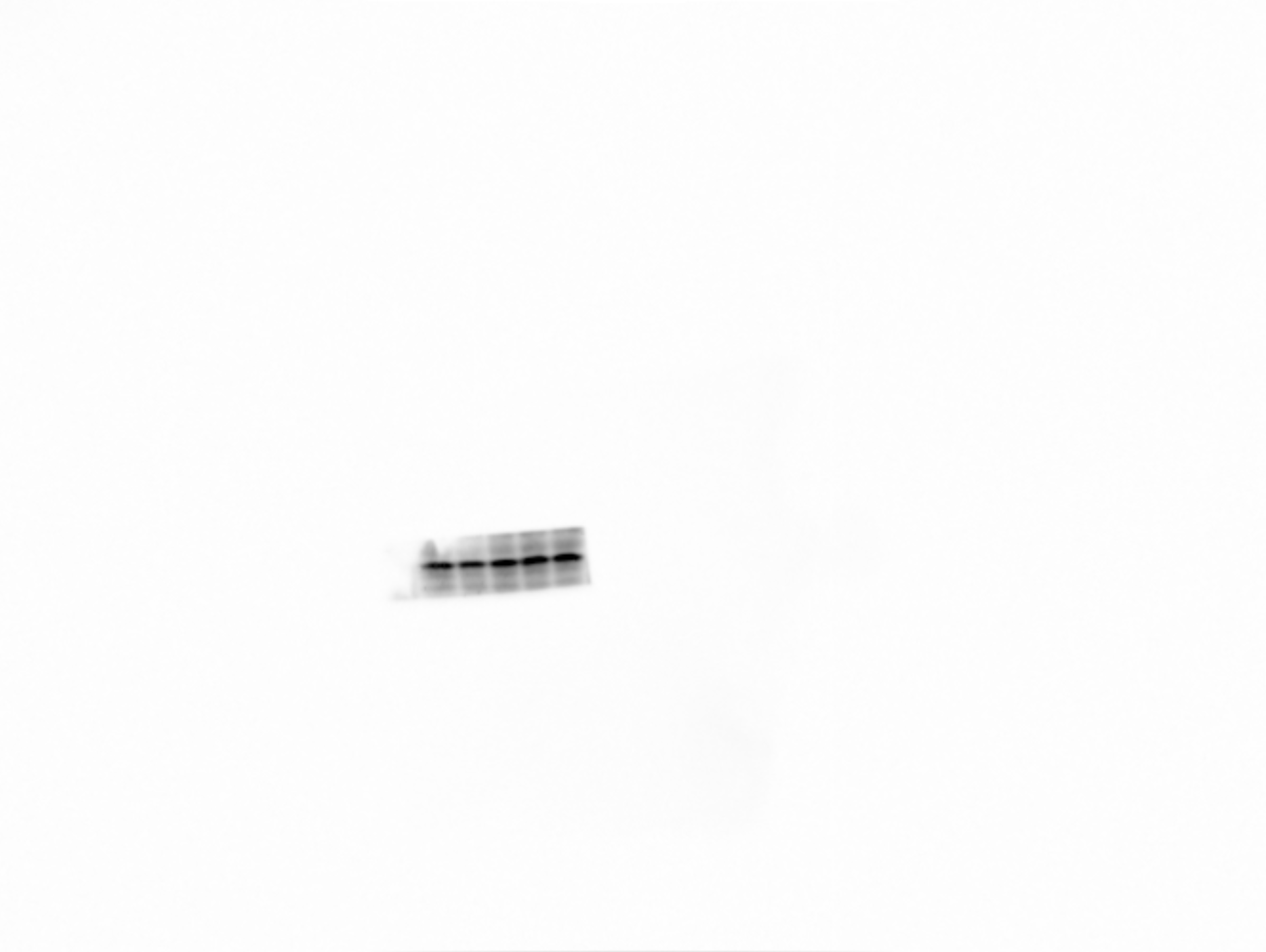

Supplement: S2 File — Original picture of the western blot experiments in the manuscript. (ZIP) [file pone.0274620.s002.zip › S2. blot results/Fig 3/p-Src/2sham/4.tif]

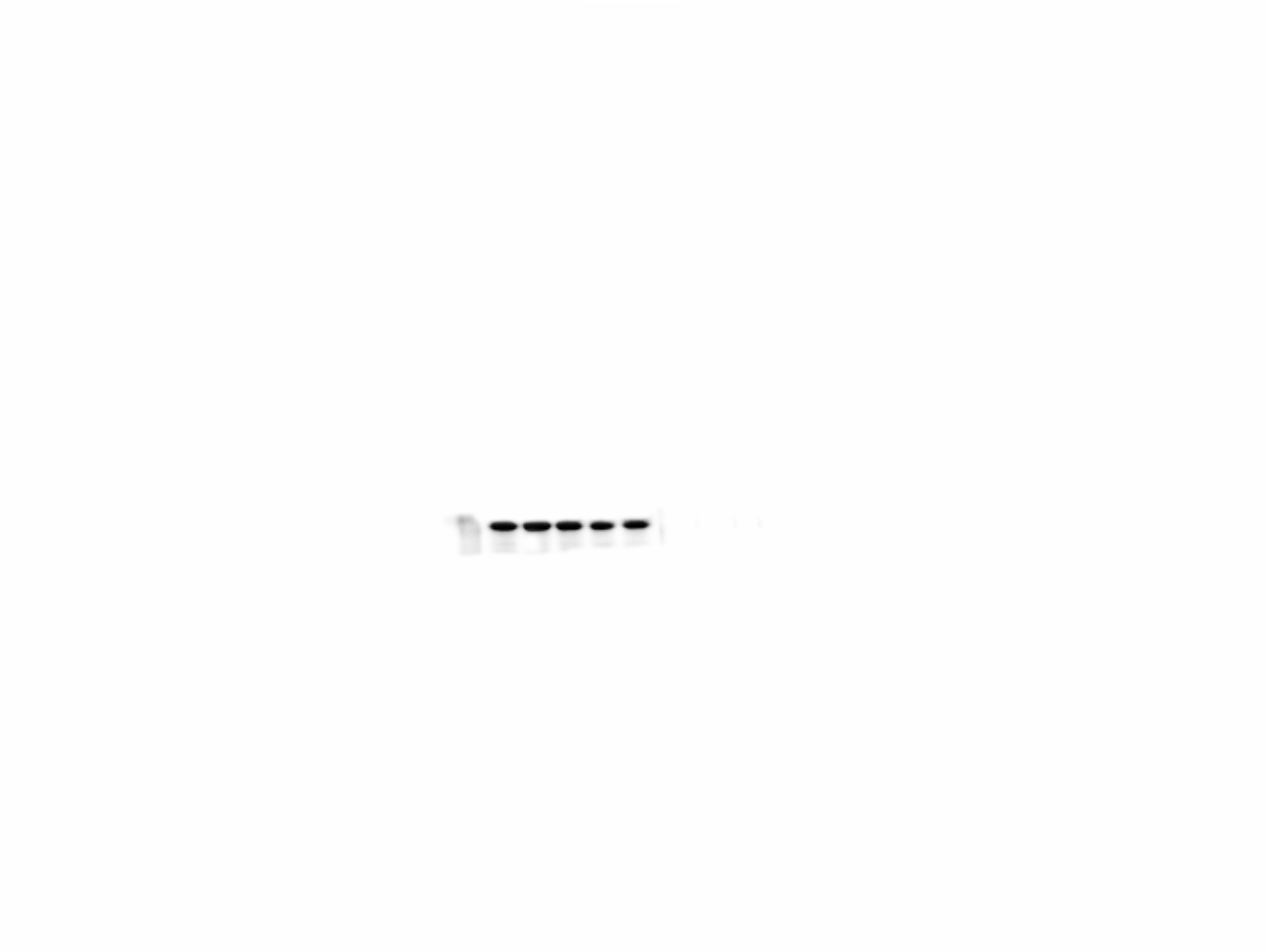

Supplement: S2 File — Original picture of the western blot experiments in the manuscript. (ZIP) [file pone.0274620.s002.zip › S2. blot results/Fig 3/p-Src/2sham/5.tif]

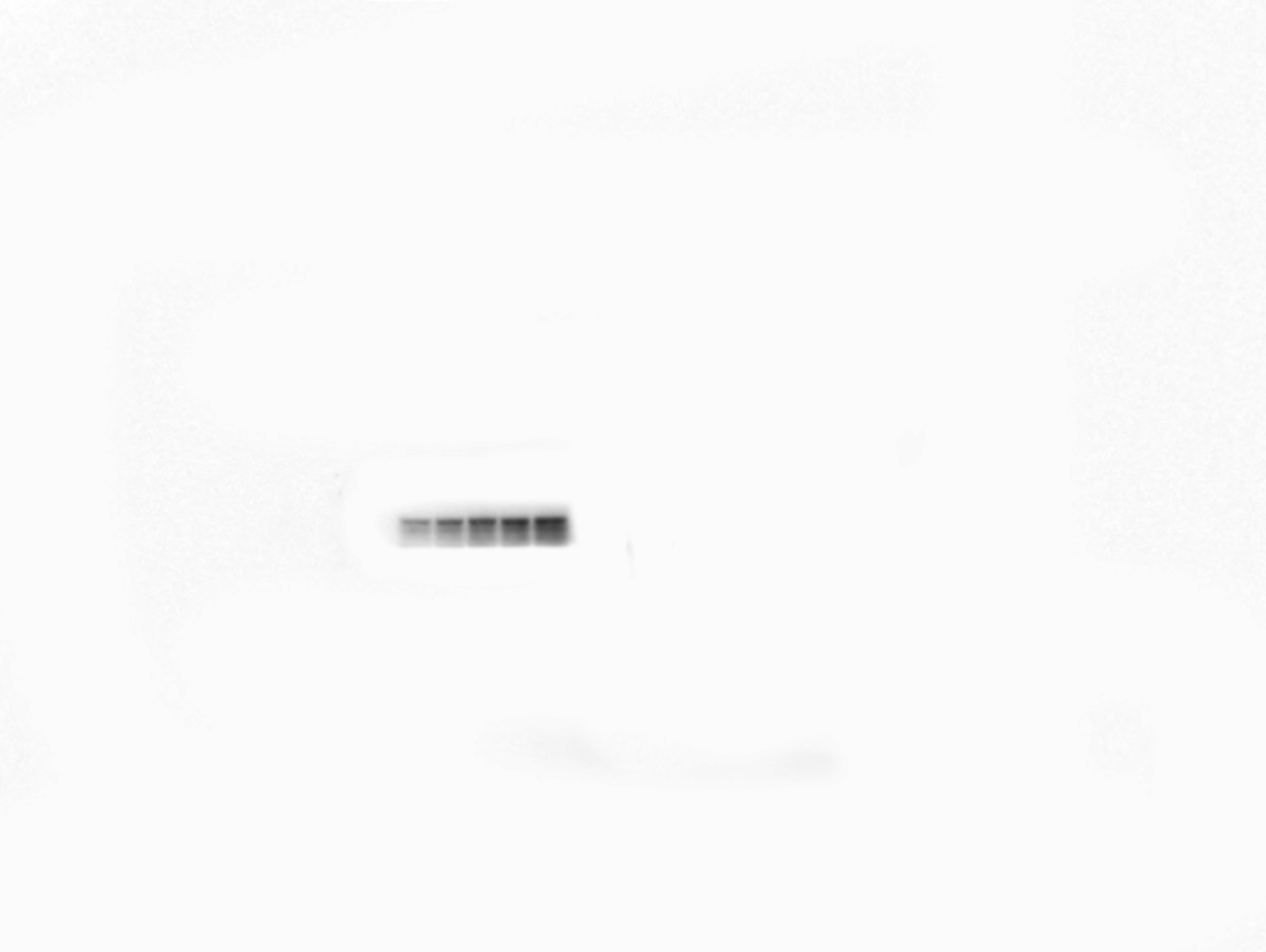

Supplement: S2 File — Original picture of the western blot experiments in the manuscript. (ZIP) [file pone.0274620.s002.zip › S2. blot results/Fig 3/p-Src/3model/1.tif]

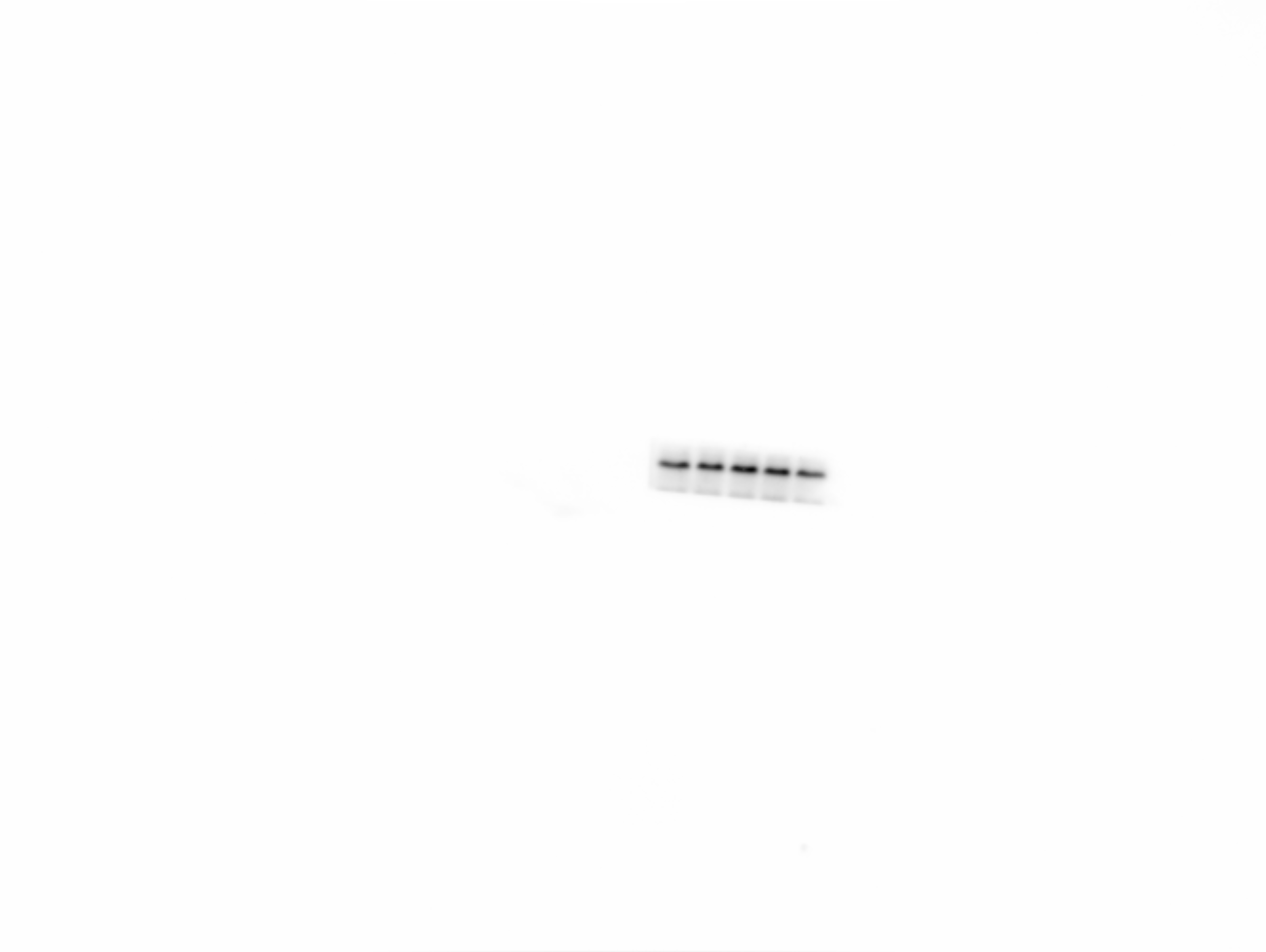

Supplement: S2 File — Original picture of the western blot experiments in the manuscript. (ZIP) [file pone.0274620.s002.zip › S2. blot results/Fig 3/p-Src/3model/2.tif]

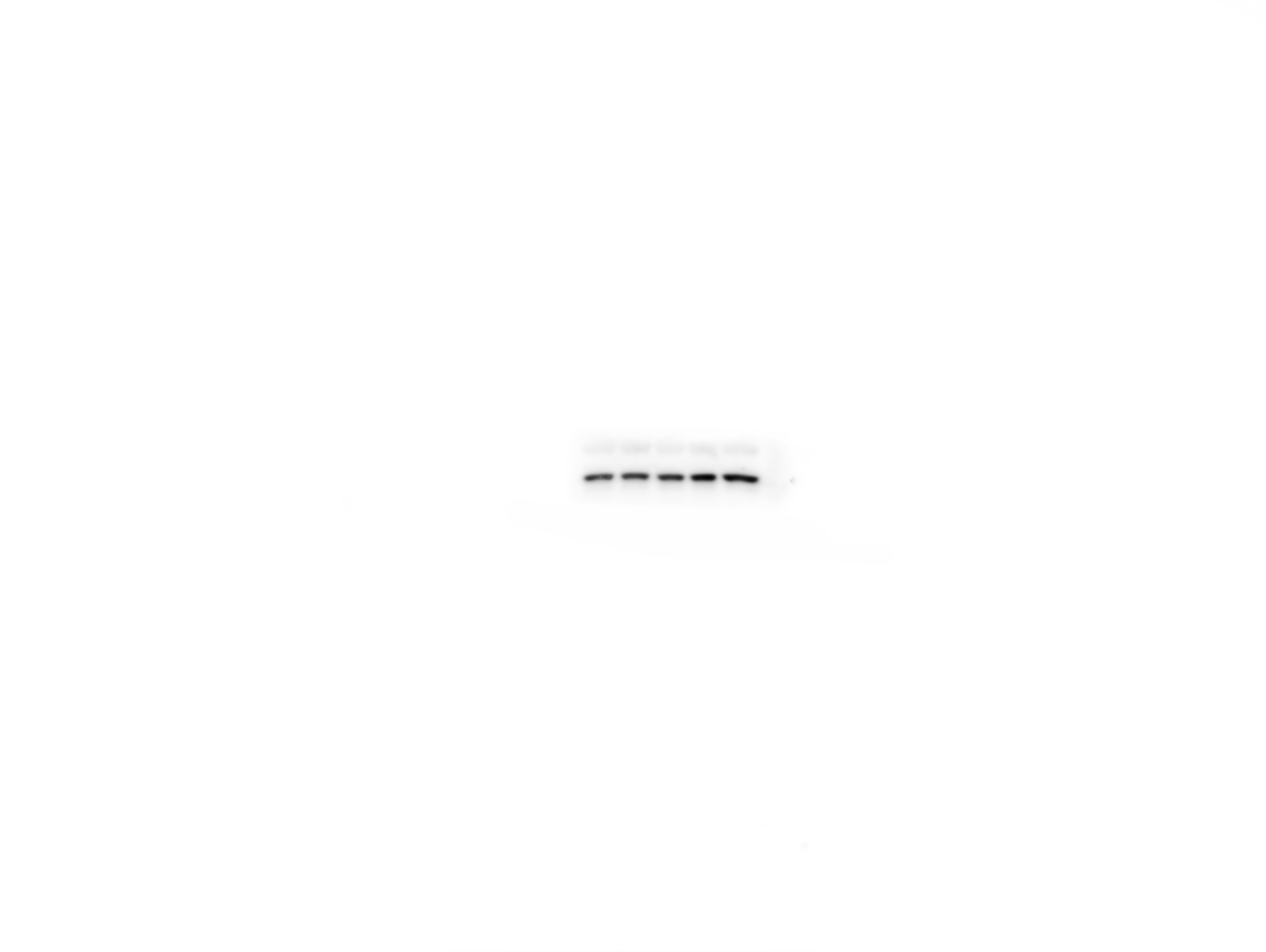

Supplement: S2 File — Original picture of the western blot experiments in the manuscript. (ZIP) [file pone.0274620.s002.zip › S2. blot results/Fig 3/p-Src/3model/3.tif]

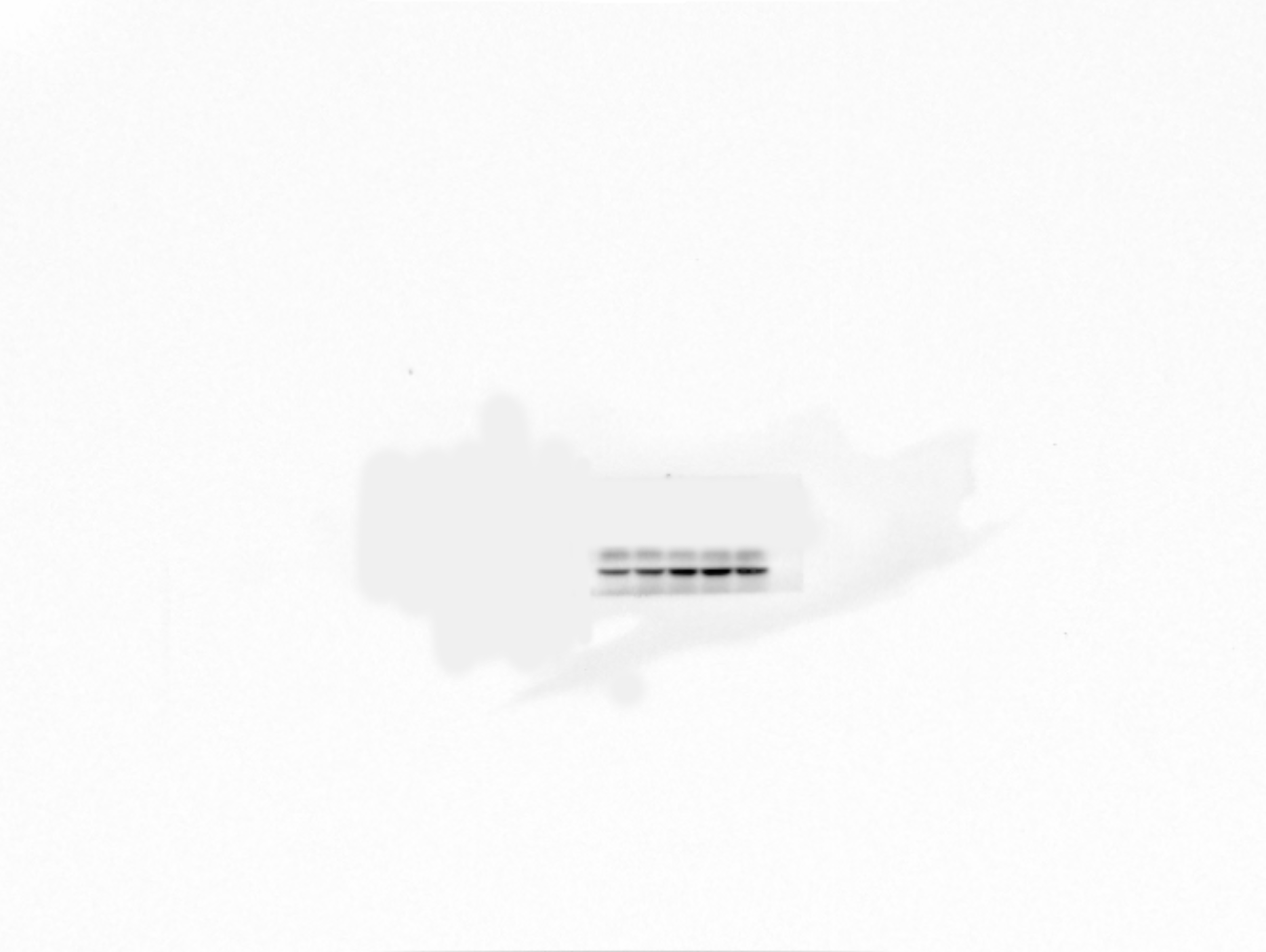

Supplement: S2 File — Original picture of the western blot experiments in the manuscript. (ZIP) [file pone.0274620.s002.zip › S2. blot results/Fig 3/p-Src/3model/4.tif]

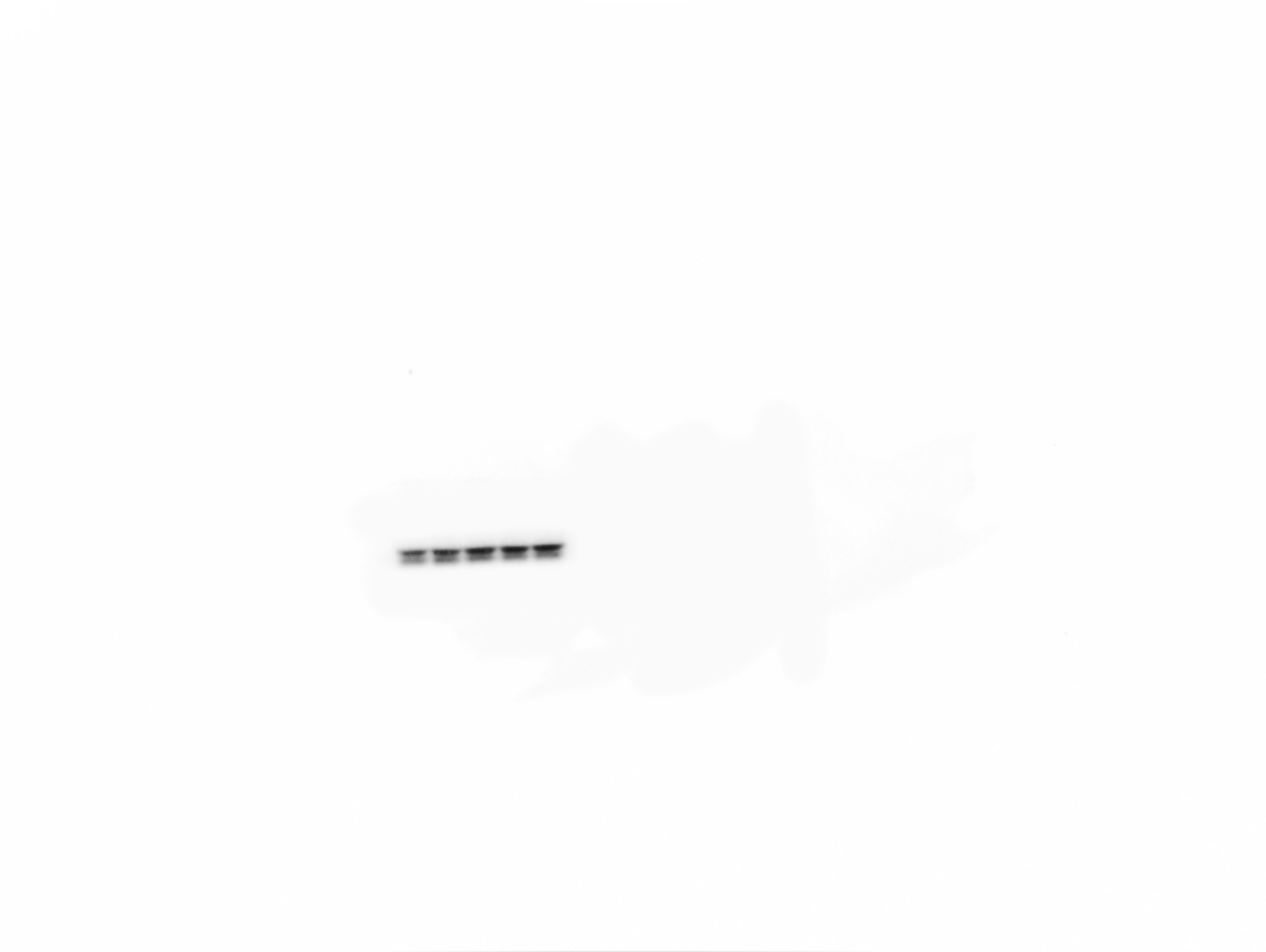

Supplement: S2 File — Original picture of the western blot experiments in the manuscript. (ZIP) [file pone.0274620.s002.zip › S2. blot results/Fig 3/p-Src/3model/5.tif]

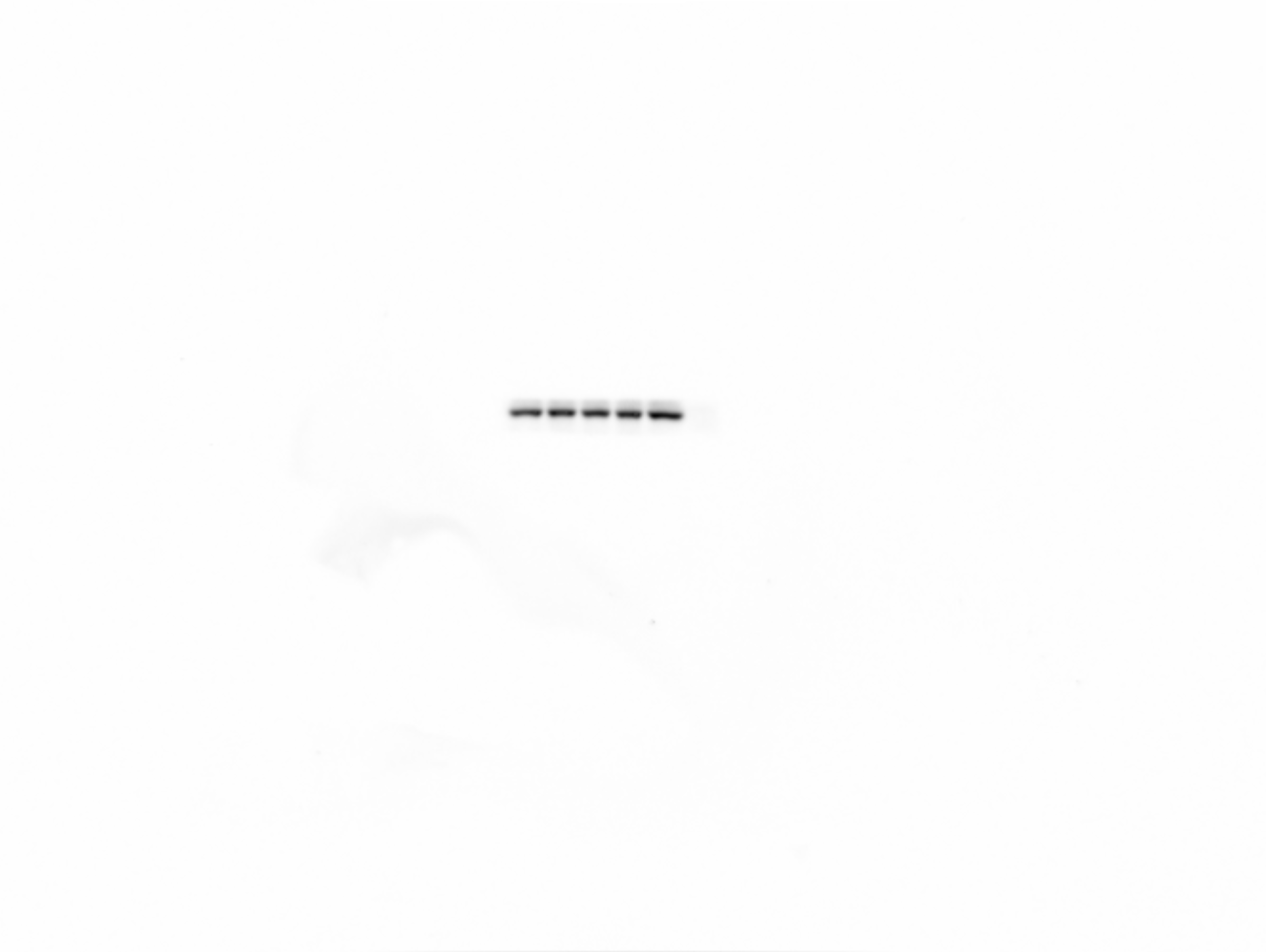

Supplement: S2 File — Original picture of the western blot experiments in the manuscript. (ZIP) [file pone.0274620.s002.zip › S2. blot results/Fig 3/p-Src/4EA/1.tif]

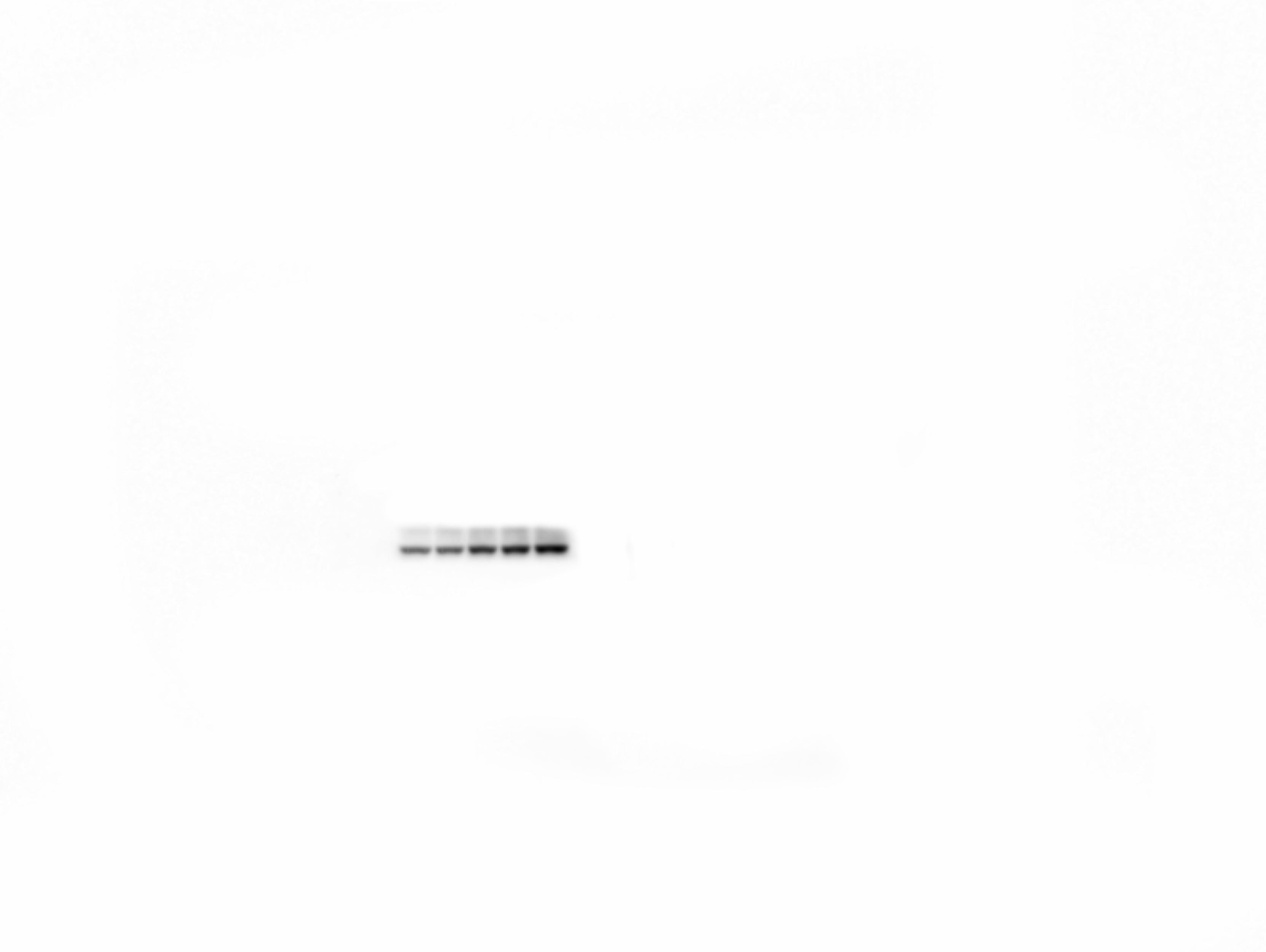

Supplement: S2 File — Original picture of the western blot experiments in the manuscript. (ZIP) [file pone.0274620.s002.zip › S2. blot results/Fig 3/p-Src/4EA/2.tif]

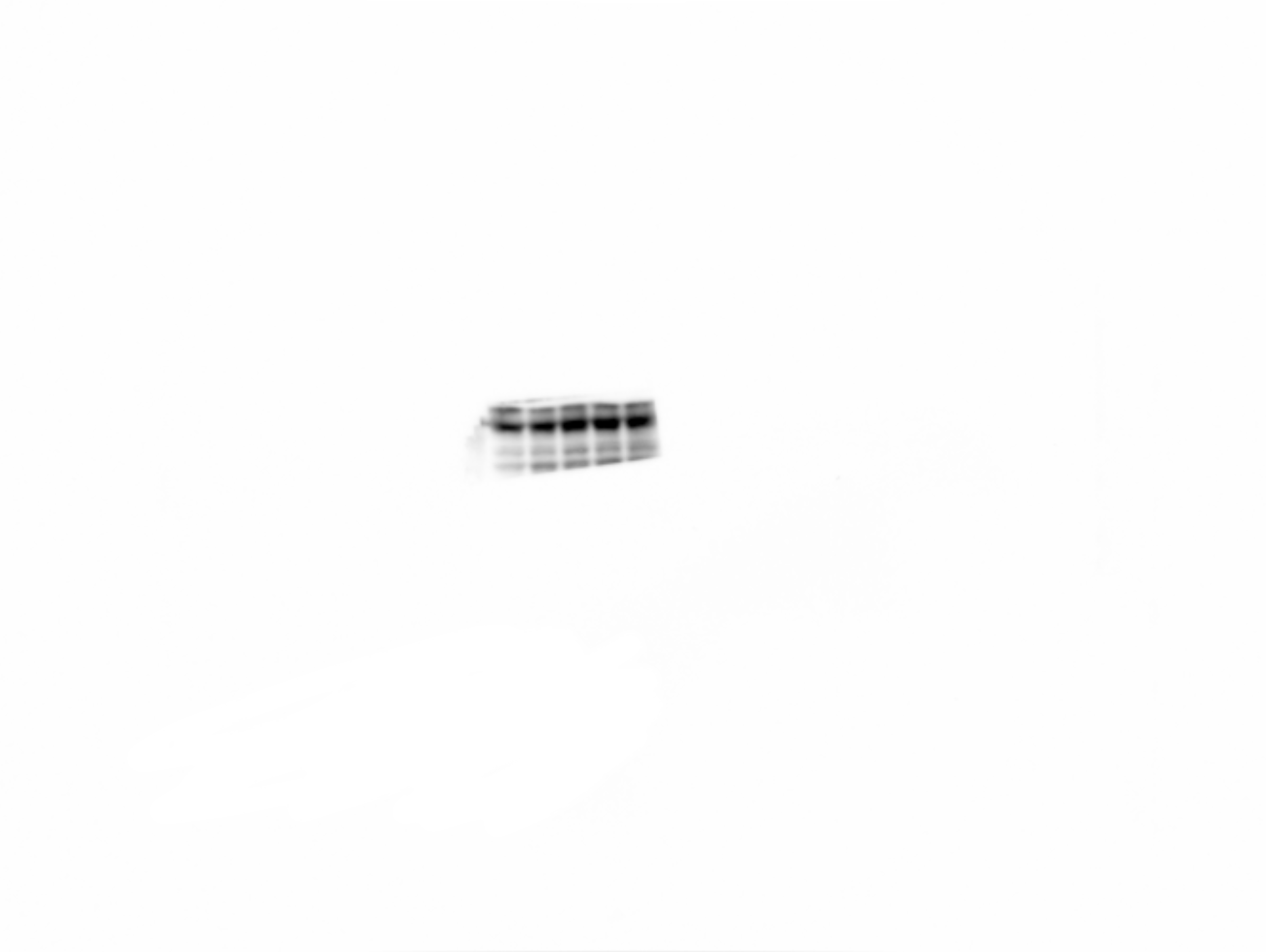

Supplement: S2 File — Original picture of the western blot experiments in the manuscript. (ZIP) [file pone.0274620.s002.zip › S2. blot results/Fig 3/p-Src/4EA/3.tif]

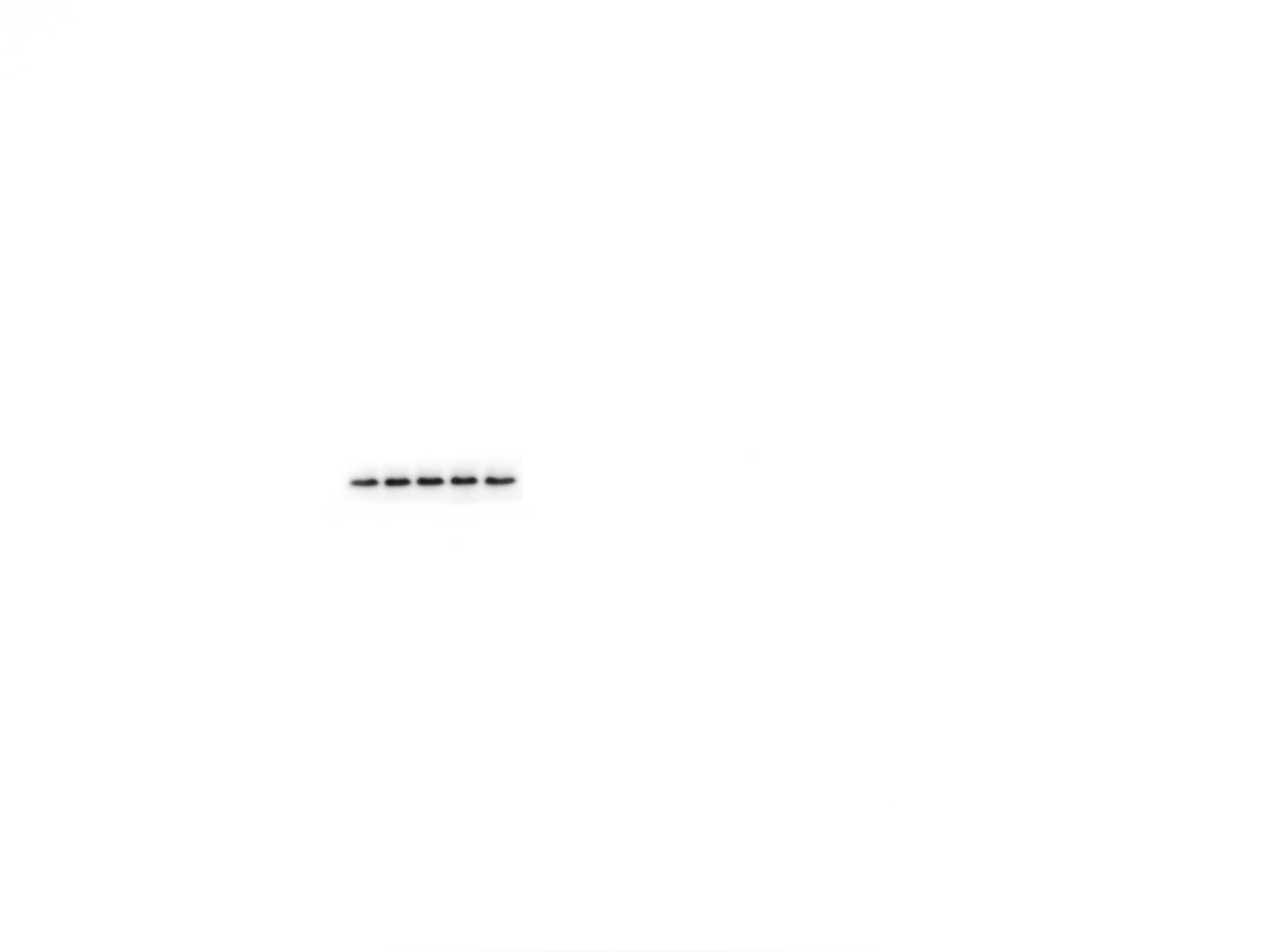

Supplement: S2 File — Original picture of the western blot experiments in the manuscript. (ZIP) [file pone.0274620.s002.zip › S2. blot results/Fig 3/p-Src/4EA/4.tif]

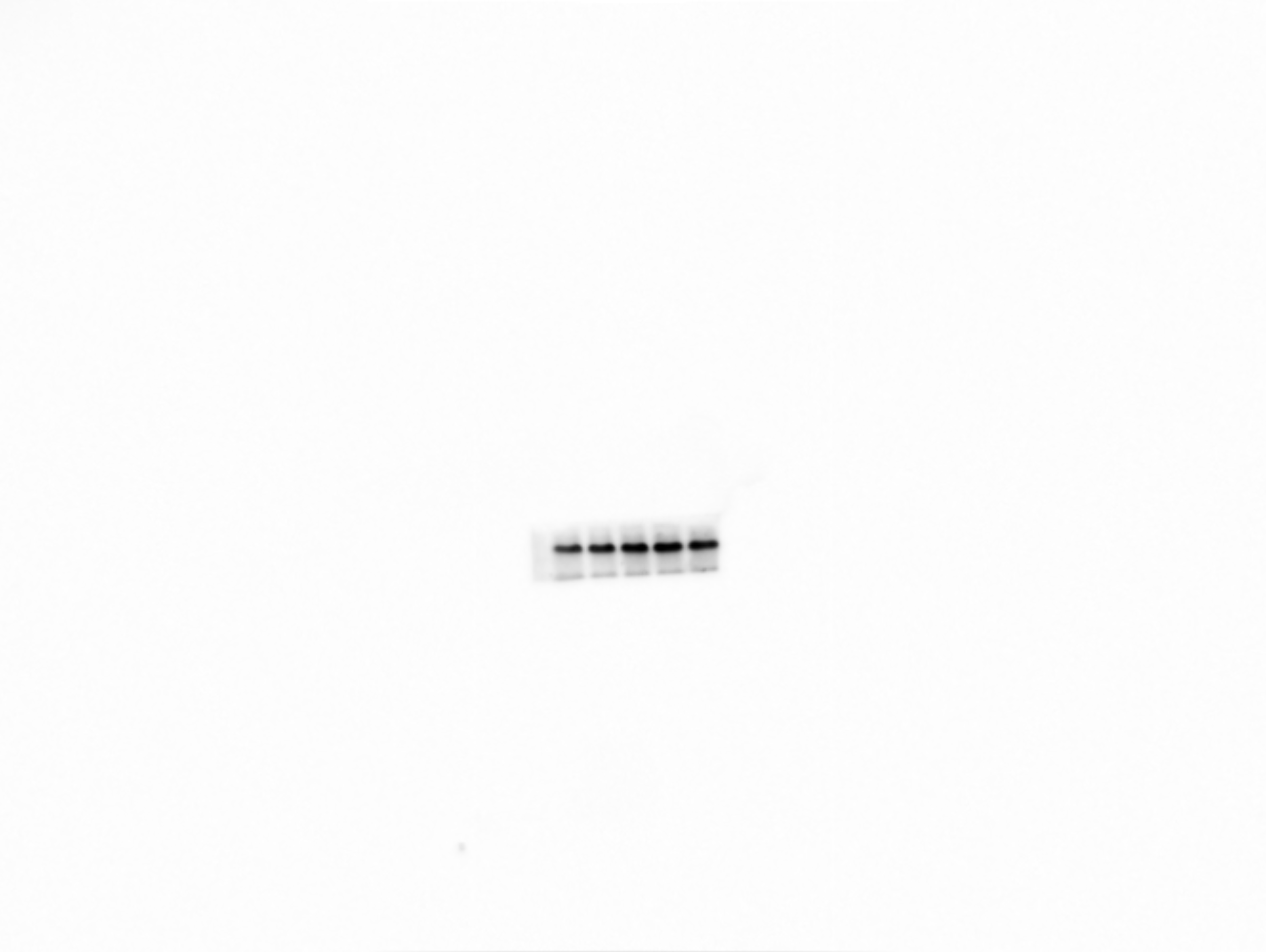

Supplement: S2 File — Original picture of the western blot experiments in the manuscript. (ZIP) [file pone.0274620.s002.zip › S2. blot results/Fig 3/p-Src/4EA/5.tif]

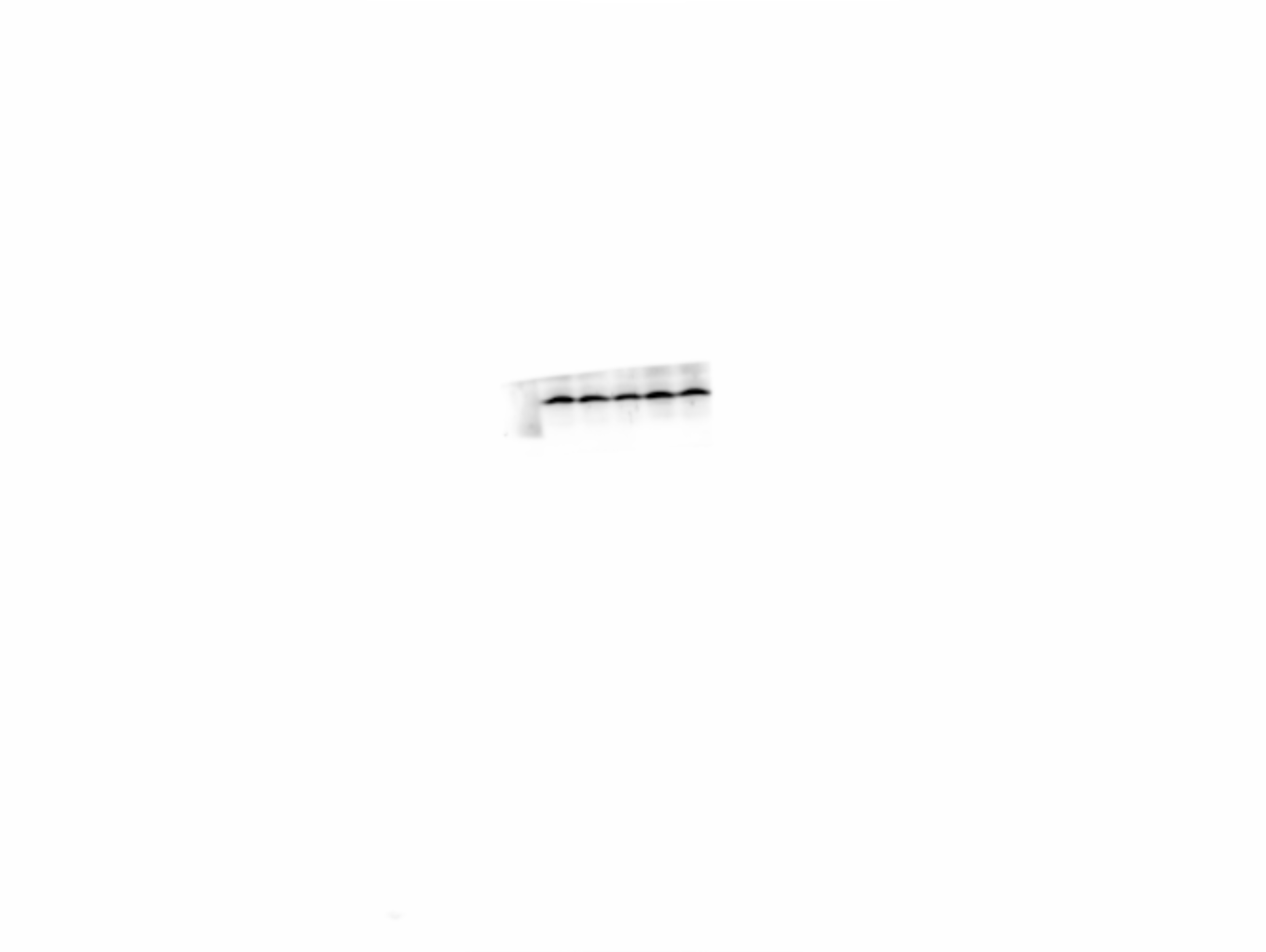

Supplement: S2 File — Original picture of the western blot experiments in the manuscript. (ZIP) [file pone.0274620.s002.zip › S2. blot results/Fig 3/VEGF/1control/1.tif]

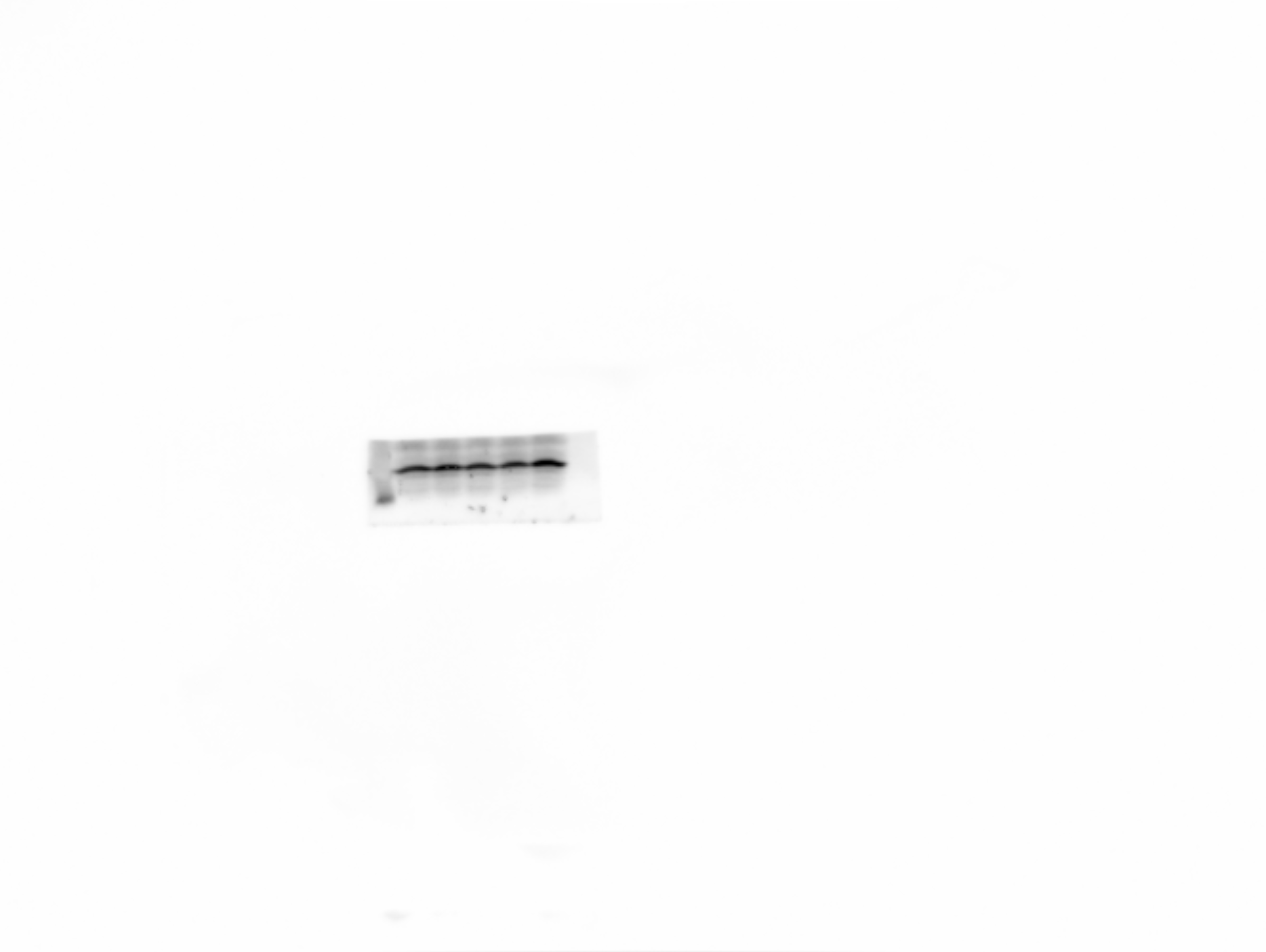

Supplement: S2 File — Original picture of the western blot experiments in the manuscript. (ZIP) [file pone.0274620.s002.zip › S2. blot results/Fig 3/VEGF/1control/2.tif]

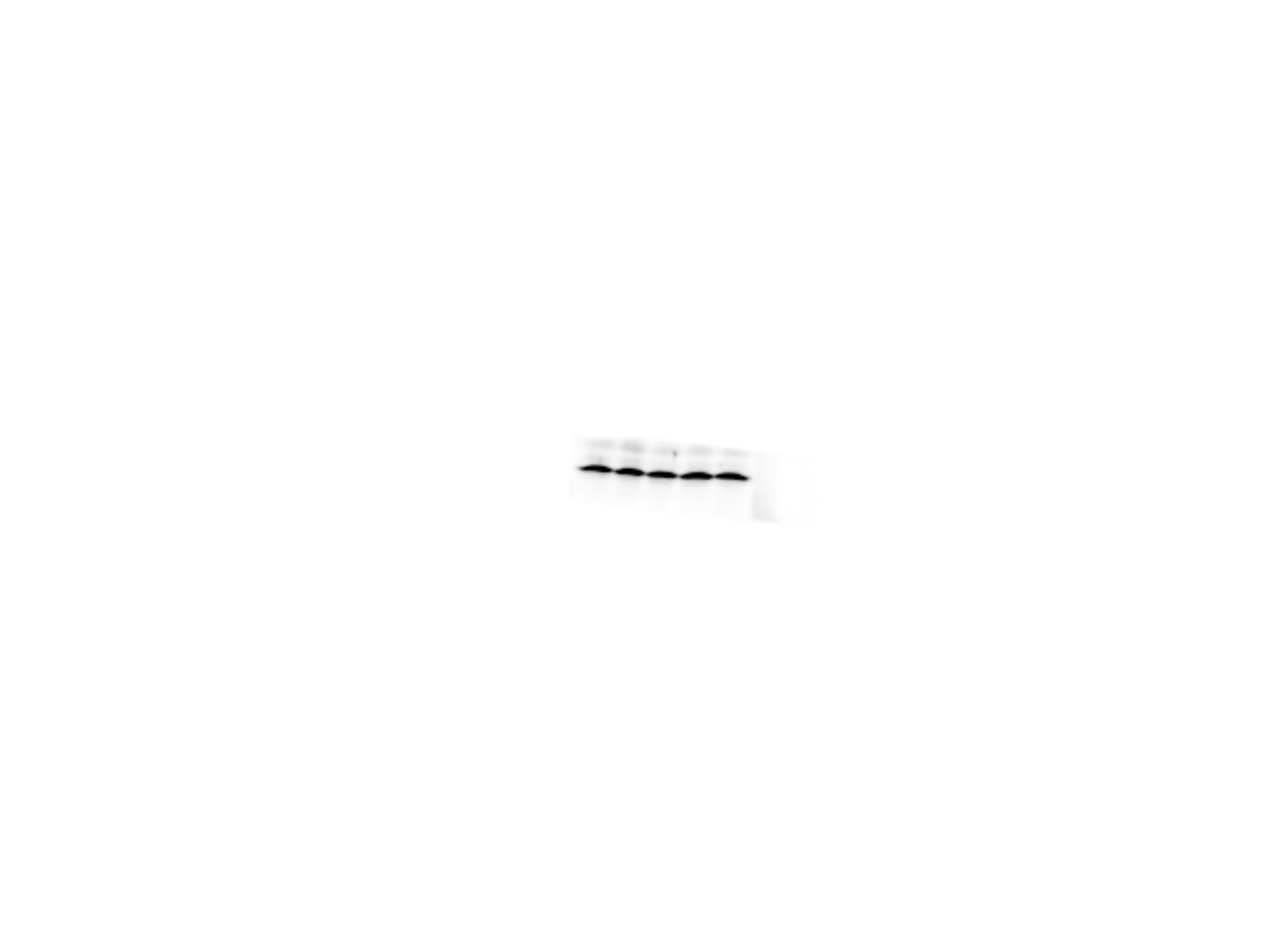

Supplement: S2 File — Original picture of the western blot experiments in the manuscript. (ZIP) [file pone.0274620.s002.zip › S2. blot results/Fig 3/VEGF/1control/3.tif]

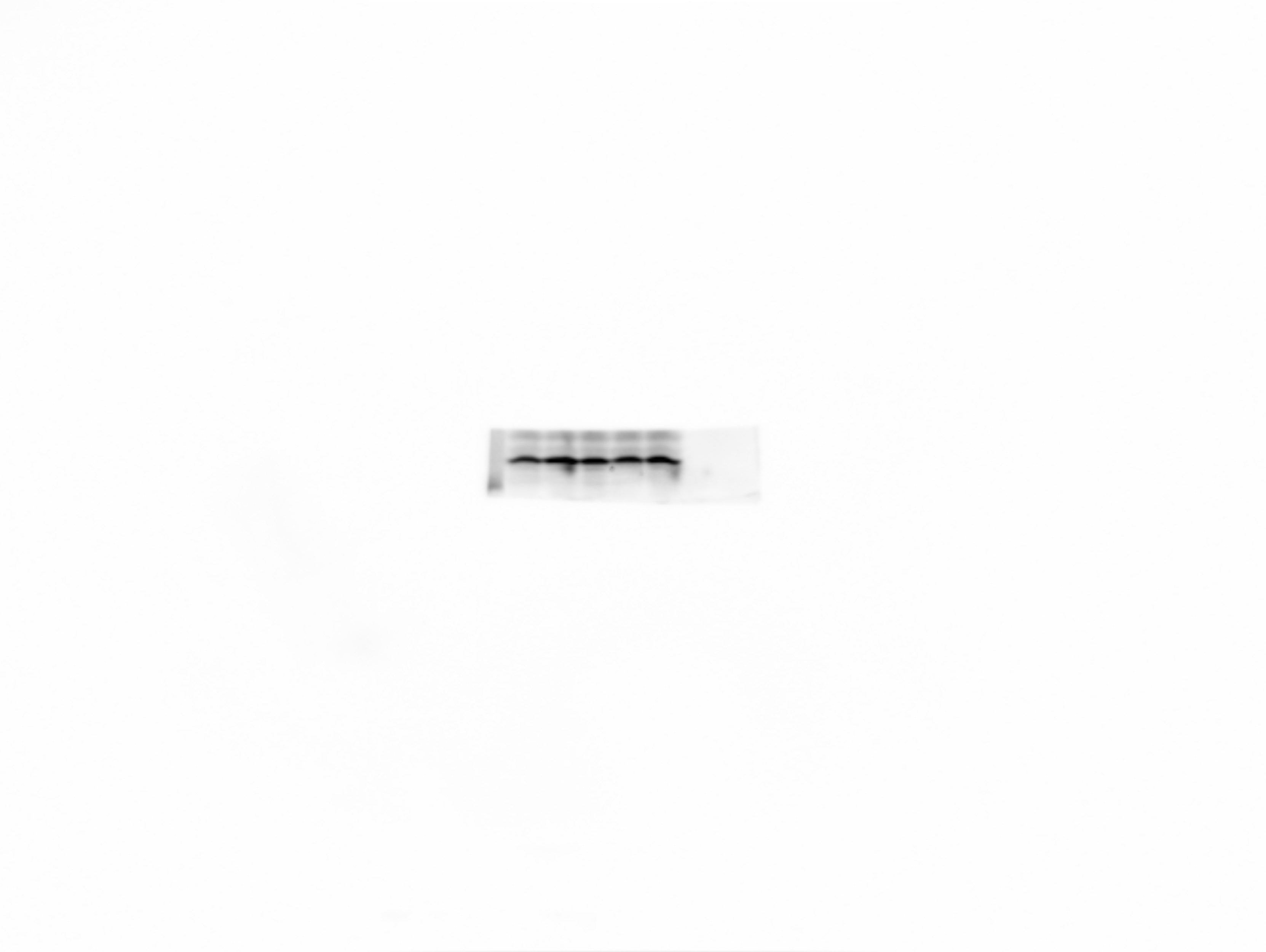

Supplement: S2 File — Original picture of the western blot experiments in the manuscript. (ZIP) [file pone.0274620.s002.zip › S2. blot results/Fig 3/VEGF/1control/4.tif]

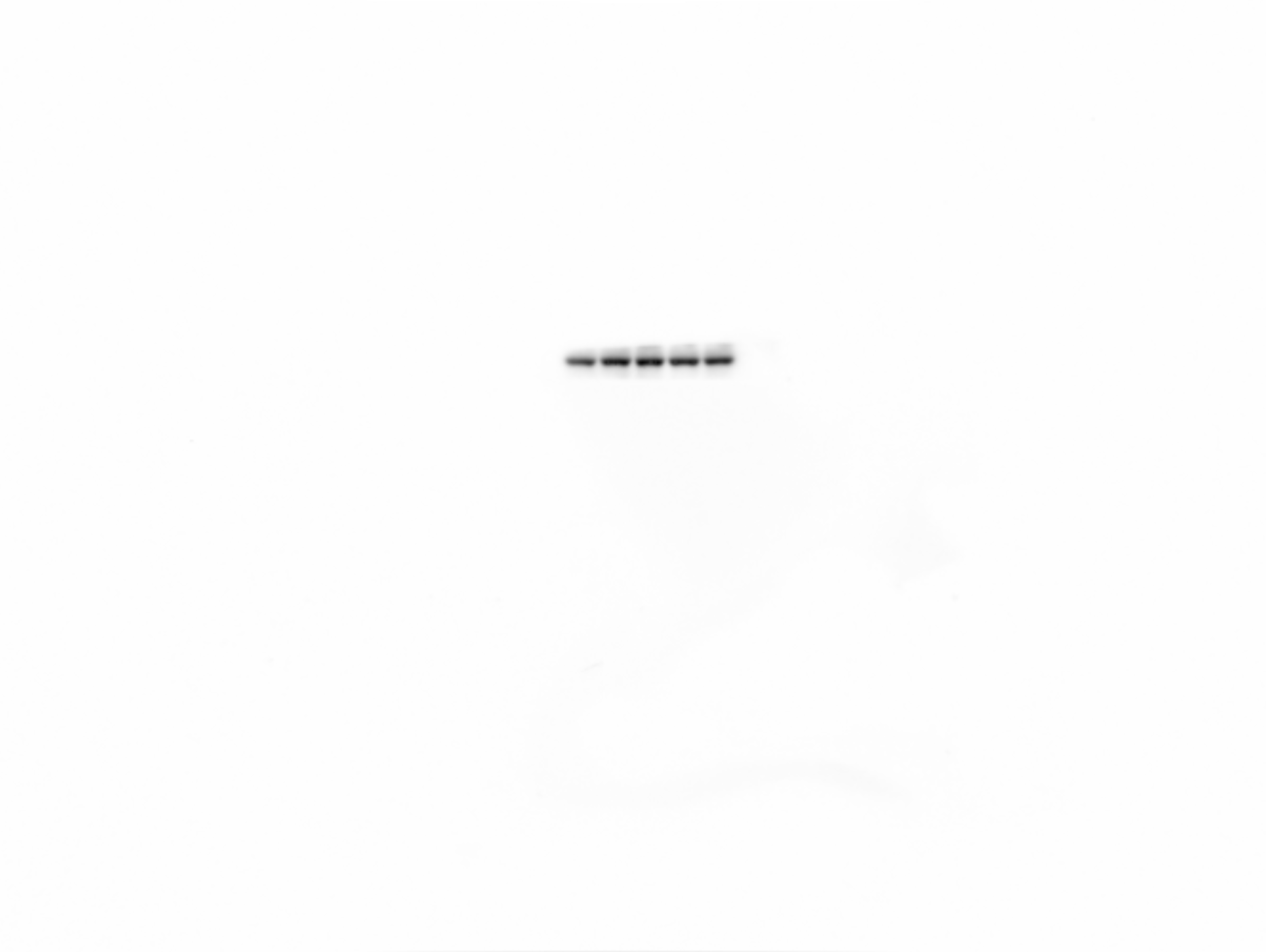

Supplement: S2 File — Original picture of the western blot experiments in the manuscript. (ZIP) [file pone.0274620.s002.zip › S2. blot results/Fig 3/VEGF/1control/5.tif]

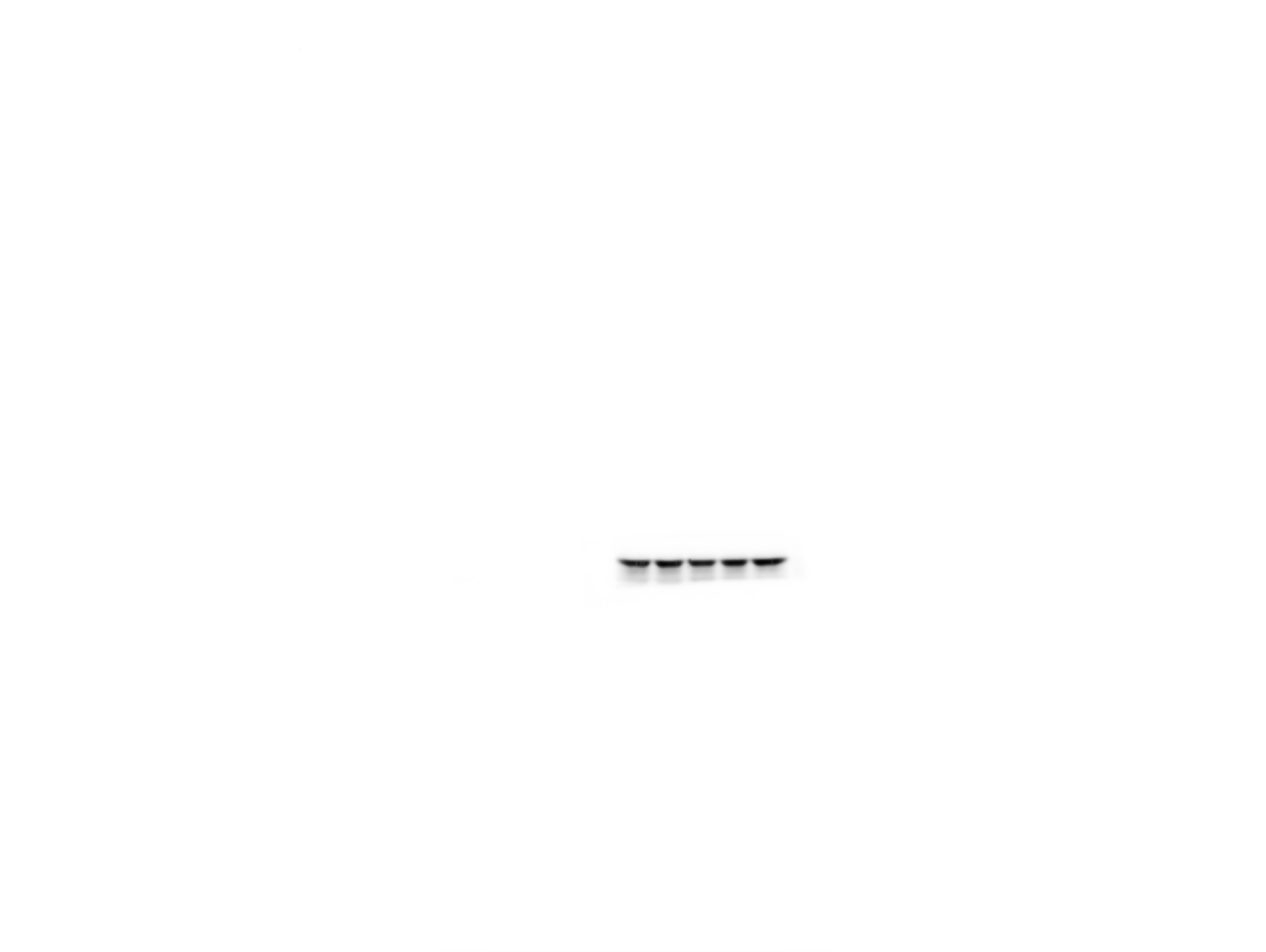

Supplement: S2 File — Original picture of the western blot experiments in the manuscript. (ZIP) [file pone.0274620.s002.zip › S2. blot results/Fig 3/VEGF/2sham/1.tif]

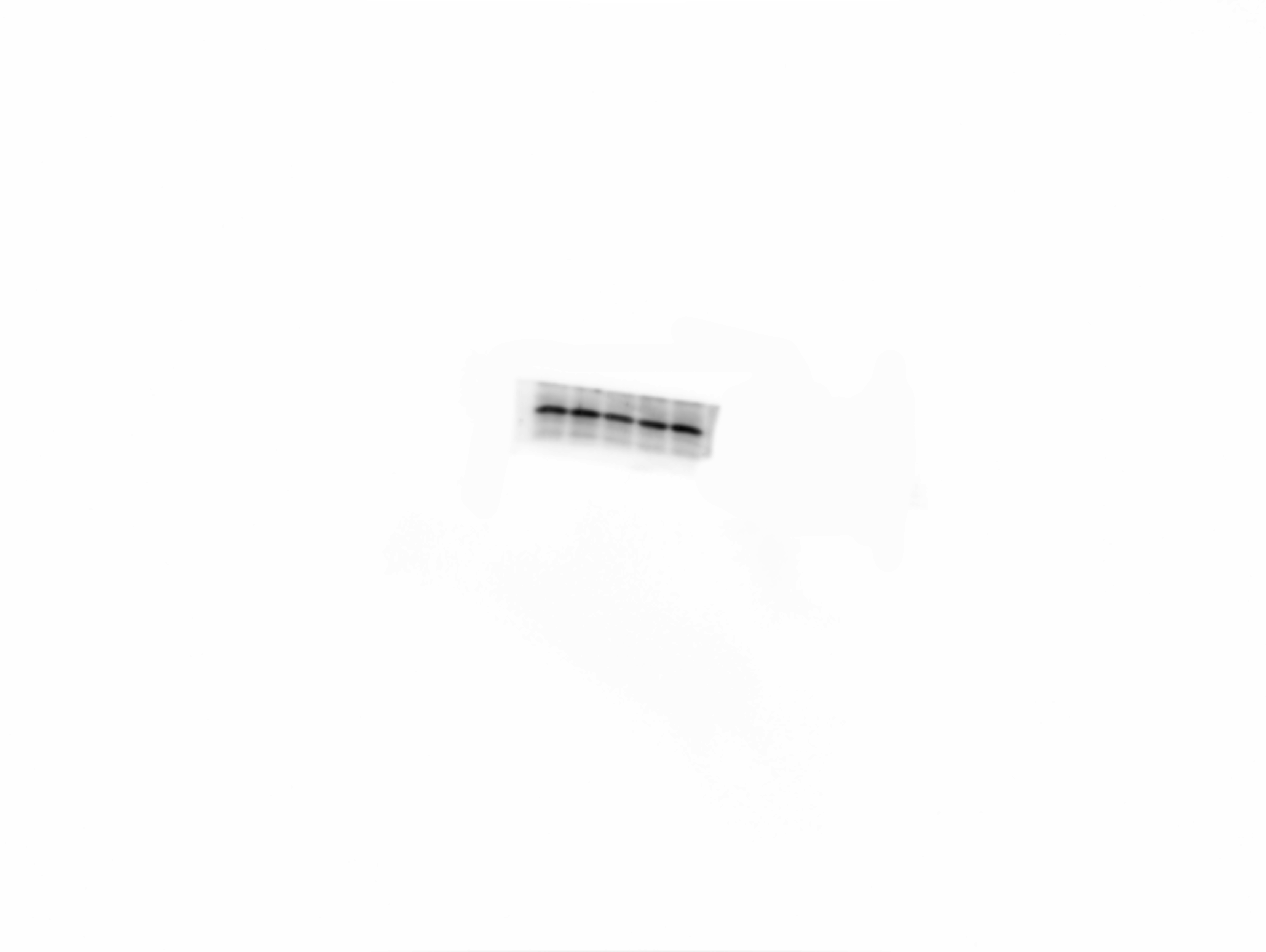

Supplement: S2 File — Original picture of the western blot experiments in the manuscript. (ZIP) [file pone.0274620.s002.zip › S2. blot results/Fig 3/VEGF/2sham/2.tif]

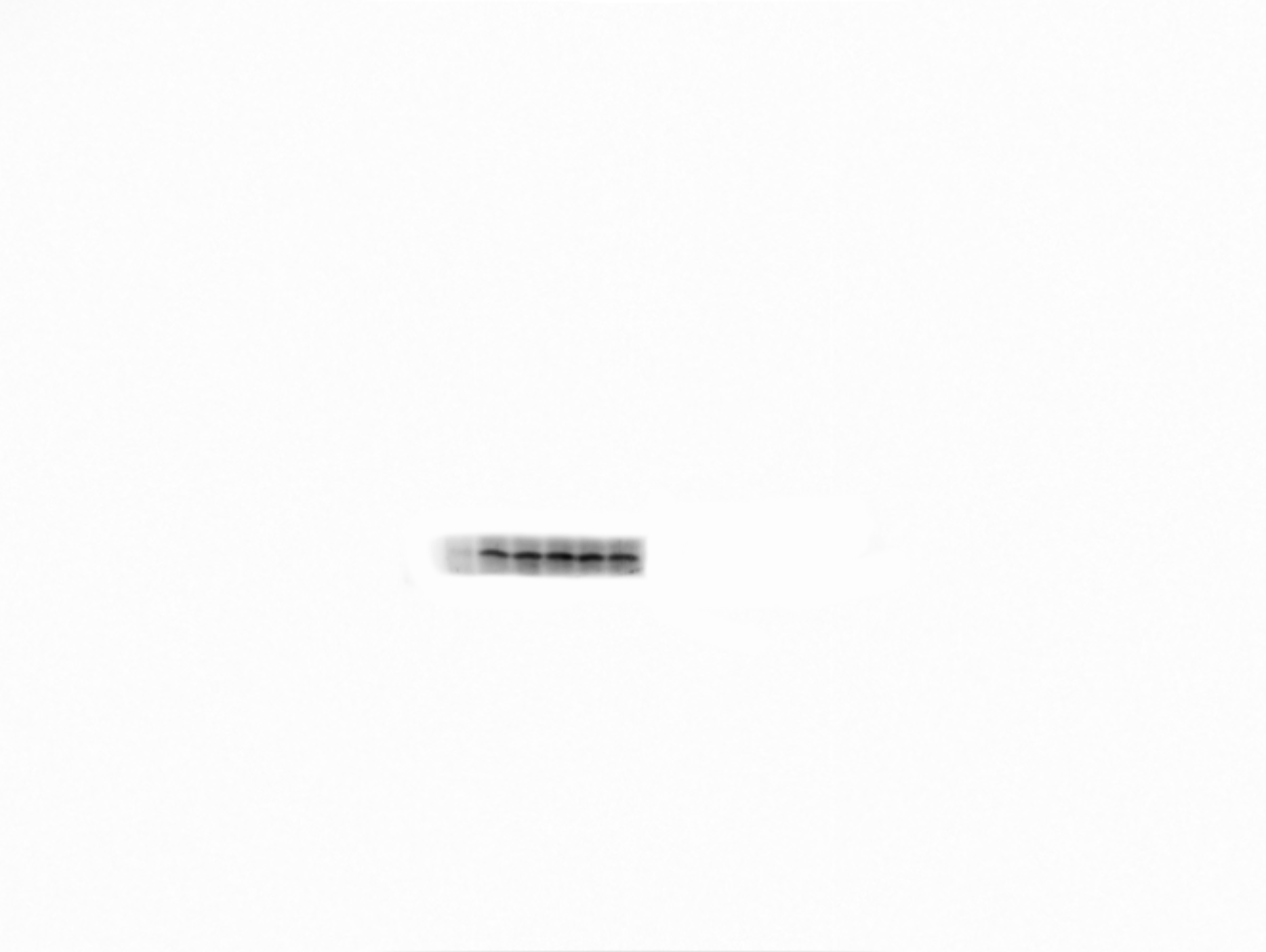

Supplement: S2 File — Original picture of the western blot experiments in the manuscript. (ZIP) [file pone.0274620.s002.zip › S2. blot results/Fig 3/VEGF/2sham/3.tif]

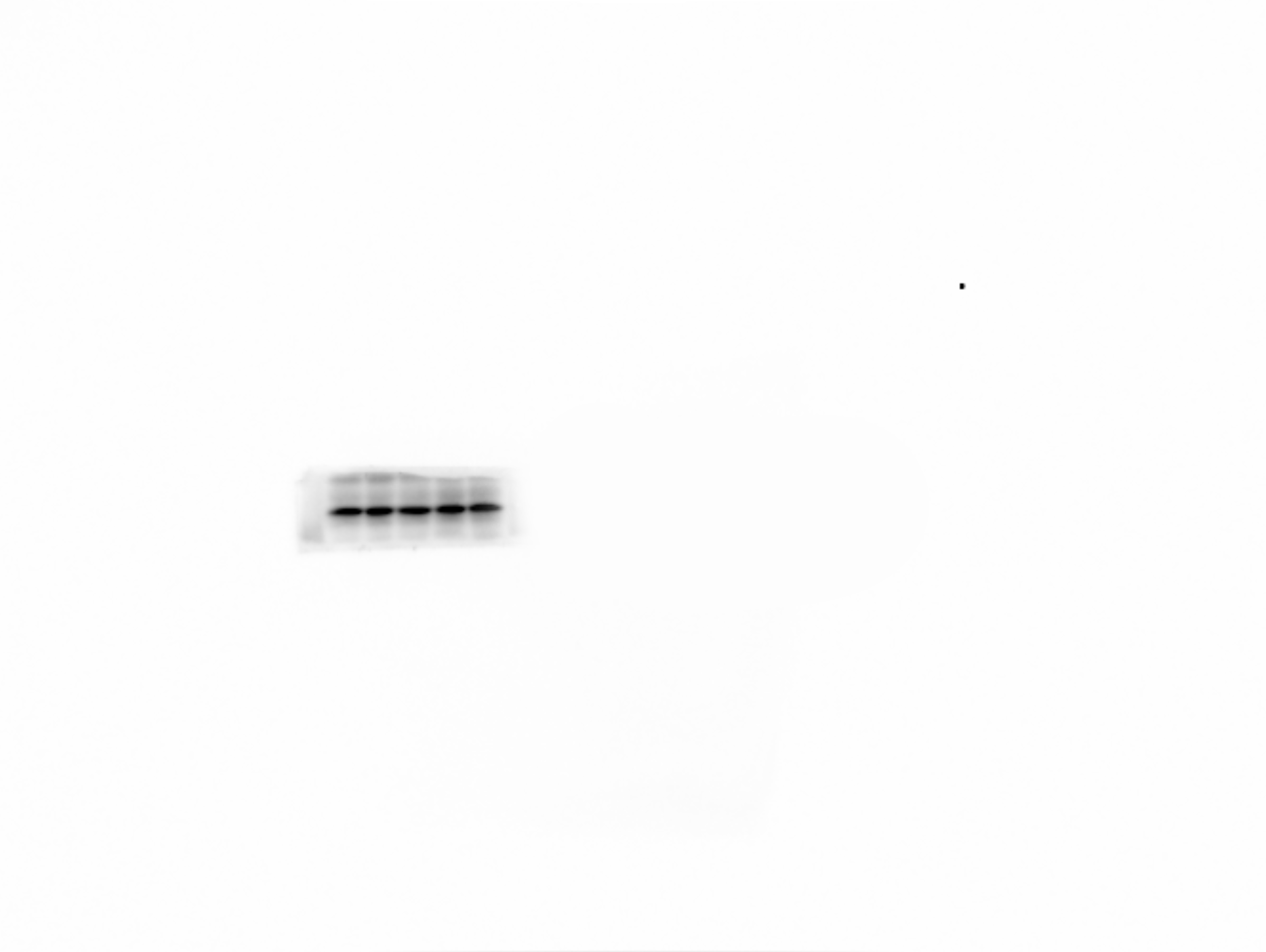

Supplement: S2 File — Original picture of the western blot experiments in the manuscript. (ZIP) [file pone.0274620.s002.zip › S2. blot results/Fig 3/VEGF/2sham/5.tif]

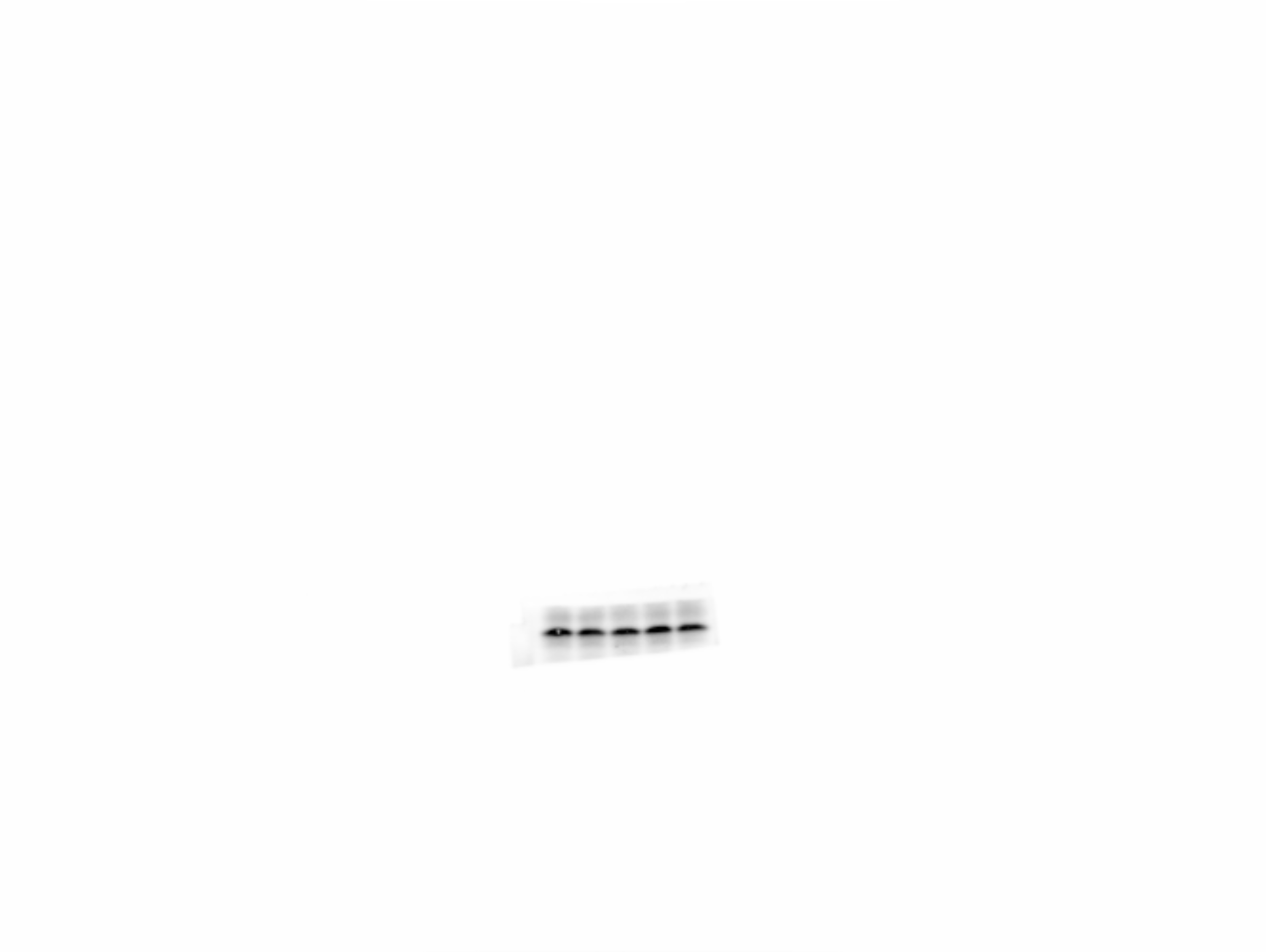

Supplement: S2 File — Original picture of the western blot experiments in the manuscript. (ZIP) [file pone.0274620.s002.zip › S2. blot results/Fig 3/VEGF/2sham/6.tif]

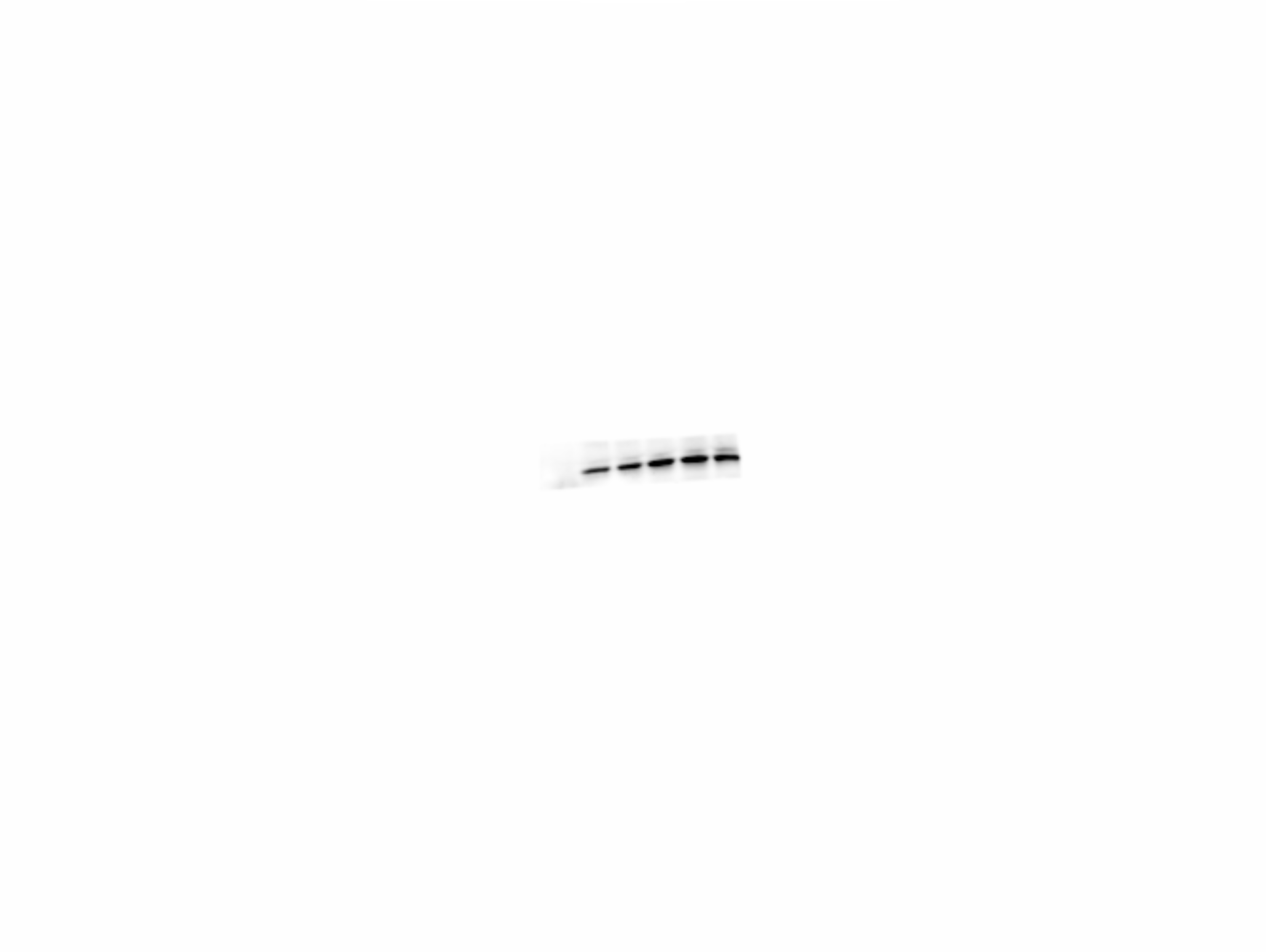

Supplement: S2 File — Original picture of the western blot experiments in the manuscript. (ZIP) [file pone.0274620.s002.zip › S2. blot results/Fig 3/VEGF/3model/1.tif]

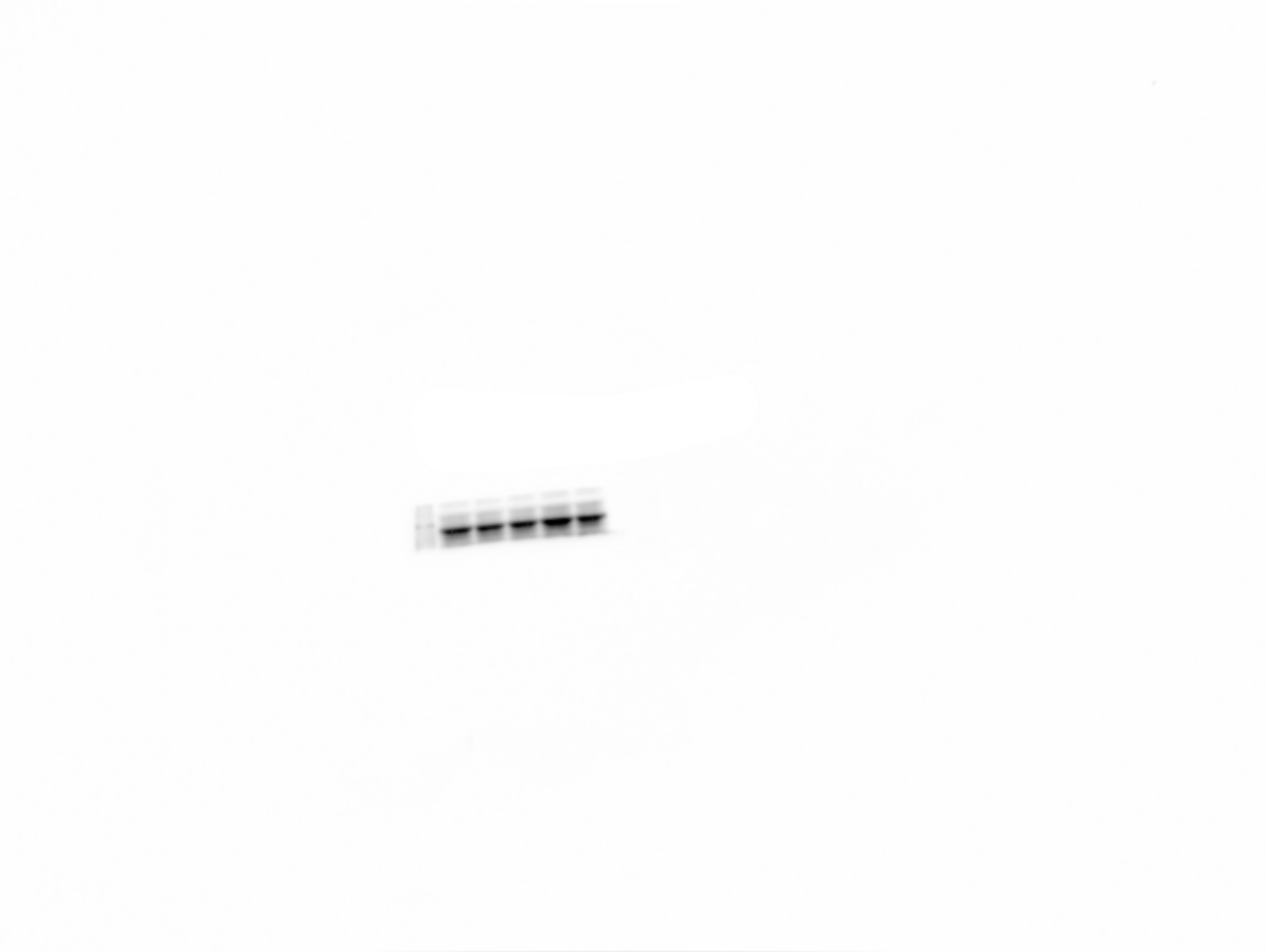

Supplement: S2 File — Original picture of the western blot experiments in the manuscript. (ZIP) [file pone.0274620.s002.zip › S2. blot results/Fig 3/VEGF/3model/2.tif]

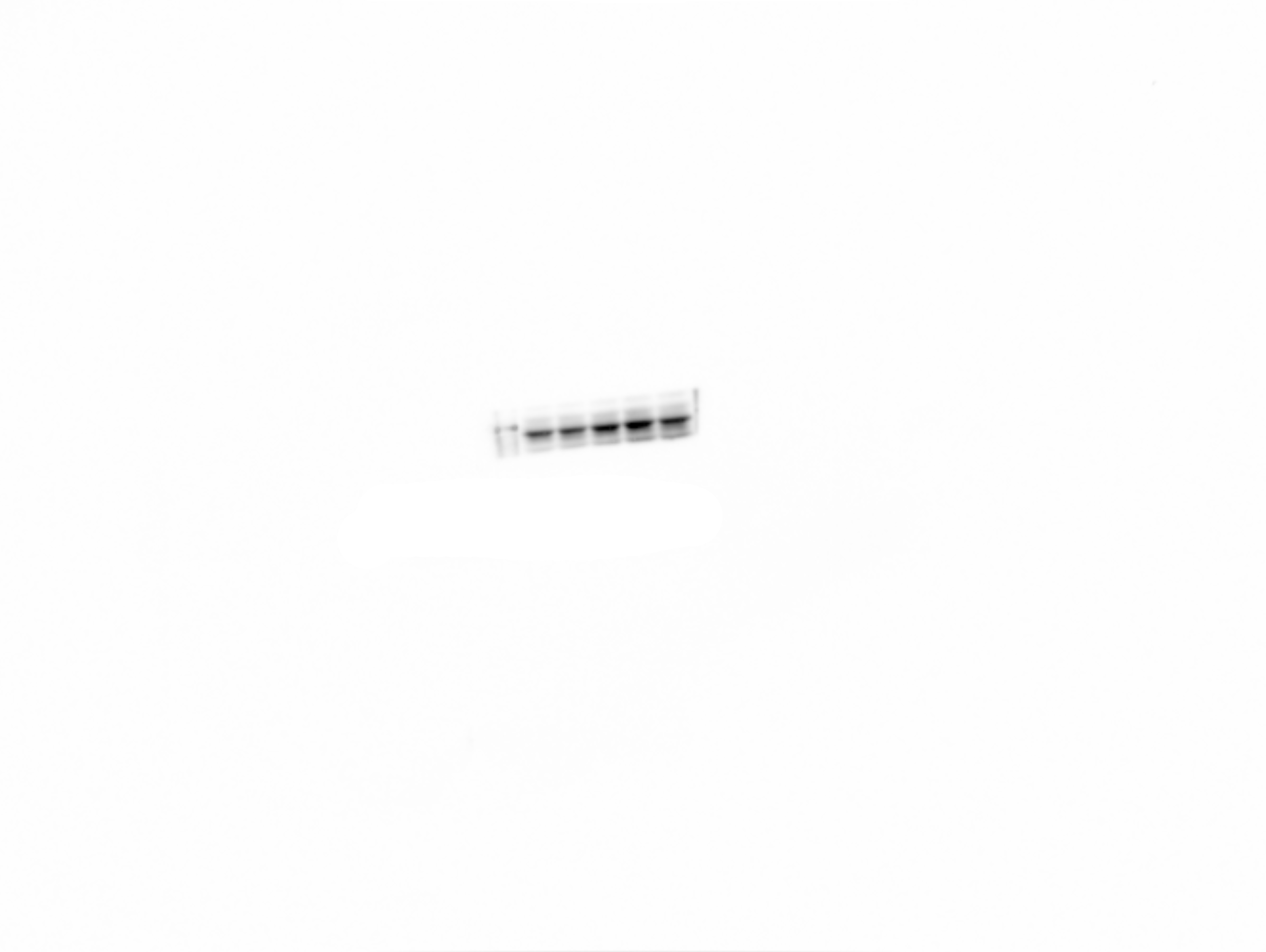

Supplement: S2 File — Original picture of the western blot experiments in the manuscript. (ZIP) [file pone.0274620.s002.zip › S2. blot results/Fig 3/VEGF/3model/3.tif]

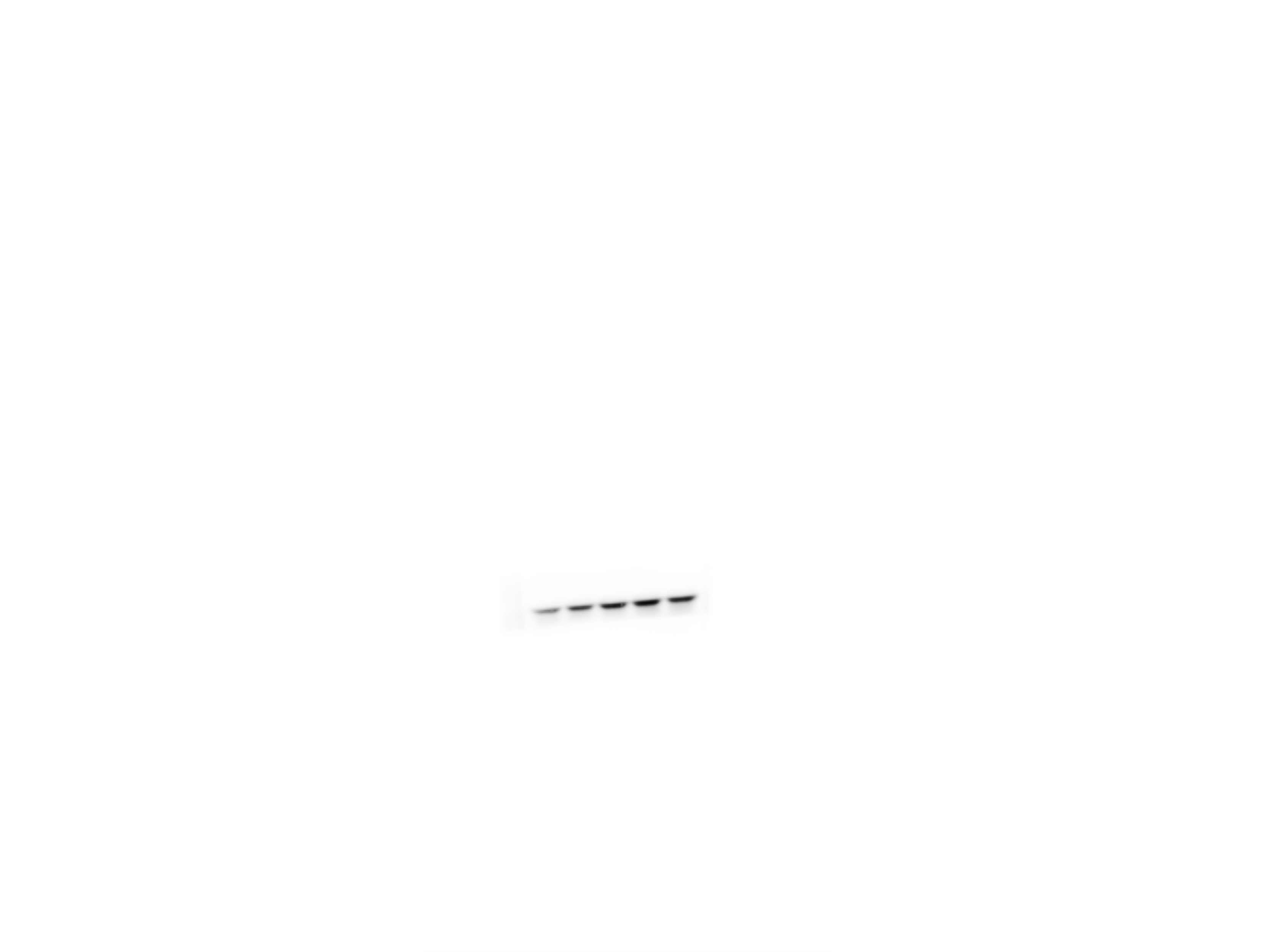

Supplement: S2 File — Original picture of the western blot experiments in the manuscript. (ZIP) [file pone.0274620.s002.zip › S2. blot results/Fig 3/VEGF/3model/4.tif]

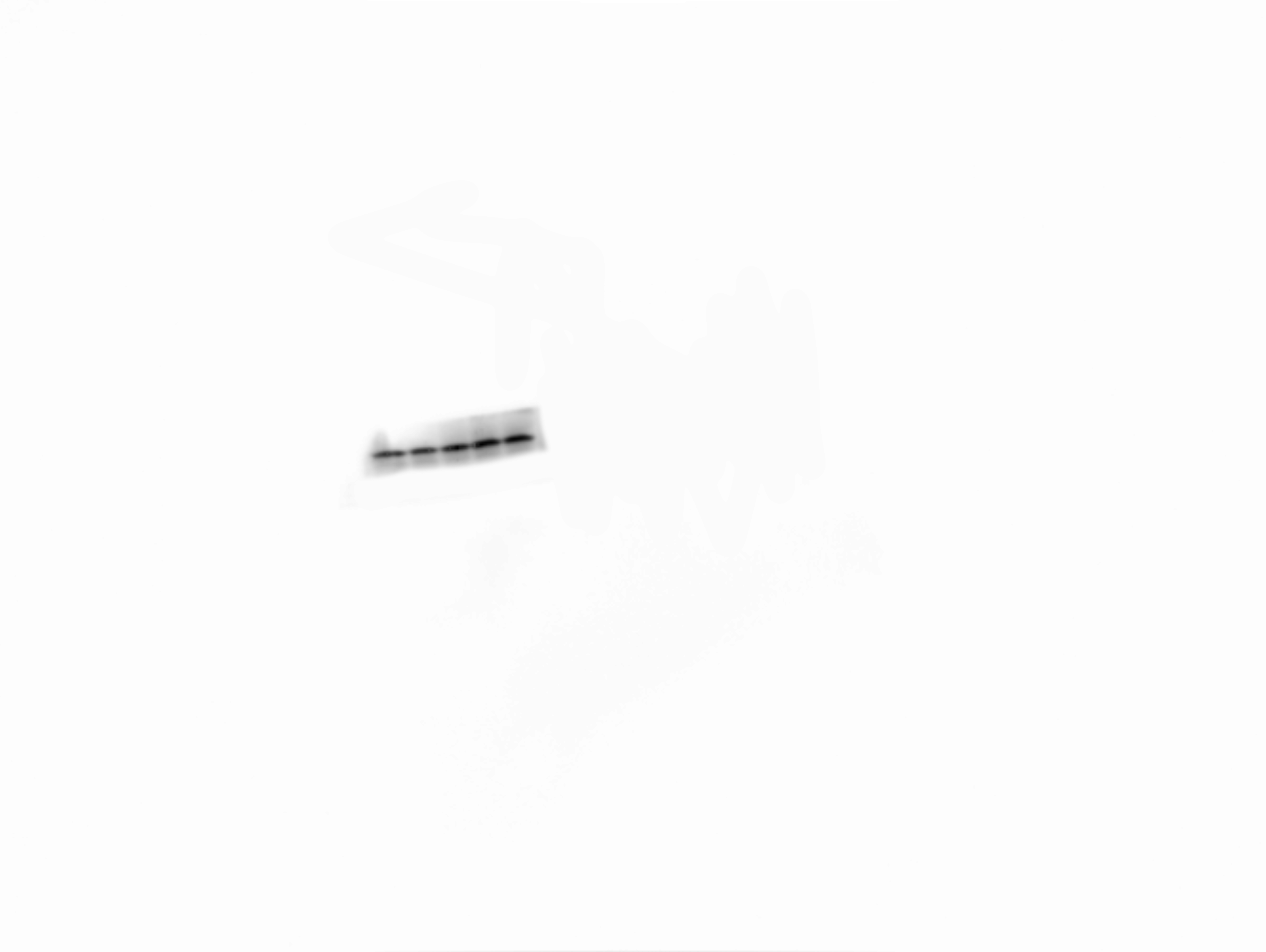

Supplement: S2 File — Original picture of the western blot experiments in the manuscript. (ZIP) [file pone.0274620.s002.zip › S2. blot results/Fig 3/VEGF/3model/5.tif]

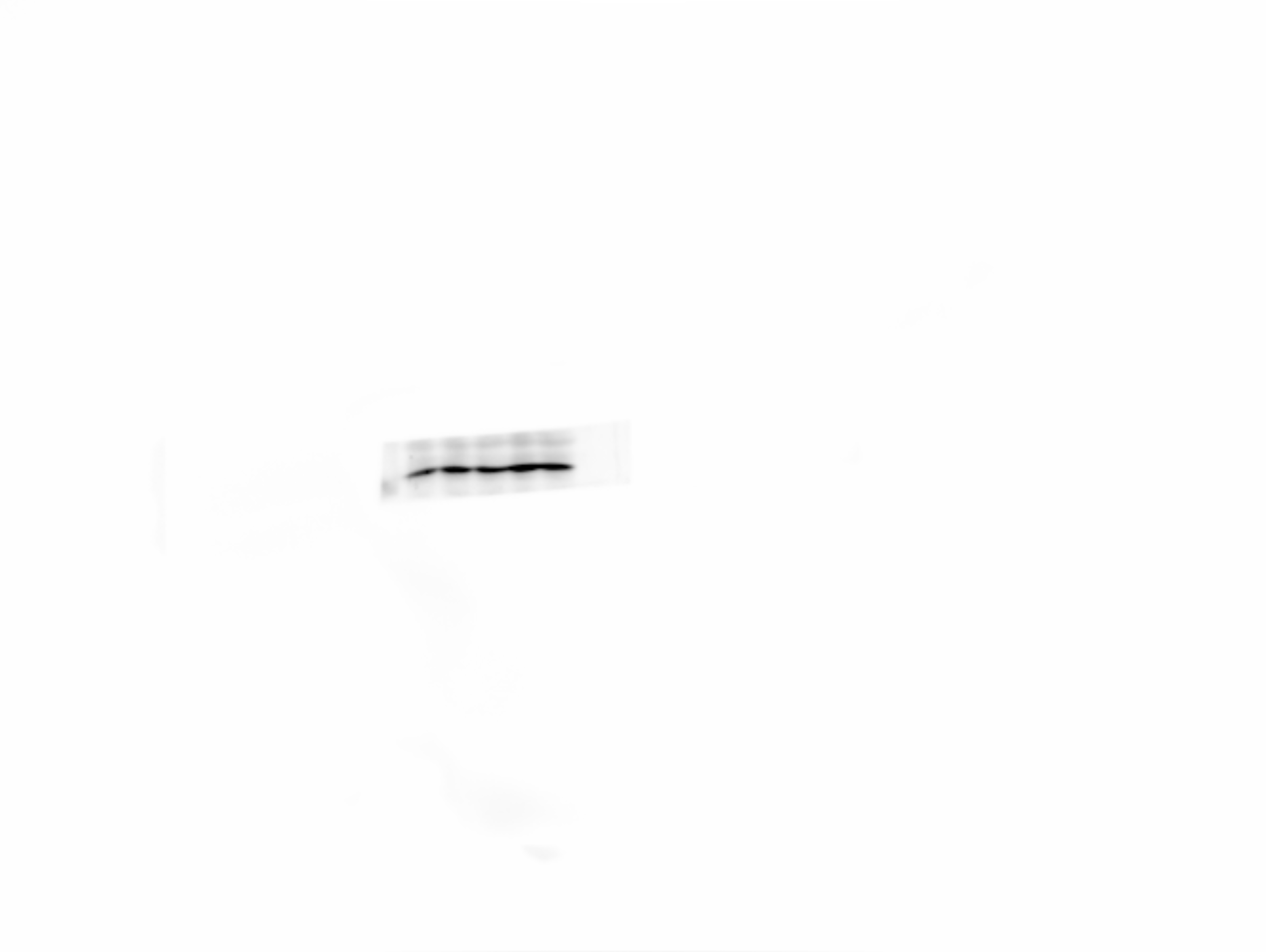

Supplement: S2 File — Original picture of the western blot experiments in the manuscript. (ZIP) [file pone.0274620.s002.zip › S2. blot results/Fig 3/VEGF/4EA/1.tif]

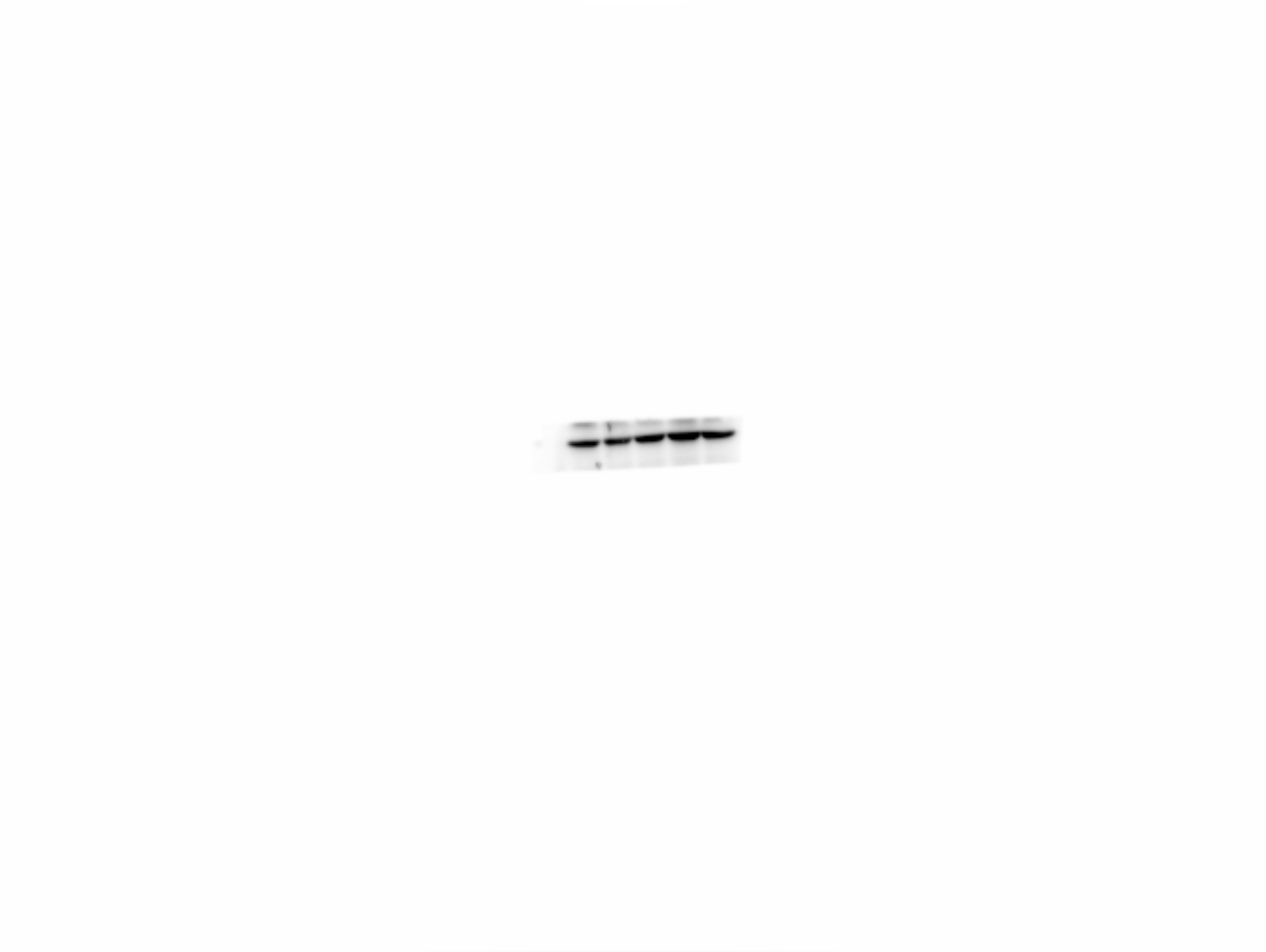

Supplement: S2 File — Original picture of the western blot experiments in the manuscript. (ZIP) [file pone.0274620.s002.zip › S2. blot results/Fig 3/VEGF/4EA/2.tif]

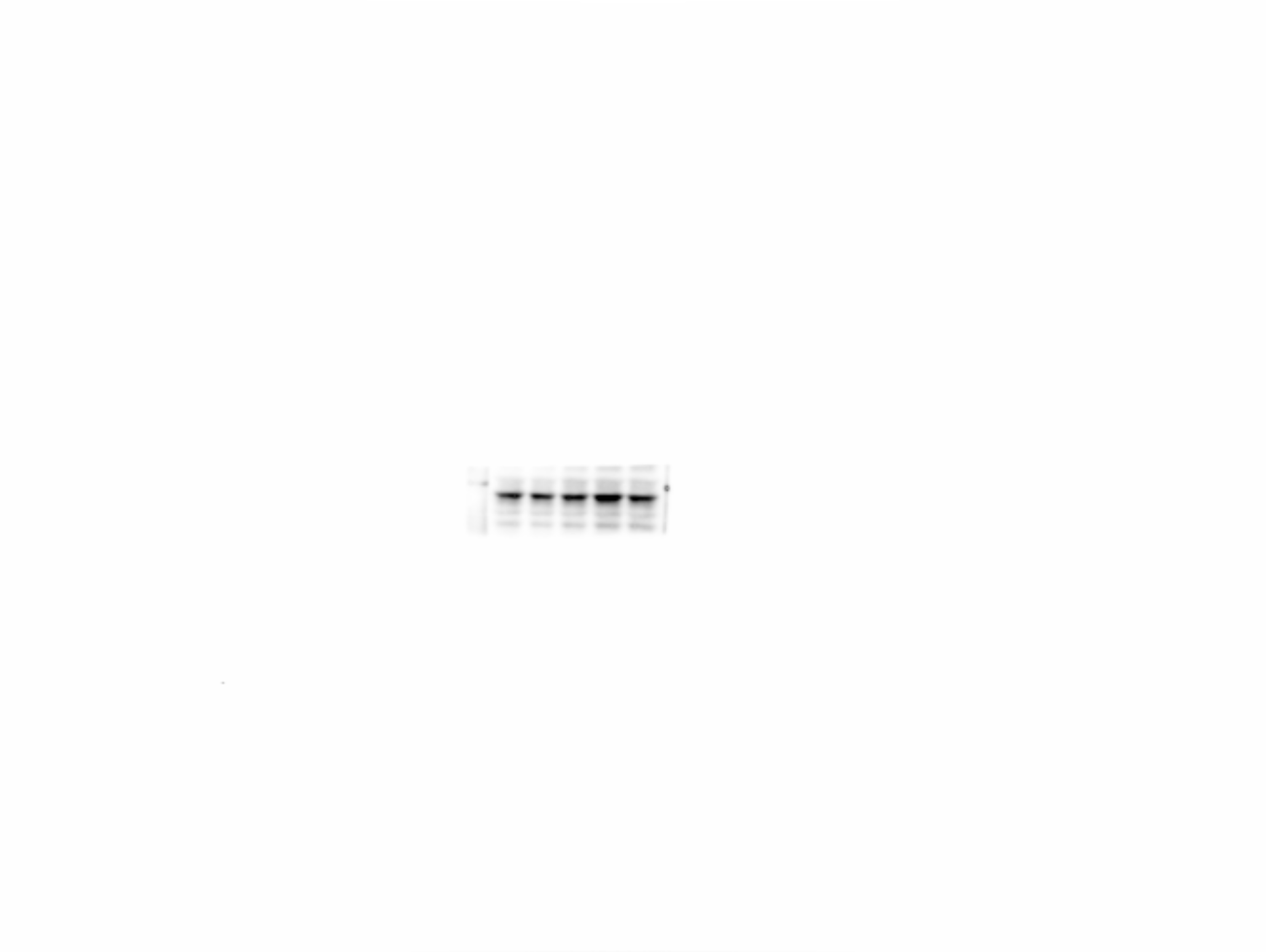

Supplement: S2 File — Original picture of the western blot experiments in the manuscript. (ZIP) [file pone.0274620.s002.zip › S2. blot results/Fig 3/VEGF/4EA/3.tif]

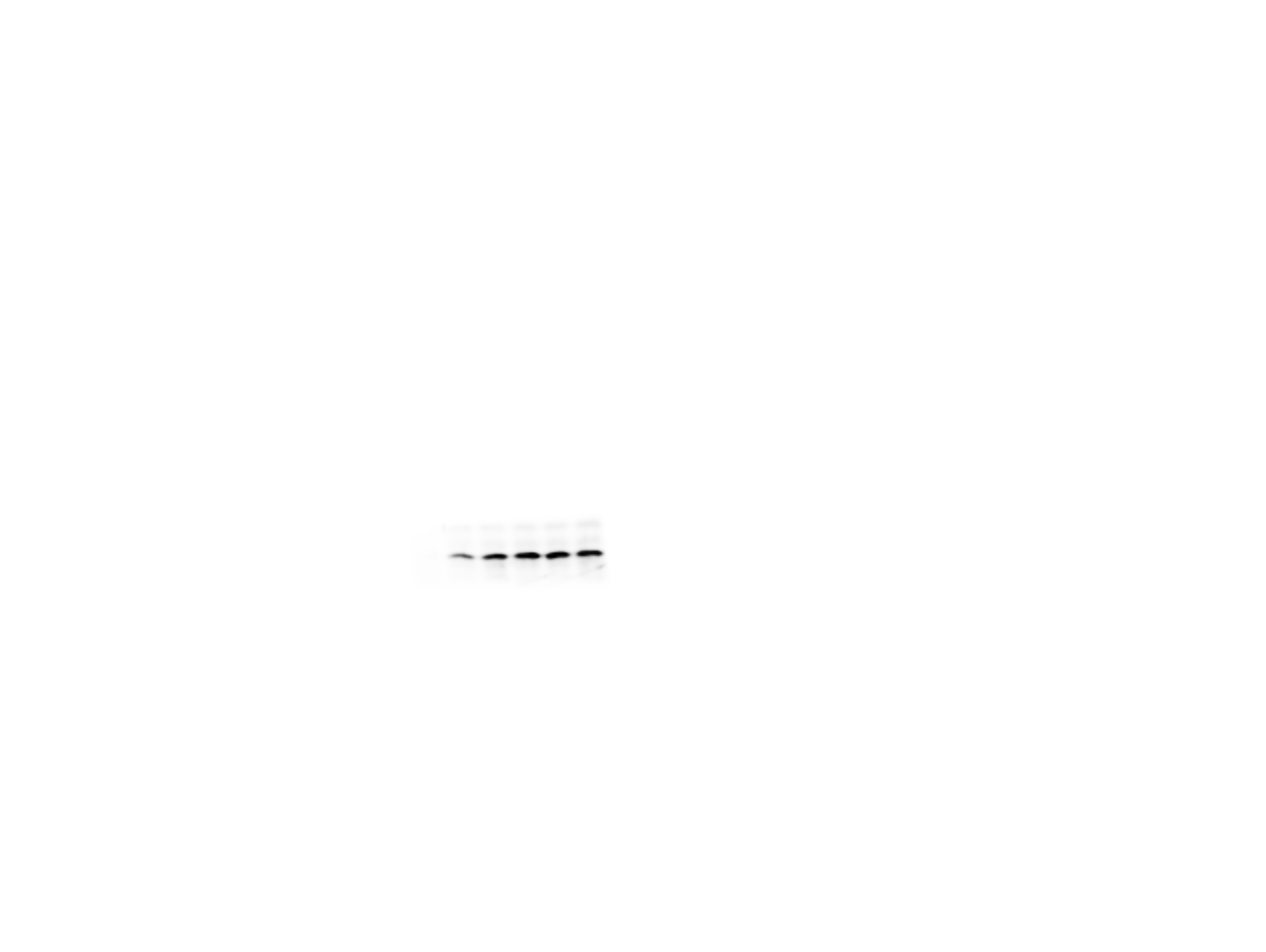

Supplement: S2 File — Original picture of the western blot experiments in the manuscript. (ZIP) [file pone.0274620.s002.zip › S2. blot results/Fig 3/VEGF/4EA/4.tif]

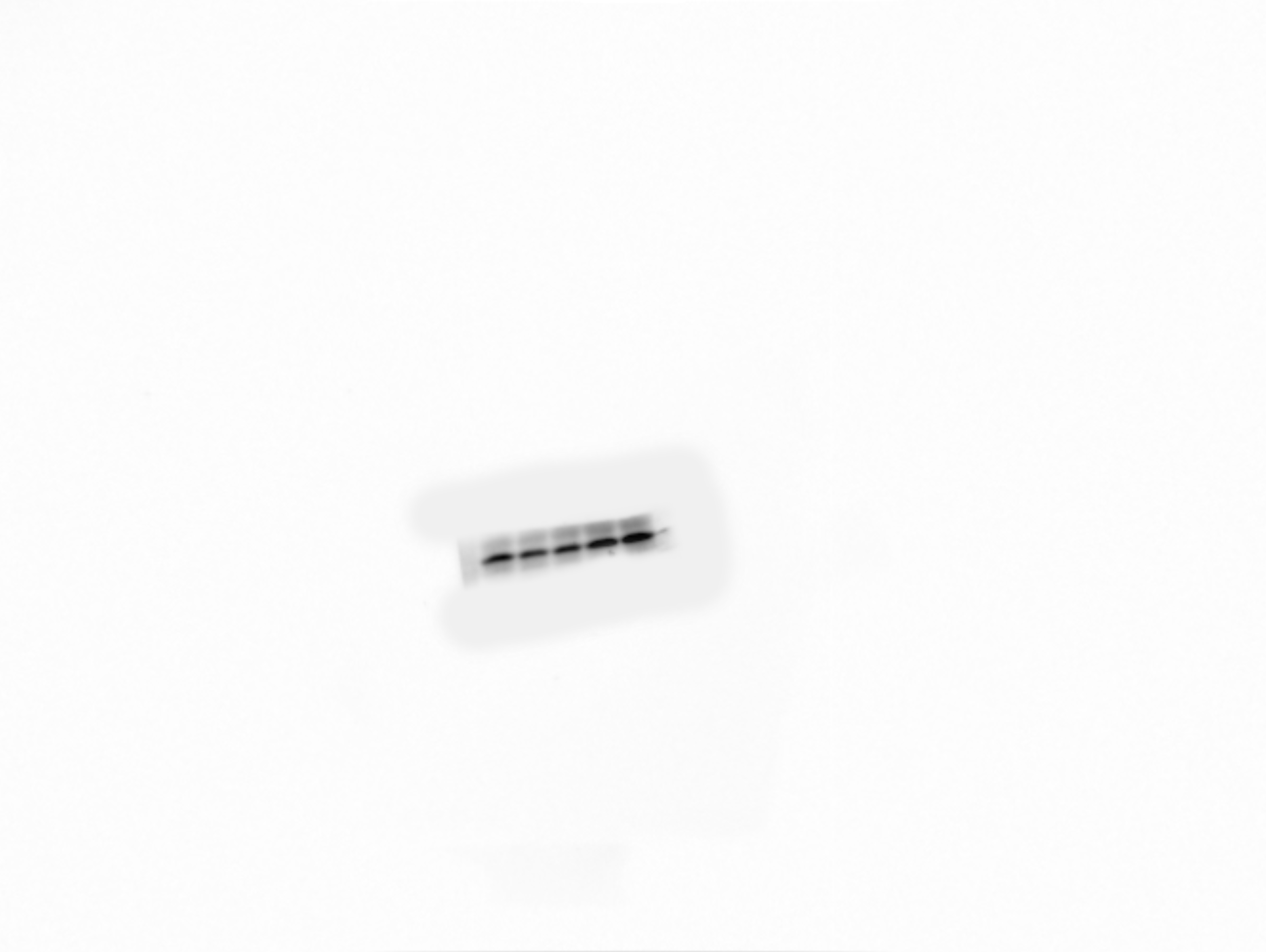

Supplement: S2 File — Original picture of the western blot experiments in the manuscript. (ZIP) [file pone.0274620.s002.zip › S2. blot results/Fig 3/VEGF/4EA/5.tif]
